# Inhibition of Protein *N-*Glycosylation Blocks SARS-CoV-2 Infection

**DOI:** 10.1128/mbio.03718-21

**Published:** 2022-02-15

**Authors:** Aitor Casas-Sanchez, Alessandra Romero-Ramirez, Eleanor Hargreaves, Cameron C. Ellis, Brian I. Grajeda, Igor L. Estevao, Edward I. Patterson, Grant L. Hughes, Igor C. Almeida, Tobias Zech, Álvaro Acosta-Serrano

**Affiliations:** a Department of Vector Biology, Liverpool School of Tropical Medicine, Liverpool, United Kingdom; b Department of Tropical Disease Biology, Liverpool School of Tropical Medicine, Liverpool, United Kingdom; c Department of Molecular and Cellular Physiology, University of Liverpool, Liverpool, United Kingdom; d Department of Biological Sciences, Border Biomedical Research Center, University of Texas at El Paso, El Paso, Texas, USA; e Department of Biological Sciences, Brock University, St. Catharines, Canada; Catholic University of America

**Keywords:** SARS-CoV-2, COVID-19, coronavirus, *N-*glycosylation, viral infection, antiviral agents, glycosylation

## Abstract

Severe acute respiratory syndrome coronavirus-2 (SARS-CoV-2) extensively *N-*glycosylates its spike proteins, which are necessary for host cell invasion and the target of both vaccines and immunotherapies. These *N-*glycans are predicted to modulate spike binding to the host receptor by stabilizing its open conformation and host immunity evasion. Here, we investigated the essentiality of both the host *N*-glycosylation pathway and SARS-CoV-2 *N-*glycans for infection. Ablation of host *N-*glycosylation using RNA interference or inhibitors, including FDA-approved drugs, reduced the spread of the infection, including that of variants B.1.1.7 (Alpha), B.1.351 (Beta), P.1 (Gamma) and B.1.617.2 (Delta). Under these conditions, cells produced fewer virions and some completely lost their infectivity. Furthermore, partial enzymatic deglycosylation of intact virions showed that surface-exposed *N-*glycans are critical for cell invasion. Altogether, we propose protein *N-*glycosylation as a targetable pathway with clinical potential for treatment of COVID-19.

## INTRODUCTION

The severe acute respiratory syndrome coronavirus-2 (SARS-CoV-2), causing the coronavirus disease-19 (COVID-19) pandemic, is a positive single-stranded RNA enveloped betacoronavirus (1). Its homotrimeric spike glycoprotein has been extensively studied, as it is the target of most current vaccines due to its surface exposure, immunogenicity, and essential role in infection. It is well established that the main route for SARS-CoV-2 to infect the host cell is via attachment to the host receptor angiotensin-converting enzyme 2 (ACE-2) through the spike’s receptor-binding domain (RBD), followed by membrane fusion and invasion (2, 3). Both spike and ACE-2 are post-translationally modified by the addition of a variable number of *N-*glycans. While ACE-2 contains seven occupied *N-*glycosylation sites (4), the spike protein is heavily *N-*glycosylated with up to 66 oligosaccharides (22 per monomer) (5, 6), including vaccine-derived spikes (7, 8). Molecular dynamics simulations predict critical roles for spike *N-*glycans in stabilizing the trimer and the RBD open conformation (5, 6, 9–11). In addition, they are suggested to be important for binding to ACE-2 (12, 13), and mutations in specific *N-*glycosylation sites (i.e., N331 and N343) drastically reduced pseudotyped virus invasion (14). Moreover, spike *N-*glycans are flexible and can occupy a large surface, hiding epitopes from antibodies to escape the host immune response (14–16). Considering the important roles protein *N*-glycosylation has in the biology of SARS-CoV-2, the *N-*glycosylation pathway serves as an attractive target to develop new approaches against COVID-19. However, little is known about the functional role of *N-*glycosylation during a real SARS-CoV-2 infection. A few studies have shown the efficacy at reducing viral infection using inhibitors of the endoplasmic reticulum (ER) α-glucosidases (17–20), which are essential enzymes for the maturation of *N-*glycan structures. ER α-glucosidases have been previously validated as targets for antivirals of broad spectrum, as they are important in the life cycle of enveloped viruses (21, 22). In addition, inhibition of Golgi’s α-mannosidase I and the knockout of *N*-acetylglucosaminyl-transferase I (GnT-I) MGAT1 decreased pseudovirus infection (23) or resulted in reduced spike binding (12). Most of these findings are based on work using heterologous spike expression, pseudotyped vesicular stomatitis virus (24), and computational models, but little is known from experimental evidence using SARS-CoV-2 virions. In this study, we investigated the essentiality of host *N-*glycosylation and SARS-CoV-2 *N-*glycans for infection *in vitro*. We show evidence that ablation of host *N-*glycosylation reduces the spread of the infection *in vitro*, suggesting this is a druggable pathway against COVID-19.

## RESULTS

### Genetic ablation of host *N-*glycosylation reduces SARS-CoV-2 infection.

**FIG 1:**
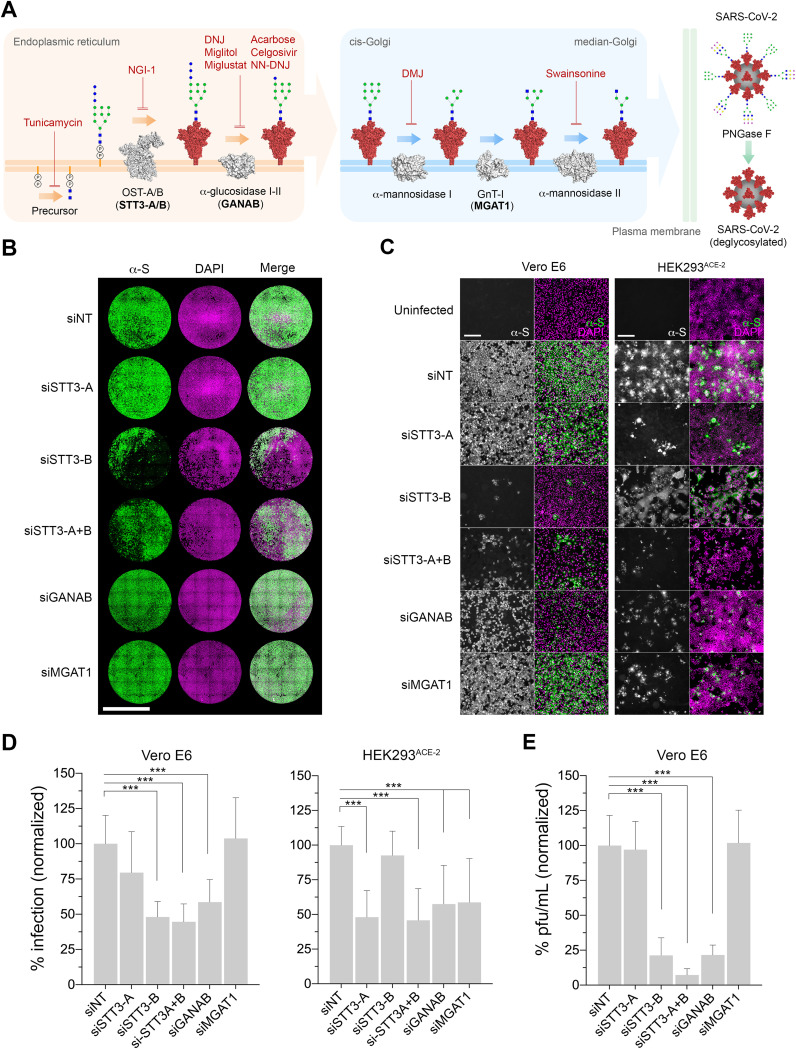
Genetic ablation of host *N-*glycosylation reduces SARS-CoV-2 infection. (A) Schematic of the key steps in the *N-*glycosylation pathway targeted in this study. The precursor *N-*glycan is first synthesized in the ER and transferred to the SARS-CoV-2 spike protein (red) by the OST’s catalytic subunit, STT3 (isoforms A and B). The *N-*glycans are further processed by the α-glucosidases I and II (GANAB catalytic subunit) before the glycoprotein is exported to the Golgi apparatus, where the glycans are modified by the α-mannosidase I, GnT-I (MGAT1), and α-mannosidase II. Mature glycosylated virions egress to the extracellular space, where they can be artificially deglycosylated using PNGase F. Glycosylation enzymes, gray; glycosylation inhibitors, red text; glycosylation enzymes targeted by siRNA, boldface text. *N-*glycans are represented using CFG nomenclature. Blue square, *N*-acetylglucosamine; green circle, mannose; blue circle, glucose; yellow circle, galactose; magenta diamond, sialic acid; virion *N-*glycan structures are representative only. (B) Representative whole-well scans of confluent Vero E6 monolayers transfected either with siNT (nontargeting control) or siRNAs targeting STT3-A, STT3-B, STT3A+B, GANAB, and MGAT1 and infected with SARS-CoV-2 (MOI of 0.05, 24 h). Cells immunostained using anti-spike antibodies (green), counterstained with DAPI DNA staining (magenta), and merged. Scale bar, 5 mm. (C) Representative images of Vero E6 and HEK293^ACE-2^ siRNA cells infected with SARS-CoV-2 (MOI of 0.05 for 24 h, Vero; MOI of 0.1 for 24 h, HEK293). Anti-spike, gray; merge, green; with DAPI, magenta. Scale bars, 200 μm. (D) Percentage of Vero E6 and HEK293^ACE-2^ infected cells (anti-spike positive) in panel A, normalized to siNT. Three technical replicates and four and three biological replicates were done for Vero and HEK cells, respectively. (E) Infectious viral particles (PFU/mL) in supernatants from infected Vero E6 siRNA cells in panel A, normalized to siNT. Bars indicate mean values, error bars represent ± standard deviations (SD), and asterisks indicate significance (*P < *0.001).

Considering the potential important roles of spike *N-*glycans, we hypothesized that the partial or complete inhibition of the host *N-*glycosylation pathway interferes with the normal development of SARS-CoV-2 infection (Fig. 1A). This may prevent the invasion of cells with glycosylation defects in key surface receptors, affect the normal production of viral proteins and virions, and/or hamper their capacity to infect further host cells. In both Vero E6 (African green monkey kidney cells) and HEK293^ACE-2^ (lentiviral overexpression of ACE-2 in human embryonic kidney cells), we used RNA interference (RNAi) to knock down the following key enzymes in the *N-*glycosylation pathway prior to SARS-CoV-2 infection: (i) oligosaccharyltransferase catalytic subunit STT3 (isoforms A, B, and both A and B) to abolish transfer of *N-*glycan precursors to proteins in the ER; (ii) the GANAB catalytic subunit of the ER α-glucosidase II to prevent the deglucosylation of immature *N-*glycans attached to proteins; and (iii) Golgi MGAT1 to block the formation of hybrid and complex *N-*glycans (Fig. 1A). As measured by quantification of anti-spike-positive cells (Fig. 1B to D), knockdown of both *STT3* isoforms (si*STT3-A*+*B*) led to an average infection reduction of 55.4% in Vero and 54.2% in HEK cells. Specific knockdowns were then performed separately to determine the contribution of each STT3 isoform. While si*STT3-A* showed a profound inhibitory effect in HEK cells (52% reduction), it did not significantly reduce viral infection in Vero cells. On the contrary, si*STT3-B* reduced the infection in Vero cells down to 48% but did not protect HEK cells from infection, likely due to inefficient knockdown (see Fig. S1 in the supplemental material). Reduction of *GANAB* consistently resulted in less infected Vero and HEK cells, although the effect was not as pronounced as that in si*STT3* (41.4% and 42.5%, respectively). Lastly, while si*MGAT1* had no effect in Vero cells, it showed some protection in HEK cells (58.8% infection). We further validated these results by quantifying the amount of infectious viral particles released by infected Vero siRNA cells (Fig. 1E), which significantly decreased upon knockdown of STT3-B, STT3-A+B, and GANAB, in correlation with the infectivity results. Knockdown levels were determined for each group and biological replicate by immunoblotting using specific antibodies (Fig. S1), showing that only HEK cells seemed to be consistently resistant to *STT3-B* knockdown. Hydrophilic interaction liquid chromatography-ultrahigh-performance liquid chromatography (HILIC-UPLC) analyses of released *N-*glycans from short interfering RNA (siRNA) cells suggests an expected overall *N-*glycan downregulation in si*STT3* and si*GANAB* cells as well as the accumulation of MGAT1 substrate M5 glycan (Man_5_GlcNAc_2_) in si*MGAT1* cells (see Fig. 3 and Fig. S3 and Data Set S1).

10.1128/mbio.03718-21.1 FIG S1 Representative Western blotting of lysates from either Vero E6 (A) or HEK293^ACE-2^ (B) cells treated with siRNA targeting glycosylation genes (i.e., STT3-A, STT3-B, STT3-A+B, GANAB, and MGAT1). Lysates probed with specific antibodies against the targeted protein were compared to the nontargeted control (siNT). Estimated apparent molecular weights are annotated. GAPDH was used as a loading control. (C) Quantification of the percentage of remaining targeted protein compared to the corresponding siNT control, normalized against GAPDH and based on band intensities. Four biological replicates for Vero E6 (except siSTT3-A+B, with three) and three biological replicates for HEK293^ACE-2^ cells. Download FIG S1, TIF file, 1.5 MB. Copyright © 2022 Casas-Sanchez et al. 2022 Casas-Sanchez et al. https://creativecommons.org/licenses/by/4.0/ This content is distributed under the terms of the Creative Commons Attribution 4.0 International license.

10.1128/mbio.03718-21.3 FIG S3 HILIC-UPLC chromatograms plotting fluorescence intensity total counts over time (minutes) of *N-*glycans released from (A) untreated uninfected HEK293^ACE-2^ and Vero E6 cells; (B) uninfected HEK293^ACE-2^ cells treated with siRNAs targeting glycosylation genes (STT3-A, STT3A+B, GANAB and MGAT1); (C) Vero E6 cells pretreated overnight with several glycosylation inhibitors (NGI-1 at 5 mM, miglustat at 100 mM, miglitol at 100 mM, celgosivir at100 mM) and infected (INF) or not (uninfected) with SARS-CoV-2 (MOI of 0.001, 48 h). Average glucose units (GU) are indicated in the top bar; peaks are annotated with glycan possible structures annotated on control samples (CFG nomenclature; legend in panels A and B). Glycan structures assumed from chromatographic mobility of standards and not confirmed by mass spectrometry. Peaks are identified with numbers; colors represent intensity decrease (blue) or increase (red) compared to the corresponding control sample. The procainamide molecule linked to each glycan is omitted. Download FIG S3, TIF file, 2.8 MB. Copyright © 2022 Casas-Sanchez et al. 2022 Casas-Sanchez et al. https://creativecommons.org/licenses/by/4.0/ This content is distributed under the terms of the Creative Commons Attribution 4.0 International license.

10.1128/mbio.03718-21.5 DATA SET S1 *N-*Glycoprofiling raw data and controls. Download Data Set S1, XLSX file, 0.3 MB. Copyright © 2022 Casas-Sanchez et al. 2022 Casas-Sanchez et al. https://creativecommons.org/licenses/by/4.0/ This content is distributed under the terms of the Creative Commons Attribution 4.0 International license.

### *N-*Glycosylation inhibitors are effective against SARS-CoV-2 infection *in vitro*.

**FIG 2:**
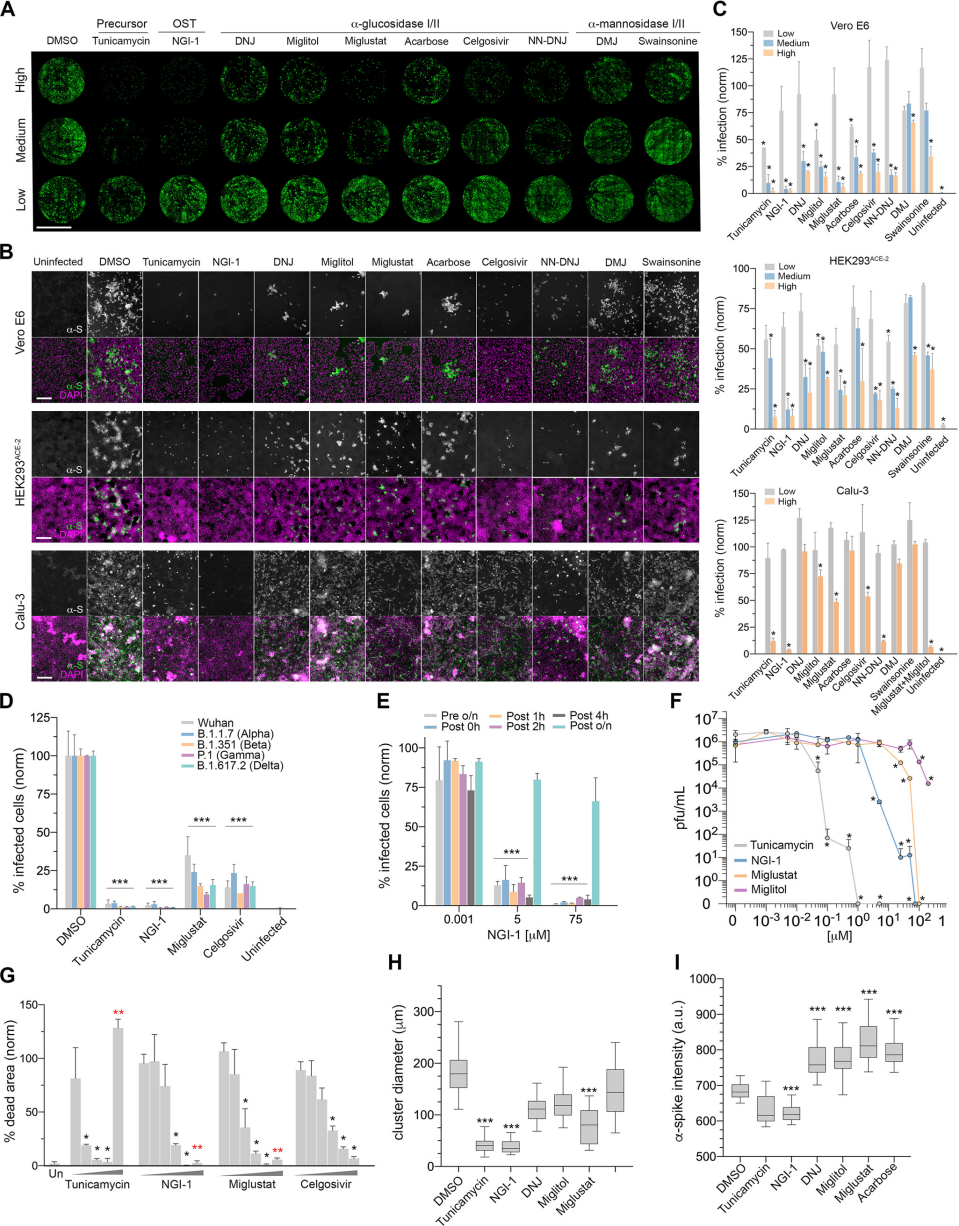
Glycosylation inhibitors reduce the spread of SARS-CoV-2 infection. (A) Representative whole-well scans of confluent Vero E6 monolayers treated with either high, medium, or low dose of inhibitors and infected with SARS-CoV-2 (MOI of 0.05, 24 h). Cells were immunostained using anti-spike antibodies (green). Scale bar, 5 mm. (B) Representative images of Vero E6 in panel A treated with high-dose inhibitors, HEK293^ACE-2^ cells (MOI of 0.1, 24 h), and Calu-3 cells (MOI of 0.1, 24 h). Anti-spike, gray; merge, green; with DAPI, magenta. Scale bars, 200 μm. (C) Percentage of infected (anti-spike positive) cells in panels A and B, normalized to DMSO controls. Two technical replicates and two biological replicates were done. Asterisks indicate significance (*P < *0.05). Inhibitor concentrations (low, medium, ands high, in μM) were the following: tunicamycin (0.001, 0.01, 0.05); NGI-1 (0.001, 5, 75); DNJ (0.01, 60, 120); miglitol (0.01, 50, 200); acarbose (0.01, 100, 300); miglustat (0.01, 50, 100); celgosivir, swainsonine, NN-DNJ, and DMJ (0.01, 25, 100). (D) Percentage of infected Vero E6 cells pretreated with high-dose inhibitors and infected with either the Wuhan (Liverpool) isolate or variants B.1.1.7, B.1.351, P.1, and B.1.617.2, normalized to DMSO controls (MOI of 0.05, 24 h). (E) Percentage of infected Vero E6 cells (MOI of 0.05, 24 h) treated with inhibitors at different time points in relation to inoculation (preincubation overnight [o/n]; 0 h coincubation; and 1, 2, 4 h and overnight postincubation) with 0.001, 5, or 75 μM NGI-1, normalized to DMSO controls. (F) Viral titers in Vero E6 supernatants treated with inhibitors and infected with SARS-CoV-2 (MOI of 0.001, 48 h); dots indicate mean values from two technical replicates and two biological replicates. (G) Area percentage covered by dead Vero E6 cells treated with inhibitors (low to high dose) and infected with SARS-CoV-2 (MOI of 0.001, 48 h), normalized to DMSO controls. Two technical replicates and two biological replicates were done; two red asterisks indicate death by toxicity. (H) Box plot of cluster diameter (μm) of infected Vero E6 cells in panels A and B treated with high-dose inhibitors; horizontal lines represent mean values, and whiskers represent ± confidence intervals. (I) Fluorescence intensity of anti-spike antibodies in infected Vero E6 cells in panels A and B treated with high-dose inhibitors. Bars indicate mean values; error bars represent ± SD; and asterisks indicate significance (*P < *0.05).

**FIG 3:**
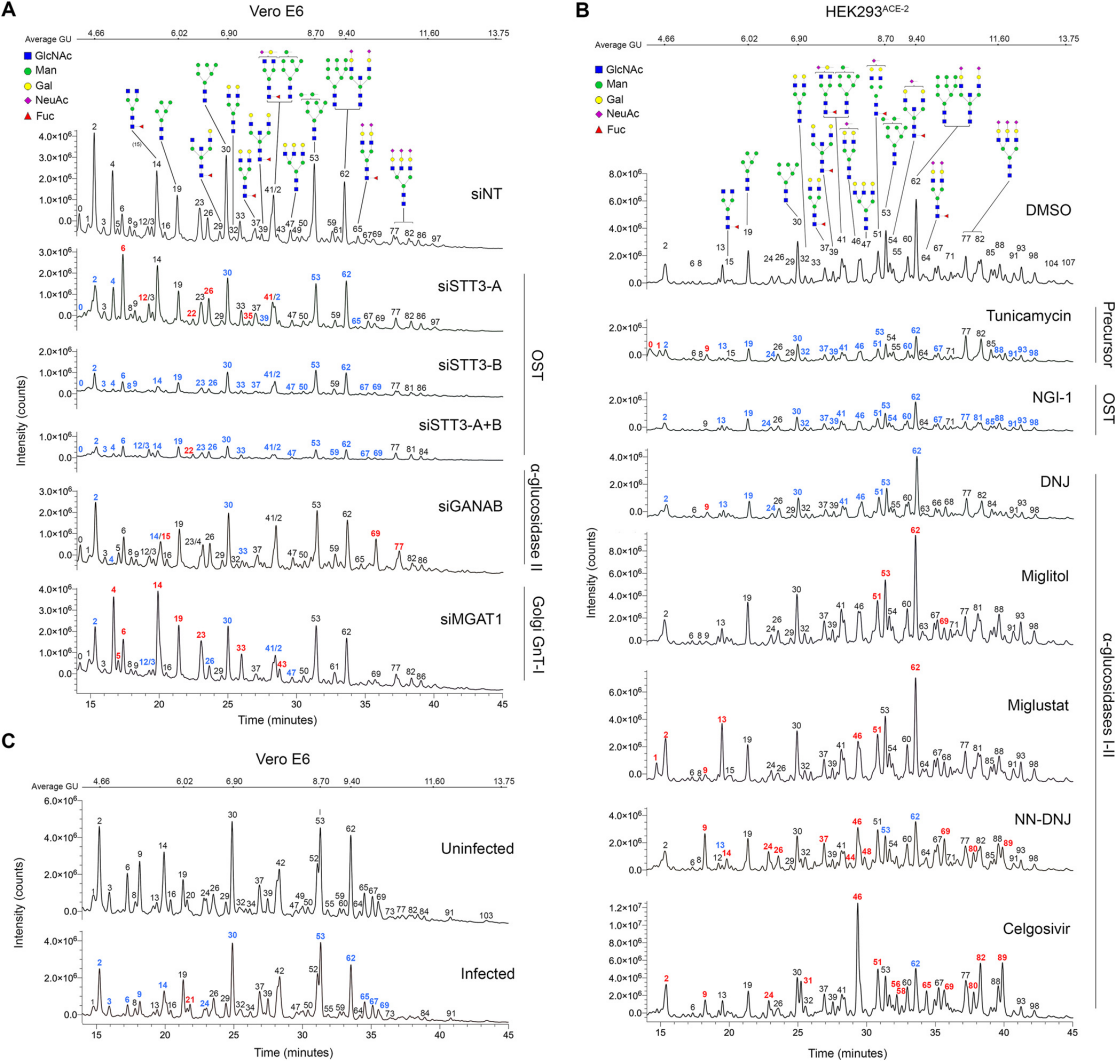
*N-*Glycoprofiling confirms glycosylation changes in siRNA- and drug-treated cells. (A) HILIC-UPLC chromatograms plotting fluorescence intensity total counts over time of released *N-*glycans from Vero E6 cells treated with siNT (control), siSTT3-A, siSTT3-B, siSTT3-A+B, siGANAB, and siMGAT1. (B) Chromatograms of *N-*glycans from HEK293^ACE-2^ cells treated with glycosylation inhibitors (tunicamycin, 0.1 μM; NGI-1, 5 μM; DNJ, 100 μM; Miglitol, 100 μM; miglustat, 100 μM; NN-DNJ, 100 μM; and celgosivir, 100 μM) or DMSO. (C) Chromatograms of *N-*glycans from Vero E6 cells either infected with SARS-CoV-2 or noninfected. Glycan are structures in CFG nomenclature; structures are assumed from chromatographic mobility of standards and not confirmed by mass spectrometry. Peaks are identified with numbers; colors represent intensity decrease (blue) or increase (red) compared to the corresponding control sample. The procainamide molecule linked to each glycan is omitted.

Having identified several enzymes whose reduction in expression lowered SARS-CoV-2 infections, we then investigated whether chemical inhibition of these and other enzymes throughout the *N-*glycosylation pathway also promotes protection against the virus. We tested several compounds known to inhibit important *N-*glycosylation steps at concentrations we found in pilot experiments to be at the low or high end of the effective range (Fig. 1A). To emulate the effects of si*STT3* knocking down the early pathway, we used tunicamycin to block formation of the *N*-glycan intermediate GlcNAc_2_-dolichol phosphate and NGI-1, which inhibits STT3 directly. Further down in the pathway, as in *si*GANAB cells, we inhibited the ER α-glucosidases I and II using the iminosugars 1-deoxynojirimycin (DNJ), miglitol (25), *N*-butyl-deoxynojirimycin (miglustat [26]), *N*-nonyldeoxynojirimycin (NN-DNJ), celgosivir (27), and acarbose (28). Late in the pathway and resembling *si*MGAT1 treatment, we tested the efficacy of deoxymannojirimycin (DMJ) and swainsonine to inhibit the Golgi α-mannosidases I and II, respectively. Preincubation of Vero E6 and HEK293^ACE-2^ cells and the more relevant cell line Calu-3 (human lung epithelial) with most inhibitors prior to SARS-CoV-2 inoculation resulted in a significant reduction in infection compared to untreated cells (Fig. 2A to C). Importantly, NGI-1, miglustat, and celgosivir were equally effective at reducing the infection in both Vero and HEK cells by SARS-CoV-2 variants of concern, B.1.1.7 (Alpha), B.1.351 (Beta), P.1 (Gamma), and B.1.617.2 (Delta) (Fig. 2D and Fig. S2A). These protective effects were observed when the inhibitors were added alongside the virus and up to 4 h after virus inoculation (Fig. 2E), regardless of the viral inoculum except at the highest multiplicity of infection (MOI) used (Fig. S2B and C). This suggests that glycosylation inhibitors are effective at reducing the spread of the infection but not the initial invasion round. For selected inhibitors, we quantified infectious viral titers in cell supernatants (Fig. 2F) and measured the proportion of cells showing cytopathic effects (CPE) as a result of the infection (Fig. 2G). While many untreated control cells became infected and arranged in clusters, both tunicamycin and NGI-1 provided the highest protection rates against SARS-CoV-2 in Vero cells (97.3% and 97%), HEK cells (92.3% and 92%), and Calu cells (87.5% and 93.7%) (Fig. 2A to C), showing only a few isolated infected cells (Fig. 2B and H). They also protected cells against CPE (94% and 99.5%) (Fig. 2G) and, at high doses, led to undetectable levels of infectious particles in cell supernatants (Fig. 2F). Inhibitors of ER α-glucosidases proved to be less effective and more variable. The most effective ones (i.e., miglustat, celgosivir, and NN-DNJ) showed reduced overall infections in Vero, HEK, and Calu cells (Fig. 2A to C), smaller clusters of infected cells (Fig. 2H), CPE protection, and a moderate virus titer reduction in supernatants (Fig. 2F to G). DNJ, miglitol, and acarbose showed little effect at the highest doses only. While the protection given by tunicamycin and NGI-1 could be attributed to the absence of *N*-glycans, the effects produced by α-glucosidase inhibitors could result from alterations in *N-*glycan structure. In addition, increased binding of anti-spike in drug-treated cells (Fig. 2I) suggests an accumulation of viral glycoproteins in the ER as a consequence of the inhibition of the calnexin/calreticulin cycle (29). Inhibition of the Golgi α-mannosidases using DMJ and swainsonine produced no effect in Vero cells but did reduce HEK cell infections, resembling the phenotypes seen in si*MGAT1* (i.e., only effective in HEK cells) (Fig. 1). Differences in baseline *N-*glycosylation profiles between Vero and HEK cells (Fig. S3A, File S1) as well as the differential expression of glycosylation enzymes may account for variations between cell lines, particularly the Golgi endo-α-1,2-mannosidase (MANEA) alternative pathway (30) that can rescue glycoproteins improperly glycosylated by malfunctioning ER α-glucosidases. Cell viability assays were run in parallel to rule out poor metabolism or toxicity as the main cause of infection decrease (Fig. 2D), although drug off-target effects may play unexpected roles at high doses. The corresponding changes in protein *N-*glycosylation upon drug treatment were analyzed by HILIC-UPLC *N-*glycoprofiling (Fig. 3 and Fig. S3B, File S1). Lastly, we investigated whether two inhibitors combined can increase their potency (Fig. S2E). While the combination of tunicamycin and NGI-1 did not significantly improve their respective individual efficacies, simultaneous treatment of NGI-1 with any α-glucosidase inhibitor promoted higher protection rates than NGI-1 or α-glucosidase inhibitors alone. Furthermore, some combinations of α-glucosidase inhibitors were particularly efficacious (e.g., miglustat combined with miglitol), and combinations of those with α-mannosidase inhibitors also resulted in augmented efficacies.

10.1128/mbio.03718-21.2 FIG S2 (A) Percentage of infected HEK293^ACE-2^ cells pretreated with high doses of glycosylation inhibitors and inoculated with either SARS-CoV-2 Wuhan (Liverpool) isolate or the variants B.1.1.7, B.1.351, and P.1, normalized to DMSO controls (MOI of 0.05, 24 h). (B) Percentage of infected Vero E6 cells inoculated with SARS-CoV-2 (MOI of 1; 24 h) and treated with either NGI-1 at 0.001, 5, or 75 μM, normalized to DMSO controls. NGI-1 was added either 16 h before virus inoculation (Pre o/n), during inoculation (Post 0h), or 1, 2, 4, and 16 h after inoculation (Post 1h, 2h, 4h, o/n). (C) Percentage of infected Vero E6 cells at 24 h postinoculation, pretreated with DMSO or 0.001, 5, or 75 μM NGI-1 and inoculated with SARS-CoV-2 at an MOI of 0.05, 1, or 10. (D) AlamarBlue toxicity assays (percentage toxicity) on Vero E6 cells incubated with a range of inhibitors at different doses (μM) for 48 h. (E) Percentage of infected cells pretreated overnight with combinations of glycosylation inhibitors (doses annotated in graph) and inoculated with SARS-CoV-2 (MOI of 0.05, 24 h), normalized to DMSO controls. Bars represent mean values; error bars indicate +SD; asterisks indicate significance (*P < *0.05). Download FIG S2, TIF file, 1.1 MB. Copyright © 2022 Casas-Sanchez et al. 2022 Casas-Sanchez et al. https://creativecommons.org/licenses/by/4.0/ This content is distributed under the terms of the Creative Commons Attribution 4.0 International license.

### Glycan analyses confirm alterations in protein *N-*glycosylation after genetic and chemical inhibition.

To corroborate changes in protein *N-*glycosylation upon siRNA and drug treatment, we compared the *N-*glycan profiles from treated and untreated cells (Fig. 3 and Fig. S3). *N-*glycans were enzymatically released from 3 × 10^6^ cells per sample, labeled with procainamide and analyzed by HILIC-UPLC. Glycan structures were inferred from chromatographic mobility of standard. The analyzed cell types appeared to differ in their overall *N-*glycan profiles and Vero E6 appeared to produce more oligomannose and smaller *N-*glycans than larger complex- or hybrid-type ones unlike HEK293^ACE-2^ cells (Fig. S3). When STT3-A was knocked down in Vero cells, only a slight reduction (26.7%) in the overall levels of *N-*glycans was observed (Fig. S3B), potentially explaining the absence of a protective effect against SARS-CoV-2 (Fig. 1B to D). In contrast, siSTT3-A treatment in HEK cells produced a more pronounced overall downregulation of *N-*glycans (43%) similar to that in siSTT3-A+B. Both siSTT3-B and siSTT3-A+B Vero cells presented a stronger *N-*glycan overall downregulation (77.4% and 84.7%, respectively) and a significant decrease in the abundance of the most common *N-*glycans corresponding to peaks 2, 4, 19 (Man_5_GlcNAc_2_; M5 glycan), 30 (Man_6_GlcNAc_2_; M6 glycan), 42 (Man_7_GlcNAc_2_; M7 glycan), 53 (Man_8_GlcNAc_2_; M8 glycan), and 62 (Man_9_GlcNAc_2_; M9 glycan/A3G3S3). The knockdown of GANAB and MGAT1 barely reduced the total amount of *N-*glycans (22% and 18%, respectively). siGANAB cells had altered the major peaks 30 (M6 glycan), 2, and 14 and completely lost peak 4. As expected, siMAGT1 cells accumulated MGAT1’s substrate M5 glycan (peak 19) as well as other smaller glycans corresponding to peaks 4, 14, 23, and 33 while reducing M6 (peak 30) and M7 (peak 42).

When either Vero or HEK cells were incubated with glycosylation inhibitors, abnormal *N-*glycosylation patterns were observed in all cases compared to those from untreated dimethyl sulfoxide (DMSO) control cells (Fig. 3 and Fig. S3). As expected, incubation of HEK cells with either tunicamycin or NGI-1 significantly reduced the overall amount of *N-*glycans (56.3% and 53.8%, respectively). On the other hand, each α-glucosidase inhibitor modified the *N*-glycan profile in a unique manner, i.e., DNJ reduced the overall glycan levels down to 67.8%, while NN-DNJ, miglustat, miglitol, and celgosivir increased it by 1.0%, 29.5%, 59.1%, and 182.6%, respectively. While miglitol barely modified the relative glycan composition, except for an increase in major peaks 51, 53, and 62, corresponding to the assigned structures FA2G2S1, M8, and M9/A3G3S3, respectively; miglustat mainly promoted an increase in peaks 2, 13, 46 (A2G2S1), 51 (FA2G2S1), 53 (M8), and 62 (M9/A3G3S3). In addition, NN-DNJ augmented the abundance of a broad range of peaks, including 9, 37, and 46 (A2G2S1) while decreasing the major peaks 13, 53 (M8), and 62 (M9). Celgosivir led to the accumulation of peak 46 (A2G2S1) at the expense of reducing the amount of M9/A3G3S3 (peak 62).

Lastly, we analyzed the *N-*glycosylation profiles of SARS-CoV-2-infected Vero E6 cells either untreated or treated with several inhibitors to determine whether viral infection has a role in modifying host protein *N-*glycosylation (Fig. S4). Infection of untreated cells led to a slight reduction (38.3%) of total *N-*glycans. It did modify the overall glycosylation profile of cells by reducing peaks 2, 6, 9, and 14 while also decreasing the amounts of M6, M8, and M9 glycan structures. Infected cells treated with inhibitors presented even greater glycosylation reductions and profile alterations, likely due to the combination of both effects.

**FIG 4:**
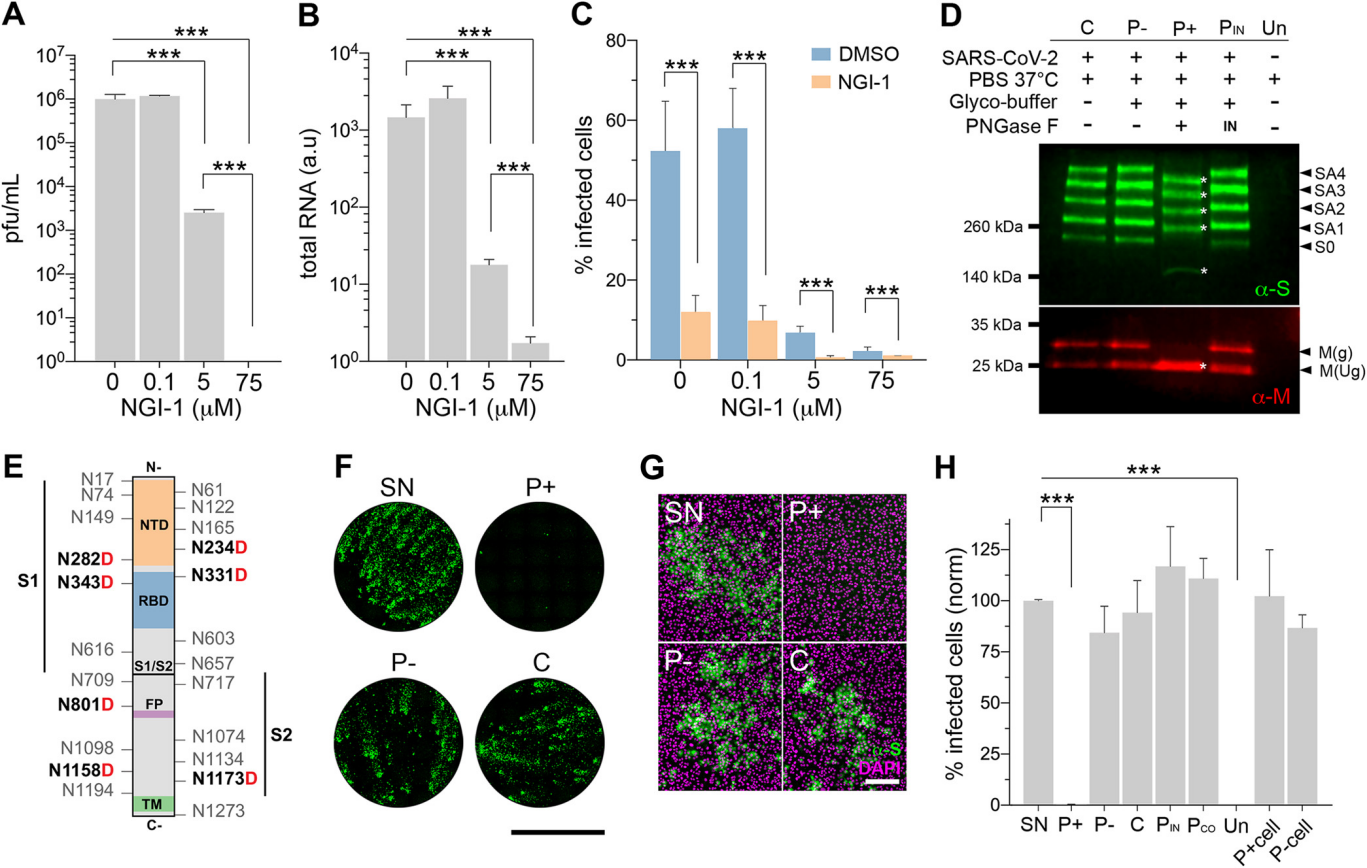
SARS-CoV-2 surface *N-*glycans are essential for infection. (A) Infective particles in infected Vero E6 supernatants (MOI of 0.001, 48 h) treated with 0, 0.1, 5, and 75 μM NGI-1. (B) Total SARS-CoV-2 RNA in supernatants in panel A. (C) Proportion of infected Vero E6 cells treated with 0, 0.1, 5, and 75 μM NGI-1 and infected with virus from either untreated (DMSO) or 5 μM NGI-1-treated cell supernatants (MOI of 0.05, 24 h); inoculum normalized by total viral RNA. (D) Western blot using anti-spike (green) and anti-M (red) antibodies on lysed native purified virions (C) of mock treated (P-), PNGase F-treated (P+), and heat-inactivated PNGase F-treated (P_IN_) cells or supernatant from uninfected cells (Un). Treatment legend (top), apparent molecular mass (left), and band identity (right) are given. Full-length spike (S0), spike aggregates (SA), and glycosylated (g) and unglycosylated (Ug) M protein are shown; asterisks highlight downshifted bands. (E) Spike protein schematic highlighting all *N-*glycosylation sites and asparagines (N) converted to aspartic acid (boldface red D) after PNGase F deglycosylation as found by mass spectrometry and spike subunits (S1, S2), N-terminal domain (NTD), receptor binding domain (RBD), fusion peptide (FP), ands transmembrane domain (TM). (F) Whole-well scans of confluent Vero E6 cells infected with SARS-CoV-2 supernatant (SN) or purified virions analyzed in panels D (MOI of 0.05, 24 h). Anti-spike antibodies, green; scale bar, 5 mm. (G) Representative images of Vero E6 cells in panel F. Anti-spike, green; merged with DAPI counterstain, magenta. Scale bar, 200 μm. (H) Percentage of infected cells in panel F, including purified virions coinoculated with active PNGase F (P_CO_) and cells either pretreated (P+_cell_) or not treated (P-_cell_) with active PNGase F prior to inoculation with native virions, normalized to SN. Bars indicate mean values, error bars show +SD, and asterisks indicate significance (*P < *0.001).

**FIG 5:**
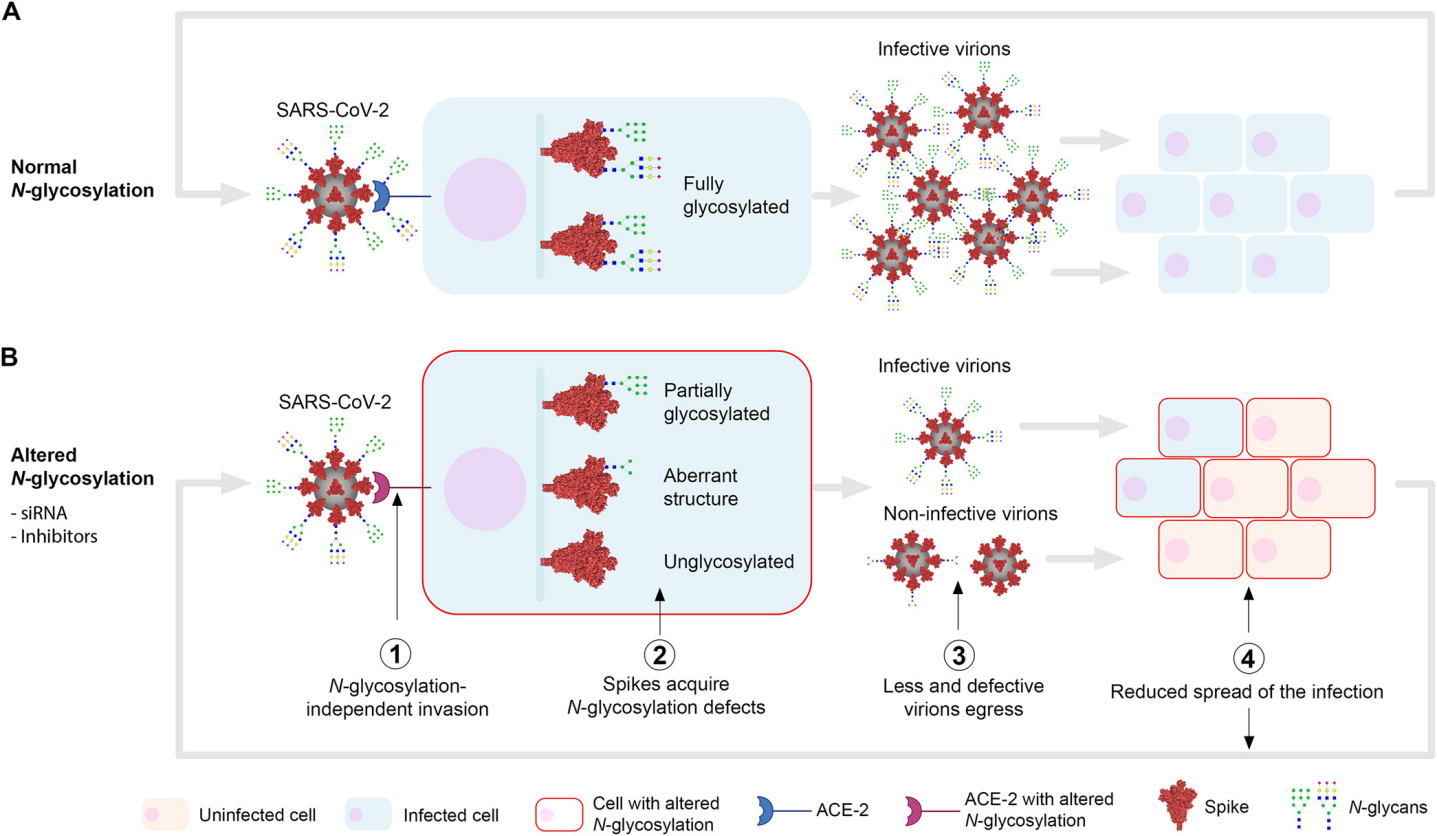
Schematic of the role of *N-*glycosylation in SARS-CoV-2 infection. Initially, SARS-CoV-2 virions can invade the host cell regardless of its *N-*glycosylation status (i.e., inhibited *N-*glycosylation, deglycosylated ACE-2) (1). Once it starts replicating, unlike in control cells (A), where spike proteins are normally *N-*glycosylated (66 sites per trimer), cells with inhibited *N-*glycosylation (B) (e.g., treated with glycosylation inhibitors or glycosylation genes downregulated using siRNAs) will produce spikes presenting partial glycosylation (<66 glycosylated sites), aberrant glycan structures, and/or no glycosylation at all (2). Improperly glycosylated spikes may misfold, accumulate in the ER, and/or degrade, leading to egression of fewer and defective virions (3). These virions become less infective or noninfective to neighboring cells as they lack spike *N-*glycan structures essential for invasion (4). Replication in these cells with inhibited *N-*glycosylation again will generate less and noninfective virions (2 to 4), therefore exponentially amplifying the reduction of the spread of the infection. *N-*glycan structures (CFG nomenclature) are representative only; the blue intracellular vertical bar represents the secretory pathway.

10.1128/mbio.03718-21.4 FIG S4 (A) Lectin blotting of virion lysates from Fig. 4D probed with concanavalin A-FITC (ConA-FITC); control purified virions (C), mock treated virions (P-), PNGase F-treated virions (*P*+), virions treated with heat-inactivated PNGase F (P_IN_), and uninfected sample (Un) were used. Membrane was stained with nigrosin for loading control, with apparent molecular weights on the left. (B) Total viral RNA quantification by qRT-PCR in supernatants from Fig. 5E. (C) Virus titers in supernatants from Fig. 5E quantified by plaque assay. Bars indicate mean values; error bars show +SD; asterisks indicate significance (*P < *0.001). (D) Blotting probed with streptavidin conjugated to Alexa Fluor 555 on lysates of Vero E6 monolayers either untreated (Pcell-) or pretreated (Pcell+) with PNGase F prior to infection and surface biotinylated. Apparent molecular weight (left) and nigrosine staining as loading control (right) are shown, and asterisks indicate biotinylated surface proteins that have downshifted after PNGase F deglycosylation. Download FIG S4, TIF file, 0.8 MB. Copyright © 2022 Casas-Sanchez et al. 2022 Casas-Sanchez et al. https://creativecommons.org/licenses/by/4.0/ This content is distributed under the terms of the Creative Commons Attribution 4.0 International license.

### SARS-CoV-2 surface *N-*glycans are essential for invasion.

We hypothesized that virions assembled in cells with altered *N-*glycosylation may present abnormal spike *N-*glycosylation profiles and consequently become less infectious. As a model, we used virions produced in Vero E6 cells treated with a subinhibitory dose of the STT3 inhibitor NGI-1. This treatment was previously observed to reduce the overall amount of *N-*glycans (Fig. 3 and Fig. S3) and the proportion of infected cells while allowing the formation of fewer infective viral particles (Fig. 2). Supernatants from infected NGI-1-treated cells contained on average of 392-fold fewer infective viral particles than untreated cells (Fig. 4A). However, quantification of total viral RNA (i.e., infective and noninfective particles) showed only 82-fold reduction (Fig. 4B). This imbalance between total and infective particles (4.8-fold) may account for virions incapable of infecting host cells as a result of missing or expressing aberrant protein *N-*glycans. To validate the hypothesis, supernatants were used to infect cells normalizing the virus inoculum based on total viral RNA. Virions released by NGI-1-treated cells still produced lower infections (4.4-fold), evidencing the existence of a subpopulation of defective virions unable to infect subsequent cells (Fig. 4C). Cells pretreated with NGI-1 showed an even stronger reduction when inoculated with virions produced from NGI-1-treated cells, suggesting a potential cumulative effect that takes place throughout replication cycles in treated cells.

To confirm that the absence of surface *N-*glycans can render viral particles noninfective, we incubated purified SARS-CoV-2 virions with peptide-N-glycosidase F (PNGase F) prior to infection (Fig. 4D to H). PNGase F removes all types of *N-*linked oligosaccharides from glycoproteins, except those with core α1,3-fucose (31, 32) (not described in mammalian cells). Under this condition, the enzyme is only expected to cleave accessible *N-*glycans from the surface-exposed viral glycoproteins spike, membrane (M), and envelope (E). Overall protein deglycosylation was analyzed by concanavalin A (ConA) blotting (Fig. S4A), a lectin that binds to a broad range of α-linked core mannose glycans. It confirmed that only PNGase F-treated virion samples (*P*+) presented less recognition by the lectin due to partial glycan removal under native conditions. We also analyzed the mobility of spike and M proteins by Western blotting (Fig. 4D). Mock-treated (P-) and control (C) virions showed a full-length spike band (S0; ∼250 kDa) and four more of higher apparent molecular mass likely corresponding to aggregates resistant to denaturing, multimeric, or more glycosylated forms. In virions treated with PNGase F (*P*+), all bands significantly downshifted due to the loss of *N-*glycans. In addition, two M bands were detected in control samples corresponding to unglycosylated (∼25 kDa) and glycosylated (∼30 kDa) forms, with the latter collapsing to ∼25 kDa upon enzyme digestion. Remarkably, deglycosylated virions (*P*+) were unable to infect host cells, while cells inoculated with either mock (P-) or control (C) virions presented infection levels comparable to those of cells inoculated with nonpurified supernatant virions (SN) (Fig. 4F to H). Quantification of total viral RNA (Fig. S4B) and infectious particles in cell supernatants (Fig. S4C) confirmed that *P*+ did not develop a SARS-CoV-2 infection. As expected, cells inoculated either with virions treated with heat-inactivated PNGase F (P_IN_) or virions treated with PNGase F only during inoculation (P_CO_) produced normal infections.

We then used liquid chromatography coupled to high-resolution tandem mass spectrometry (LC-HR-MS/MS) to identify the spike *N-*glycosylation sites whose glycans were removed by the enzyme. By looking at the conversion of asparagine into aspartate after PNGase F deglycosylation, we found five spike peptides containing seven modified sites (i.e., N234D, N282D, N331D, N343D, N801D, N1158D, and N1173D) (Fig. 4E and Data Sets S2 to S5 and Text S1). Although other undetected sites could also be deglycosylated, removal of the complex-type spike *N-*glycans on sites N234, N331, and N343 (the last two within the RBD) is likely the cause of *P*+ infection failure, as suggested in previous studies (5, 9, 11, 14). Lastly, deglycosylation of cell surface glycoproteins prior to virus inoculation did not affect the infection outcome (Fig. 4H), suggesting that host surface *N-*glycans (i.e., susceptible to PNGase F digestion) play no role in initial virus invasion, as previously suggested (33). Partial deglycosylation of host surface glycoproteins was confirmed by mobility in streptavidin blotting of cell lysates surface-biotinylated under native conditions after PNGase F treatment (Fig. S4D).

10.1128/mbio.03718-21.6 DATA SET S2 SARS-CoV-2 protein clusters and peptides identified by mass spectrometry. Download Data Set S2, PDF file, 0.6 MB. Copyright © 2022 Casas-Sanchez et al. 2022 Casas-Sanchez et al. https://creativecommons.org/licenses/by/4.0/ This content is distributed under the terms of the Creative Commons Attribution 4.0 International license.

10.1128/mbio.03718-21.7 DATA SET S3 SARS-CoV-2 spike protein sequences identified by mass spectrometry. Download Data Set S3, PDF file, 2.7 MB. Copyright © 2022 Casas-Sanchez et al. 2022 Casas-Sanchez et al. https://creativecommons.org/licenses/by/4.0/ This content is distributed under the terms of the Creative Commons Attribution 4.0 International license.

10.1128/mbio.03718-21.8 DATA SET S4 SARS-CoV-2 spike and membrane proteins and peptides identified by mass spectrometry. Download Data Set S4, PDF file, 1.0 MB. Copyright © 2022 Casas-Sanchez et al. 2022 Casas-Sanchez et al. https://creativecommons.org/licenses/by/4.0/ This content is distributed under the terms of the Creative Commons Attribution 4.0 International license.

10.1128/mbio.03718-21.9 DATA SET S5 Assignment of the fragment (MS/MS) ions of peptides with *N*-glycans removed by PNGase F treatment. Tryptic peptides were analyzed by LC-HR-MS/MS and the resulting proteomic data files processed by Proteome Discoverer and Scaffold Q+ softwares, as described in Materials and Methods in the supplemental material. (A to E) Analysis of the Scaffold Q+ MS/MS data of the Spike-derived peptides DLPQGFSALEPLVDLPIGIDITR, YNEDGTITDAVDCALDPLSETK, FPDITNLCPFGEVFDATR, TPPIKDFGGFDFSQILPDPSKPSK, and DHTSPDVDLGDISGIDASVVNIQK, which exhibited Asn (N) residue(s) modified to Asp (D) (Data Sets S2 and S3). MS/MS spectrum (1), fragmentation table (2), and assignments of the major fragment ions identified by proteomic analysis (3) are shown. (F, top sequence) Major Spike mature protein sequence (accession number A0A7L9W8W9) identified by LC-HR-MS/MS in biosample 3 (purified virions treated with PNGase F, followed by trypsin digestion). The 22 potential *N*-glycosylation sites are indicated. (Bottom sequence) Same major Spike protein (A0A7L9W8W9) with the 22 Asn (N) residues of the N-glycosylation sequons modified to Asp (D), using an in-house script, to account for an experimental deglycosylation by PNGase F treatment. The NDM annotation following the accession number was manually added to indicate the N-to-D modification(s) in the sequence. The de-*N*-glycosylated peptides identified by MS/MS are indicated (blue-lined rectangles). Download Data Set S5, PDF file, 1.3 MB. Copyright © 2022 Casas-Sanchez et al. 2022 Casas-Sanchez et al. https://creativecommons.org/licenses/by/4.0/ This content is distributed under the terms of the Creative Commons Attribution 4.0 International license.

10.1128/mbio.03718-21.10 TEXT S1 Supplemental proteomics results. Download Text S1, DOCX file, 0.02 MB. Copyright © 2022 Casas-Sanchez et al. 2022 Casas-Sanchez et al. https://creativecommons.org/licenses/by/4.0/ This content is distributed under the terms of the Creative Commons Attribution 4.0 International license.

## DISCUSSION

In this work, we investigated the essentiality of SARS-CoV-2 *N-*glycosylation in relation to infection to assess its therapeutic potential against COVID-19. Our data suggest that inhibiting host *N-*glycosylation does not prevent virus invasion in the first place, in agreement with previous studies (19, 33). However, we show that altering the *N-*glycosylation of newly synthesized viral glycoproteins has a negative impact on the maturation and production of new infective virions. Cells with altered *N-*glycosylation release fewer virions with missing or aberrant spike *N-*glycans that are essential for invasion, rendering a proportion of virions noninfectious to subsequent cells. Altogether, altering host *N-*glycosylation leads to a significant reduction of the spread of SARS-CoV-2 infection (Fig. 5).

We have demonstrated the essentiality of *N-*glycosylation for SARS-CoV-2 infection using both genetic (i.e., knocking down key glycosylation genes) and chemical means in green monkey Vero E6 cells, human HEK292 cells overexpressing the ACE-2 receptor, and ultimately in human Calu-3 lung epithelial cells as the most relevant *in vitro* model for the disease. In general, the most effective strategies at reducing viral infection were those targeting early steps in the pathway, i.e., inhibition of the formation of GlcNAc_2_-dolichol by tunicamycin and the direct inhibition of STT3 using NGI-1 (or siRNAs) led to the strongest reductions in the spread of the infection or production of infectious particles in all cell lines. Knocking down *STT3-B*, unlike *STT3-A*, was particularly effective in Vero cells, as previously reported in Lassa virus (34), and their differential substrate specificities may account for such differences between isoforms (35). Understanding the effects of inhibiting the ER α-glucosidases I and II is of particular interest because of the already available range of inhibitory compounds that are safe, well tolerated, or even FDA approved for unrelated disease (36), with repurposing potential. α-Glucosidase-II, through its catalytic subunit GANAB, deglucosylates the recently attached *N*-glycan so the glycoprotein goes through the calnexin/calreticulin folding quality control cycle. Both α-glucosidase I/II inhibitors and siGANAB successfully reduced SARS-CoV-2 infections, although not as effectively as the inhibitors of the early pathway, perhaps due to the action of the Golgi membrane’s MANEA. Importantly, since human pneumocytes do not express MANEA (37), α-glucosidase inhibitors could be more effective if clinically administered. In our experiments, not all α-glucosidase inhibitors were equally effective, and such heterogeneity was also reflected in the distinct *N-*glycoprofiles of treated cells, suggesting they affect glycosylation in different manners. The iminosugars miglustat, celgosivir, and NN-DNJ were the most effective ones, and the addition of miglitol to miglustat treatment made it particularly more efficacious at reducing viral infection and should be further investigated. Miglustat was previously proven to modify spike and ACE-2 glycosylation (38) and to reduce SARS-CoV-2 infection in Vero cells (19). It remains unknown whether efficacy of these compounds results from specific *N*-glycosylation alterations or accumulation of viral proteins in the ER due to misfolding in the calnexin/calreticulin cycle, as suggested by the increase in antispike fluorescence seen in treated cells. Moreover, alterations further down in the Golgi membrane to stop the formation of hybrid and complex *N*-glycans resulted in the least effective anti-SARS-CoV-2 strategies. Either by using DMJ to inhibit the Golgi’s α-mannosidase I, knocking down MGAT1, or inhibiting the α-mannosidase II with swainsonine, infection was reduced in HEK but did not affect Vero cells. It was previously suggested that spike complex *N*-glycans are important for infection, since MGAT1 knockout HEK293 cells were partially protected against spike-pseudotyped vesicular stomatitis virus (12), spike fusion proteins produced in MGAT1 knockout cells bound less effectively to ACE-2 (12), and most spike *N*-glycans are hybrid or complex typed (39–41). Despite glycan analyses of siMGAT1 cells showing an accumulation of Man_5_GlcNAc_2_ (the enzyme’s substrate) in both cell types, variations in the baseline *N*-glycosylation profiles between cell types may account for such different phenotypes. Since the SARS-CoV-2 isolates used in this study were originally isolated and passaged in Vero E6 cells, they also may have adapted to effectively infect Vero cells, relying less on complex *N*-glycosylation. Most of the *N*-glycosylation inhibitors tested here provided protective effects against SARS-CoV-2 infection, including a reduction in number of infected cells, new viral particles released into the supernatant, and cytopathic effects. Unlike untreated samples, which displayed most infected cells in clusters, probably formed by the spread of the virus from an infected cell to neighboring ones, inhibitor-treated samples showed discrete individual infected cells or smaller clusters instead. Protection occurred regardless of the inhibitors being added before, during, or after virus inoculation for up to 4 h, and experiments involving high MOI rates showed that the number of infected cells in inhibitor-treated samples was proportional to the initial virus inoculum, unlike untreated cells, which rapidly became fully infected. In addition, surface enzymatic deglycosylation of host cells did not alter the infection outcome, altogether suggesting that glycosylation inhibitors do not initially prevent cell invasion but affect the subsequent events of replication and infection spread.

We analyzed the supernatant of infected cells treated with a subinhibitory dose of inhibitor and quantified the number of infective virions and the amount of viral genetic material. We found that inhibitor-treated cells released fewer viral particles, suggesting that production and/or egression of virions is compromised, but we also found the presence of a significant subpopulation of virions incapable of infecting further cells. Further validation involved enzymatic deglycosylation of intact virions, which rendered them completely noninfective, evidencing the essential role of surface SARS-CoV-2 *N*-glycans for infection. Using mass spectrometry, we identified seven spike sites that were deglycosylated by PNGase F but none on the single *N-*glycosylation site in M, despite its (downshift) susceptibility to this enzyme observed by Western blotting. The *N-*glycans from RBD sites N331 and N343 were previously found to be important for pseudotype virus infection and binding to ACE-2 (5, 9, 11, 14). In addition, N234 was predicted to stabilize the RBD open conformation to promote binding to the ACE-2 receptor (5). Our results indirectly confirm the critical roles predicted for some of these glycans.

The evidence generated in this study allows further research on developing ways to exploit *N-*glycosylation to treat COVID-19. Some of the inhibitors tested here (i.e., miglustat, miglitol, acarbose, and celgosivir) are drugs in advanced clinical trials or are FDA approved, used to treat unrelated human diseases, and could be considered for repurposing if found effective in trials, alone or in combination. Further new treatments may consist of blocking essential spike *N-*glycans using antibodies or lectins to reduce the spread of the infection (42). Concerns are arising about the constant generation of new SARS-CoV-2 variants that could escape vaccine-derived antibody neutralization. Our results show evidence that infection by SARS-CoV-2 variants B.1.1.7 (Alpha), B.1.351 (Beta), P.1 (Gamma), and B.1.617.2 (Delta), some of the most common worldwide, is equally affected by the action of glycosylation inhibitors. This brings hope for a standalone therapy and may also maximize the efficacy of current vaccines, as partially glycosylated viruses may be more vulnerable to neutralizing anti-spike antibodies elicited from vaccines (14, 43). Importantly, approaches targeting essential *N-*glycosylation may offer an advantage over protein-based ones as essential *N-*glycans should be less favorable to mutation. In fact, no mutations concerning *N-*glycosylation sites have been identified to date (43, 44). Altogether, the study of *N-*glycosylation in relation to viral diseases may offer new opportunities to fight not only SARS-CoV-2 but future coronaviral outbreaks.

## MATERIALS AND METHODS

### Cell and SARS-CoV-2 culture.

Vero E6 cells (C1008; green African monkey kidney cells) were obtained from Public Health England and cultured in Dulbecco’s modified Eagle’s medium (DMEM) (Lonza) supplemented with 10% fetal bovine serum (FBS; Gibco) and 50 mg/mL gentamicin (Sigma) at 37°C and 5% CO_2_. HEK293^ACE-2^ cells (lentiviral expression of human angiotensin-converting enzyme 2) were cultured in DMEM supplemented with 10% FBS, 1 mM sodium pyruvate (Gibco), and 2 mg/mL puromycin (Invivogen) at 37°C and 5% CO_2_. Calu-3 cells were cultured in MEM supplemented with 10% FBS, 1 mM sodium pyruvate, 1× MEM nonessential amino acids (Gibco), and GlutaMAX (Gibco). Cells were regularly passaged (passages 2 to 30) by trypsinization when >80% confluence was reached. SARS-CoV-2 isolate human/Liverpool/REMRQ0001/2020 and variant B.1.617.2 were isolated from nasopharyngeal swabs from a patient in March 2020 and August 2021, respectively, and passaged four times in Vero E6 cells (45). Experimental cells were infected by incubation with SARS-CoV-2 (passage 4 in Vero E6) supernatant containing 2 × 10^7^ PFU/mL in DMEM–2% FBS for 30 min at 37°C and 5% CO_2_ at various MOIs (described below). SARS-CoV-2 variants B.1.1.7, B.1.351, and P.1 were obtained from BEI Resources and passaged once in Vero E6 before use.

### siRNA knockdowns.

A total of 2 × 10^5^ Vero E6 or HEK293^ACE-2^ cells were seeded 24 h before transfection with one of the following siRNAs pools (Qiagen SMARTpool): siSTT3-A (number GS3703), siSTT3-B (number GS201595), siGANAB (number GS23193), or siMGAT1 (number GS84061). Double knockdown of STT3-A and STT3-B was performed by transfecting equal amounts of siSTT3-A and siSTT3-B pools. Nontargeting control (siNT) was obtained from Dharmacon; 4 μL of 100 μM siRNA pool was combined with 6 μL Lipofectamine RNAiMax (Invitrogen) in 200 μL Opti-MEM (Gibco) and incubated for 20 min at room temperature (RT) before being added dropwise to the cells. Cells were flushed out with fresh medium 4 h after transfection. For siGANAB and siMGAT1, 2 hits of siRNA over a 120-h incubation period were performed, while for siSTT3-A, siSTT3-B, and siSTT3-A+B, 3 hits of siRNA over a 168-h period were needed. The day before optimum knockdown (96 h for 2 hits, 144 h for 3 hits), cells were trypsinized and 2 × 10^4^ cells were seeded for each condition onto 96-well optical plates (Greiner) in quadruplicate and infected the following day. Infections were carried out by incubation with SARS-CoV-2 P4 supernatant at an MOI of 0.05 (Vero E6) or MOI of 0.1 (HEK293^ACE-2^) for 30 min at 37°C and 5% CO_2_, with washing and incubation in DMEM, 2% FBS at 37°C and 5% CO_2_ for 24 h.

### *N-*Glycosylation inhibitors.

Cells were treated with either DMSO or several *N*-glycosylation inhibitors (from DMSO stock solutions; DMSO final concentration never exceeding 2%) in DMEM–2% FBS. Inhibitors used were tunicamycin (Sigma), NGI-1 (Sigma), DNJ (Cambridge Bioscience), miglitol (Stratech Scientific), miglustat (Cambridge Bioscience), acarbose (Fisher Scientific), NN-DNJ (Cambridge Bioscience), celgosivir (Sigma), DMJ (Tebu-Bio), and swainsonine (Cambridge Bioscience). Cells were then infected by incubation with SARS-CoV-2 P4 supernatant with the corresponding inhibitor for 30 min at 37°C and 5% CO_2_ at MOI of 0.001 (Vero E6 for plaque assay), MOI of 0.05, or MOI of 0.1 (Vero E6 and HEK293^ACE-2^ for immunofluorescence assay, respectively). Cells were washed and left in DMEM–2% FBS and *N-*glycosylation inhibitor at 37°C and 5% CO_2_ for 48 h (Vero E6 plaque assay) or 24 h (immunofluorescence). For toxicity assays, cells in 96-well plates were treated with variable concentrations of inhibitors in DMEM–2% FBS in triplicate, and after 48 h, an alamarBlue 10× solution (Invitrogen) was added and incubated for 4 h at 37°C before measuring well fluorescence intensity at 590 nm using a FLUOstar Omega plate reader (BMG Labtech).

### Plaque assays.

Vero E6 were cultured in DMEM–10% FBS in 24-well plates and allowed to reach confluence overnight. Cells were incubated with 0.1 mL serially diluted supernatant containing SARS-CoV-2 for 30 min at 37°C and 5% CO_2_ before being left in 1.5 mL overlay (DMEM, 2% FBS, 1.2% cellulose microcrystalline [Sigma]) at 37°C and 5% CO_2_ for 72 h. Cells were fixed in formaldehyde for 30 min, washed, and stained with 0.1% crystal violet, 20% ethanol solution for manual plaque counting.

### Reverse transcription-qPCR viral RNA quantification.

Total RNA was extracted from supernatants of SARS-CoV-2-infected Vero E6 cells using the RNeasy kit (Qiagen) by following the manufacturer’s protocol; 4 mL purified RNA was subsequently used for reverse transcription-quantitative PCR (qPCR) using the Luna Universal one-step reverse transcription-qPCR kit (New England Biolabs) and 0.4 μM each primer: CoV2-N-F (CACATTGGCACCCGCAATC) and CoV2-N-R (GAGGAACGAGAAGAGGCTTGAC). Reactions were set and analyzed in an Agilent Mx3005P machine as 55°C for 10 min, 95°C for 1 min, and 40 cycles of 95°C for 10 s, followed by 60°C for 30 s, with a final melting curve.

### Immunofluorescence assay.

Cells cultured in optical plates were fixed in 4% formaldehyde (Pierce) for 30 min at RT, washed in phosphate-buffered saline (PBS), and permeabilized in 0.5% saponin for 10 min. Cells were then blocked in 1% bovine serum albumin (BSA)–PBS for 45 min and further incubated in blocking solution containing anti-SARS-CoV-2 spike protein subunit 2 (Abcam) at 1:1,000 dilution for 1 h at 37°C. Blocking solution containing 1 mg/mL 4′,6-diamidino-2-phenylindole (DAPI) and Alexa Fluor 488 Plus-conjugated anti-mouse IgG (ThermoFisher) was then incubated for 1 h at RT. Cells were washed and left in PBS for imaging. Imaging was carried out using a Zeiss LSM800 confocal laser scanning microscope by tile-scanning entire plates, conserving identical settings between comparable samples. Images were analyzed using Fiji (ImageJ) by thresholding to select for true signal and measuring relative cell area coverage in the DAPI channel (total number of cells) and anti-spike area or mean fluorescence intensity (infected cells). Infection rates given as normalized infected area (anti-spike positive) over total cell area (DAPI).

### PNGase F deglycosylation of intact SARS-CoV-2 virions.

Two milliliters of SARS-CoV-2 P4 supernatant containing 2 × 10^7^ PFU/mL was purified using an Amicon Ultra column (molecular size cutoff, 100 kDa; Merck) by centrifugation and washing in PBS at 2,000 × *g*; 40 mL purified supernatant was combined with 2,500 U recombinant glycerol-free PNGase F and Glycobuffer 2 (New England Biolabs) by following the manufacturer’s protocol for nondenaturing digestion and incubated at 37°C for 5 h. Parallel controls included incubation of purified virions with or without PNGase F and Glycobuffer, incubation with heat-inactivated (75°C for 10 min) PNGase F, and incubation with PNGase F and Glycobuffer during virus inoculation of host cells for 30 min. After incubation, samples were either directly used for infection or combined with radioimmunoprecipitation assay (RIPA) buffer supplemented with protease inhibitors to generate lysates for blotting. Alternatively, Vero E6 cells were preincubated with or without 100,000 U/mL PNGase F in serum-free Opti-MEM for 5 h at 37°C and 5% CO_2_ before being either inoculated with SARS-CoV-2 or surface biotinylated. To biotinylate surface proteins, cells were washed in PBS, pH 8, incubated with 2.5 mM EZ-link NHS-biotin (ThermoFisher) for 30 min on ice, washed in PBS–100 mM glycine, and lysed in RIPA buffer and protease inhibitors as described above.

### Western, lectin, and streptavidin blotting.

siRNA cell lysates were obtained at either 120 h (2 hits) or 168 h (3 hits) by washing cells in cold PBS and incubation in RIPA buffer with 1% phosphatase and protease inhibitor cocktail (ThermoScientific). Protein concentration was determined using Precision Red (Cytoskeleton), and 60 μg of protein was separated using 4 to 15% SDS-PAGE gels and transferred onto nitrocellulose membrane. Alternatively, virion lysates were run in 4 to 12% SDS-PAGE and dry transferred onto polyvinylidene difluoride membranes. Membranes were blocked in either 5% milk or 2% BSA in Tris-buffered saline or PBS, 0.1% Tween 20 (TBS-T) for 30 min and incubated with their corresponding primary antibodies at 4°C overnight. Primary antibodies used were anti-STT3-A (HPA030735; Cambridge Bioscience), anti-STT3-B (A15574; Universal Biologicals), anti-GANAB (A13851; Universal Biologicals), anti-MGAT1 (CSB-PA013773ESR1Hu; 2B Scientific), anti-glyceraldehyde-3-phosphate dehydrogenase (GAPDH) (10494-1-AP; ProteinTech), anti-spike S2 (Abcam), and anti-M (Rockland). Blots were then incubated with anti-rabbit IgG conjugated with horseradish peroxidase (Cell Signaling) or Alexa Fluor Plus 800 (ThermoFisher) or anti-mouse IgG conjugated with Alexa Fluor Plus 680 (ThermoFisher) for 1 h. Blots were visualized on a Bio-Rad ChemiDoc touch gel imaging system using enhanced chemiluminescence substrate (for horseradish peroxidase secondary antibody) or on a LI-COR Odyssey Fc (for fluorescence). Virion lysate blots were then incubated with 5 mg/mL concanavalin A fluorescein isothiocyanate (FITC) conjugated in 1% BSA, 0.05% Tween 20, PBS for 30 min. GAPDH or nigrosine staining was utilized as a loading control. Streptavidin blots were incubated in streptavidin-Alexa Fluor 555 (ThermoFisher) at 5 mg/mL in PBS, 0.1% Tween 20, 0.02% SDS for 30 min at RT. Lectin and streptavidin blots were imaged using a ThermoFisher iBright FL1500 system.

### *N-*Glycoprofiling.

Either siRNA- or inhibitor-treated cells were harvested by trypsinization, washed in cold PBS, manually counted, and snap-frozen. A total of 2 × 10^6^ (Vero E6) or 3 × 10^6^ (HEK293^ACE-2^) cells were used for the analyses alongside Ludger IgG as positive and water as negative controls for in-solution *N-*glycan release, labeling, and cleanup. Samples were denatured at 100°C with SDS and dithiothreitol before incubation overnight with PNGase F in NP-40 buffered solution. The released glycans were then converted to aldoses with 0.1% formic acid, filtered through a protein binding membrane, and dried. *N-*glycans were then labeled with procainamide, cleaned up in water, dried, and reconstituted in water. Samples were analyzed by HILIC-UPLC using an Acquity UPLC BEH-glycan (1.7 mm, 2.1 by 150 mm) column at 40°C on a Thermo Scientific UltiMate 3000 UPLC with a fluorescence detector (excitation, 310 nm; emission, 370 nm) controlled by HyStar software v3.2. Gradient conditions were 0 to 53.5 min, 24% to 49% buffer (A) at a flow rate of 0.4 mL/min; 53.5 to 55.5 min, 49% to 100% A at 0.4 to 0.2 mL/min; 55.5 to 57.5 min, 100% A at 0.2 mL/min; 57.5 to 59.5 min, 100% to 24% A at 0.2 mL/min; 59.5 to 65.5 min, 24% A at 0.2 mL/min; 65.5 to 66.5 min, 24% A at 0.2 to 0.4 mL/min; 66.5 to 70 min, 24% A at 0.4 mL/min. Buffer A was 50 mM ammonium formate. Buffer B was acetonitrile (acetonitrile 190 far-UV/gradient quality). Samples were injected in 25% aqueous, 75% acetonitrile, with an injection volume of 25 μL. Ludger procainamide-labeled glucose homopolymer, A2 and A3 mix, FA2 mix, and Man mix were used as standards and as external calibrations for GU allocation. All standards were run in the same injection volume and conditions as the samples. Thermo Scientific Chromeleon software v7.2.1 was used to allocate GU values to peaks. Glycan structures were assumed from chromatographic mobility by comparison with standards and may not be definitive, as they were not confirmed by mass spectrometry. Abundances were quantified by integration of area under each peak. When software did not split peaks that were not fully resolved, areas were merged together across peaks. Glycan structures in figures are depicted using CFG nomenclature; the Oxford nomenclature is used in Data Set S1 due to its broader applicability. Glycan analyses were performed by Ludger Ltd. (46–48).

### Trypsin digestion for proteomic analysis.

Purified virions were subjected to PNGase F treatment (*P*+; sample 3) or heat-inactivated PNGase F treatment (P_IN_; sample 4) as described above. The positive controls (for viral proteins) included purified virions (C; sample 1) and PNGase F mock treatment of purified virions (P-; sample 2). The negative controls (devoid of viral proteins) included DMEM, 2% FBS (sample 5), and Vero E6 cell culture conditioned supernatant or secretome in DMEM, 2% FBS (sample 6), both purified by size exclusion as described above. Samples were incubated in 80% ice-cold acetone for 1 h at −20°C for protein precipitation and spun down at 14,000 × *g* for 15 min, and pellets were air dried. Peptides were obtained from the via FASP protein digestion kit (ab270519; Abcam, Cambridge, MA) using trypsin (proteomics grade; T6567; Sigma-Aldrich, St. Louis, MO). Lyophilized intact protein samples 1 to 6 (141 to 478 μg protein) were redissolved in 200 μl 100 mM Tris-HCl, pH 7.0, containing 8 M urea (urea sample solution, or USS). Protein digestion was performed with 100 μl of each sample and transferred to a 1.5-mL microtube containing 200 μL USS; 100 mM dl-dithiothreitol (D0632; Sigma-Aldrich) was added to reduce protein disulfide bonds and achieve complete protein unfolding. Samples were vortexed for 30 s and then kept swaying on a nutating mixer (Nutator mixer model 117; TCS Scientific Corp.) for 45 min at RT prior to the proteome extract digestion protocol. Samples were transferred to a 30-kDa spin filter (FASP kit), previously assembled on a collection tube, and centrifuged at 14,000 × *g* for 15 min at RT; 200 μl USS was added to each spin filter and further centrifuged for 15 min. Flowthrough from the collection tube was discarded, and 100 μl of fresh prepared 10 mM iodoacetamide, resuspended in USS, was added to the spin filter and samples subsequently subjected to incubation without mixing for 20 min in the dark at RT for alkylation. After incubation, the spin filters were centrifuged at 14,000 × *g* for 10 min at RT; 100 μl of USS was added to the spin filter, and samples were centrifuged at 14,000 × *g* for 15 min. This step was repeated twice. Flowthrough was again discarded; 100 μL 50 mM ammonium bicarbonate solution was added to the spin filter, and the sample was centrifuged at 14,000 × *g* for 15 min at RT. This step was repeated twice. After USS removal by ammonium bicarbonate, spin filter containing protein sample was transferred to a new collection tube, and 100 μL trypsin was added to the spin filter at an enzyme-to-protein ratio of 1:50. Evaporation was prevented by wrapping the top of the tubes with Parafilm. The spin filter was incubated at 37°C for 16 h without mixing; 200 μL LC-MS grade 0.1% formic acid was added to stop the proteolysis, and each sample was then centrifuged at 14,000 × *g* for 10 min to obtain the filtrate containing the tryptic peptides. The collection tubes containing the peptides were dried down in a Speedvac concentrator (Savant; Thermo Fisher Scientific), and the volume was adjusted with LC-MS-grade 0.1% formic acid (Thermo Fisher Scientific) to a final concentration of 1 μg/μL tryptic peptides prior to liquid chromatography-high resolution-tandem mass spectrometry (LC-HR-MS/MS) analysis.

### Proteomic analysis by LC-HR-MS/MS.

The equivalent of 1 μg of protein digest from each sample (samples 1 to 6), as measured by a NanoDrop One/One Microvolume UV-visible spectrophotometer (Thermo Fisher Scientific), was loaded onto a 50-cm μPAC capLC column (PharmaFluidics, Ghent, Belgium) separated by a Dionex ultimate 3000 RSLCnano (Thermo Fisher Scientific) with 99% solvent A (100% water, 0.1% formic acid) and 1% solvent B (90% acetonitrile, 0.1% formic acid) at a flow rate of 750 nL/min for 5 min. The flow rate was decreased to 300 nL/min at 5.1 min to start the elution gradient. Solvent B was increased to 22% over 64.9 min and then to 45% solvent B over 15 min. The gradient was increased to 95% solvent B over 5 min and maintained at a plateau for an additional 9 min. The gradient sharply decreased to 1% solvent B over 1 min for equilibration with a flow rate at 750 nL/min and maintained for 10 min, thus ending the 120-min sample runtime. Eluted peptides were ionized with a μPAC Flex iON connect (PharmaFluidics, Ghent, Belgium) attached to a Nanospray Flex Ion Source (Thermo Fisher Scientific) equipped with a nanoESI emitter (FOSSILIONTECH, Madrid, Spain). Data were acquired using a Q Exactive Plus hybrid quadrupole Orbitrap mass spectrometer (QE Plus, Thermo Fisher Scientific). The QE Plus analysis parameters were full MS resolution at 70,000; automatic gain control (AGC) target of 1e^6^; and scan from 350 to 1,400 *m/z* range. Top 10 data-dependent MS^2^ parameters were set to 17,500 resolution, ACG target of 1e^5^, (N)CE of 27, and charged exclusion at unassigned, +1, +6 to 8, and >8 with mass exclusion set to ON. Samples 5 and 6 were run previously to generate an exclusion list. The top 200 ions were collected every 5 min (in a total run of 120 min) to generate a 4,800-ion exclusion list prior to sample injection containing virions (samples 1 to 4). All samples were run in technical duplicate.

### Bioinformatic analysis of proteomic data.

Proteomic data analysis was initially performed using Proteome Discoverer (PD) v2.5.0.400 (Thermo Fisher Scientific), with an estimated false discovery rate (FDR) of 1%. Common contaminants such as trypsin autolysis fragments, human keratins, and protein lab standards were included as well as in-house contaminants, which may be found in the cRAP contaminant database (49). The SARS-CoV-2 database (36,795 entries) was downloaded in FASTA format on 18 May 2021 from UniProtKB (http://www.uniprot.org/). Additionally, a second SARS-CoV-2 database was added to include the modification of asparagine (N) to aspartic acid (D), resulting from PNGase F treatment (31), to illustrate the conserved *N*-glycosylation sequon N-X-S/T, where X is any amino acid except proline. The following parameters were used in the PD analysis: HCD MS/MS; fully tryptic peptides only; up to 2 missed cleavages; parent-ion mass tolerance of 10 ppm (monoisotopic); and fragment mass tolerances of 0.6 Da (in Sequest) and 0.02 Da (in PD 2.1.1.21) (monoisotopic). A filter of two high-confidence peptides per protein was applied for identifications. The PD data set was further processed through Scaffold Q+ 4.8.2 (Proteome Software, Portland, OR) to obtain the protein quantification and further analysis of the MS/MS spectra. A protein threshold of 95%, peptide threshold of 90%, and a minimum of 1 peptide were used for protein validation by manual inspection of each MS/MS spectrum.

### Statistical analyses.

Biological and technical replicates are defined in the corresponding figure legends. Data were analyzed using one-tailed *t* test assuming normal distribution.

### Data availability.

All data are available within the main manuscript and supplemental material. Raw proteomics data are deposited into PRIDE (EMBL-EBI) repository under accession number PXD026431.
