# Supplementary figures and images for: Inhibition of Protein N-Glycosylation Blocks SARS-CoV-2 Infection
Source: mBio. 2022 Feb 15;13(1):e03718-21. doi: 10.1128/mbio.03718-21 (PMC8844921; doi:10.1128/mbio.03718-21)

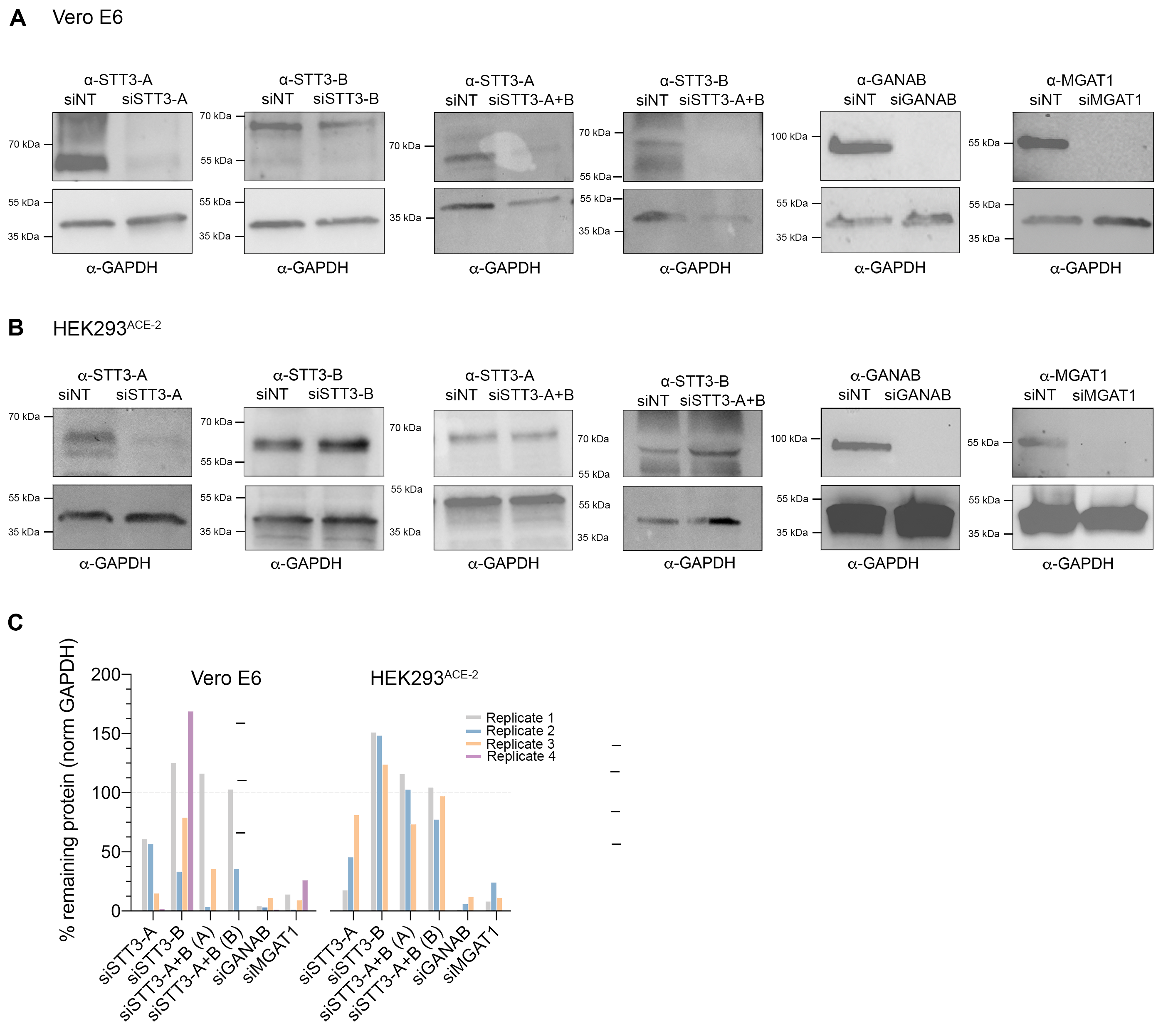

Supplement: FIG S1 [file mbio.03718-21-sf001.tif]

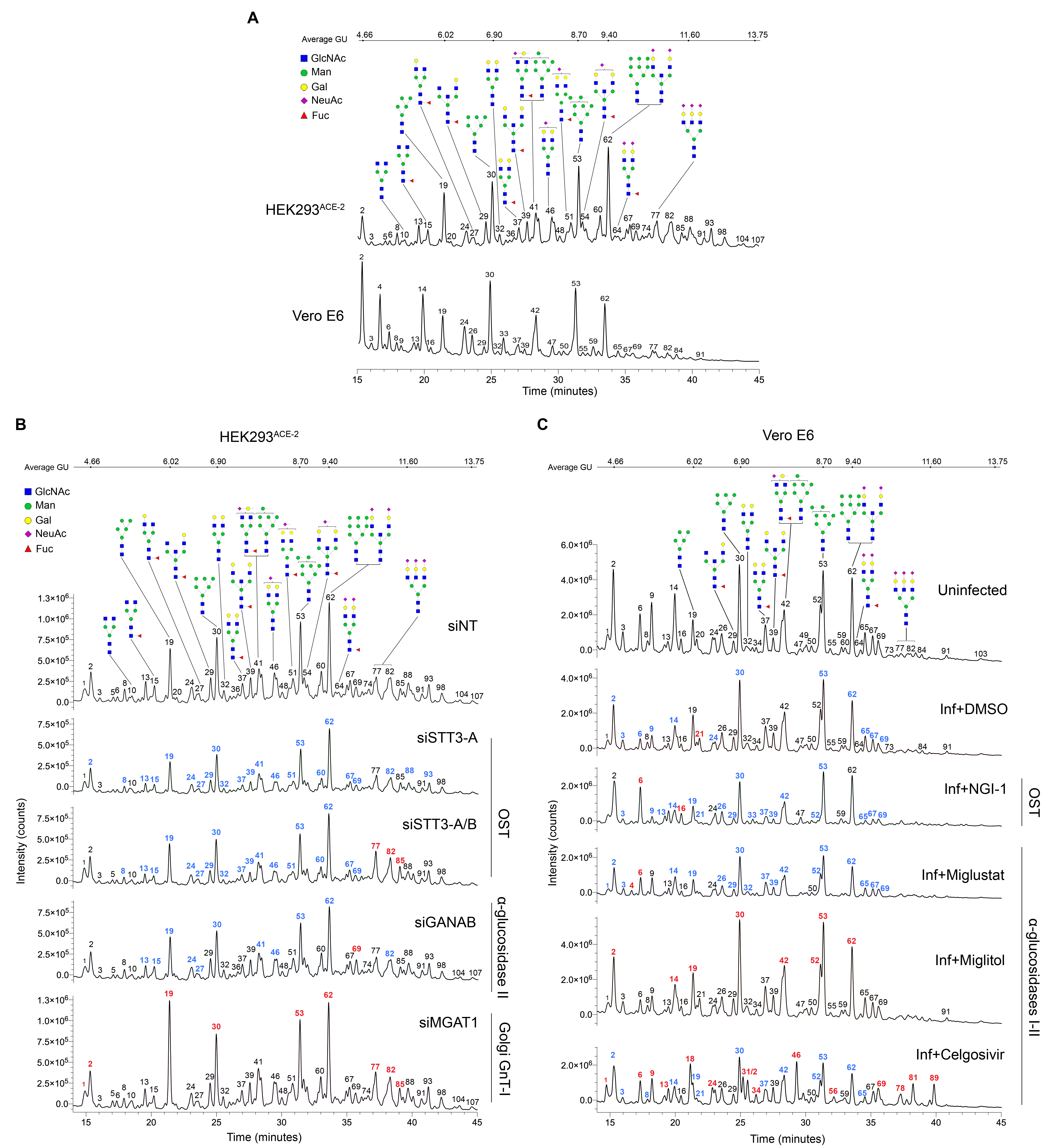

Supplement: FIG S3 [file mbio.03718-21-sf003.tif]

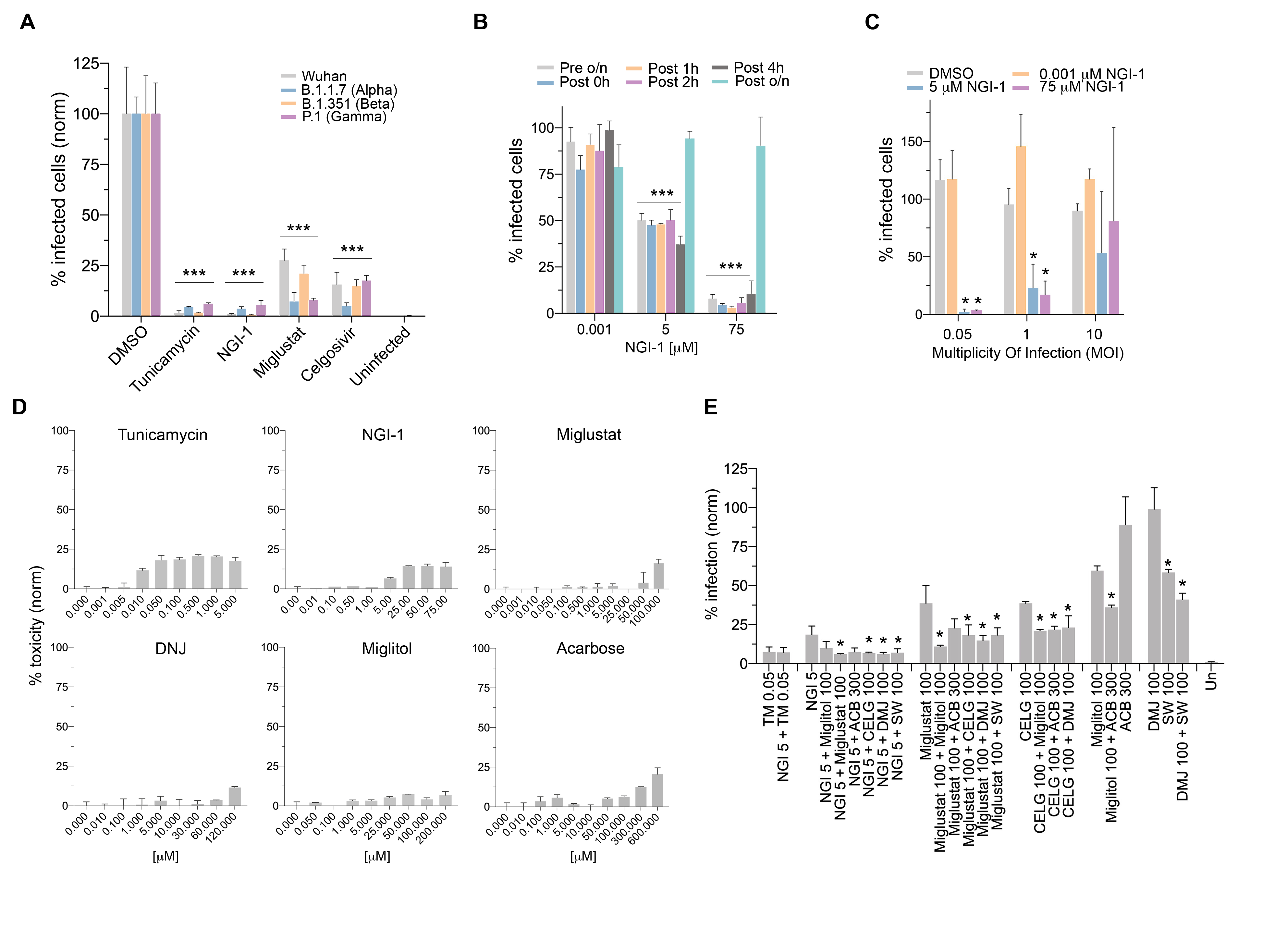

Supplement: FIG S2 [file mbio.03718-21-sf002.tif]

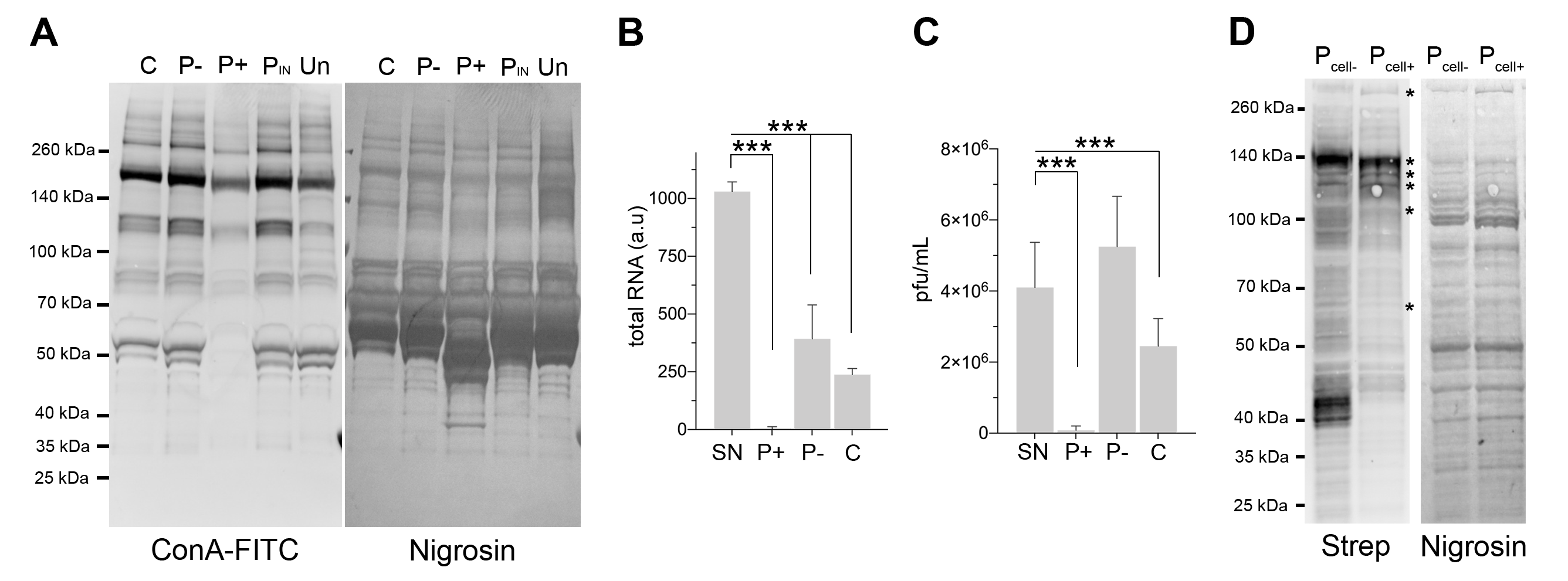

Supplement: FIG S4 [file mbio.03718-21-sf004.tif]
