# Supplementary material for: Inhibition of Protein N-Glycosylation Blocks SARS-CoV-2 Infection
Source: mBio. 2022 Feb 15;13(1):e03718-21. doi: 10.1128/mbio.03718-21 (PMC8844921; doi:10.1128/mbio.03718-21)
Supplement: DATA SET S2 [file mbio.03718-21-sd002.pdf]

Identified SARS-CoV-2 Protein Clusters

#	Protein Cluster/Protein	Accession Number	Alternate ID	Molecular Mass (kDa)	1_Control_Purified_Virions_1 [a]	1_Control_Purified_Virions_2 [a]	2_Mock_Treated_Purified_Virions_1 [a]	2_Mock_Treated_Purified_Virions_2 [a]	3_PNGase-F_Treated_Purified_Virions_1 [a]	3_PNGase-F_Treated_Purified_Virions_2 [a]	4_Inactivated _PNGase-F_Treated_Purified_Virions_1 [a]	4_Inactivated_PNGase-F_Treated_Purified_Virions_2 [a]
**2**	**Cluster of Surface/Spike (S) Glycoprotein OS=Severe acute respiratory syndrome coronavirus 2 OX=2697049 GN=S PE=4 SV=1**		**S**		**12**	**12**	**14**	**9**	**36**	**35**	**35**	**30**
2a	Surface glycoprotein OS=Severe acute respiratory syndrome coronavirus 2 OX=2697049 GN=S PE=4 SV=1	A0A7L9W8W9_NDM [b]	S	140	9	10	11	5	33	34	33	30
2b	Surface glycoprotein OS=Severe acute respiratory syndrome coronavirus 2 OX=2697049 GN=S PE=4 SV=1	A0A7L8Z0C9	S	141	9	11	8	5	31	30	31	30
2c	Surface glycoprotein OS=Severe acute respiratory syndrome coronavirus 2 OX=2697049 GN=S PE=4 SV=1	A0A7L8AB65	S	141	5	5	5	2	19	20	19	16
2d	Spike glycoprotein OS=Severe acute respiratory syndrome coronavirus 2 OX=2697049 GN=S PE=3 SV=1	A0A7G3UUL1	S	141	3	4	2	1	19	20	19	17
2e	Surface glycoprotein OS=Severe acute respiratory syndrome coronavirus 2 OX=2697049 GN=S PE=4 SV=1	A0A7L9F8J2	S	141	5	4	5	3	18	17	16	14
2f	Spike glycoprotein OS=Severe acute respiratory syndrome coronavirus 2 OX=2697049 GN=S PE=3 SV=1	A0A7D7LL25	S	141	6	6	6	3	15	17	13	11
2g	Spike glycoprotein OS=Severe acute respiratory syndrome coronavirus 2 OX=2697049 GN=S PE=3 SV=1	A0A7D5XMN0	S	141	3	4	2	2	5	6	4	4
2h	Spike glycoprotein OS=Severe acute respiratory syndrome coronavirus 2 OX=2697049 GN=S PE=3 SV=1	A0A6N1WN54	S	141	0	0	0	2	0	0	0	0
2i	Spike glycoprotein OS=Severe acute respiratory syndrome coronavirus 2 OX=2697049 GN=S PE=3 SV=1	A0A7D7XU89	S	141	1	0	1	0	0	0	0	0
**3**	**Cluster of Membrane (M) Protein OS=Severe acute respiratory syndrome coronavirus 2 OX=2697049 GN=M PE=3 SV=1**		**M**		**3**	**0**	**1**	**1**	**8**	**8**	**8**	**11**
3a	Membrane glycoprotein OS=Severe acute respiratory syndrome coronavirus 2 OX=2697049 GN=M PE=4 SV=1	A0A7M1HLZ4	M	25	1	0	1	1	7	8	7	10
3b	Membrane protein OS=Severe acute respiratory syndrome coronavirus 2 OX=2697049 GN=M PE=3 SV=1	A0A6M4NE17	M	25	1	0	1	1	7	8	7	10
**1**	**Cluster of Nucleoprotein SP Negative Modified OS=Severe acute respiratory syndrome coronavirus 2 OX=2697049 GN=N PE=1 SV=1**		**N**		**23**	**28**	**26**	**13**	**49**	**46**	**54**	**56**
1a	Nucleocapsid phosphoprotein OS=Severe acute respiratory syndrome coronavirus 2 OX=2697049 GN=N PE=4 SV=1	A0A7L8MNE6	N	46	18	21	15	8	43	40	43	50
1b	Nucleocapsid phosphoprotein OS=Severe acute respiratory syndrome coronavirus 2 OX=2697049 GN=N PE=4 SV=1	A0A7M2G704	N	46	10	12	9	5	16	14	13	21
1c	Nucleoprotein OS=Severe acute respiratory syndrome coronavirus 2 OX=2697049 GN=N PE=3 SV=1	A0A7H1CZG8	N	46	0	4	4	1	5	3	2	6
1d	Nucleoprotein OS=Severe acute respiratory syndrome coronavirus 2 OX=2697049 GN=N PE=3 SV=1	A0A7G8ATM2	N	46	6	11	9	5	4	2	1	3
1e	Nucleoprotein OS=Severe acute respiratory syndrome coronavirus 2 OX=2697049 GN=N PE=3 SV=1	A0A7D7NMS6	N	46	1	6	4	2	4	2	0	1

[a] All values are quantitative values, expressed as normalized total spectra.

[b] NDM, Asn to Asp modification. The NDM annotation was manually added following the accession number of the original Spike sequence to indicate that the asparagine (N) residues were in-silico-modified to aspartic acid (D) by an in-house script, to account for an experimental *N* -deglycosylation by the PNGase-F treatment.

All Individual Proteins and Peptides

Biological sample category	Protein name	Protein accession number [a]	Alternate ID	Protein molecular mass (Da)	Protein identification probability (Prob)	Exclusive unique peptide count (#Pep)	Exclusive unique spectrum count (#Unique)	Total spectrum count (#Spec)	Percentage of total spectra (%Spec)	Percentage of amino acids identified (%Cov)	Peptide sequence	Previous amino acid	Next amino acid	Best Peptide Identification Probability	Best Sequest deltaCn	Best Sequest XCorr	Best Sequest SpRank	Number of identified +1H spectra	Number of identified +2H spectra	Number of identified +3H spectra	Number of identified +4H spectra	Calculated +1H Peptide Mass (AMU)	Median Retention Time	Total TIC	Peptide start index	Peptide stop index	Other Proteins
1_Control_Purified_Virions	Spike glycoprotein OS=Severe acute respiratory syndrome coronavirus 2 OX=2697049 GN=S PE=3 SV=1	A0A7D7LL25	S	141,350.10	69.30%	0	0	5	0.02%	3.69%	FQTLLALHR	R	S	99.00%	0.7610	2.3200	1	0	0	1	0	1,098.64	4,952.73	383,852	238	246	A0A7L8AB65,A0A7L8Z0C9,A0A7G3UUL1,A0A7L9W8W9_NDM,A0A7L9F8J2
1_Control_Purified_Virions	Spike glycoprotein OS=Severe acute respiratory syndrome coronavirus 2 OX=2697049 GN=S PE=3 SV=1	A0A7D7LL25	S	141,350.10	69.30%	0	0	5	0.02%	3.69%	GVYYPDKVFR	R	S	99.00%	0.7470	2.0200	1	0	0	1	0	1,243.65	4,530.86	1,168,410	35	44	A0A7L8AB65,A0A7L8Z0C9,A0A7G3UUL1,A0A7L9W8W9_NDM
1_Control_Purified_Virions	Spike glycoprotein OS=Severe acute respiratory syndrome coronavirus 2 OX=2697049 GN=S PE=3 SV=1	A0A7D7LL25	S	141,350.10	69.30%	0	0	5	0.02%	3.69%	KSNLKPFER	R	D	99.00%	0.5640	2.0200	1	0	0	2	0	1,118.63	2,890.75	2,587,020	458	466	A0A7L8AB65,A0A7L8Z0C9,A0A7L9W8W9_NDM,A0A7L9F8J2
1_Control_Purified_Virions	Spike glycoprotein OS=Severe acute respiratory syndrome coronavirus 2 OX=2697049 GN=S PE=3 SV=1	A0A7D7LL25	S	141,350.10	69.30%	0	0	5	0.02%	3.69%	VVVLSFELLHAPATVCGPK	R	K	99.00%	0.7200	4.0400	1	0	0	1	0	2,037.12	6,357.62	2,207,880	510	528	A0A7L8Z0C9,A0A7L9W8W9_NDM,A0A7L9F8J2
1_Control_Purified_Virions	Spike glycoprotein OS=Severe acute respiratory syndrome coronavirus 2 OX=2697049 GN=S PE=3 SV=1	A0A7D7LL25	S	141,350.10	97.30%	0	0	5	0.02%	3.69%	FQTLLALHR	R	S	97.20%	0.8380	2.8900	1	0	0	1	0	1,098.64	4,933.91	621,494	238	246	A0A7L8AB65,A0A7L8Z0C9,A0A7G3UUL1,A0A7L9W8W9_NDM,A0A7L9F8J2
1_Control_Purified_Virions	Spike glycoprotein OS=Severe acute respiratory syndrome coronavirus 2 OX=2697049 GN=S PE=3 SV=1	A0A7D7LL25	S	141,350.10	97.30%	0	0	5	0.02%	3.69%	GVYYPDKVFR	R	S	97.20%	0.7700	2.0200	1	0	0	2	0	1,243.65	4,494.26	2,285,430	35	44	A0A7L8AB65,A0A7L8Z0C9,A0A7G3UUL1,A0A7L9W8W9_NDM
1_Control_Purified_Virions	Spike glycoprotein OS=Severe acute respiratory syndrome coronavirus 2 OX=2697049 GN=S PE=3 SV=1	A0A7D7LL25	S	141,350.10	97.30%	0	0	5	0.02%	3.69%	KSNLKPFER	R	D	97.20%	0.6230	2.6800	1	0	0	1	0	1,118.63	2,913.98	1,222,680	458	466	A0A7L8AB65,A0A7L8Z0C9,A0A7L9W8W9_NDM,A0A7L9F8J2
1_Control_Purified_Virions	Spike glycoprotein OS=Severe acute respiratory syndrome coronavirus 2 OX=2697049 GN=S PE=3 SV=1	A0A7D7LL25	S	141,350.10	97.30%	0	0	5	0.02%	3.69%	VVVLSFELLHAPATVCGPK	R	K	97.20%	0.7990	4.2600	1	0	0	1	0	2,037.12	6,352.68	1,301,320	510	528	A0A7L8Z0C9,A0A7L9W8W9_NDM,A0A7L9F8J2
1_Control_Purified_Virions	Spike glycoprotein OS=Severe acute respiratory syndrome coronavirus 2 OX=2697049 GN=S PE=3 SV=1	A0A7G3UUL1	S	141,447.00	88.70%	0	0	3	0.01%	1.49%	FQTLLALHR	R	S	97.20%	0.8380	2.8900	1	0	0	1	0	1,098.64	4,933.91	621,494	238	246	A0A7L8AB65,A0A7L8Z0C9,A0A7D7LL25,A0A7L9W8W9_NDM,A0A7L9F8J2
1_Control_Purified_Virions	Spike glycoprotein OS=Severe acute respiratory syndrome coronavirus 2 OX=2697049 GN=S PE=3 SV=1	A0A7G3UUL1	S	141,447.00	88.70%	0	0	3	0.01%	1.49%	GVYYPDKVFR	R	S	97.20%	0.7700	2.0200	1	0	0	2	0	1,243.65	4,494.26	2,285,430	35	44	A0A7L8AB65,A0A7L8Z0C9,A0A7D7LL25,A0A7L9W8W9_NDM
1_Control_Purified_Virions	Spike glycoprotein OS=Severe acute respiratory syndrome coronavirus 2 OX=2697049 GN=S PE=3 SV=1	A0A7D5XMN0	S	141,214.70	74.60%	0	0	3	0.01%	4.95%	ALTGIAVEQDKNTQEVFAQVK	R	Q	97.20%	0.7940	5.8000	1	0	0	1	0	2,289.21	5,424.23	2,331,850	766	786	A0A7L8Z0C9,A0A7L9W8W9_NDM
1_Control_Purified_Virions	Spike glycoprotein OS=Severe acute respiratory syndrome coronavirus 2 OX=2697049 GN=S PE=3 SV=1	A0A7D5XMN0	S	141,214.70	74.60%	0	0	3	0.01%	4.95%	LNDLCFTNVYADSFVIRGDEVR	K	Q	97.20%	0.8400	3.4200	1	0	0	1	0	2,603.26	6,390.85	718,966	387	408	A0A7L8Z0C9,A0A7L9W8W9_NDM
1_Control_Purified_Virions	Spike glycoprotein OS=Severe acute respiratory syndrome coronavirus 2 OX=2697049 GN=S PE=3 SV=1	A0A7D5XMN0	S	141,214.70	74.60%	0	0	3	0.01%	4.95%	SFIEDLLFNKVTLADAGFIK	R	Q	97.20%	0.8960	4.2700	1	0	0	1	0	2,241.22	6,720.77	630,101	816	835	A0A7L8Z0C9,A0A7L9W8W9_NDM
1_Control_Purified_Virions	Spike glycoprotein OS=Severe acute respiratory syndrome coronavirus 2 OX=2697049 GN=S PE=3 SV=1	A0A7G3UUL1	S	141,447.00	97.70%	0	0	2	0.01%	1.49%	FQTLLALHR	R	S	99.00%	0.7610	2.3200	1	0	0	1	0	1,098.64	4,952.73	383,852	238	246	A0A7L8AB65,A0A7L8Z0C9,A0A7D7LL25,A0A7L9W8W9_NDM,A0A7L9F8J2
1_Control_Purified_Virions	Spike glycoprotein OS=Severe acute respiratory syndrome coronavirus 2 OX=2697049 GN=S PE=3 SV=1	A0A7G3UUL1	S	141,447.00	97.70%	0	0	2	0.01%	1.49%	GVYYPDKVFR	R	S	99.00%	0.7470	2.0200	1	0	0	1	0	1,243.65	4,530.86	1,168,410	35	44	A0A7L8AB65,A0A7L8Z0C9,A0A7D7LL25,A0A7L9W8W9_NDM
1_Control_Purified_Virions	Spike glycoprotein OS=Severe acute respiratory syndrome coronavirus 2 OX=2697049 GN=S PE=3 SV=1	A0A7D5XMN0	S	141,214.70	61.80%	0	0	2	0.01%	3.38%	ALTGIAVEQDKNTQEVFAQVK	R	Q	99.00%	0.7990	5.5600	1	0	0	1	0	2,289.21	5,435.09	1,868,740	766	786	A0A7L8Z0C9,A0A7L9W8W9_NDM
1_Control_Purified_Virions	Spike glycoprotein OS=Severe acute respiratory syndrome coronavirus 2 OX=2697049 GN=S PE=3 SV=1	A0A7D5XMN0	S	141,214.70	61.80%	0	0	2	0.01%	3.38%	LNDLCFTNVYADSFVIRGDEVR	K	Q	99.00%	0.8380	3.7800	1	0	0	1	0	2,603.26	6,394.46	832,082	387	408	A0A7L8Z0C9,A0A7L9W8W9_NDM
1_Control_Purified_Virions	Spike glycoprotein OS=Severe acute respiratory syndrome coronavirus 2 OX=2697049 GN=S PE=3 SV=1	A0A7D7XU89	S	141,169.60	96.50%	1	1	1	0.00%	2.28%	VCEFQFCNDPFLGVYYHENNKSWMESEFR	K	V	96.90%	0.9560	0.4090	1	0	0	0	0	3,732.60	5,892.84	367,183	130	158	
1_Control_Purified_Virions	Surface glycoprotein OS=Severe acute respiratory syndrome coronavirus 2 OX=2697049 GN=S PE=4 SV=1	A0A7L9F8J2	S	141,154.70	53.50%	0	0	3	0.01%	2.91%	FQTLLALHR	R	S	97.20%	0.8380	2.8900	1	0	0	1	0	1,098.64	4,933.91	621,494	238	246	A0A7L8AB65,A0A7L8Z0C9,A0A7D7LL25,A0A7G3UUL1,A0A7L9W8W9_NDM
1_Control_Purified_Virions	Surface glycoprotein OS=Severe acute respiratory syndrome coronavirus 2 OX=2697049 GN=S PE=4 SV=1	A0A7L9F8J2	S	141,154.70	53.50%	0	0	3	0.01%	2.91%	KSNLKPFER	R	D	97.20%	0.6230	2.6800	1	0	0	1	0	1,118.63	2,913.98	1,222,680	458	466	A0A7L8AB65,A0A7L8Z0C9,A0A7D7LL25,A0A7L9W8W9_NDM
1_Control_Purified_Virions	Surface glycoprotein OS=Severe acute respiratory syndrome coronavirus 2 OX=2697049 GN=S PE=4 SV=1	A0A7L9F8J2	S	141,154.70	53.50%	0	0	3	0.01%	2.91%	VVVLSFELLHAPATVCGPK	R	K	97.20%	0.7990	4.2600	1	0	0	1	0	2,037.12	6,352.68	1,301,320	510	528	A0A7L8Z0C9,A0A7D7LL25,A0A7L9W8W9_NDM
[illegible]	[illegible]	[illegible]	[illegible]	[illegible]	[illegible]	[illegible]	[illegible]	[illegible]	[illegible]	[illegible]	[illegible]	[illegible]	[illegible]	[illegible]	[illegible]	[illegible]	[illegible]	[illegible]	[illegible]	[illegible]	[illegible]	[illegible]	[illegible]	[illegible]	[illegible]	[illegible]	[illegible]

All Individual Proteins and Peptides

Biological sample category	Protein name	Protein accession number [a]	Alternate ID	Protein molecular mass (Da)	Protein identification probability (Prob)	Exclusive unique peptide count (#Pep)	Exclusive unique spectrum count (#Unique)	Total spectrum count (#Spec)	Percentage of total spectra (%Spec)	Percentage of amino acids identified (%Cov)	Peptide sequence	Previous amino acid	Next amino acid	Best Peptide Identification Probability	Best Sequest deltaCn	Best Sequest XCorr	Best Sequest SpRank	Number of identified +1H spectra	Number of identified +2H spectra	Number of identified +3H spectra	Number of identified +4H spectra	Calculated +1H Peptide Mass (AMU)	Median Retention Time	Total TIC	Peptide start index	Peptide stop index	Other Proteins
3_PNGase-F_Treated_Purified_Virions	Surface glycoprotein OS=Severe acute respiratory syndrome coronavirus 2 OX=2697049 GN=S PE=4 SV=1	A0A7L9W8W9_NDM	S	139,739.00	100.00%	3	3	32	0.14%	29.40%	VVVLSFELLHAPATVCGPK	R	K	96.10%	0.7950	3.4500	1	0	0	1	0	2,037.12	6,368.36	649,757	497	515	A0A7L8Z0C9,A0A7D7LL25,A0A7L9F8J2
3_PNGase-F_Treated_Purified_Virions	Surface glycoprotein OS=Severe acute respiratory syndrome coronavirus 2 OX=2697049 GN=S PE=4 SV=1	A0A7L9W8W9_NDM	S	139,739.00	100.00%	3	3	32	0.14%	29.40%	YNEDGTITDAVDCALDPLSETK	K	C	96.30%	0.8890	4.8500	1	0	1	0	0	2,427.09	6,378.45	1,101,920	266	287	
3_PNGase-F_Treated_Purified_Virions	Surface glycoprotein OS=Severe acute respiratory syndrome coronavirus 2 OX=2697049 GN=S PE=4 SV=1	A0A7L8Z0C9	S	141,155.70	92.90%	0	0	31	0.13%	24.60%	CYGVSPTK	K	L	96.20%	0.6840	1.6500	1	0	1	0	0	911.4292	2,716.87	131,418	379	386	A0A7G3UUL1,A0A7L9W8W9_NDM
3_PNGase-F_Treated_Purified_Virions	Surface glycoprotein OS=Severe acute respiratory syndrome coronavirus 2 OX=2697049 GN=S PE=4 SV=1	A0A7L8Z0C9	S	141,155.70	92.90%	0	0	31	0.13%	24.60%	DIADTTDAVR	R	D	96.80%	0.7680	1.3300	1	0	1	0	0	1,076.52	3,347.32	873,387	568	577	A0A7L8AB65,A0A7G3UUL1,A0A7L9W8W9_NDM
3_PNGase-F_Treated_Purified_Virions	Surface glycoprotein OS=Severe acute respiratory syndrome coronavirus 2 OX=2697049 GN=S PE=4 SV=1	A0A7L8Z0C9	S	141,155.70	92.90%	0	0	31	0.13%	24.60%	FASVYAWNR	R	K	97.00%	0.7890	2.3200	1	0	1	0	0	1,113.55	4,822.20	1,047,410	347	355	A0A7G3UUL1,A0A7L9W8W9_NDM
3_PNGase-F_Treated_Purified_Virions	Surface glycoprotein OS=Severe acute respiratory syndrome coronavirus 2 OX=2697049 GN=S PE=4 SV=1	A0A7L8Z0C9	S	141,155.70	92.90%	0	0	31	0.13%	24.60%	FLPFQQFGR	K	D	97.00%	0.8040	1.7500	1	0	1	0	0	1,139.60	5,858.06	1,321,460	559	567	A0A7L8AB65,A0A7G3UUL1,A0A7L9W8W9_NDM
3_PNGase-F_Treated_Purified_Virions	Surface glycoprotein OS=Severe acute respiratory syndrome coronavirus 2 OX=2697049 GN=S PE=4 SV=1	A0A7L8Z0C9	S	141,155.70	92.90%	0	0	31	0.13%	24.60%	FQTLLALHR	R	S	99.60%	0.7560	2.5300	1	0	0	1	0	1,098.64	4,960.98	435,797	238	246	A0A7L8AB65,A0A7D7LL25,A0A7G3UUL1,A0A7L9W8W9_NDM,A0A7L9F8J2
3_PNGase-F_Treated_Purified_Virions	Surface glycoprotein OS=Severe acute respiratory syndrome coronavirus 2 OX=2697049 GN=S PE=4 SV=1	A0A7L8Z0C9	S	141,155.70	92.90%	0	0	31	0.13%	24.60%	GIYQTSNFR	K	V	97.00%	0.7790	1.8900	1	0	1	0	0	1,085.54	3,814.02	626,867	311	319	A0A7D7LL25,A0A7G3UUL1,A0A7L9W8W9_NDM,A0A7L9F8J2
3_PNGase-F_Treated_Purified_Virions	Surface glycoprotein OS=Severe acute respiratory syndrome coronavirus 2 OX=2697049 GN=S PE=4 SV=1	A0A7L8Z0C9	S	141,155.70	92.90%	0	0	31	0.13%	24.60%	GWIFGTTLDSK	R	T	97.00%	0.8050	2.5300	1	0	2	0	0	1,224.63	6,000.38	705,513	103	113	A0A7L8AB65,A0A7D7LL25,A0A7G3UUL1,A0A7L9W8W9_NDM
3_PNGase-F_Treated_Purified_Virions	Surface glycoprotein OS=Severe acute respiratory syndrome coronavirus 2 OX=2697049 GN=S PE=4 SV=1	A0A7L8Z0C9	S	141,155.70	92.90%	0	0	31	0.13%	24.60%	HTPINLVR	K	D	96.30%	0.7020	1.5600	1	0	1	0	0	949.5578	3,563.24	833,341	207	214	A0A7L8AB65,A0A7L9W8W9_NDM,A0A7L9F8J2
3_PNGase-F_Treated_Purified_Virions	Surface glycoprotein OS=Severe acute respiratory syndrome coronavirus 2 OX=2697049 GN=S PE=4 SV=1	A0A7L8Z0C9	S	141,155.70	92.90%	0	0	31	0.13%	24.60%	IADYNYK	K	L	90.00%	0.7470	1.7600	1	0	1	0	0	886.4305	3,062.68	4,307,390	418	424	A0A7L8AB65,A0A7D7LL25,A0A7G3UUL1,A0A7L9W8W9_NDM
3_PNGase-F_Treated_Purified_Virions	Surface glycoprotein OS=Severe acute respiratory syndrome coronavirus 2 OX=2697049 GN=S PE=4 SV=1	A0A7L8Z0C9	S	141,155.70	92.90%	0	0	31	0.13%	24.60%	KSNLKPFER	R	D	99.60%	0.6260	2.1100	1	0	0	2	0	1,118.63	2,894.99	2,302,180	458	466	A0A7L8AB65,A0A7D7LL25,A0A7L9W8W9_NDM,A0A7L9F8J2
3_PNGase-F_Treated_Purified_Virions	Surface glycoprotein OS=Severe acute respiratory syndrome coronavirus 2 OX=2697049 GN=S PE=4 SV=1	A0A7L8Z0C9	S	141,155.70	92.90%	0	0	31	0.13%	24.60%	LDKVEAEVQIDR	R	L	99.60%	0.5530	2.6300	1	0	0	1	0	1,414.75	4,037.08	355,296	984	995	A0A7L8AB65,A0A7D7LL25,A0A7G3UUL1,A0A7L9W8W9_NDM
3_PNGase-F_Treated_Purified_Virions	Surface glycoprotein OS=Severe acute respiratory syndrome coronavirus 2 OX=2697049 GN=S PE=4 SV=1	A0A7L8Z0C9	S	141,155.70	92.90%	0	0	31	0.13%	24.60%	LIANQFNSAIGK	K	I	95.40%	0.5450	1.8800	1	0	1	0	0	1,275.71	4,590.89	520,886	922	933	A0A7L8AB65,A0A7G3UUL1,A0A7L9W8W9_NDM,A0A7L9F8J2
3_PNGase-F_Treated_Purified_Virions	Surface glycoprotein OS=Severe acute respiratory syndrome coronavirus 2 OX=2697049 GN=S PE=4 SV=1	A0A7L8Z0C9	S	141,155.70	92.90%	0	0	31	0.13%	24.60%	LNDLCFTNVYADSFVIR	K	G	97.00%	0.7610	3.7200	1	0	1	0	0	2,047.00	6,488.96	908,975	387	403	A0A7D7LL25,A0A7L9W8W9_NDM,A0A7L9F8J2
3_PNGase-F_Treated_Purified_Virions	Surface glycoprotein OS=Severe acute respiratory syndrome coronavirus 2 OX=2697049 GN=S PE=4 SV=1	A0A7L8Z0C9	S	141,155.70	92.90%	0	0	31	0.13%	24.60%	LPDDFTGCVIAWNSNNLDSK	K	V	97.00%	0.9420	5.2700	1	0	1	0	0	2,266.04	6,188.35	579,815	425	444	A0A7D5XMN0,A0A7L9W8W9_NDM
3_PNGase-F_Treated_Purified_Virions	Surface glycoprotein OS=Severe acute respiratory syndrome coronavirus 2 OX=2697049 GN=S PE=4 SV=1	A0A7L8Z0C9	S	141,155.70	92.90%	0	0	31	0.13%	24.60%	LQDVVNQNAQALNTLVK	K	Q	97.00%	0.8090	2.7100	1	0	1	0	0	1,868.02	5,457.69	1,055,200	948	964	A0A7D7LL25,A0A7L9W8W9_NDM,A0A7L9F8J2
3_PNGase-F_Treated_Purified_Virions	Surface glycoprotein OS=Severe acute respiratory syndrome coronavirus 2 OX=2697049 GN=S PE=4 SV=1	A0A7L8Z0C9	S	141,155.70	92.90%	0	0	31	0.13%	24.60%	LQSLQTYVTQQLIR	R	A	97.00%	0.8140	3.5900	1	0	1	0	0	1,690.95	6,236.56	2,320,250	1001	1014	A0A7L8AB65,A0A7G3UUL1,A0A7L9W8W9_NDM
3_PNGase-F_Treated_Purified_Virions	Surface glycoprotein OS=Severe acute respiratory syndrome coronavirus 2 OX=2697049 GN=S PE=4 SV=1	A0A7L8Z0C9	S	141,155.70	92.90%	0	0	31	0.13%	24.60%	NIDGYFK	K	I	90.00%	0.6690	1.6600	1	0	1	0	0	856.42	4,236.03	1,065,270	196	202	A0A7L8AB65,A0A7L9W8W9_NDM,A0A7L9F8J2
3_PNGase-F_Treated_Purified_Virions	Surface glycoprotein OS=Severe acute respiratory syndrome coronavirus 2 OX=2697049 GN=S PE=4 SV=1	A0A7L8Z0C9	S	141,155.70	92.90%	0	0	31	0.13%	24.60%	QYGDCLGDIAAR	K	D	97.00%	0.8190	2.9400	1	0	1	0	0	1,338.61	4,436.26	1,260,660	836	847	A0A7L8AB65,A0A7D7LL25,A0A7G3UUL1,A0A7L9W8W9_NDM,A0A7L9F8J2
3_PNGase-F_Treated_Purified_Virions	Surface glycoprotein OS=Severe acute respiratory syndrome coronavirus 2 OX=2697049 GN=S PE=4 SV=1	A0A7L8Z0C9	S	141,155.70	92.90%	0	0	31	0.13%	24.60%	RFDNPVLPFNDGVYFASTEK	K	S	99.60%	0.6540	2.6500	1	0	0	1	0	2,316.13	6,263.08	8,030,750	78	97	A0A7D5XMN0,A0A7L9W8W9_NDM
[illegible]	[illegible]	[illegible]	[illegible]	[illegible]	[illegible]	[illegible]	[illegible]	[illegible]	[illegible]	[illegible]	[illegible]	[illegible]	[illegible]	[illegible]	[illegible]	[illegible]	[illegible]	[illegible]	[illegible]	[illegible]	[illegible]	[illegible]	[illegible]	[illegible]	[illegible]	[illegible]	[illegible]

All Individual Proteins and Peptides

Biological sample category	Protein name	Protein accession number [a]	Alternate ID	Protein molecular mass (Da)	Protein identification probability (Prob)	Exclusive unique peptide count (#Pep)	Exclusive unique spectrum count (#Unique)	Total spectrum count (#Spec)	Percentage of total spectra (%Spec)	Percentage of amino acids identified (%Cov)	Peptide sequence	Previous amino acid	Next amino acid	Best Peptide Identification Probability	Best Sequest deltaCn	Best Sequest XCorr	Best Sequest SpRank	Number of identified +1H spectra	Number of identified +2H spectra	Number of identified +3H spectra	Number of identified +4H spectra	Calculated +1H Peptide Mass (AMU)	Median Retention Time	Total TIC	Peptide start index	Peptide stop index	Other Proteins
4_Inactivated PNGase-F_Treated_Purified_Virions	Nucleocapsid phosphoprotein OS=Severe acute respiratory syndrome coronavirus 2 OX=2697049 GN=N PE=4 SV=1	A0A7L8MNE6	N	45,655.30	87.90%	0	0	35	0.17%	60.10%	ADETQALPQR	K	Q	91.40%	0.8220	2.7500	1	0	2	0	0	1,128.56	2,943.92	647,438	376	385	
4_Inactivated PNGase-F_Treated_Purified_Virions	Nucleocapsid phosphoprotein OS=Severe acute respiratory syndrome coronavirus 2 OX=2697049 GN=N PE=4 SV=1	A0A7L8MNE6	N	45,655.30	87.90%	0	0	35	0.17%	60.10%	DQVILLNK	K	H	91.40%	0.6620	1.9600	1	0	1	0	0	942.562	4,102.00	235,857	348	355	
4_Inactivated PNGase-F_Treated_Purified_Virions	Nucleocapsid phosphoprotein OS=Severe acute respiratory syndrome coronavirus 2 OX=2697049 GN=N PE=4 SV=1	A0A7L8MNE6	N	45,655.30	87.90%	0	0	35	0.17%	60.10%	GFYAEGSR	K	G	91.40%	0.7530	1.8000	1	0	1	0	0	886.4053	2,975.89	2,740,770	170	177	
4_Inactivated PNGase-F_Treated_Purified_Virions	Nucleocapsid phosphoprotein OS=Severe acute respiratory syndrome coronavirus 2 OX=2697049 GN=N PE=4 SV=1	A0A7L8MNE6	N	45,655.30	87.90%	0	0	35	0.17%	60.10%	GPEQTQGNFGDQELIR	R	Q	91.40%	0.7480	2.3600	1	0	1	0	0	1,788.85	4,592.04	47,985	278	293	A0A7M2G704
4_Inactivated PNGase-F_Treated_Purified_Virions	Nucleocapsid phosphoprotein OS=Severe acute respiratory syndrome coronavirus 2 OX=2697049 GN=N PE=4 SV=1	A0A7L8MNE6	N	45,655.30	87.90%	0	0	35	0.17%	60.10%	GQGVPINTNSSPDDQIGYYR	R	R	91.40%	0.8900	4.2700	1	0	2	0	0	2,181.02	4,761.81	1,043,460	69	88	
4_Inactivated PNGase-F_Treated_Purified_Virions	Nucleocapsid phosphoprotein OS=Severe acute respiratory syndrome coronavirus 2 OX=2697049 GN=N PE=4 SV=1	A0A7L8MNE6	N	45,655.30	87.90%	0	0	35	0.17%	60.10%	GQQQQGQTVTK	K	K	91.40%	0.5790	1.9800	1	0	1	0	0	1,202.61	1,276.48	509,413	238	248	
4_Inactivated PNGase-F_Treated_Purified_Virions	Nucleocapsid phosphoprotein OS=Severe acute respiratory syndrome coronavirus 2 OX=2697049 GN=N PE=4 SV=1	A0A7L8MNE6	N	45,655.30	87.90%	0	0	35	0.17%	60.10%	HWPQIAQFAPSASAFFGMSR	K	I	97.90%	0.7210	5.0400	1	0	0	1	0	2,236.08	6,561.18	249,033	300	319	A0A7G8ATM2,A0A7D7NMS6,A0A7H1CZG8,A0A7M2G704
4_Inactivated PNGase-F_Treated_Purified_Virions	Nucleocapsid phosphoprotein OS=Severe acute respiratory syndrome coronavirus 2 OX=2697049 GN=N PE=4 SV=1	A0A7L8MNE6	N	45,655.30	87.90%	0	0	35	0.17%	60.10%	IGMEVTPSGTWLTYTGAIK	R	L	91.40%	0.6220	1.7100	1	0	1	0	0	2,025.04	6,309.47	296,875	320	338	A0A7M2G704
4_Inactivated PNGase-F_Treated_Purified_Virions	Nucleocapsid phosphoprotein OS=Severe acute respiratory syndrome coronavirus 2 OX=2697049 GN=N PE=4 SV=1	A0A7L8MNE6	N	45,655.30	87.90%	0	0	35	0.17%	60.10%	ITFGGPSDSTGSNQNGER	R	S	91.40%	0.8330	2.9600	1	0	2	0	0	1,823.82	3,674.32	185,063	15	32	
4_Inactivated PNGase-F_Treated_Purified_Virions	Nucleocapsid phosphoprotein OS=Severe acute respiratory syndrome coronavirus 2 OX=2697049 GN=N PE=4 SV=1	A0A7L8MNE6	N	45,655.30	87.90%	0	0	35	0.17%	60.10%	KADETQALPQR	K	Q	91.40%	0.8020	3.7400	1	0	1	0	0	1,256.66	2,751.47	421,210	375	385	
4_Inactivated PNGase-F_Treated_Purified_Virions	Nucleocapsid phosphoprotein OS=Severe acute respiratory syndrome coronavirus 2 OX=2697049 GN=N PE=4 SV=1	A0A7L8MNE6	N	45,655.30	87.90%	0	0	35	0.17%	60.10%	KKADETQALPQR	K	Q	92.00%	0.3330	2.6800	1	0	0	1	0	1,384.75	2,617.48	878,963	374	385	
4_Inactivated PNGase-F_Treated_Purified_Virions	Nucleocapsid phosphoprotein OS=Severe acute respiratory syndrome coronavirus 2 OX=2697049 GN=N PE=4 SV=1	A0A7L8MNE6	N	45,655.30	87.90%	0	0	35	0.17%	60.10%	KQQTVTLLPAADLDDFSK	K	Q	97.90%	0.8480	4.5200	1	0	0	1	0	1,990.05	6,013.22	758,831	388	405	A0A7M2G704
4_Inactivated PNGase-F_Treated_Purified_Virions	Nucleocapsid phosphoprotein OS=Severe acute respiratory syndrome coronavirus 2 OX=2697049 GN=N PE=4 SV=1	A0A7L8MNE6	N	45,655.30	87.90%	0	0	35	0.17%	60.10%	LDDKDPNFK	K	D	91.90%	0.4810	1.0300	1	0	0	1	0	1,091.54	2,967.97	248,501	339	347	
4_Inactivated PNGase-F_Treated_Purified_Virions	Nucleocapsid phosphoprotein OS=Severe acute respiratory syndrome coronavirus 2 OX=2697049 GN=N PE=4 SV=1	A0A7L8MNE6	N	45,655.30	87.90%	0	0	35	0.17%	60.10%	LDDKDPNFKDQVILLNK	K	H	97.90%	0.7990	4.9900	1	0	0	2	3	2,015.08	5,085.15	2,162,310	339	355	A0A7M2G704
4_Inactivated PNGase-F_Treated_Purified_Virions	Nucleocapsid phosphoprotein OS=Severe acute respiratory syndrome coronavirus 2 OX=2697049 GN=N PE=4 SV=1	A0A7L8MNE6	N	45,655.30	87.90%	0	0	35	0.17%	60.10%	LNQLESK	R	M	90.00%	0.5530	2.0400	1	0	1	0	0	831.4571	2,279.28	1,212,400	227	233	
4_Inactivated PNGase-F_Treated_Purified_Virions	Nucleocapsid phosphoprotein OS=Severe acute respiratory syndrome coronavirus 2 OX=2697049 GN=N PE=4 SV=1	A0A7L8MNE6	N	45,655.30	87.90%	0	0	35	0.17%	60.10%	MAGNGGDAALALLLLDR	R	L	91.40%	0.9120	5.0000	1	0	1	0	0	1,670.89	6,598.80	997,550	210	226	
4_Inactivated PNGase-F_Treated_Purified_Virions	Nucleocapsid phosphoprotein OS=Severe acute respiratory syndrome coronavirus 2 OX=2697049 GN=N PE=4 SV=1	A0A7L8MNE6	N	45,655.30	87.90%	0	0	35	0.17%	60.10%	MKDLSPR	K	W	90.00%	0.6790	2.1200	1	0	2	0	0	846.4503	2,457.65	519,381	101	107	
4_Inactivated PNGase-F_Treated_Purified_Virions	Nucleocapsid phosphoprotein OS=Severe acute respiratory syndrome coronavirus 2 OX=2697049 GN=N PE=4 SV=1	A0A7L8MNE6	N	45,655.30	87.90%	0	0	35	0.17%	60.10%	NPANNAAIVLQLPQGTTLPK	R	G	97.90%	0.7110	3.5000	1	0	2	2	0	2,060.15	5,994.97	937,113	150	169	
[illegible]	[illegible]	[illegible]	[illegible]	[illegible]	[illegible]	[illegible]	[illegible]	[illegible]	[illegible]	[illegible]	[illegible]	[illegible]	[illegible]	[illegible]	[illegible]	[illegible]	[illegible]	[illegible]	[illegible]	[illegible]	[illegible]	[illegible]	[illegible]	[illegible]	[illegible]	[illegible]	[illegible]

[a] NDM, Asn to Asp modification. The NDM annotation was manually added following the accession number of the original Spike sequence to indicate that the asparagine (N) residues were in-silico-modified to aspartic acid (D) by an in-house script, to account for an experimental *N*-deglycosylation by PNGase-F treatment.
