# Supplementary material for: Inhibition of Protein N-Glycosylation Blocks SARS-CoV-2 Infection
Source: mBio. 2022 Feb 15;13(1):e03718-21. doi: 10.1128/mbio.03718-21 (PMC8844921; doi:10.1128/mbio.03718-21)
Supplement: DATA SET S3 [file mbio.03718-21-sd003.pdf]

Identified Spike Proteins

Bio Sample Category / Sequence Coverage [a,b,c]	Protein	Accession Number [b]	Bio Sample / Category	Protein identification probability (Prob)	Exclusive unique peptide count (#Pep)	Exclusive unique spectrum count (#Unique)	Total spectrum count (#Spec)	Percentage of total spectra (%Spec)	Percentage of amino acids identified (%Cov)	Protein molecular mass
**1_Control_Purified_Virions**										
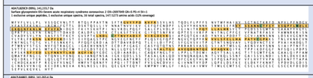	Surface glycoprotein OS=Severe acute respiratory syndrome coronavirus 2 OX=2697049 GN=S PE=4 SV=1	A0A7L8Z0C9	1_Control_Purified_Virions	99%	1	1	16	0.0341%	11.5%	141 kDa
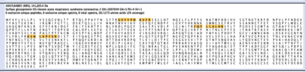	Surface glycoprotein OS=Severe acute respiratory syndrome coronavirus 2 OX=2697049 GN=S PE=4 SV=1	A0A7L8AB65	1_Control_Purified_Virions	88%	0	0	8	0.0170%	2.2%	141 kDa
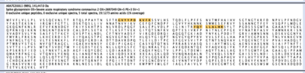	Spike glycoprotein OS=Severe acute respiratory syndrome coronavirus 2 OX=2697049 GN=S PE=3 SV=1	A0A7G3UUL1	1_Control_Purified_Virions	98%	0	0	5	0.0106%	1.5%	141 kDa
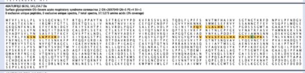	Surface glycoprotein OS=Severe acute respiratory syndrome coronavirus 2 OX=2697049 GN=S PE=4 SV=1	A0A7L9F8J2	1_Control_Purified_Virions	61%	0	0	7	0.0149%	2.9%	141 kDa
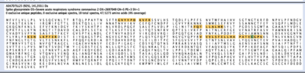	Spike glycoprotein OS=Severe acute respiratory syndrome coronavirus 2 OX=2697049 GN=S PE=3 SV=1	A0A7D7LL25	1_Control_Purified_Virions	97%	0	0	10	0.0213%	3.7%	141 kDa
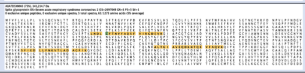	Spike glycoprotein OS=Severe acute respiratory syndrome coronavirus 2 OX=2697049 GN=S PE=3 SV=1	A0A7D5XMN0	1_Control_Purified_Virions	75%	0	0	5	0.0106%	4.9%	141 kDa
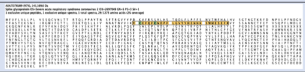	Spike glycoprotein OS=Severe acute respiratory syndrome coronavirus 2 OX=2697049 GN=S PE=3 SV=1	A0A7D7XU89	1_Control_Purified_Virions	97%	1	1	1	0.0021%	2.3%	141 kDa
**2_Mock_Treated_Purified_Virions**										
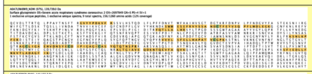	Surface glycoprotein OS=Severe acute respiratory syndrome coronavirus 2 OX=2697049 GN=S PE=4 SV=1	A0A7L9W8W9_NDM	2_Mock_Treated_Purified_Virions	97%	1	1	9	0.0190%	12.4%	140 kDa
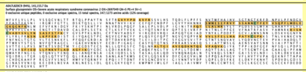	Surface glycoprotein OS=Severe acute respiratory syndrome coronavirus 2 OX=2697049 GN=S PE=4 SV=1	A0A7L8Z0C9	2_Mock_Treated_Purified_Virions	94%	0	0	15	0.0317%	11.5%	141 kDa
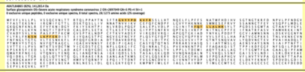	Surface glycoprotein OS=Severe acute respiratory syndrome coronavirus 2 OX=2697049 GN=S PE=4 SV=1	A0A7L8AB65	2_Mock_Treated_Purified_Virions	82%	0	0	8	0.0169%	2.2%	141 kDa
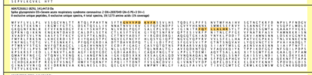	Spike glycoprotein OS=Severe acute respiratory syndrome coronavirus 2 OX=2697049 GN=S PE=3 SV=1	A0A7G3UUL1	2_Mock_Treated_Purified_Virions	82%	0	0	4	0.0085%	1.5%	141 kDa
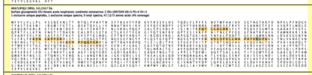	Surface glycoprotein OS=Severe acute respiratory syndrome coronavirus 2 OX=2697049 GN=S PE=4 SV=1	A0A7L9F8J2	2_Mock_Treated_Purified_Virions	98%	1	1	9	0.0190%	3.7%	141 kDa
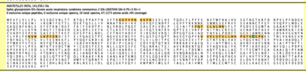	Spike glycoprotein OS=Severe acute respiratory syndrome coronavirus 2 OX=2697049 GN=S PE=3 SV=1	A0A7D7LL25	2_Mock_Treated_Purified_Virions	92%	0	0	10	0.0212%	3.7%	141 kDa
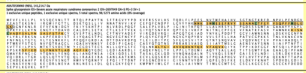	Spike glycoprotein OS=Severe acute respiratory syndrome coronavirus 2 OX=2697049 GN=S PE=3 SV=1	A0A7D5XMN0	2_Mock_Treated_Purified_Virions	96%	1	1	5	0.0106%	7.7%	141 kDa
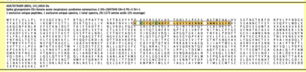	Spike glycoprotein OS=Severe acute respiratory syndrome coronavirus 2 OX=2697049 GN=S PE=3 SV=1	A0A6N1WN54	2_Mock_Treated_Purified_Virions	97%	1	1	2	0.0042%	4.6%	141 kDa
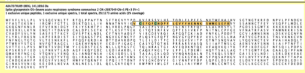	Spike glycoprotein OS=Severe acute respiratory syndrome coronavirus 2 OX=2697049 GN=S PE=3 SV=1	A0A7D7XU89	2_Mock_Treated_Purified_Virions	86%	1	1	1	0.0021%	2.3%	141 kDa
**3_PNGase-F_Treated_Purified_Virions**										
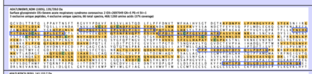	Surface glycoprotein OS=Severe acute respiratory syndrome coronavirus 2 OX=2697049 GN=S PE=4 SV=1	A0A7L9W8W9_NDM	3_PNGase-F_Treated_Purified_Virions	100%	3	4	66	0.1384%	37.1%	140 kDa
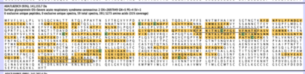	Surface glycoprotein OS=Severe acute respiratory syndrome coronavirus 2 OX=2697049 GN=S PE=4 SV=1	A0A7L8Z0C9	3_PNGase-F_Treated_Purified_Virions	93%	0	0	59	0.1237%	30.7%	141 kDa
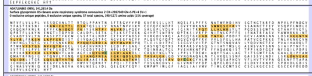	Surface glycoprotein OS=Severe acute respiratory syndrome coronavirus 2 OX=2697049 GN=S PE=4 SV=1	A0A7L8AB65	3_PNGase-F_Treated_Purified_Virions	98%	0	0	37	0.0776%	14.9%	141 kDa
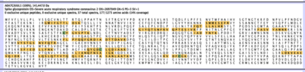	Spike glycoprotein OS=Severe acute respiratory syndrome coronavirus 2 OX=2697049 GN=S PE=3 SV=1	A0A7G3UUL1	3_PNGase-F_Treated_Purified_Virions	100%	0	0	37	0.0776%	13.9%	141 kDa
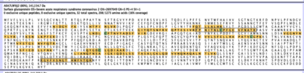	Surface glycoprotein OS=Severe acute respiratory syndrome coronavirus 2 OX=2697049 GN=S PE=4 SV=1	A0A7L9F8J2	3_PNGase-F_Treated_Purified_Virions	80%	0	0	32	0.0671%	16.3%	141 kDa
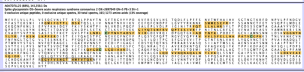	Spike glycoprotein OS=Severe acute respiratory syndrome coronavirus 2 OX=2697049 GN=S PE=3 SV=1	A0A7D7LL25	3_PNGase-F_Treated_Purified_Virions	88%	0	0	30	0.0629%	13.0%	141 kDa
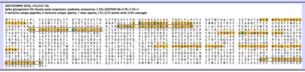	Spike glycoprotein OS=Severe acute respiratory syndrome coronavirus 2 OX=2697049 GN=S PE=3 SV=1	A0A7D5XMN0	3_PNGase-F_Treated_Purified_Virions	85%	0	0	7	0.0147%	13.5%	141 kDa

4_Inactivated PNGase-F_Treated_Purified_Virions										
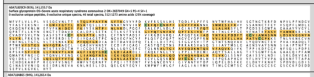	Surface glycoprotein OS=Severe acute respiratory syndrome coronavirus 2 OX=2697049 GN=S PE=4 SV=1	A0A7L8Z0C9	4_Inactivated_PNGase-F_Treated_Purified_Virions	91%	0	0	46	0.1073%	24.5%	141 kDa
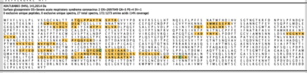	Surface glycoprotein OS=Severe acute respiratory syndrome coronavirus 2 OX=2697049 GN=S PE=4 SV=1	A0A7L8AB65	4_Inactivated_PNGase-F_Treated_Purified_Virions	94%	0	0	27	0.0630%	13.5%	141 kDa
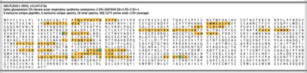	Spike glycoprotein OS=Severe acute respiratory syndrome coronavirus 2 OX=2697049 GN=S PE=3 SV=1	A0A7G3UUL1	4_Inactivated_PNGase-F_Treated_Purified_Virions	94%	0	0	28	0.0653%	13.2%	141 kDa
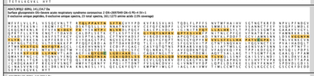	Surface glycoprotein OS=Severe acute respiratory syndrome coronavirus 2 OX=2697049 GN=S PE=4 SV=1	A0A7L9F8J2	4_Inactivated_PNGase-F_Treated_Purified_Virions	69%	0	0	23	0.0537%	12.6%	141 kDa
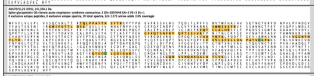	Spike glycoprotein OS=Severe acute respiratory syndrome coronavirus 2 OX=2697049 GN=S PE=3 SV=1	A0A7D7LL25	4_Inactivated_PNGase-F_Treated_Purified_Virions	93%	0	0	19	0.0443%	9.7%	141 kDa
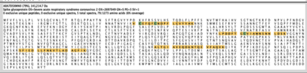	Spike glycoprotein OS=Severe acute respiratory syndrome coronavirus 2 OX=2697049 GN=S PE=3 SV=1	A0A7D5XMN0	4_Inactivated_PNGase-F_Treated_Purified_Virions	79%	0	0	5	0.0117%	6.2%	141 kDa

[a] All identified peptides are highlighted in orange. Modified Cys residues are highlighted in green. *N*-Deglycosylated peptides are indicated as dark blue-lined retangles. Identified non-*N*-glycosylated peptides are indicated as gray-lined retangles.

[b] NDM, Asn to Asp modification. The NDM annotation was manually added following the accession number of the original Spike sequence to indicate that the asparagine (N) residues were in-silico-modified to aspartic acid (D) by an in-house script, to account for an experimental *N*-deglycosylation by PNGase-F treatment.

[c] Note that all sequences containing NDMs do not contain the ER Signal Peptide (MFVFLVLLPLVSS).
