# Supplementary material for: Inhibition of Protein N-Glycosylation Blocks SARS-CoV-2 Infection
Source: mBio. 2022 Feb 15;13(1):e03718-21. doi: 10.1128/mbio.03718-21 (PMC8844921; doi:10.1128/mbio.03718-21)
Supplement: DATA SET S4 [file mbio.03718-21-sd004.pdf]

Identified Spike Peptides

Valid	Weight	Sequence [a,b]	Protein identification probability (Prob)	SEQUEST XCorr	SEQUEST deltaCn	Modifications	Observed Mass (Da)	Actual Mass (Da)	Charge	Delta Da	Delta PPM	Retention Time	# Other Proteins	Other Proteins	Total Ion Chromatogram (TIC)	Spectrum ID	Peptide Start Index	Peptide Stop Index
1_Control_Purified_Virions																		
Surface glycoprotein OS=Severe acute respiratory syndrome coronavirus 2 OX=2697049 GN=S PE=4 SV=1 Accession A0A7L9W8W9_NDM [c]																		
TRUE	0.0067	(R)GVYYPDKVFR(S)	99%	2.0205	0.7473		415.2208	1242.6405	3	0.0007	0.5574	4,530.86	4	A0A7L8AB65,A0A7L8Z0C9,A0A7D7LL25,A0A7G3UUL1	1,168,410	1_Control_Purified_Virions_1-16007-24431_24431	35	44
TRUE	0.0067	(R)GVYYPDKVFR(S)	97%	2.0155	0.7700		415.2204	1242.6393	3	-0.0005	-0.3996	4,504.70	4	A0A7L8AB65,A0A7L8Z0C9,A0A7D7LL25,A0A7G3UUL1	1,577,920	1_Control_Purified_Virions_2-15451-24244_24244	35	44
TRUE	0.0067	(R)GVYYPDKVFR(S)	97%	2.0658	0.6332		415.2206	1242.6401	3	0.0003	0.2629	4,483.81	4	A0A7L8AB65,A0A7L8Z0C9,A0A7D7LL25,A0A7G3UUL1	707,501	1_Control_Purified_Virions_2-15337-24105_24105	35	44
TRUE	0.0070	(R)FQTLLALHR(S)	99%	2.3250	0.7606		366.8858	1097.6356	3	0.0009	0.8287	4,952.73	5	A0A7L8AB65,A0A7L8Z0C9,A0A7D7LL25,A0A7G3UUL1,A0A7L9F8J2	383,852	1_Control_Purified_Virions_1-18172-27118_27118	238	246
TRUE	0.0070	(R)FQTLLALHR(S)	97%	2.8920	0.8377		366.8854	1097.6344	3	-0.0003	-0.2547	4,933.91	5	A0A7L8AB65,A0A7L8Z0C9,A0A7D7LL25,A0A7G3UUL1,A0A7L9F8J2	621,494	1_Control_Purified_Virions_2-17652-26974_26974	238	246
TRUE	0.0161	(K)LNDLcFTNVYADSFVIRGDEVR(Q)	99%	3.7817	0.8379	Carbamidomethyl (+57)	868.4216	2602.2431	3	-0.0057	-2.1790	6,394.46	2	A0A7L8Z0C9,A0A7D5XMN0	832,082	1_Control_Purified_Virions_1-26323-36625_36625	387	408
TRUE	0.0161	(K)LNDLcFTNVYADSFVIRGDEVR(Q)	97%	3.4239	0.8404	Carbamidomethyl (+57)	868.4241	2602.2506	3	0.0018	0.7050	6,390.85	2	A0A7L8Z0C9,A0A7D5XMN0	718,966	1_Control_Purified_Virions_2-25823-36553_36553	387	408
TRUE	0.0076	(R)KSNLKPFER(D)	99%	2.0241	0.5636		373.5490	1117.6253	3	0.0008	0.7384	2,880.55	4	A0A7L8AB65,A0A7L8Z0C9,A0A7D7LL25,A0A7L9F8J2	1,734,420	1_Control_Purified_Virions_1-8460-14475_14475	458	466
TRUE	0.0076	(R)KSNLKPFER(D)	97%	2.6823	0.6230		373.5486	1117.6241	3	-0.0004	-0.3256	2,913.98	4	A0A7L8AB65,A0A7L8Z0C9,A0A7D7LL25,A0A7L9F8J2	1,222,680	1_Control_Purified_Virions_2-8202-14661_14661	458	466
TRUE	0.0076	(R)KSNLKPFER(D)	94%	1.4772	0.4827		373.5489	1117.6248	3	0.0004	0.3292	2,900.94	4	A0A7L8AB65,A0A7L8Z0C9,A0A7D7LL25,A0A7L9F8J2	852,608	1_Control_Purified_Virions_1-8548-14593_14593	458	466
TRUE	0.0058	(R)VVVLSFELLHAPATVcGPK(K)	99%	4.0406	0.7204	Carbamidomethyl (+57)	679.7117	2036.1134	3	0.0003	0.1612	6,357.62	3	A0A7L8Z0C9,A0A7D7LL25,A0A7L9F8J2	2,207,880	1_Control_Purified_Virions_1-26096-36375_36375	510	528
TRUE	0.0058	(R)VVVLSFELLHAPATVcGPK(K)	97%	4.2563	0.7988	Carbamidomethyl (+57)	679.7123	2036.1152	3	0.0022	1.0600	6,352.68	3	A0A7L8Z0C9,A0A7D7LL25,A0A7L9F8J2	1,301,320	1_Control_Purified_Virions_2-25588-36294_36294	510	528
TRUE	0.0187	(R)ALTGIAVEQDKNTQEVFAQVK(Q)	99%	5.5613	0.7991		763.7410	2288.2013	3	-0.0002	-0.0943	5,435.09	2	A0A7L8Z0C9,A0A7D5XMN0	1,868,740	1_Control_Purified_Virions_1-20775-30245_30245	766	786
TRUE	0.0187	(R)ALTGIAVEQDKNTQEVFAQVK(Q)	97%	5.8025	0.7940		763.7410	2288.2011	3	-0.0004	-0.1742	5,424.23	2	A0A7L8Z0C9,A0A7D5XMN0	2,331,850	1_Control_Purified_Virions_2-20297-30148_30148	766	786
TRUE	0.0185	(R)SFIEDLLFNKVTLADAGFIK(Q)	97%	4.2669	0.8960		747.7430	2240.2073	3	-0.0023	-1.0140	6,720.77	2	A0A7L8Z0C9,A0A7D5XMN0	630,101	1_Control_Purified_Virions_2-27828-38792_38792	816	835
Surface glycoprotein OS=Severe acute respiratory syndrome coronavirus 2 OX=2697049 GN=S PE=4 SV=1 Accession A0A7L8Z0C9																		
TRUE	0.0666	(R)GVYYPDKVFR(S)	99%	2.0205	0.7473		415.2208	1242.6405	3	0.0007	0.5574	4,530.86	4	A0A7L8AB65,A0A7D7LL25,A0A7G3UUL1,A0A7L9W8W9_NDM	1,168,410	1_Control_Purified_Virions_1-16007-24431_24431	35	44
TRUE	0.0666	(R)GVYYPDKVFR(S)	97%	2.0155	0.7700		415.2204	1242.6393	3	-0.0005	-0.3996	4,504.70	4	A0A7L8AB65,A0A7D7LL25,A0A7G3UUL1,A0A7L9W8W9_NDM	1,577,920	1_Control_Purified_Virions_2-15451-24244_24244	35	44
TRUE	0.0666	(R)GVYYPDKVFR(S)	97%	2.0658	0.6332		415.2206	1242.6401	3	0.0003	0.2629	4,483.81	4	A0A7L8AB65,A0A7D7LL25,A0A7G3UUL1,A0A7L9W8W9_NDM	707,501	1_Control_Purified_Virions_2-15337-24105_24105	35	44
TRUE	1.0000	(R)VYSSANNcTFEYVSQPFLmDLEGKQGNFKNLMEFVFK(N)	93%	0.8057	0.5858	Carbamidomethyl (+57), Oxidation (+16)	1100.2654	4397.0324	4	-0.0160	-3.6450	6,531.57	0		422,473	1_Control_Purified_Virions_2-26668-37505_37505	159	195
TRUE	0.0690	(R)FQTLLALHR(S)	99%	2.3250	0.7606		366.8858	1097.6356	3	0.0009	0.8287	4,952.73	5	A0A7L8AB65,A0A7D7LL25,A0A7G3UUL1,A0A7L9W8W9_NDM,A0A7L9F8J2	383,852	1_Control_Purified_Virions_1-18172-27118_27118	238	246
TRUE	0.0690	(R)FQTLLALHR(S)	97%	2.8920	0.8377		366.8854	1097.6344	3	-0.0003	-0.2547	4,933.91	5	A0A7L8AB65,A0A7D7LL25,A0A7G3UUL1,A0A7L9W8W9_NDM,A0A7L9F8J2	621,494	1_Control_Purified_Virions_2-17652-26974_26974	238	246
TRUE	0.1602	(K)LNDLcFTNVYADSFVIRGDEVR(Q)	99%	3.7817	0.8379	Carbamidomethyl (+57)	868.4216	2602.2431	3	-0.0057	-2.1790	6,394.46	2	A0A7D5XMN0,A0A7L9W8W9_NDM	832,082	1_Control_Purified_Virions_1-26323-36625_36625	387	408
TRUE	0.1602	(K)LNDLcFTNVYADSFVIRGDEVR(Q)	97%	3.4239	0.8404	Carbamidomethyl (+57)	868.4241	2602.2506	3	0.0018	0.7050	6,390.85	2	A0A7D5XMN0,A0A7L9W8W9_NDM	718,966	1_Control_Purified_Virions_2-25823-36553_36553	387	408
TRUE	0.0754	(R)KSNLKPFER(D)	99%	2.0241	0.5636		373.5490	1117.6253	3	0.0008	0.7384	2,880.55	4	A0A7L8AB65,A0A7D7LL25,A0A7L9W8W9_NDM,A0A7L9F8J2	1,734,420	1_Control_Purified_Virions_1-8460-14475_14475	458	466
TRUE	0.0754	(R)KSNLKPFER(D)	97%	2.6823	0.6230		373.5486	1117.6241	3	-0.0004	-0.3256	2,913.98	4	A0A7L8AB65,A0A7D7LL25,A0A7L9W8W9_NDM,A0A7L9F8J2	1,222,680	1_Control_Purified_Virions_2-8202-14661_14661	458	466
TRUE	0.0754	(R)KSNLKPFER(D)	94%	1.4772	0.4827		373.5489	1117.6248	3	0.0004	0.3292	2,900.94	4	A0A7L8AB65,A0A7D7LL25,A0A7L9W8W9_NDM,A0A7L9F8J2	852,608	1_Control_Purified_Virions_1-8548-14593_14593	458	466
TRUE	0.0577	(R)VVVLSFELLHAPATVcGPK(K)	99%	4.0406	0.7204	Carbamidomethyl (+57)	679.7117	2036.1134	3	0.0003	0.1612	6,357.62	3	A0A7D7LL25,A0A7L9W8W9_NDM,A0A7L9F8J2	2,207,880	1_Control_Purified_Virions_1-26096-36375_36375	510	528
TRUE	0.0577	(R)VVVLSFELLHAPATVcGPK(K)	97%	4.2563	0.7988	Carbamidomethyl (+57)	679.7123	2036.1152	3	0.0022	1.0600	6,352.68	3	A0A7D7LL25,A0A7L9W8W9_NDM,A0A7L9F8J2	1,301,320	1_Control_Purified_Virions_2-25588-36294_36294	510	528
TRUE	0.1857	(R)ALTGIAVEQDKNTQEVFAQVK(Q)	99%	5.5613	0.7991		763.7410	2288.2013	3	-0.0002	-0.0943	5,435.09	2	A0A7D5XMN0,A0A7L9W8W9_NDM	1,868,740	1_Control_Purified_Virions_1-20775-30245_30245	766	786
TRUE	0.1857	(R)ALTGIAVEQDKNTQEVFAQVK(Q)	97%	5.8025	0.7940		763.7410	2288.2011	3	-0.0004	-0.1742	5,424.23	2	A0A7D5XMN0,A0A7L9W8W9_NDM	2,331,850	1_Control_Purified_Virions_2-20297-30148_30148	766	786
TRUE	0.1841	(R)SFIEDLLFNKVTLADAGFIK(Q)	97%	4.2669	0.8960		747.7430	2240.2073	3	-0.0023	-1.0140	6,720.77	2	A0A7D5XMN0,A0A7L9W8W9_NDM	630,101	1_Control_Purified_Virions_2-27828-38792_38792	816	835
Surface glycoprotein OS=Severe acute respiratory syndrome coronavirus 2 OX=2697049 GN=S PE=4 SV=1 Accession A0A7L8AB65																		
TRUE	0.1513	(R)GVYYPDKVFR(S)	99%	2.0205	0.7473		415.2208	1242.6405	3	0.0007	0.5574	4,530.86	4	A0A7L8Z0C9,A0A7D7LL25,A0A7G3UUL1,A0A7L9W8W9_NDM	1,168,410	1_Control_Purified_Virions_1-16007-24431_24431	35	44
TRUE	0.1513	(R)GVYYPDKVFR(S)	97%	2.0155	0.7700		415.2204	1242.6393	3	-0.0005	-0.3996	4,504.70	4	A0A7L8Z0C9,A0A7D7LL25,A0A7G3UUL1,A0A7L9W8W9_NDM	1,577,920	1_Control_Purified_Virions_2-15451-24244_24244	35	44
TRUE	0.1513	(R)GVYYPDKVFR(S)	97%	2.0658	0.6332		415.2206	1242.6401	3	0.0003	0.2629	4,483.81	4	A0A7L8Z0C9,A0A7D7LL25,A0A7G3UUL1,A0A7L9W8W9_NDM	707,501	1_Control_Purified_Virions_2-15337-24105_24105	35	44
TRUE	0.1567	(R)FQTLLALHR(S)	99%	2.3250	0.7606		366.8858	1097.6356	3	0.0009	0.8287	4,952.73	5	A0A7L8Z0C9,A0A7D7LL25,A0A7G3UUL1,A0A7L9W8W9_NDM,A0A7L9F8J2	383,852	1_Control_Purified_Virions_1-18172-27118_27118	238	246
TRUE	0.1567	(R)FQTLLALHR(S)	97%	2.8920	0.8377		366.8854	1097.6344	3	-0.0003	-0.2547	4,933.91	5	A0A7L8Z0C9,A0A7D7LL25,A0A7G3UUL1,A0A7L9W8W9_NDM,A0A7L9F8J2	621,494	1_Control_Purified_Virions_2-17652-26974_26974	238	246
TRUE	0.1713	(R)KSNLKPFER(D)	99%	2.0241	0.5636		373.5490	1117.6253	3	0.0008	0.7384	2,880.55	4	A0A7L8Z0C9,A0A7D7LL25,A0A7L9W8W9_NDM,A0A7L9F8J2	1,734,420	1_Control_Purified_Virions_1-8460-14475_14475	458	466
TRUE	0.1713	(R)KSNLKPFER(D)	97%	2.6823	0.6230		373.5486	1117.6241	3	-0.0004	-0.3256	2,913.98	4	A0A7L8Z0C9,A0A7D7LL25,A0A7L9W8W9_NDM,A0A7L9F8J2	1,222,680	1_Control_Purified_Virions_2-8202-14661_14661	458	466
TRUE	0.1713	(R)KSNLKPFER(D)	94%	1.4772	0.4827		373.5489	1117.6248	3	0.0004	0.3292	2,900.94	4	A0A7L8Z0C9,A0A7D7LL25,A0A7L9W8W9_NDM,A0A7L9F8J2	852,608	1_Control_Purified_Virions_1-8548-14593_14593	458	466
Spike glycoprotein OS=Severe acute respiratory syndrome coronavirus 2 OX=2697049 GN=S PE=3 SV=1 Accession A0A7G3UUL1																		
TRUE	0.1630	(R)GVYYPDKVFR(S)	99%	2.0205	0.7473		415.2208	1242.6405	3	0.0007	0.5574	4,530.86	4	A0A7L8AB65,A0A7L8Z0C9,A0A7D7LL25,A0A7L9W8W9_NDM	1,168,410	1_Control_Purified_Virions_1-16007-24431_24431	35	44
TRUE	0.1630	(R)GVYYPDKVFR(S)	97%	2.0155	0.7700		415.2204	1242.6393	3	-0.0005	-0.3996	4,504.70	4	A0A7L8AB65,A0A7L8Z0C9,A0A7D7LL25,A0A7L9W8W9_NDM	1,577,920	1_Control_Purified_Virions_2-15451-24244_24244	35	44
TRUE	0.1630	(R)GVYYPDKVFR(S)	97%	2.0658	0.6332		415.2206	1242.6401	3	0.0003	0.2629	4,483.81	4	A0A7L8AB65,A0A7L8Z0C9,A0A7D7LL25,A0A7L9W8W9_NDM	707,501	1_Control_Purified_Virions_2-15337-24105_24105	35	44
TRUE	0.1688	(R)FQTLLALHR(S)	99%	2.3250	0.7606		366.8858	1097.6356	3	0.0009	0.8287	4,952.73	5	A0A7L8AB65,A0A7L8Z0C9,A0A7D7LL25,A0A7L9W8W9_NDM,A0A7L9F8J2	383,852	1_Control_Purified_Virions_1-18172-27118_27118	238	246
TRUE	0.1688	(R)FQTLLALHR(S)	97%	2.8920	0.8377		366.8854	1097.6344	3	-0.0003	-0.2547	4,933.91	5	A0A7L8AB65,A0A7L8Z0C9,A0A7D7LL25,A0A7L9W8W9_NDM,A0A7L9F8J2	621,494	1_Control_Purified_Virions_2-17652-26974_26974	238	246
Surface glycoprotein OS=Severe acute respiratory syndrome coronavirus 2 OX=2697049 GN=S PE=4 SV=1 Accession A0A7L9F8J2																		
TRUE	0.0719	(R)FQTLLALHR(S)	99%	2.3250	0.7606		366.8858	1097.6356	3	0.0009	0.8287	4,952.73	5	A0A7L8AB65,A0A7L8Z0C9,A0A7D7LL25,A0A7G3UUL1,A0A7L9W8W9_NDM	383,852	1_Control_Purified_Virions_1-18172-27118_27118	238	246
TRUE	0.0719	(R)FQTLLALHR(S)	97%	2.8920	0.8377		366.8854	1097.6344	3	-0.0003	-0.2547	4,933.91	5	A0A7L8AB65,A0A7L8Z0C9,A0A7D7LL25,A0A7G3UUL1,A0A7L9W8W9_NDM	621,494	1_Control_Purified_Virions_2-17652-26974_26974	238	246
TRUE	0.0786	(R)KSNLKPFER(D)	99%	2.0241	0.5636		373.5490	1117.6253	3	0.0008	0.7384	2,880.55	4	A0A7L8AB65,A0A7L8Z0C9,A0A7D7LL25,A0A7L9W8W9_NDM	1,734,420	1_Control_Purified_Virions_1-8460-14475_14475	458	466
TRUE	0.0786	(R)KSNLKPFER(D)	97%	2.6823	0.6230		373.5486	1117.6241	3	-0.0004	-0.3256	2,913.98	4	A0A7L8AB65,A0A7L8Z0C9,A0A7D7LL25,A0A7L9W8W9_NDM	1,222,680	1_Control_Purified_Virions_2-8202-14661_14661	458	466
TRUE	0.0786	(R)KSNLKPFER(D)	94%	1.4772	0.4827		373.5489	1117.6248	3	0.0004	0.3292	2,900.94	4	A0A7L8AB65,A0A7L8Z0C9,A0A7D7LL25,A0A7L9W8W9_NDM	852,608	1_Control_Purified_Virions_1-8548-14593_14593	458	466
TRUE	0.0601	(R)VVVLSFELLHAPATVcGPK(K)	99%	4.0406	0.7204	Carbamidomethyl (+57)	679.7117	2036.1134	3	0.0003	0.1612	6,357.62	3	A0A7L8Z0C9,A0A7D7LL25,A0A7L9W8W9_NDM	2,207,880	1_Control_Purified_Virions_1-26096-36375_36375	510	528
TRUE	0.0601	(R)VVVLSFELLHAPATVcGPK(K)	97%	4.2563	0.7988	Carbamidomethyl (+57)	679.7123	2036.1152	3	0.0022	1.0600	6,352.68	3	A0A7L8Z0C9,A0A7D7LL25,A0A7L9W8W9_NDM	1,301,320	1_Control_Purified_Virions_2-25588-36294_36294	510	528
Spike glycoprotein OS=Severe acute respiratory syndrome coronavirus 2 OX=2697049 GN=S PE=3 SV=1 Accession A0A7D7LL25																		
TRUE	0.1487	(R)GVYYPDKVFR(S)	99%	2.0205	0.7473		415.2208	1242.6405	3	0.0007	0.5574	4,530.86	4	A0A7L8AB65,A0A7L8Z0C9,A0A7G3UUL1,A0A7L9W8W9_NDM	1,168,410	1_Control_Purified_Virions_1-16007-24431_24431	35	44
TRUE	0.1487	(R)GVYYPDKVFR(S)	97%	2.0155	0.7700		415.2204	1242.6393	3	-0.0005	-0.3996	4,504.70	4	A0A7L8AB65,A0A7L8Z0C9,A0A7G3UUL1,A0A7L9W8W9_NDM	1,577,920	1_Control_Purified_Virions_2-15451-24244_24244	35	44
TRUE	0.1487	(R)GVYYPDKVFR(S)	97%	2.0658	0.6332		415.2206	1242.6401	3	0.0003	0.2629	4,483.81	4	A0A7L8AB65,A0A7L8Z0C9,A0A7G3UUL1,A0A7L9W8W9_NDM	707,501	1_Control_Purified_Virions_2-15337-24105_24105	35	44
TRUE	0.1539	(R)FQTLLALHR(S)	99%	2.3250	0.7606		366.8858	1097.6356	3	0.0009	0.8287	4,952.73	5	A0A7L8AB65,A0A7L8Z0C9,A0A7G3UUL1,A0A7L9W8W9_NDM,A0A7L9F8J2	383,852	1_Control_Purified_Virions_1-18172-27118_27118	238	246
TRUE	0.1539	(R)FQTLLALHR(S)	97%	2.8920	0.8377		366.8854	1097.6344	3	-0.0003	-0.2547	4,933.91	5	A0A7L8AB65,A0A7L8Z0C9,A0A7G3UUL1,A0A7L9W8W9_NDM,A0A7L9F8J2	621,494	1_Control_Purified_Virions_2-17652-26974_26974	238	246
TRUE	0.1683	(R)KSNLKPFER(D)	99%	2.0241	0.5636		373.5490	1117.6253	3	0.0008	0.7384	2,880.55	4	A0A7L8AB65,A0A7L8Z0C9,A0A7L9W8W9_NDM,A0A7L9F8J2	1,734,420	1_Control_Purified_Virions_1-8460-14475_14475	458	466
TRUE	0.1683	(R)KSNLKPFER(D)	97%	2.6823	0.6230		373.5486	1117.6241	3	-0.0004	-0.3256	2,913.98	4	A0A7L8AB65,A0A7L8Z0C9,A0A7L9W8W9_NDM,A0A7L9F8J2	1,222,680	1_Control_Purified_Virions_2-8202-14661_14661	458	466
TRUE	0.1683	(R)KSNLKPFER(D)	94%	1.4772	0.4827		373.5489	1117.6248	3	0.0004	0.3292	2,900.94	4	A0A7L8AB65,A0A7L8Z0C9,A0A7L9W8W9_NDM,A0A7L9F8J2	852,608	1_Control_Purified_Virions_1-8548-14593_14593	458	466
TRUE	0.1287	(R)VVVLSFELLHAPATVcGPK(K)	99%	4.0406	0.7204	Carbamidomethyl (+57)	679.7117	2036.1134	3	0.0003	0.1612	6,357.62	3	A0A7L8Z0C9,A0A7L9W8W9_NDM,A0A7L9F8J2	2,207,880	1_Control_Purified_Virions_1-26096-36375_36375	510	528
TRUE	0.1287	(R)VVVLSFELLHAPATVcGPK(K)	97%	4.2563	0.7988	Carbamidomethyl (+57)	679.7123	2036.1152	3	0.0022	1.0600	6,352.68	3	A0A7L8Z0C9,A0A7L9W8W9_NDM,A0A7L9F8J2	1,301,320	1_Control_Purified_Virions_2-25588-36294_36294	510	528
Spike glycoprotein OS=Severe acute respiratory syndrome coronavirus 2 OX=2697049 GN=S PE=3 SV=1 Accession A0A7D5XMN0																		
TRUE	0.3108	(K)LNDLcFTNVYADSFVIRGDEVR(Q)	99%	3.7817	0.8379	Carbamidomethyl (+57)	868.4216	2602.2431	3	-0.0057	-2.1790	6,394.46	2	A0A7L8Z0C9,A0A7L9W8W9_NDM	832,082	1_Control_Purified_Virions_1-26323-36625_36625	387	408
TRUE	0.3108	(K)LNDLcFTNVYADSFVIRGDEVR(Q)	97%	3.4239	0.8404	Carbamidomethyl (+57)	868.4241	2602.2506	3	0.0018	0.7050	6,390.85	2	A0A7L8Z0C9,A0A7L9W8W9_NDM	718,966	1_Control_Purified_Virions_2-25823-36553_36553	387	408
TRUE	0.3602	(R)ALTGIAVEQDKNTQEVFAQVK(Q)	99%	5.5613	0.7991		763.7410	2288.2013	3	-0.0002	-0.0943	5,435.09	2	A0A7L8Z0C9,A0A7L9W8W9_NDM	1,868,740	1_Control_Purified_Virions_1-20775-30245_30245	766	786
TRUE	0.3602	(R)ALTGIAVEQDKNTQEVFAQVK(Q)	97%	5.8025	0.7940		763.7410	2288.2011	3	-0.0004	-0.1742	5,424.23	2	A0A7L8Z0C9,A0A7L9W8W9_NDM	2,331,850	1_Control_Purified_Virions_2-20297-30148_30148	766	786
TRUE	0.3571	(R)SFIEDLLFNKVTLADAGFIK(Q)	97%	4.2669	0.8960		747.7430	2240.2073	3	-0.0023	-1.0140	6,720.77	2	A0A7L8Z0C9,A0A7L9W8W9_NDM	630,101	1_Control_Purified_Virions_2-27828-38792_38792	816	835
Spike glycoprotein OS=Severe acute respiratory syndrome coronavirus 2 OX=2697049 GN=S PE=3 SV=1 Accession A0A7D7XU89																		
TRUE	1.0000	(K)VcEFQFcNDPFLGVYYHENNKSWMESEFR(V)	97%	0.4088	0.9556	Carbamidomethyl (+57), Carbamidomethyl (+57)	747.3212	3731.5695	5	-0.0214	-5.7190	5,892.84	0		367,183	1_Control_Purified_Virions_1-23380-33277_33277	130	158
2_Mock_Purified_Virions_Mock_Treated																		
Surface glycoprotein OS=Severe acute respiratory syndrome coronavirus 2 OX=2697049 GN=S PE=4 SV=1 Accession A0A7L9W8W9_NDM [c]																		
TRUE	0.0067	(R)GVYYPDKVFR(S)	95%	2.0970	0.7826		415.2213	1242.6420	3	0.0023	1.8090	4,537.40	4	A0A7L8AB65,A0A7L8Z0C9,A0A7D7LL25,A0A7G3UUL1	882,949	2_Mock_Treated_Purified_Virions_1-15961-24386_24386	22	31
TRUE	0.0085	(K)SWMESEFRVYSSANNcTFEYVSQPFLMDLEGK(Q)	92%	0.2968	0.7966	Carbamidomethyl (+57)	771.3394	3851.6604	5	-0.0190	-4.9420	4,855.36	1	A0A6N1WN54	626,326	2_Mock_Treated_Purified_Virions_1-17610-26427_26427	138	169
TRUE	0.0070	(R)FQTLLALHR(S)	95%	2.7804	0.7483		366.8852	1097.6337	3	-0.0010	-0.9213	4,980.96	5	A0A7L8AB65,A0A7L8Z0C9,A0A7D7LL25,A0A7G3UUL1,A0A7L9F8J2	543,637	2_Mock_Treated_Purified_Virions_1-18280-27246_27246	225	233
TRUE	0.0076	(R)KSNLKPFER(D)	95%	1.7749	0.4661		373.5487	1117.6242	3	-0.0003	-0.2437	2,925.46	4	A0A7L8AB65,A0A7L8Z0C9,A0A7D7LL25,A0A7L9F8J2	810,585	2_Mock_Treated_Purified_Virions_1-8347-14531_14531	445	453
TRUE	0.0076	(R)KSNLKPFER(D)	91%	1.5428	0.3498		373.5488	1117.6246	3	0.0001	0.0836	2,904.27	4	A0A7L8AB65,A0A7L8Z0C9,A0A7D7LL25,A0A7L9F8J2	822,949	2_Mock_Treated_Purified_Virions_1-8251-14404_14404	445	453
TRUE	0.0058	(R)VVVLSFELLHAPATVcGPK(K)	95%	4.5843	0.7983	Carbamidomethyl (+57)	679.7111	2036.1113	3	-0.0017	-0.8276	6,379.73	3	A0A7L8Z0C9,A0A7D7LL25,A0A7L9F8J2	1,685,240	2_Mock_Treated_Purified_Virions_1-26258-36506_36506	497	515
TRUE	1.0000	(R)AGcLIGAEHVDNSYEcDIPIGAGIcASYQTQTNSPR(R)	96%	0.2959	0.7460	Carbamidomethyl (+57), Carbamidomethyl (+57), Carbamidomethyl (+57)	981.9421	3923.7392	4	-0.0125	-3.1750	5,413.04	0		232,276	2_Mock_Treated_Purified_Virions_1-20635-30058_30058	634	669
TRUE	0.0187	(R)ALTGIAVEQDKNTQEVFAQVK(Q)	95%	5.6070	0.7790		763.7410	2288.2013	3	-0.0002	-0.0943	5,463.06	2	A0A7L8Z0C9,A0A7D5XMN0	2,657,430	2_Mock_Treated_Purified_Virions_1-20939-30396_30396	753	773
TRUE	0.0185	(R)SFIEDLLFNKVTLADAGFIK(Q)	95%	3.9433	0.8233		747.7440	2240.2100	3	0.0005	0.2112	6,727.53	2	A0A7L8Z0C9,A0A7D5XMN0	339,030	2_Mock_Treated_Purified_Virions_1-28346-38852_38852	803	822
Surface glycoprotein OS=Severe acute respiratory syndrome coronavirus 2 OX=2697049 GN=S PE=4 SV=1 Accession A0A7L8Z0C9																		
TRUE	0.0666	(R)GVYYPDKVFR(S)	96%	2.2518	0.7712		415.2209	1242.6407	3	0.0010	0.7783	4,541.98	4	A0A7L8AB65,A0A7D7LL25,A0A7G3UUL1,A0A7L9W8W9_NDM	905,291	2_Mock_Treated_Purified_Virions_2-16174-24779_24779	35	44
TRUE	0.0666	(R)GVYYPDKVFR(S)	95%	2.0970	0.7826		415.2213	1242.6420	3	0.0023	1.8090	4,537.40	4	A0A7L8AB65,A0A7D7LL25,A0A7G3UUL1,A0A7L9W8W9_NDM	882,949	2_Mock_Treated_Purified_Virions_1-15961-24386_24386	35	44
TRUE	0.0699	(K)SWMESEFRVYSSANNcTFEYVSQPFLMDLEGKQGNFK(N)	92%	0.2624	0.9003	Carbamidomethyl (+57)	886.0045	4424.9862	5	0.0045	1.0090	5,112.68	1	A0A6N1WN54	49,997	2_Mock_Treated_Purified_Virions_2-19161-28460_28460	151	187
TRUE	0.0690	(R)FQTLLALHR(S)	96%	2.5584	0.8084		366.8853	1097.6341	3	-0.0006	-0.5047	4,992.44	5	A0A7L8AB65,A0A7D7LL25,A0A7G3UUL1,A0A7L9W8W9_NDM,A0A7L9F8J2	426,559	2_Mock_Treated_Purified_Virions_2-18517-27677_27677	238	246
TRUE	0.0690	(R)FQTLLALHR(S)	95%	2.7804	0.7483		366.8852	1097.6337	3	-0.0010	-0.9213	4,980.96	5	A0A7L8AB65,A0A7D7LL25,A0A7G3UUL1,A0A7L9W8W9_NDM,A0A7L9F8J2	543,637	2_Mock_Treated_Purified_Virions_1-18280-27246_27246	238	246
TRUE	0.1587	(K)RISNcVADYSVLYNSASFSTFK(C)	96%	0.9689	0.3438	Carbamidomethyl (+57)	843.7335	2528.1785	3	-0.0219	-8.6530	6,208.45	1	A0A7D5XMN0	387,566	2_Mock_Treated_Purified_Virions_2-25409-35709_35709	357	378
TRUE	0.0754	(R)KSNLKPFER(D)	96%	1.6281	0.5371		373.5484	1117.6235	3	-0.0010	-0.8985	2,932.95	4	A0A7L8AB65,A0A7D7LL25,A0A7L9W8W9_NDM,A0A7L9F8J2	714,936	2_Mock_Treated_Purified_Virions_2-8583-14931_14931	458	466
TRUE	0.0754	(R)KSNLKPFER(D)	96%	1.5488	0.4429		373.5487	1117.6242	3	-0.0003	-0.2437	2,912.56	4	A0A7L8AB65,A0A7D7LL25,A0A7L9W8W9_NDM,A0A7L9F8J2	651,705	2_Mock_Treated_Purified_Virions_2-8495-14812_14812	458	466
TRUE	0.0754	(R)KSNLKPFER(D)	95%	1.7749	0.4661		373.5487	1117.6242	3	-0.0003	-0.2437	2,925.46	4	A0A7L8AB65,A0A7D7LL25,A0A7L9W8W9_NDM,A0A7L9F8J2	810,585	2_Mock_Treated_Purified_Virions_1-8347-14531_14531	458	466
TRUE	0.0754	(R)KSNLKPFER(D)	91%	1.5428	0.3498		373.5488	1117.6246	3	0.0001	0.0836	2,904.27	4	A0A7L8AB65,A0A7D7LL25,A0A7L9W8W9_NDM,A0A7L9F8J2	822,949	2_Mock_Treated_Purified_Virions_1-8251-14404_14404	458	466
TRUE	0.0577	(R)VVVLSFELLHAPATVcGPK(K)	96%	4.5465	0.7370	Carbamidomethyl (+57)	679.7106	2036.1101	3	-0.0030	-1.4570	6,379.13	3	A0A7D7LL25,A0A7L9W8W9_NDM,A0A7L9F8J2	2,127,240	2_Mock_Treated_Purified_Virions_2-26422-36852_36852	510	528
TRUE	0.0577	(R)VVVLSFELLHAPATVcGPK(K)	95%	4.5843	0.7983	Carbamidomethyl (+57)	679.7111	2036.1113	3	-0.0017	-0.8276	6,379.73	3	A0A7D7LL25,A0A7L9W8W9_NDM,A0A7L9F8J2	1,685,240	2_Mock_Treated_Purified_Virions_1-26258-36506_36506	510	528
TRUE	0.1857	(R)ALTGIAVEQDKNTQEVFAQVK(Q)	96%	5.9330	0.7924		763.7412	2288.2018	3	0.0003	0.1457	5,477.57	2	A0A7D5XMN0,A0A7L9W8W9_NDM	2,081,330	2_Mock_Treated_Purified_Virions_2-21203-30853_30853	766	786
TRUE	0.1857	(R)ALTGIAVEQDKNTQEVFAQVK(Q)	95%	5.6070	0.7790		763.7410	2288.2013	3	-0.0002	-0.0943	5,463.06	2	A0A7D5XMN0,A0A7L9W8W9_NDM	2,657,430	2_Mock_Treated_Purified_Virions_1-20939-30396_30396	766	786
TRUE	0.1841	(R)SFIEDLLFNKVTLADAGFIK(Q)	95%	3.9433	0.8233		747.7440	2240.2100	3	0.0005	0.2112	6,727.53	2	A0A7D5XMN0,A0A7L9W8W9_NDM	339,030	2_Mock_Treated_Purified_Virions_1-28346-38852_38852	816	835
Surface glycoprotein OS=Severe acute respiratory syndrome coronavirus 2 OX=2697049 GN=S PE=4 SV=1 Accession A0A7L8AB65																		
TRUE	0.1513	(R)GVYYPDKVFR(S)	96%	2.2518	0.7712		415.2209	1242.6407	3	0.0010	0.7783	4,541.98	4	A0A7L8Z0C9,A0A7D7LL25,A0A7G3UUL1,A0A7L9W8W9_NDM	905,291	2_Mock_Treated_Purified_Virions_2-16174-24779_24779	35	44
TRUE	0.1513	(R)GVYYPDKVFR(S)	95%	2.0970	0.7826		415.2213	1242.6420	3	0.0023	1.8090	4,537.40	4	A0A7L8Z0C9,A0A7D7LL25,A0A7G3UUL1,A0A7L9W8W9_NDM	882,949	2_Mock_Treated_Purified_Virions_1-15961-24386_24386	35	44
TRUE	0.1567	(R)FQTLLALHR(S)	96%	2.5584	0.8084		366.8853	1097.6341	3	-0.0006	-0.5047	4,992.44	5	A0A7L8Z0C9,A0A7D7LL25,A0A7G3UUL1,A0A7L9W8W9_NDM,A0A7L9F8J2	426,559	2_Mock_Treated_Purified_Virions_2-18517-27677_27677	238	246
TRUE	0.1567	(R)FQTLLALHR(S)	95%	2.7804	0.7483		366.8852	1097.6337	3	-0.0010	-0.9213	4,980.96	5	A0A7L8Z0C9,A0A7D7LL25,A0A7G3UUL1,A0A7L9W8W9_NDM,A0A7L9F8J2	543,637	2_Mock_Treated_Purified_Virions_1-18280-27246_27246	238	246
TRUE	0.1713	(R)KSNLKPFER(D)	96%	1.6281	0.5371		373.5484	1117.6235	3	-0.0010	-0.8985	2,932.95	4	A0A7L8Z0C9,A0A7D7LL25,A0A7L9W8W9_NDM,A0A7L9F8J2	714,936	2_Mock_Treated_Purified_Virions_2-8583-14931_14931	458	466
TRUE	0.1713	(R)KSNLKPFER(D)	96%	1.5488	0.4429		373.5487	1117.6242	3	-0.0003	-0.2437	2,912.56	4	A0A7L8Z0C9,A0A7D7LL25,A0A7L9W8W9_NDM,A0A7L9F8J2	651,705	2_Mock_Treated_Purified_Virions_2-8495-14812_14812	458	466
TRUE	0.1713	(R)KSNLKPFER(D)	95%	1.7749	0.4661		373.5487	1117.6242	3	-0.0003	-0.2437	2,925.46	4	A0A7L8Z0C9,A0A7D7LL25,A0A7L9W8W9_NDM,A0A7L9F8J2	810,585	2_Mock_Treated_Purified_Virions_1-8347-14531_14531	458	466
TRUE	0.1713	(R)KSNLKPFER(D)	91%	1.5428	0.3498		373.5488	1117.6246	3	0.0001	0.0836	2,904.27	4	A0A7L8Z0C9,A0A7D7LL25,A0A7L9W8W9_NDM,A0A7L9F8J2	822,949	2_Mock_Treated_Purified_Virions_1-8251-14404_14404	458	466
Spike glycoprotein OS=Severe acute respiratory syndrome coronavirus 2 OX=2697049 GN=S PE=3 SV=1 Accession A0A7G3UUL1																		
TRUE	0.1630	(R)GVYYPDKVFR(S)	96%	2.2518	0.7712		415.2209	1242.6407	3	0.0010	0.7783	4,541.98	4	A0A7L8AB65,A0A7L8Z0C9,A0A7D7LL25,A0A7L9W8W9_NDM	905,291	2_Mock_Treated_Purified_Virions_2-16174-24779_24779	35	44
TRUE	0.1630	(R)GVYYPDKVFR(S)	95%	2.0970	0.7826		415.2213	1242.6420	3	0.0023	1.8090	4,537.40	4	A0A7L8AB65,A0A7L8Z0C9,A0A7D7LL25,A0A7L9W8W9_NDM	882,949	2_Mock_Treated_Purified_Virions_1-15961-24386_24386	35	44
TRUE	0.1688	(R)FQTLLALHR(S)	96%	2.5584	0.8084		366.8853	1097.6341	3	-0.0006	-0.5047	4,992.44	5	A0A7L8AB65,A0A7L8Z0C9,A0A7D7LL25,A0A7L9W8W9_NDM,A0A7L9F8J2	426,559	2_Mock_Treated_Purified_Virions_2-18517-27677_27677	238	246
TRUE	0.1688	(R)FQTLLALHR(S)	95%	2.7804	0.7483		366.8852	1097.6337	3	-0.0010	-0.9213	4,980.96	5	A0A7L8AB65,A0A7L8Z0C9,A0A7D7LL25,A0A7L9W8W9_NDM,A0A7L9F8J2	543,637	2_Mock_Treated_Purified_Virions_1-18280-27246_27246	238	246
Surface glycoprotein OS=Severe acute respiratory syndrome coronavirus 2 OX=2697049 GN=S PE=4 SV=1 Accession A0A7L9F8J2																		
TRUE	0.0719	(R)FQTLLALHR(S)	96%	2.5584	0.8084		366.8853	1097.6341	3	-0.0006	-0.5047	4,992.44	5	A0A7L8AB65,A0A7L8Z0C9,A0A7D7LL25,A0A7G3UUL1,A0A7L9W8W9_NDM	426,559	2_Mock_Treated_Purified_Virions_2-18517-27677_27677	238	246
TRUE	0.0719	(R)FQTLLALHR(S)	95%	2.7804	0.7483		366.8852	1097.6337	3	-0.0010	-0.9213	4,980.96	5	A0A7L8AB65,A0A7L8Z0C9,A0A7D7LL25,A0A7G3UUL1,A0A7L9W8W9_NDM	543,637	2_Mock_Treated_Purified_Virions_1-18280-27246_27246	238	246
TRUE	0.0786	(R)KSNLKPFER(D)	96%	1.6281	0.5371		373.5484	1117.6235	3	-0.0010	-0.8985	2,932.95	4	A0A7L8AB65,A0A7L8Z0C9,A0A7D7LL25,A0A7L9W8W9_NDM	714,936	2_Mock_Treated_Purified_Virions_2-8583-14931_14931	458	466
TRUE	0.0786	(R)KSNLKPFER(D)	96%	1.5488	0.4429		373.5487	1117.6242	3	-0.0003	-0.2437	2,912.56	4	A0A7L8AB65,A0A7L8Z0C9,A0A7D7LL25,A0A7L9W8W9_NDM	651,705	2_Mock_Treated_Purified_Virions_2-8495-14812_14812	458	466
TRUE	0.0786	(R)KSNLKPFER(D)	95%	1.7749	0.4661		373.5487	1117.6242	3	-0.0003	-0.2437	2,925.46	4	A0A7L8AB65,A0A7L8Z0C9,A0A7D7LL25,A0A7L9W8W9_NDM	810,585	2_Mock_Treated_Purified_Virions_1-8347-14531_14531	458	466
TRUE	0.0786	(R)KSNLKPFER(D)	91%	1.5428	0.3498		373.5488	1117.6246	3	0.0001	0.0836	2,904.27	4	A0A7L8AB65,A0A7L8Z0C9,A0A7D7LL25,A0A7L9W8W9_NDM	822,949	2_Mock_Treated_Purified_Virions_1-8251-14404_14404	458	466
TRUE	0.0601	(R)VVVLSFELLHAPATVcGPK(K)	96%	4.5465	0.7370	Carbamidomethyl (+57)	679.7106	2036.1101	3	-0.0030	-1.4570	6,379.13	3	A0A7L8Z0C9,A0A7D7LL25,A0A7L9W8W9_NDM	2,127,240	2_Mock_Treated_Purified_Virions_2-26422-36852_36852	510	528
TRUE	0.0601	(R)VVVLSFELLHAPATVcGPK(K)	95%	4.5843	0.7983	Carbamidomethyl (+57)	679.7111	2036.1113	3	-0.0017	-0.8276	6,379.73	3	A0A7L8Z0C9,A0A7D7LL25,A0A7L9W8W9_NDM	1,685,240	2_Mock_Treated_Purified_Virions_1-26258-36506_36506	510	528
TRUE	1.0000	(K)KFPPFQQFGR(D)	96%	1.1072	0.2985		417.8926	1250.6561	3	-0.0001	-0.0653	2,930.21	0		886,858	2_Mock_Treated_Purified_Virions_2-8570-14914_14914	558	567
Spike glycoprotein OS=Severe acute respiratory syndrome coronavirus 2 OX=2697049 GN=S PE=3 SV=1 Accession A0A7D7LL25																		
TRUE	0.1487	(R)GVYYPDKVFR(S)	96%	2.2518	0.7712		415.2209	1242.6407	3	0.0010	0.7783	4,541.98	4	A0A7L8AB65,A0A7L8Z0C9,A0A7G3UUL1,A0A7L9W8W9_NDM	905,291	2_Mock_Treated_Purified_Virions_2-16174-24779_24779	35	44
TRUE	0.1487	(R)GVYYPDKVFR(S)	95%	2.0970	0.7826		415.2213	1242.6420	3	0.0023	1.8090	4,537.40	4	A0A7L8AB65,A0A7L8Z0C9,A0A7G3UUL1,A0A7L9W8W9_NDM	882,949	2_Mock_Treated_Purified_Virions_1-15961-24386_24386	35	44
TRUE	0.1539	(R)FQTLLALHR(S)	96%	2.5584	0.8084		366.8853	1097.6341	3	-0.0006	-0.5047	4,992.44	5	A0A7L8AB65,A0A7L8Z0C9,A0A7G3UUL1,A0A7L9W8W9_NDM,A0A7L9F8J2	426,559	2_Mock_Treated_Purified_Virions_2-18517-27677_27677	238	246
TRUE	0.1539	(R)FQTLLALHR(S)	95%	2.7804	0.7483		366.8852	1097.6337	3	-0.0010	-0.9213	4,980.96	5	A0A7L8AB65,A0A7L8Z0C9,A0A7G3UUL1,A0A7L9W8W9_NDM,A0A7L9F8J2	543,637	2_Mock_Treated_Purified_Virions_1-18280-27246_27246	238	246
TRUE	0.1683	(R)KSNLKPFER(D)	96%	1.6281	0.5371		373.5484	1117.6235	3	-0.0010	-0.8985	2,932.95	4	A0A7L8AB65,A0A7L8Z0C9,A0A7L9W8W9_NDM,A0A7L9F8J2	714,936	2_Mock_Treated_Purified_Virions_2-8583-14931_14931	458	466
TRUE	0.1683	(R)KSNLKPFER(D)	96%	1.5488	0.4429		373.5487	1117.6242	3	-0.0003	-0.2437	2,912.56	4	A0A7L8AB65,A0A7L8Z0C9,A0A7L9W8W9_NDM,A0A7L9F8J2	651,705	2_Mock_Treated_Purified_Virions_2-8495-14812_14812	458	466
TRUE	0.1683	(R)KSNLKPFER(D)	95%	1.7749	0.4661		373.5487	1117.6242	3	-0.0003	-0.2437	2,925.46	4	A0A7L8AB65,A0A7L8Z0C9,A0A7L9W8W9_NDM,A0A7L9F8J2	810,585	2_Mock_Treated_Purified_Virions_1-8347-14531_14531	458	466
TRUE	0.1683	(R)KSNLKPFER(D)	91%	1.5428	0.3498		373.5488	1117.6246	3	0.0001	0.0836	2,904.27	4	A0A7L8AB65,A0A7L8Z0C9,A0A7L9W8W9_NDM,A0A7L9F8J2	822,949	2_Mock_Treated_Purified_Virions_1-8251-14404_14404	458	466
TRUE	0.1287	(R)VVVLSFELLHAPATVcGPK(K)	96%	4.5465	0.7370	Carbamidomethyl (+57)	679.7106	2036.1101	3	-0.0030	-1.4570	6,379.13	3	A0A7L8Z0C9,A0A7L9W8W9_NDM,A0A7L9F8J2	2,127,240	2_Mock_Treated_Purified_Virions_2-26422-36852_36852	510	528
TRUE	0.1287	(R)VVVLSFELLHAPATVcGPK(K)	95%	4.5843	0.7983	Carbamidomethyl (+57)	679.7111	2036.1113	3	-0.0017	-0.8276	6,379.73	3	A0A7L8Z0C9,A0A7L9W8W9_NDM,A0A7L9F8J2	1,685,240	2_Mock_Treated_Purified_Virions_1-26258-36506_36506	510	528
Spike glycoprotein OS=Severe acute respiratory syndrome coronavirus 2 OX=2697049 GN=S PE=3 SV=1 Accession A0A7D5XMN0																		
TRUE	1.0000	(K)NNKSWmESEFRVYSSANNcTFEYVSQPFFmDLEGK(Q)	91%	0.5813	0.6522	Oxidation (+16), Carbamidomethyl (+57), Oxidation (+16)	855.5721	4272.8243	5	-0.0259	-6.0670	5,075.99	0		610,234	2_Mock_Treated_Purified_Virions_2-18985-28233_28233	148	182
TRUE	0.3079	(K)RISNcVADYSVLYNSASFSTFK(C)	96%	0.9689	0.3438	Carbamidomethyl (+57)	843.7335	2528.1785	3	-0.0219	-8.6530	6,208.45	1	A0A7L8Z0C9	387,566	2_Mock_Treated_Purified_Virions_2-25409-35709_35709	357	378
TRUE	0.3602	(R)ALTGIAVEQDKNTQEVFAQVK(Q)	96%	5.9330	0.7924		763.7412	2288.2018	3	0.0003	0.1457	5,477.57	2	A0A7L8Z0C9,A0A7L9W8W9_NDM	2,081,330	2_Mock_Treated_Purified_Virions_2-21203-30853_30853	766	786
TRUE	0.3602	(R)ALTGIAVEQDKNTQEVFAQVK(Q)	95%	5.6070	0.7790		763.7410	2288.2013	3	-0.0002	-0.0943	5,463.06	2	A0A7L8Z0C9,A0A7L9W8W9_NDM	2,657,430	2_Mock_Treated_Purified_Virions_1-20939-30396_30396	766	786
TRUE	0.3571	(R)SFIEDLLFNKVTLADAGFIK(Q)	95%	3.9433	0.8233		747.7440	2240.2100	3	0.0005	0.2112	6,727.53	2	A0A7L8Z0C9,A0A7L9W8W9_NDM	339,030	2_Mock_Treated_Purified_Virions_1-28346-38852_38852	816	835
Spike glycoprotein OS=Severe acute respiratory syndrome coronavirus 2 OX=2697049 GN=S PE=3 SV=1 Accessioon A0A6N1WN54																		
TRUE	1.0000	(-)MFVSLVLLPLVSSQcVNLTTR(T)	96%	0.9480	0.3243	Carbamidomethyl (+57)	793.1000	2376.2781	3	-0.0129	-5.4420	5,969.68	0		794,756	2_Mock_Treated_Purified_Virions_2-24015-34118_34118	1	21
TRUE	0.0729	(K)SWMESEFRVYSSANNcTFEYVSQPFLMDLEGKQGNFK(N)	96%	0.2624	0.9003	Carbamidomethyl (+57)	886.0045	4424.9862	5	0.0045	1.0090	5,112.68	1	A0A7L8Z0C9	49,997	2_Mock_Treated_Purified_Virions_2-19161-28460_28460	151	187
Spike glycoprotein OS=Severe acute respiratory syndrome coronavirus 2 OX=2697049 GN=S PE=3 SV=1 Accession A0A7D7XU89																		
TRUE	1.0000	(K)VcEFQFcNDPFLGVYYHENNKSWMESEFR(V)	96%	0.2476	0.9576	Carbamidomethyl (+57), Carbamidomethyl (+57)	747.3232	3731.5795	5	-0.0113	-3.0210	5,930.00	0		457,936	2_Mock_Treated_Purified_Virions_1-23604-33500_33500	130	158
3_PNGase-F_Treated_Purified_Virions																		
Surface glycoprotein OS=Severe acute respiratory syndrome coronavirus 2 OX=2697049 GN=S PE=4 SV=1 Accession A0A7L9W8W9_NDM [c]																		
TRUE	0.0143	(K)RFDNPVLPFNDGVYFASTEK(S)	100%	2.6473	0.6536		772.7159	2315.1258	3	0.0034	1.4870	6,263.08	2	A0A7L8Z0C9,A0A7D5XMN0	8,030,750	3_PNGase-F_Treated_Purified_Virions_2-27217-36852_36852	65	84
TRUE	0.0071	(R)GWIFGTTLDSK(T)	97%	2.5328	0.8053		612.8166	1223.6186	2	-0.0002	-0.1992	6,011.15	4	A0A7L8AB65,A0A7L8Z0C9,A0A7D7LL25,A0A7G3UUL1	346,633	3_PNGase-F_Treated_Purified_Virions_2-25715-35163_35163	90	100
TRUE	0.0071	(R)GWIFGTTLDSK(T)	97%	2.4684	0.8142		612.8170	1223.6194	2	0.0005	0.3989	5,989.61	4	A0A7L8AB65,A0A7L8Z0C9,A0A7D7LL25,A0A7G3UUL1	358,879	3_PNGase-F_Treated_Purified_Virions_2-25582-35016_35016	90	100
TRUE	0.0071	(R)GWIFGTTLDSK(T)	96%	2.7065	0.8265		612.8171	1223.6196	2	0.0007	0.5983	6,006.27	4	A0A7L8AB65,A0A7L8Z0C9,A0A7D7LL25,A0A7G3UUL1	523,138	3_PNGase-F_Treated_Purified_Virions_1-24137-34277_34277	90	100
TRUE	0.0192	(K)VcEFQFcNDPFLGVYYHK(N)	100%	4.6695	0.8798	Carbamidomethyl (+57), Carbamidomethyl (+57)	775.0156	2322.0250	3	0.0011	0.4929	6,351.23	2	A0A7L8Z0C9,A0A7D5XMN0	1,460,760	3_PNGase-F_Treated_Purified_Virions_2-27724-37438_37438	117	134
TRUE	0.0192	(K)VcEFQFcNDPFLGVYYHK(N)	96%	4.9430	0.8719	Carbamidomethyl (+57), Carbamidomethyl (+57)	775.0164	2322.0272	3	0.0033	1.4390	6,351.11	2	A0A7L8Z0C9,A0A7D5XMN0	1,963,620	3_PNGase-F_Treated_Purified_Virions_1-26152-36573_36573	117	134
TRUE	0.0069	(K)SWMESEFR(V)	97%	1.6456	0.9016		536.2317	1070.4488	2	-0.0002	-0.2275	4,952.82	4	A0A7L8AB65,A0A7L8Z0C9,A0A7D7LL25,A0A7G3UUL1	375,500	3_PNGase-F_Treated_Purified_Virions_2-19601-28112_28112	138	145
TRUE	0.0069	(K)SWMESEFR(V)	96%	1.8237	0.8855		536.2322	1070.4498	2	0.0007	0.6840	4,984.73	4	A0A7L8AB65,A0A7L8Z0C9,A0A7D7LL25,A0A7G3UUL1	511,569	3_PNGase-F_Treated_Purified_Virions_1-18520-27618_27618	138	145
TRUE	0.0089	(K)NIDGYFK(I)	90%	1.6610	0.6689		428.7141	855.4136	2	0.0008	0.9796	4,236.03	3	A0A7L8AB65,A0A7L8Z0C9,A0A7L9F8J2	1,065,270	3_PNGase-F_Treated_Purified_Virions_2-15582-23376_23376	183	189
TRUE	0.0089	(K)NIDGYFK(I)	90%	1.5320	0.6494		428.7140	855.4134	2	0.0007	0.7658	4,265.37	3	A0A7L8AB65,A0A7L8Z0C9,A0A7L9F8J2	801,284	3_PNGase-F_Treated_Purified_Virions_1-14714-22977_22977	183	189
TRUE	0.0103	(K)HTPINLVR(D)	96%	1.5640	0.7021		475.2830	948.5514	2	0.0009	0.9019	3,563.24	3	A0A7L8AB65,A0A7L8Z0C9,A0A7L9F8J2	833,341	3_PNGase-F_Treated_Purified_Virions_2-12093-19080_19080	194	201
TRUE	0.0103	(K)HTPINLVR(D)	96%	1.8884	0.7449		475.2831	948.5516	2	0.0010	1.0950	3,594.57	3	A0A7L8AB65,A0A7L8Z0C9,A0A7L9F8J2	869,729	3_PNGase-F_Treated_Purified_Virions_1-11510-18855_18855	194	201
TRUE	1.0000	(R)DLPQGFSALEPLVDLPIGIDITR(F)	94%	5.2058	0.3290		1240.1747	2478.3348	2	-0.0028	-1.1120	6,749.62	0		1,444,260	3_PNGase-F_Treated_Purified_Virions_2-30114-40118_40118	202	224
TRUE	1.0000	(R)DLPQGFSALEPLVDLPIGIDITR(F)	91%	3.4776	0.2746		827.1200	2478.3382	3	0.0006	0.2364	6,750.22	0		488,681	3_PNGase-F_Treated_Purified_Virions_1-28568-39269_39269	202	224
TRUE	0.0070	(R)FQTLLALHR(S)	100%	2.5350	0.7561		366.8854	1097.6344	3	-0.0003	-0.2547	4,960.98	5	A0A7L8AB65,A0A7L8Z0C9,A0A7D7LL25,A0A7G3UUL1,A0A7L9F8J2	435,797	3_PNGase-F_Treated_Purified_Virions_2-19651-28167_28167	225	233
TRUE	0.0070	(R)FQTLLALHR(S)	96%	2.2677	0.6768		366.8856	1097.6348	3	0.0002	0.1620	4,994.33	5	A0A7L8AB65,A0A7L8Z0C9,A0A7D7LL25,A0A7G3UUL1,A0A7L9F8J2	423,990	3_PNGase-F_Treated_Purified_Virions_1-18576-27682_27682	225	233
TRUE	1.0000	(K)YNEDGTITDAVDcALDPLSETK(C)	97%	4.2618	0.8775	Carbamidomethyl (+57)	1214.0477	2426.0809	2	0.0010	0.4001	6,378.96	0		741,945	3_PNGase-F_Treated_Purified_Virions_2-27894-37625_37625	266	287
TRUE	1.0000	(K)YNEDGTITDAVDcALDPLSETK(C)	96%	4.8463	0.8894	Carbamidomethyl (+57)	1214.0502	2426.0858	2	0.0059	2.4120	6,378.45	0		1,101,920	3_PNGase-F_Treated_Purified_Virions_1-26319-36757_36757	266	287
TRUE	0.0065	(K)GIYQTSNFR(V)	97%	1.8943	0.7794		543.2726	1084.5307	2	0.0006	0.5209	3,814.02	4	A0A7L8Z0C9,A0A7D7LL25,A0A7G3UUL1,A0A7L9F8J2	626,867	3_PNGase-F_Treated_Purified_Virions_2-13337-20648_20648	298	306
TRUE	0.0065	(K)GIYQTSNFR(V)	96%	2.0406	0.8229		543.2713	1084.5280	2	-0.0021	-1.9530	3,844.11	4	A0A7L8Z0C9,A0A7D7LL25,A0A7G3UUL1,A0A7L9F8J2	657,407	3_PNGase-F_Treated_Purified_Virions_1-12603-20324_20324	298	306
TRUE	0.0065	(R)VQPTESIVR(F)	97%	2.1545	0.5921		514.7906	1027.5667	2	0.0005	0.4630	3,410.59	4	A0A7L8Z0C9,A0A7D7LL25,A0A7G3UUL1,A0A7L9F8J2	661,845	3_PNGase-F_Treated_Purified_Virions_2-11276-18092_18092	307	315
TRUE	0.0065	(R)VQPTESIVR(F)	96%	1.9877	0.5545		514.7907	1027.5669	2	0.0006	0.5816	3,440.76	4	A0A7L8Z0C9,A0A7D7LL25,A0A7G3UUL1,A0A7L9F8J2	780,276	3_PNGase-F_Treated_Purified_Virions_1-10733-17882_17882	307	315
TRUE	0.0065	(R)VQPTESIVR(F)	95%	1.7273	0.5011		514.7907	1027.5669	2	0.0006	0.5816	3,389.70	4	A0A7L8Z0C9,A0A7D7LL25,A0A7G3UUL1,A0A7L9F8J2	365,253	3_PNGase-F_Treated_Purified_Virions_2-11167-17958_17958	307	315
TRUE	0.0065	(R)VQPTESIVR(F)	94%	1.5213	0.4637		514.7908	1027.5670	2	0.0007	0.7003	3,419.76	4	A0A7L8Z0C9,A0A7D7LL25,A0A7G3UUL1,A0A7L9F8J2	161,472	3_PNGase-F_Treated_Purified_Virions_1-10633-17751_17751	307	315
TRUE	1.0000	(R)FPDITNLcPFGEVFDATR(F)	97%	5.2669	0.8652	Carbamidomethyl (+57)	1049.9979	2097.9813	2	-0.0019	-0.9235	6,632.94	0		1,795,360	3_PNGase-F_Treated_Purified_Virions_2-29400-39326_39326	316	333
TRUE	1.0000	(R)FPDITNLcPFGEVFDATR(F)	96%	5.1096	0.8664	Carbamidomethyl (+57)	1049.9973	2097.9801	2	-0.0032	-1.5050	6,631.25	0		1,339,390	3_PNGase-F_Treated_Purified_Virions_2-27840-38462_38462	316	333
TRUE	0.0088	(R)FASVYAWNR(K)	97%	2.3151	0.7888		557.2776	1112.5406	2	0.0005	0.4069	4,822.20	2	A0A7L8Z0C9,A0A7G3UUL1	1,047,410	3_PNGase-F_Treated_Purified_Virions_2-18859-27242_27242	334	342
TRUE	0.0088	(R)FASVYAWNR(K)	96%	2.1253	0.7141		557.2780	1112.5415	2	0.0013	1.1740	4,851.89	2	A0A7L8Z0C9,A0A7G3UUL1	716,410	3_PNGase-F_Treated_Purified_Virions_1-17828-26767_26767	334	342
TRUE	0.0087	(K)cYGVSPTK(L)	96%	2.1334	0.7404	Carbamidomethyl (+57)	456.2185	910.4225	2	0.0006	0.6417	2,759.24	2	A0A7L8Z0C9,A0A7G3UUL1	257,199	3_PNGase-F_Treated_Purified_Virions_1-7740-13842_13842	366	373
TRUE	0.0087	(K)cYGVSPTK(L)	96%	1.6549	0.6837	Carbamidomethyl (+57)	456.2182	910.4218	2	-0.0001	-0.1619	2,716.87	2	A0A7L8Z0C9,A0A7G3UUL1	131,418	3_PNGase-F_Treated_Purified_Virions_2-7972-13846_13846	366	373
TRUE	0.0089	(K)LNDLcFTNVYADSFVIR(G)	97%	3.7152	0.7610	Carbamidomethyl (+57)	1024.0061	2045.9977	2	0.0095	4.6500	6,488.96	3	A0A7L8Z0C9,A0A7D7LL25,A0A7L9F8J2	908,975	3_PNGase-F_Treated_Purified_Virions_2-28556-38361_38361	374	390
TRUE	0.0089	(K)LNDLcFTNVYADSFVIR(G)	96%	4.0813	0.8234	Carbamidomethyl (+57)	1024.0062	2045.9979	2	0.0098	4.7700	6,487.75	3	A0A7L8Z0C9,A0A7D7LL25,A0A7L9F8J2	886,567	3_PNGase-F_Treated_Purified_Virions_1-26981-37492_37492	374	390
TRUE	0.0067	(K)IADYNYK(L)	90%	2.0170	0.7673		443.7220	885.4294	2	0.0061	6.8660	3,088.53	4	A0A7L8AB65,A0A7L8Z0C9,A0A7D7LL25,A0A7G3UUL1	1,292,480	3_PNGase-F_Treated_Purified_Virions_1-9243-15825_15825	405	411
TRUE	0.0067	(K)IADYNYK(L)	90%	1.7636	0.7470		443.7206	885.4267	2	0.0034	3.8360	3,062.68	4	A0A7L8AB65,A0A7L8Z0C9,A0A7D7LL25,A0A7G3UUL1	4,307,390	3_PNGase-F_Treated_Purified_Virions_2-9631-15966_15966	405	411
TRUE	0.0143	(K)LPDDFTGcVIAWNSNNLDSK(V)	97%	5.2705	0.9424	Carbamidomethyl (+57)	1133.5253	2265.0360	2	-0.0015	-0.6402	6,188.35	2	A0A7L8Z0C9,A0A7D5XMN0	579,815	3_PNGase-F_Treated_Purified_Virions_2-26759-36346_36346	412	431
TRUE	0.0069	(K)VGGNYNYLYR(L)	97%	3.4868	0.7657		609.7985	1217.5825	2	-0.0004	-0.3092	4,337.90	4	A0A7L8AB65,A0A7L8Z0C9,A0A7D7LL25,A0A7L9F8J2	1,383,380	3_PNGase-F_Treated_Purified_Virions_2-16111-24024_24024	432	441
TRUE	0.0069	(K)VGGNYNYLYR(L)	96%	3.3406	0.7891		609.7991	1217.5837	2	0.0008	0.6926	4,369.26	4	A0A7L8AB65,A0A7L8Z0C9,A0A7D7LL25,A0A7L9F8J2	1,363,820	3_PNGase-F_Treated_Purified_Virions_1-15222-23620_23620	432	441
TRUE	0.0076	(R)KSNLKPFER(D)	100%	2.1141	0.6261		373.5487	1117.6244	3	-0.0001	-0.0801	2,884.70	4	A0A7L8AB65,A0A7L8Z0C9,A0A7D7LL25,A0A7L9F8J2	1,289,000	3_PNGase-F_Treated_Purified_Virions_2-8780-14875_14875	445	453
TRUE	0.0076	(R)KSNLKPFER(D)	96%	1.6289	0.5833		373.5487	1117.6243	3	-0.0002	-0.1619	2,916.20	4	A0A7L8AB65,A0A7L8Z0C9,A0A7D7LL25,A0A7L9F8J2	739,797	3_PNGase-F_Treated_Purified_Virions_1-8449-14777_14777	445	453
TRUE	0.0076	(R)KSNLKPFER(D)	93%	1.5707	0.4993		373.5486	1117.6240	3	-0.0005	-0.4074	2,905.27	4	A0A7L8AB65,A0A7L8Z0C9,A0A7D7LL25,A0A7L9F8J2	1,013,180	3_PNGase-F_Treated_Purified_Virions_2-8883-15004_15004	445	453
TRUE	0.0058	(R)VVVLSFELLHAPATVcGPK(K)	100%	4.9151	0.7457	Carbamidomethyl (+57)	679.7120	2036.1141	3	0.0011	0.5207	6,370.21	3	A0A7L8Z0C9,A0A7D7LL25,A0A7L9F8J2	1,900,120	3_PNGase-F_Treated_Purified_Virions_2-27840-37566_37566	497	515
TRUE	0.0058	(R)VVVLSFELLHAPATVcGPK(K)	96%	3.4543	0.7949	Carbamidomethyl (+57)	679.7123	2036.1152	3	0.0022	1.0600	6,368.36	3	A0A7L8Z0C9,A0A7D7LL25,A0A7L9F8J2	649,757	3_PNGase-F_Treated_Purified_Virions_1-26257-36689_36689	497	515
TRUE	0.0076	(K)FLPFQQFGR(D)	97%	1.7527	0.8042		570.3038	1138.5930	2	0.0005	0.4575	5,858.06	3	A0A7L8AB65,A0A7L8Z0C9,A0A7G3UUL1	1,321,460	3_PNGase-F_Treated_Purified_Virions_2-24826-34144_34144	546	554
TRUE	0.0076	(K)FLPFQQFGR(D)	96%	1.7690	0.8141		570.3038	1138.5930	2	0.0005	0.4575	5,881.52	3	A0A7L8AB65,A0A7L8Z0C9,A0A7G3UUL1	1,018,840	3_PNGase-F_Treated_Purified_Virions_1-23437-33461_33461	546	554
TRUE	0.0076	(K)FLPFQQFGR(D)	96%	1.7673	0.7981		570.3040	1138.5934	2	0.0009	0.7788	5,861.09	3	A0A7L8AB65,A0A7L8Z0C9,A0A7G3UUL1	823,990	3_PNGase-F_Treated_Purified_Virions_1-23326-33328_33328	546	554
TRUE	0.0073	(R)DIADTTDAVR(D)	97%	1.3291	0.7683		538.7649	1075.5152	2	0.0005	0.4296	3,347.32	3	A0A7L8AB65,A0A7L8Z0C9,A0A7G3UUL1	873,387	3_PNGase-F_Treated_Purified_Virions_2-10953-17690_17690	555	564
TRUE	0.0069	(R)VYSTGSNVFQTR(A)	97%	3.3441	0.8418		679.8397	1357.6649	2	0.0023	1.7080	4,117.61	4	A0A7L8AB65,A0A7L8Z0C9,A0A7G3UUL1,A0A7L9F8J2	848,752	3_PNGase-F_Treated_Purified_Virions_2-14966-22618_22618	622	633
TRUE	0.0187	(R)ALTGIAVEQDKNTQEVFAQVK(Q)	96%	4.1841	0.7620		763.7409	2288.2009	3	-0.0006	-0.2542	5,456.09	2	A0A7L8Z0C9,A0A7D5XMN0	266,173	3_PNGase-F_Treated_Purified_Virions_1-21022-30653_30653	753	773
TRUE	0.0265	(K)TPPIKDFGGFDFSQILPDPSKPSK(R)	95%	2.8270	0.8015		655.3449	2617.3505	4	0.0072	2.7360	6,440.38	1	A0A7D5XMN0	585,524	3_PNGase-F_Treated_Purified_Virions_2-28259-38034_38034	778	801
TRUE	0.0073	(R)SFIEDLLFNK(V)	97%	2.8636	0.7353		613.3271	1224.6396	2	0.0005	0.3689	6,581.63	4	A0A7L8AB65,A0A7L8Z0C9,A0A7G3UUL1,A0A7L9F8J2	1,798,220	3_PNGase-F_Treated_Purified_Virions_2-29084-38976_38976	803	812
TRUE	0.0073	(R)SFIEDLLFNK(V)	96%	2.7287	0.6910		613.3268	1224.6391	2	0.0000	-0.0295	6,581.03	4	A0A7L8AB65,A0A7L8Z0C9,A0A7G3UUL1,A0A7L9F8J2	1,370,570	3_PNGase-F_Treated_Purified_Virions_1-27530-38119_38119	803	812
TRUE	0.0067	(K)QYGDcLGDIAAR(D)	97%	2.9417	0.8188	Carbamidomethyl (+57)	669.8084	1337.6023	2	-0.0014	-1.0210	4,436.26	5	A0A7L8AB65,A0A7L8Z0C9,A0A7D7LL25,A0A7G3UUL1,A0A7L9F8J2	1,260,660	3_PNGase-F_Treated_Purified_Virions_2-16634-24662_24662	823	834
TRUE	0.0067	(K)QYGDcLGDIAAR(D)	96%	3.1760	0.8291	Carbamidomethyl (+57)	669.8084	1337.6023	2	-0.0014	-1.0210	4,467.28	5	A0A7L8AB65,A0A7L8Z0C9,A0A7D7LL25,A0A7G3UUL1,A0A7L9F8J2	1,224,550	3_PNGase-F_Treated_Purified_Virions_1-15683-24220_24220	823	834
TRUE	0.0074	(K)LIANQFNSAIGK(I)	96%	2.6862	0.7259		638.3564	1274.6983	2	0.0000	-0.0251	4,629.21	4	A0A7L8AB65,A0A7L8Z0C9,A0A7G3UUL1,A0A7L9F8J2	301,302	3_PNGase-F_Treated_Purified_Virions_1-16591-25293_25293	909	920
TRUE	0.0074	(K)LIANQFNSAIGK(I)	95%	1.8770	0.5454		638.3569	1274.6992	2	0.0008	0.6447	4,590.89	4	A0A7L8AB65,A0A7L8Z0C9,A0A7G3UUL1,A0A7L9F8J2	520,886	3_PNGase-F_Treated_Purified_Virions_2-17529-25700_25700	909	920
TRUE	0.0093	(K)IQDSLSSTASALGK(L)	96%	3.8149	0.8918		689.3645	1376.7145	2	-0.0004	-0.3039	4,433.10	3	A0A7L8AB65,A0A7L8Z0C9,A0A7L9F8J2	357,144	3_PNGase-F_Treated_Purified_Virions_1-15491-23994_23994	921	934
TRUE	0.0075	(K)LQDVVNQNAQALNTLVK(Q)	97%	2.7123	0.8093		934.5232	1867.0318	2	0.0154	8.2250	5,457.69	3	A0A7L8Z0C9,A0A7D7LL25,A0A7L9F8J2	1,055,200	3_PNGase-F_Treated_Purified_Virions_2-22494-31465_31465	935	951
TRUE	0.0075	(K)LQDVVNQNAQALNTLVK(Q)	96%	3.7087	0.8221		934.5233	1867.0320	2	0.0155	8.2900	5,483.31	3	A0A7L8Z0C9,A0A7D7LL25,A0A7L9F8J2	775,126	3_PNGase-F_Treated_Purified_Virions_1-21180-30835_30835	935	951
TRUE	0.0063	(R)LDKVEAEVQIDR(L)	100%	2.6277	0.5532		472.2569	1413.7488	3	0.0022	1.5550	4,037.08	4	A0A7L8AB65,A0A7L8Z0C9,A0A7D7LL25,A0A7G3UUL1	355,296	3_PNGase-F_Treated_Purified_Virions_2-14497-22074_22074	971	982
TRUE	0.0073	(R)LQSLQTYVTQQLIR(A)	97%	3.5916	0.8139		845.9792	1689.9439	2	0.0024	1.4040	6,236.56	3	A0A7L8AB65,A0A7L8Z0C9,A0A7G3UUL1	2,320,250	3_PNGase-F_Treated_Purified_Virions_2-27054-36672_36672	988	1001
TRUE	0.0073	(R)LQSLQTYVTQQLIR(A)	96%	3.7699	0.7898		845.9786	1689.9427	2	0.0012	0.6817	6,235.21	3	A0A7L8AB65,A0A7L8Z0C9,A0A7G3UUL1	1,881,990	3_PNGase-F_Treated_Purified_Virions_2-25481-35803_35803	988	1001
TRUE	0.0077	(R)ASANLAATK(M)	96%	2.7680	0.6600		423.7376	845.4607	2	0.0000	-0.0034	2,267.86	3	A0A7L8AB65,A0A7L8Z0C9,A0A7G3UUL1	598,948	3_PNGase-F_Treated_Purified_Virions_1-5767-11060_11060	1007	1015
TRUE	0.0077	(R)ASANLAATK(M)	96%	2.8312	0.6200		423.7377	845.4609	2	0.0002	0.2129	2,270.84	3	A0A7L8AB65,A0A7L8Z0C9,A0A7G3UUL1	606,657	3_PNGase-F_Treated_Purified_Virions_1-5779-11077_11077	1007	1015
TRUE	0.0079	(K)RVDFcGK(G)	90%	2.3953	0.6864	Carbamidomethyl (+57)	441.2178	880.4211	2	-0.0016	-1.7930	2,632.00	3	A0A7L8AB65,A0A7L8Z0C9,A0A7G3UUL1	718,518	3_PNGase-F_Treated_Purified_Virions_1-7233-13127_13127	1026	1032
TRUE	0.0079	(K)RVDFcGK(G)	90%	2.2308	0.6454	Carbamidomethyl (+57)	441.2180	880.4215	2	-0.0011	-1.2390	2,577.62	3	A0A7L8AB65,A0A7L8Z0C9,A0A7G3UUL1	319,475	3_PNGase-F_Treated_Purified_Virions_2-7380-13042_13042	1026	1032
TRUE	0.0149	(K)DHTSPDVDLGDISGIDASVVNIQK(E)	96%	5.0499	0.8717		832.4140	2494.2202	3	0.0010	0.4028	6,116.36	1	A0A7D5XMN0	343,736	3_PNGase-F_Treated_Purified_Virions_1-24759-35000_35000	1145	1168
Surface glycoprotein OS=Severe acute respiratory syndrome coronavirus 2 OX=2697049 GN=S PE=4 SV=1 Accession A0A7L8Z0C9																		
TRUE	0.1415	(K)RFDNPVLPFNDGVYFASTEK(S)	100%	2.6473	0.6536		772.7159	2315.1258	3	0.0034	1.4870	6,263.08	2	A0A7D5XMN0,A0A7L9W8W9_NDM	8,030,750	3_PNGase-F_Treated_Purified_Virions_2-27217-36852_36852	78	97
TRUE	0.0708	(R)GWIFGTTLDSK(T)	97%	2.5328	0.8053		612.8166	1223.6186	2	-0.0002	-0.1992	6,011.15	4	A0A7L8AB65,A0A7D7LL25,A0A7G3UUL1,A0A7L9W8W9_NDM	346,633	3_PNGase-F_Treated_Purified_Virions_2-25715-35163_35163	103	113
TRUE	0.0708	(R)GWIFGTTLDSK(T)	97%	2.4684	0.8142		612.8170	1223.6194	2	0.0005	0.3989	5,989.61	4	A0A7L8AB65,A0A7D7LL25,A0A7G3UUL1,A0A7L9W8W9_NDM	358,879	3_PNGase-F_Treated_Purified_Virions_2-25582-35016_35016	103	113
TRUE	0.0708	(R)GWIFGTTLDSK(T)	96%	2.7065	0.8265		612.8171	1223.6196	2	0.0007	0.5983	6,006.27	4	A0A7L8AB65,A0A7D7LL25,A0A7G3UUL1,A0A7L9W8W9_NDM	523,138	3_PNGase-F_Treated_Purified_Virions_1-24137-34277_34277	103	113
TRUE	0.1908	(K)VcEFQFcNDPFLGVYYHK(N)	100%	4.6695	0.8798	Carbamidomethyl (+57), Carbamidomethyl (+57)	775.0156	2322.0250	3	0.0011	0.4929	6,351.23	2	A0A7D5XMN0,A0A7L9W8W9_NDM	1,460,760	3_PNGase-F_Treated_Purified_Virions_2-27724-37438_37438	130	147
TRUE	0.1908	(K)VcEFQFcNDPFLGVYYHK(N)	96%	4.9430	0.8719	Carbamidomethyl (+57), Carbamidomethyl (+57)	775.0164	2322.0272	3	0.0033	1.4390	6,351.11	2	A0A7D5XMN0,A0A7L9W8W9_NDM	1,963,620	3_PNGase-F_Treated_Purified_Virions_1-26152-36573_36573	130	147
TRUE	0.0689	(K)SWMESEFR(V)	97%	1.6456	0.9016		536.2317	1070.4488	2	-0.0002	-0.2275	4,952.82	4	A0A7L8AB65,A0A7D7LL25,A0A7G3UUL1,A0A7L9W8W9_NDM	375,500	3_PNGase-F_Treated_Purified_Virions_2-19601-28112_28112	151	158
TRUE	0.0689	(K)SWMESEFR(V)	96%	1.8237	0.8855		536.2322	1070.4498	2	0.0007	0.6840	4,984.73	4	A0A7L8AB65,A0A7D7LL25,A0A7G3UUL1,A0A7L9W8W9_NDM	511,569	3_PNGase-F_Treated_Purified_Virions_1-18520-27618_27618	151	158
TRUE	0.0882	(K)NIDGYFK(I)	90%	1.6610	0.6689		428.7141	855.4136	2	0.0008	0.9796	4,236.03	3	A0A7L8AB65,A0A7L9W8W9_NDM,A0A7L9F8J2	1,065,270	3_PNGase-F_Treated_Purified_Virions_2-15582-23376_23376	196	202
TRUE	0.0882	(K)NIDGYFK(I)	90%	1.5320	0.6494		428.7140	855.4134	2	0.0007	0.7658	4,265.37	3	A0A7L8AB65,A0A7L9W8W9_NDM,A0A7L9F8J2	801,284	3_PNGase-F_Treated_Purified_Virions_1-14714-22977_22977	196	202
TRUE	0.1024	(K)HTPINLVR(D)	96%	1.5640	0.7021		475.2830	948.5514	2	0.0009	0.9019	3,563.24	3	A0A7L8AB65,A0A7L9W8W9_NDM,A0A7L9F8J2	833,341	3_PNGase-F_Treated_Purified_Virions_2-12093-19080_19080	207	214
TRUE	0.1024	(K)HTPINLVR(D)	96%	1.8884	0.7449		475.2831	948.5516	2	0.0010	1.0950	3,594.57	3	A0A7L8AB65,A0A7L9W8W9_NDM,A0A7L9F8J2	869,729	3_PNGase-F_Treated_Purified_Virions_1-11510-18855_18855	207	214
TRUE	0.0690	(R)FQTLLALHR(S)	100%	2.5350	0.7561		366.8854	1097.6344	3	-0.0003	-0.2547	4,960.98	5	A0A7L8AB65,A0A7D7LL25,A0A7G3UUL1,A0A7L9W8W9_NDM,A0A7L9F8J2	435,797	3_PNGase-F_Treated_Purified_Virions_2-19651-28167_28167	238	246
TRUE	0.0690	(R)FQTLLALHR(S)	96%	2.2677	0.6768		366.8856	1097.6348	3	0.0002	0.1620	4,994.33	5	A0A7L8AB65,A0A7D7LL25,A0A7G3UUL1,A0A7L9W8W9_NDM,A0A7L9F8J2	423,990	3_PNGase-F_Treated_Purified_Virions_1-18576-27682_27682	238	246
TRUE	0.0646	(K)GIYQTSNFR(V)	97%	1.8943	0.7794		543.2726	1084.5307	2	0.0006	0.5209	3,814.02	4	A0A7D7LL25,A0A7G3UUL1,A0A7L9W8W9_NDM,A0A7L9F8J2	626,867	3_PNGase-F_Treated_Purified_Virions_2-13337-20648_20648	311	319
TRUE	0.0646	(K)GIYQTSNFR(V)	96%	2.0406	0.8229		543.2713	1084.5280	2	-0.0021	-1.9530	3,844.11	4	A0A7D7LL25,A0A7G3UUL1,A0A7L9W8W9_NDM,A0A7L9F8J2	657,407	3_PNGase-F_Treated_Purified_Virions_1-12603-20324_20324	311	319
TRUE	0.0648	(R)VQPTESIVR(F)	97%	2.1545	0.5921		514.7906	1027.5667	2	0.0005	0.4630	3,410.59	4	A0A7D7LL25,A0A7G3UUL1,A0A7L9W8W9_NDM,A0A7L9F8J2	661,845	3_PNGase-F_Treated_Purified_Virions_2-11276-18092_18092	320	328
TRUE	0.0648	(R)VQPTESIVR(F)	96%	1.9877	0.5545		514.7907	1027.5669	2	0.0006	0.5816	3,440.76	4	A0A7D7LL25,A0A7G3UUL1,A0A7L9W8W9_NDM,A0A7L9F8J2	780,276	3_PNGase-F_Treated_Purified_Virions_1-10733-17882_17882	320	328
TRUE	0.0648	(R)VQPTESIVR(F)	95%	1.7273	0.5011		514.7907	1027.5669	2	0.0006	0.5816	3,389.70	4	A0A7D7LL25,A0A7G3UUL1,A0A7L9W8W9_NDM,A0A7L9F8J2	365,253	3_PNGase-F_Treated_Purified_Virions_2-11167-17958_17958	320	328
TRUE	0.0648	(R)VQPTESIVR(F)	94%	1.5213	0.4637		514.7908	1027.5670	2	0.0007	0.7003	3,419.76	4	A0A7D7LL25,A0A7G3UUL1,A0A7L9W8W9_NDM,A0A7L9F8J2	161,472	3_PNGase-F_Treated_Purified_Virions_1-10633-17751_17751	320	328
TRUE	0.0000	(R)FPNITNLcPFGEVFNATRFASVYAWNR(K)	94%	1.8684	0.4382	Carbamidomethyl (+57)	798.6391	3190.5273	4	-0.0173	-5.4350	6,735.03	0		1,425,270	3_PNGase-F_Treated_Purified_Virions_1-28475-39167_39167	329	355
TRUE	0.0873	(R)FASVYAWNR(K)	97%	2.3151	0.7888		557.2776	1112.5406	2	0.0005	0.4069	4,822.20	2	A0A7G3UUL1,A0A7L9W8W9_NDM	1,047,410	3_PNGase-F_Treated_Purified_Virions_2-18859-27242_27242	347	355
TRUE	0.0873	(R)FASVYAWNR(K)	96%	2.1253	0.7141		557.2780	1112.5415	2	0.0013	1.1740	4,851.89	2	A0A7G3UUL1,A0A7L9W8W9_NDM	716,410	3_PNGase-F_Treated_Purified_Virions_1-17828-26767_26767	347	355
TRUE	0.0868	(K)cYGVSPTK(L)	96%	2.1334	0.7404	Carbamidomethyl (+57)	456.2185	910.4225	2	0.0006	0.6417	2,759.24	2	A0A7G3UUL1,A0A7L9W8W9_NDM	257,199	3_PNGase-F_Treated_Purified_Virions_1-7740-13842_13842	379	386
TRUE	0.0868	(K)cYGVSPTK(L)	96%	1.6549	0.6837	Carbamidomethyl (+57)	456.2182	910.4218	2	-0.0001	-0.1619	2,716.87	2	A0A7G3UUL1,A0A7L9W8W9_NDM	131,418	3_PNGase-F_Treated_Purified_Virions_2-7972-13846_13846	379	386
TRUE	0.0882	(K)LNDLcFTNVYADSFVIR(G)	97%	3.7152	0.7610	Carbamidomethyl (+57)	1024.0061	2045.9977	2	0.0095	4.6500	6,488.96	3	A0A7D7LL25,A0A7L9W8W9_NDM,A0A7L9F8J2	908,975	3_PNGase-F_Treated_Purified_Virions_2-28556-38361_38361	387	403

Identified Spike Peptides

Valid	Weight	Sequence a,b	Protein identification probability (Prob)	SEQUEST XCorr	SEQUEST deltaCn	Modifications	Observed Mass (Da)	Actual Mass (Da)	Charge	Delta Da	Delta PPM	Retention Time	# Other Proteins	Other Proteins	Total Ion Chromatogram (TIC)	Spectrum ID	Peptide Start Index	Peptide Stop Index
TRUE	0.0882	(K)LNDLcFTNVYADSFVIR(G)	96%	4.0813	0.8234	Carbamidomethyl (+57)	1024.0062	2045.9979	2	0.0098	4.7700	6,487.75	3	A0A7D7LL25,A0A7L9W8W9_NDM,A0A7L9F8J2	886,567	3_PNGase-F_Treated_Purified_Virions_1-26981-37492_37492	387	403
TRUE	0.0664	(K)IADYNYK(L)	90%	2.0170	0.7673		443.7220	885.4294	2	0.0061	6.8660	3,088.53	4	A0A7L8AB65,A0A7D7LL25,A0A7G3UUL1,A0A7L9W8W9_NDM	1,292,480	3_PNGase-F_Treated_Purified_Virions_1-9243-15825_15825	418	424
TRUE	0.0664	(K)IADYNYK(L)	90%	1.7636	0.7470		443.7206	885.4267	2	0.0034	3.8360	3,062.68	4	A0A7L8AB65,A0A7D7LL25,A0A7G3UUL1,A0A7L9W8W9_NDM	4,307,390	3_PNGase-F_Treated_Purified_Virions_2-9631-15966_15966	418	424
TRUE	0.1415	(K)LPDDFTGcVIAWNSNNLDSK(V)	97%	5.2705	0.9424	Carbamidomethyl (+57)	1133.5253	2265.0360	2	-0.0015	-0.6402	6,188.35	2	A0A7D5XMN0,A0A7L9W8W9_NDM	579,815	3_PNGase-F_Treated_Purified_Virions_2-26759-36346_36346	425	444
TRUE	0.0684	(K)VGGNYNYLYR(L)	97%	3.4868	0.7657		609.7985	1217.5825	2	-0.0004	-0.3092	4,337.90	4	A0A7L8AB65,A0A7D7LL25,A0A7L9W8W9_NDM,A0A7L9F8J2	1,383,380	3_PNGase-F_Treated_Purified_Virions_2-16111-24024_24024	445	454
TRUE	0.0684	(K)VGGNYNYLYR(L)	96%	3.3406	0.7891		609.7991	1217.5837	2	0.0008	0.6926	4,369.26	4	A0A7L8AB65,A0A7D7LL25,A0A7L9W8W9_NDM,A0A7L9F8J2	1,363,820	3_PNGase-F_Treated_Purified_Virions_1-15222-23620_23620	445	454
TRUE	0.0754	(R)KSNLKPFER(D)	100%	2.1141	0.6261		373.5487	1117.6244	3	-0.0001	-0.0801	2,884.70	4	A0A7L8AB65,A0A7D7LL25,A0A7L9W8W9_NDM,A0A7L9F8J2	1,289,000	3_PNGase-F_Treated_Purified_Virions_2-8780-14875_14875	458	466
TRUE	0.0754	(R)KSNLKPFER(D)	96%	1.6289	0.5833		373.5487	1117.6243	3	-0.0002	-0.1619	2,916.20	4	A0A7L8AB65,A0A7D7LL25,A0A7L9W8W9_NDM,A0A7L9F8J2	739,797	3_PNGase-F_Treated_Purified_Virions_1-8449-14777_14777	458	466
TRUE	0.0754	(R)KSNLKPFER(D)	93%	1.5707	0.4993		373.5486	1117.6240	3	-0.0005	-0.4074	2,905.27	4	A0A7L8AB65,A0A7D7LL25,A0A7L9W8W9_NDM,A0A7L9F8J2	1,013,180	3_PNGase-F_Treated_Purified_Virions_2-8883-15004_15004	458	466
TRUE	0.0577	(R)VVVLSFELLHAPATVcGPK(K)	100%	4.9151	0.7457	Carbamidomethyl (+57)	679.7120	2036.1141	3	0.0011	0.5207	6,370.21	3	A0A7D7LL25,A0A7L9W8W9_NDM,A0A7L9F8J2	1,900,120	3_PNGase-F_Treated_Purified_Virions_2-27840-37566_37566	510	528
TRUE	0.0577	(R)VVVLSFELLHAPATVcGPK(K)	96%	3.4543	0.7949	Carbamidomethyl (+57)	679.7123	2036.1152	3	0.0022	1.0600	6,368.36	3	A0A7D7LL25,A0A7L9W8W9_NDM,A0A7L9F8J2	649,757	3_PNGase-F_Treated_Purified_Virions_1-26257-36689_36689	510	528
TRUE	0.0755	(K)FLPFQQFGR(D)	97%	1.7527	0.8042		570.3038	1138.5930	2	0.0005	0.4575	5,858.06	3	A0A7L8AB65,A0A7G3UUL1,A0A7L9W8W9_NDM	1,321,460	3_PNGase-F_Treated_Purified_Virions_2-24826-34144_34144	559	567
TRUE	0.0755	(K)FLPFQQFGR(D)	96%	1.7690	0.8141		570.3038	1138.5930	2	0.0005	0.4575	5,881.52	3	A0A7L8AB65,A0A7G3UUL1,A0A7L9W8W9_NDM	1,018,840	3_PNGase-F_Treated_Purified_Virions_1-23437-33461_33461	559	567
TRUE	0.0755	(K)FLPFQQFGR(D)	96%	1.7673	0.7981		570.3040	1138.5934	2	0.0009	0.7788	5,861.09	3	A0A7L8AB65,A0A7G3UUL1,A0A7L9W8W9_NDM	823,990	3_PNGase-F_Treated_Purified_Virions_1-23326-33328_33328	559	567
TRUE	0.0729	(R)DIADTTDAVR(D)	97%	1.3291	0.7683		538.7649	1075.5152	2	0.0005	0.4296	3,347.32	3	A0A7L8AB65,A0A7G3UUL1,A0A7L9W8W9_NDM	873,387	3_PNGase-F_Treated_Purified_Virions_2-10953-17690_17690	568	577
TRUE	0.0686	(R)VYSTGSNVFQTR(A)	97%	3.3441	0.8418		679.8397	1357.6649	2	0.0023	1.7080	4,117.61	4	A0A7L8AB65,A0A7G3UUL1,A0A7L9W8W9_NDM,A0A7L9F8J2	848,752	3_PNGase-F_Treated_Purified_Virions_2-14966-22618_22618	635	646
TRUE	0.1857	(R)ALTGIAVEQDKNTQEVFAQVK(Q)	96%	4.1841	0.7620		763.7409	2288.2009	3	-0.0006	-0.2542	5,456.09	2	A0A7D5XMN0,A0A7L9W8W9_NDM	266,173	3_PNGase-F_Treated_Purified_Virions_1-21022-30653_30653	766	786
TRUE	0.0723	(R)SFIEDLLFNK(V)	97%	2.8636	0.7353		613.3271	1224.6396	2	0.0005	0.3689	6,581.63	4	A0A7L8AB65,A0A7G3UUL1,A0A7L9W8W9_NDM,A0A7L9F8J2	1,798,220	3_PNGase-F_Treated_Purified_Virions_2-29084-38976_38976	816	825
TRUE	0.0723	(R)SFIEDLLFNK(V)	96%	2.7287	0.6910		613.3268	1224.6391	2	0.0000	-0.0295	6,581.03	4	A0A7L8AB65,A0A7G3UUL1,A0A7L9W8W9_NDM,A0A7L9F8J2	1,370,570	3_PNGase-F_Treated_Purified_Virions_1-27530-38119_38119	816	825
TRUE	0.0664	(K)QYGDcLGDIAAR(D)	97%	2.9417	0.8188	Carbamidomethyl (+57)	669.8084	1337.6023	2	-0.0014	-1.0210	4,436.26	5	A0A7L8AB65,A0A7D7LL25,A0A7G3UUL1,A0A7L9W8W9_NDM,A0A7L9F8J2	1,260,660	3_PNGase-F_Treated_Purified_Virions_2-16634-24662_24662	836	847
TRUE	0.0664	(K)QYGDcLGDIAAR(D)	96%	3.1760	0.8291	Carbamidomethyl (+57)	669.8084	1337.6023	2	-0.0014	-1.0210	4,467.28	5	A0A7L8AB65,A0A7D7LL25,A0A7G3UUL1,A0A7L9W8W9_NDM,A0A7L9F8J2	1,224,550	3_PNGase-F_Treated_Purified_Virions_1-15683-24220_24220	836	847
TRUE	0.0736	(K)LIANQFNSAIGK(I)	96%	2.6862	0.7259		638.3564	1274.6983	2	0.0000	-0.0251	4,629.21	4	A0A7L8AB65,A0A7G3UUL1,A0A7L9W8W9_NDM,A0A7L9F8J2	301,302	3_PNGase-F_Treated_Purified_Virions_1-16591-25293_25293	922	933
TRUE	0.0736	(K)LIANQFNSAIGK(I)	95%	1.8770	0.5454		638.3569	1274.6992	2	0.0008	0.6447	4,590.89	4	A0A7L8AB65,A0A7G3UUL1,A0A7L9W8W9_NDM,A0A7L9F8J2	520,886	3_PNGase-F_Treated_Purified_Virions_2-17529-25700_25700	922	933
TRUE	0.0921	(K)IQDSLSSTASALGK(L)	96%	3.8149	0.8918		689.3645	1376.7145	2	-0.0004	-0.3039	4,433.10	3	A0A7L8AB65,A0A7L9W8W9_NDM,A0A7L9F8J2	357,144	3_PNGase-F_Treated_Purified_Virions_1-15491-23994_23994	934	947
TRUE	0.0748	(K)LQDVVNQNAQALNTLVK(Q)	97%	2.7123	0.8093		934.5232	1867.0318	2	0.0154	8.2250	5,457.69	3	A0A7D7LL25,A0A7L9W8W9_NDM,A0A7L9F8J2	1,055,200	3_PNGase-F_Treated_Purified_Virions_2-22494-31465_31465	948	964
TRUE	0.0748	(K)LQDVVNQNAQALNTLVK(Q)	96%	3.7087	0.8221		934.5233	1867.0320	2	0.0155	8.2900	5,483.31	3	A0A7D7LL25,A0A7L9W8W9_NDM,A0A7L9F8J2	775,126	3_PNGase-F_Treated_Purified_Virions_1-21180-30835_30835	948	964
TRUE	0.0626	(R)LDKVEAEVQIDR(L)	100%	2.6277	0.5532		472.2569	1413.7488	3	0.0022	1.5550	4,037.08	4	A0A7L8AB65,A0A7D7LL25,A0A7G3UUL1,A0A7L9W8W9_NDM	355,296	3_PNGase-F_Treated_Purified_Virions_2-14497-22074_22074	984	995
TRUE	0.0728	(R)LQSLQTYVTQQLIR(A)	97%	3.5916	0.8139		845.9792	1689.9439	2	0.0024	1.4040	6,236.56	3	A0A7L8AB65,A0A7G3UUL1,A0A7L9W8W9_NDM	2,320,250	3_PNGase-F_Treated_Purified_Virions_2-27054-36672_36672	1001	1014
TRUE	0.0728	(R)LQSLQTYVTQQLIR(A)	96%	3.7699	0.7898		845.9786	1689.9427	2	0.0012	0.6817	6,235.21	3	A0A7L8AB65,A0A7G3UUL1,A0A7L9W8W9_NDM	1,881,990	3_PNGase-F_Treated_Purified_Virions_1-25481-35803_35803	1001	1014
TRUE	0.0767	(R)ASANLAATK(M)	96%	2.7680	0.6600		423.7376	845.4607	2	0.0000	-0.0034	2,267.86	3	A0A7L8AB65,A0A7G3UUL1,A0A7L9W8W9_NDM	598,948	3_PNGase-F_Treated_Purified_Virions_1-5767-11060_11060	1020	1028
TRUE	0.0767	(R)ASANLAATK(M)	96%	2.8312	0.6200		423.7377	845.4609	2	0.0002	0.2129	2,270.84	3	A0A7L8AB65,A0A7G3UUL1,A0A7L9W8W9_NDM	606,657	3_PNGase-F_Treated_Purified_Virions_1-5779-11077_11077	1020	1028
TRUE	0.0787	(K)RVDFcGK(G)	90%	2.3953	0.6864	Carbamidomethyl (+57)	441.2178	880.4211	2	-0.0016	-1.7930	2,632.00	3	A0A7L8AB65,A0A7G3UUL1,A0A7L9W8W9_NDM	718,518	3_PNGase-F_Treated_Purified_Virions_1-7233-13127_13127	1039	1045
TRUE	0.0787	(K)RVDFcGK(G)	90%	2.2308	0.6454	Carbamidomethyl (+57)	441.2180	880.4215	2	-0.0011	-1.2390	2,577.62	3	A0A7L8AB65,A0A7G3UUL1,A0A7L9W8W9_NDM	319,475	3_PNGase-F_Treated_Purified_Virions_2-7380-13042_13042	1039	1045
TRUE	0.0784	(R)EGVFVSNGTHWFVTQR(N)	96%	3.2124	0.6778		621.9753	1862.9040	3	-0.0024	-1.2650	5,825.46	2	A0A7D7LL25,A0A7L9F8J2	331,928	3_PNGase-F_Treated_Purified_Virions_1-23141-33100_33100	1092	1107
**Surface glycoprotein OS=Severe acute respiratory syndrome coronavirus 2 OX=2697049 GN=S PE=4 SV=1 Accession A0A7L8AB65**																		
TRUE	0.1608	(R)GWIFGTTLDSK(T)	97%	2.5328	0.8053		612.8166	1223.6186	2	-0.0002	-0.1992	6,011.15	4	A0A7L8Z0C9,A0A7D7LL25,A0A7G3UUL1,A0A7L9W8W9_NDM	346,633	3_PNGase-F_Treated_Purified_Virions_2-25715-35163_35163	103	113
TRUE	0.1608	(R)GWIFGTTLDSK(T)	97%	2.4684	0.8142		612.8170	1223.6194	2	0.0005	0.3989	5,989.61	4	A0A7L8Z0C9,A0A7D7LL25,A0A7G3UUL1,A0A7L9W8W9_NDM	358,879	3_PNGase-F_Treated_Purified_Virions_2-25582-35016_35016	103	113
TRUE	0.1608	(R)GWIFGTTLDSK(T)	96%	2.7065	0.8265		612.8171	1223.6196	2	0.0007	0.5983	6,006.27	4	A0A7L8Z0C9,A0A7D7LL25,A0A7G3UUL1,A0A7L9W8W9_NDM	523,138	3_PNGase-F_Treated_Purified_Virions_1-24137-34277_34277	103	113
TRUE	0.1565	(K)SWMESEFR(V)	97%	1.6456	0.9016		536.2317	1070.4488	2	-0.0002	-0.2275	4,952.82	4	A0A7L8Z0C9,A0A7D7LL25,A0A7G3UUL1,A0A7L9W8W9_NDM	375,500	3_PNGase-F_Treated_Purified_Virions_2-19601-28112_28112	151	158
TRUE	0.1565	(K)SWMESEFR(V)	96%	1.8237	0.8855		536.2322	1070.4498	2	0.0007	0.6840	4,984.73	4	A0A7L8Z0C9,A0A7D7LL25,A0A7G3UUL1,A0A7L9W8W9_NDM	511,569	3_PNGase-F_Treated_Purified_Virions_1-18520-27618_27618	151	158
TRUE	0.2003	(K)NIDGYFK(I)	90%	1.6610	0.6689		428.7141	855.4136	2	0.0008	0.9796	4,236.03	3	A0A7L8Z0C9,A0A7L9W8W9_NDM,A0A7L9F8J2	1,065,270	3_PNGase-F_Treated_Purified_Virions_2-15582-23376_23376	196	202
TRUE	0.2003	(K)NIDGYFK(I)	90%	1.5320	0.6494		428.7140	855.4134	2	0.0007	0.7658	4,265.37	3	A0A7L8Z0C9,A0A7L9W8W9_NDM,A0A7L9F8J2	801,284	3_PNGase-F_Treated_Purified_Virions_1-14714-22977_22977	196	202
TRUE	0.2325	(K)HTPINLVR(D)	96%	1.5640	0.7021		475.2830	948.5514	2	0.0009	0.9019	3,563.24	3	A0A7L8Z0C9,A0A7L9W8W9_NDM,A0A7L9F8J2	833,341	3_PNGase-F_Treated_Purified_Virions_2-12093-19080_19080	207	214
TRUE	0.2325	(K)HTPINLVR(D)	96%	1.8884	0.7449		475.2831	948.5516	2	0.0010	1.0950	3,594.57	3	A0A7L8Z0C9,A0A7L9W8W9_NDM,A0A7L9F8J2	869,729	3_PNGase-F_Treated_Purified_Virions_1-11510-18855_18855	207	214
TRUE	0.1567	(R)FQTLLALHR(S)	100%	2.5350	0.7561		366.8854	1097.6344	3	-0.0003	-0.2547	4,960.98	5	A0A7L8Z0C9,A0A7D7LL25,A0A7G3UUL1,A0A7L9W8W9_NDM,A0A7L9F8J2	435,797	3_PNGase-F_Treated_Purified_Virions_2-19651-28167_28167	238	246
TRUE	0.1567	(R)FQTLLALHR(S)	96%	2.2677	0.6768		366.8856	1097.6348	3	0.0002	0.1620	4,994.33	5	A0A7L8Z0C9,A0A7D7LL25,A0A7G3UUL1,A0A7L9W8W9_NDM,A0A7L9F8J2	423,990	3_PNGase-F_Treated_Purified_Virions_1-18576-27682_27682	238	246
TRUE	0.1507	(K)IADYNYK(L)	90%	2.0170	0.7673		443.7220	885.4294	2	0.0061	6.8660	3,088.53	4	A0A7L8Z0C9,A0A7D7LL25,A0A7G3UUL1,A0A7L9W8W9_NDM	1,292,480	3_PNGase-F_Treated_Purified_Virions_1-9243-15825_15825	418	424
TRUE	0.1507	(K)IADYNYK(L)	90%	1.7636	0.7470		443.7206	885.4267	2	0.0034	3.8360	3,062.68	4	A0A7L8Z0C9,A0A7D7LL25,A0A7G3UUL1,A0A7L9W8W9_NDM	4,307,390	3_PNGase-F_Treated_Purified_Virions_2-9631-15966_15966	418	424
TRUE	0.1552	(K)VGGNYNYLYR(L)	97%	3.4868	0.7657		609.7985	1217.5825	2	-0.0004	-0.3092	4,337.90	4	A0A7L8Z0C9,A0A7D7LL25,A0A7L9W8W9_NDM,A0A7L9F8J2	1,383,380	3_PNGase-F_Treated_Purified_Virions_2-16111-24024_24024	445	454
TRUE	0.1552	(K)VGGNYNYLYR(L)	96%	3.3406	0.7891		609.7991	1217.5837	2	0.0008	0.6926	4,369.26	4	A0A7L8Z0C9,A0A7D7LL25,A0A7L9W8W9_NDM,A0A7L9F8J2	1,363,820	3_PNGase-F_Treated_Purified_Virions_1-15222-23620_23620	445	454
TRUE	0.1713	(R)KSNLKPFER(D)	100%	2.1141	0.6261		373.5487	1117.6244	3	-0.0001	-0.0801	2,884.70	4	A0A7L8Z0C9,A0A7D7LL25,A0A7L9W8W9_NDM,A0A7L9F8J2	1,289,000	3_PNGase-F_Treated_Purified_Virions_2-8780-14875_14875	458	466
TRUE	0.1713	(R)KSNLKPFER(D)	96%	1.6289	0.5833		373.5487	1117.6243	3	-0.0002	-0.1619	2,916.20	4	A0A7L8Z0C9,A0A7D7LL25,A0A7L9W8W9_NDM,A0A7L9F8J2	739,797	3_PNGase-F_Treated_Purified_Virions_1-8449-14777_14777	458	466
TRUE	0.1713	(R)KSNLKPFER(D)	93%	1.5707	0.4993		373.5486	1117.6240	3	-0.0005	-0.4074	2,905.27	4	A0A7L8Z0C9,A0A7D7LL25,A0A7L9W8W9_NDM,A0A7L9F8J2	1,013,180	3_PNGase-F_Treated_Purified_Virions_2-8883-15004_15004	458	466
TRUE	0.1713	(K)FLPFQQFGR(D)	97%	1.7527	0.8042		570.3038	1138.5930	2	0.0005	0.4575	5,858.06	3	A0A7L8Z0C9,A0A7G3UUL1,A0A7L9W8W9_NDM	1,321,460	3_PNGase-F_Treated_Purified_Virions_2-24826-34144_34144	559	567
TRUE	0.1713	(K)FLPFQQFGR(D)	96%	1.7690	0.8141		570.3038	1138.5930	2	0.0005	0.4575	5,881.52	3	A0A7L8Z0C9,A0A7G3UUL1,A0A7L9W8W9_NDM	1,018,840	3_PNGase-F_Treated_Purified_Virions_1-23437-33461_33461	559	567
TRUE	0.1713	(K)FLPFQQFGR(D)	96%	1.7673	0.7981		570.3040	1138.5934	2	0.0009	0.7788	5,861.09	3	A0A7L8Z0C9,A0A7G3UUL1,A0A7L9W8W9_NDM	823,990	3_PNGase-F_Treated_Purified_Virions_1-23326-33328_33328	559	567
TRUE	0.1654	(R)DIADTTDAVR(D)	97%	1.3291	0.7683		538.7649	1075.5152	2	0.0005	0.4296	3,347.32	3	A0A7L8Z0C9,A0A7G3UUL1,A0A7L9W8W9_NDM	873,387	3_PNGase-F_Treated_Purified_Virions_2-10953-17690_17690	568	577
TRUE	0.1557	(R)VYSTGSNVFQTR(A)	97%	3.3441	0.8418		679.8397	1357.6649	2	0.0023	1.7080	4,117.61	4	A0A7L8Z0C9,A0A7G3UUL1,A0A7L9W8W9_NDM,A0A7L9F8J2	848,752	3_PNGase-F_Treated_Purified_Virions_2-14966-22618_22618	635	646
TRUE	0.1642	(R)SFIEDLLFNK(V)	97%	2.8636	0.7353		613.3271	1224.6396	2	0.0005	0.3689	6,581.63	4	A0A7L8Z0C9,A0A7G3UUL1,A0A7L9W8W9_NDM,A0A7L9F8J2	1,798,220	3_PNGase-F_Treated_Purified_Virions_2-29084-38976_38976	816	825
TRUE	0.1642	(R)SFIEDLLFNK(V)	96%	2.7287	0.6910		613.3268	1224.6391	2	0.0000	-0.0295	6,581.03	4	A0A7L8Z0C9,A0A7G3UUL1,A0A7L9W8W9_NDM,A0A7L9F8J2	1,370,570	3_PNGase-F_Treated_Purified_Virions_1-27530-38119_38119	816	825
TRUE	0.1507	(K)QYGDcLGDIAAR(D)	97%	2.9417	0.8188	Carbamidomethyl (+57)	669.8084	1337.6023	2	-0.0014	-1.0210	4,436.26	5	A0A7L8Z0C9,A0A7D7LL25,A0A7G3UUL1,A0A7L9W8W9_NDM,A0A7L9F8J2	1,260,660	3_PNGase-F_Treated_Purified_Virions_2-16634-24662_24662	836	847
TRUE	0.1507	(K)QYGDcLGDIAAR(D)	96%	3.1760	0.8291	Carbamidomethyl (+57)	669.8084	1337.6023	2	-0.0014	-1.0210	4,467.28	5	A0A7L8Z0C9,A0A7D7LL25,A0A7G3UUL1,A0A7L9W8W9_NDM,A0A7L9F8J2	1,224,550	3_PNGase-F_Treated_Purified_Virions_1-15683-24220_24220	836	847
TRUE	0.1670	(K)LIANQFNSAIGK(I)	96%	2.6862	0.7259		638.3564	1274.6983	2	0.0000	-0.0251	4,629.21	4	A0A7L8Z0C9,A0A7G3UUL1,A0A7L9W8W9_NDM,A0A7L9F8J2	301,302	3_PNGase-F_Treated_Purified_Virions_1-16591-25293_25293	922	933
TRUE	0.1670	(K)LIANQFNSAIGK(I)	95%	1.8770	0.5454		638.3569	1274.6992	2	0.0008	0.6447	4,590.89	4	A0A7L8Z0C9,A0A7G3UUL1,A0A7L9W8W9_NDM,A0A7L9F8J2	520,886	3_PNGase-F_Treated_Purified_Virions_2-17529-25700_25700	922	933
TRUE	0.2091	(K)IQDSLSSTASALGK(L)	96%	3.8149	0.8918		689.3645	1376.7145	2	-0.0004	-0.3039	4,433.10	3	A0A7L8Z0C9,A0A7L9W8W9_NDM,A0A7L9F8J2	357,144	3_PNGase-F_Treated_Purified_Virions_1-15491-23994_23994	934	947
TRUE	0.1421	(R)LDKVEAEVQIDR(L)	100%	2.6277	0.5532		472.2569	1413.7488	3	0.0022	1.5550	4,037.08	4	A0A7L8Z0C9,A0A7D7LL25,A0A7G3UUL1,A0A7L9W8W9_NDM	355,296	3_PNGase-F_Treated_Purified_Virions_2-14497-22074_22074	984	995
TRUE	0.1652	(R)LQSLQTYVTQQLIR(A)	97%	3.5916	0.8139		845.9792	1689.9439	2	0.0024	1.4040	6,236.56	3	A0A7L8Z0C9,A0A7G3UUL1,A0A7L9W8W9_NDM	2,320,250	3_PNGase-F_Treated_Purified_Virions_2-27054-36672_36672	1001	1014
TRUE	0.1652	(R)LQSLQTYVTQQLIR(A)	96%	3.7699	0.7898		845.9786	1689.9427	2	0.0012	0.6817	6,235.21	3	A0A7L8Z0C9,A0A7G3UUL1,A0A7L9W8W9_NDM	1,881,990	3_PNGase-F_Treated_Purified_Virions_1-25481-35803_35803	1001	1014
TRUE	0.1741	(R)ASANLAATK(M)	96%	2.7680	0.6600		423.7376	845.4607	2	0.0000	-0.0034	2,267.86	3	A0A7L8Z0C9,A0A7G3UUL1,A0A7L9W8W9_NDM	598,948	3_PNGase-F_Treated_Purified_Virions_1-5767-11060_11060	1020	1028
TRUE	0.1741	(R)ASANLAATK(M)	96%	2.8312	0.6200		423.7377	845.4609	2	0.0002	0.2129	2,270.84	3	A0A7L8Z0C9,A0A7G3UUL1,A0A7L9W8W9_NDM	606,657	3_PNGase-F_Treated_Purified_Virions_1-5779-11077_11077	1020	1028
TRUE	0.1786	(K)RVDFcGK(G)	90%	2.3953	0.6864	Carbamidomethyl (+57)	441.2178	880.4211	2	-0.0016	-1.7930	2,632.00	3	A0A7L8Z0C9,A0A7G3UUL1,A0A7L9W8W9_NDM	718,518	3_PNGase-F_Treated_Purified_Virions_1-7233-13127_13127	1039	1045
TRUE	0.1786	(K)RVDFcGK(G)	90%	2.2308	0.6454	Carbamidomethyl (+57)	441.2180	880.4215	2	-0.0011	-1.2390	2,577.62	3	A0A7L8Z0C9,A0A7G3UUL1,A0A7L9W8W9_NDM	319,475	3_PNGase-F_Treated_Purified_Virions_2-7380-13042_13042	1039	1045
**Spike glycoprotein OS=Severe acute respiratory syndrome coronavirus 2 OX=2697049 GN=S PE=3 SV=1 Accession A0A7G3UUL1**																		
TRUE	0.1733	(R)GWIFGTTLDSK(T)	97%	2.5328	0.8053		612.8166	1223.6186	2	-0.0002	-0.1992	6,011.15	4	A0A7L8AB65,A0A7L8Z0C9,A0A7D7LL25,A0A7L9W8W9_NDM	346,633	3_PNGase-F_Treated_Purified_Virions_2-25715-35163_35163	103	113
TRUE	0.1733	(R)GWIFGTTLDSK(T)	97%	2.4684	0.8142		612.8170	1223.6194	2	0.0005	0.3989	5,989.61	4	A0A7L8AB65,A0A7L8Z0C9,A0A7D7LL25,A0A7L9W8W9_NDM	358,879	3_PNGase-F_Treated_Purified_Virions_2-25582-35016_35016	103	113
TRUE	0.1733	(R)GWIFGTTLDSK(T)	96%	2.7065	0.8265		612.8171	1223.6196	2	0.0007	0.5983	6,006.27	4	A0A7L8AB65,A0A7L8Z0C9,A0A7D7LL25,A0A7L9W8W9_NDM	523,138	3_PNGase-F_Treated_Purified_Virions_1-24137-34277_34277	103	113
TRUE	0.1686	(K)SWMESEFR(V)	97%	1.6456	0.9016		536.2317	1070.4488	2	-0.0002	-0.2275	4,952.82	4	A0A7L8AB65,A0A7L8Z0C9,A0A7D7LL25,A0A7L9W8W9_NDM	375,500	3_PNGase-F_Treated_Purified_Virions_2-19601-28112_28112	151	158
TRUE	0.1686	(K)SWMESEFR(V)	96%	1.8237	0.8855		536.2322	1070.4498	2	0.0007	0.6840	4,984.73	4	A0A7L8AB65,A0A7L8Z0C9,A0A7D7LL25,A0A7L9W8W9_NDM	511,569	3_PNGase-F_Treated_Purified_Virions_1-18520-27618_27618	151	158
TRUE	0.1688	(R)FQTLLALHR(S)	100%	2.5350	0.7561		366.8854	1097.6344	3	-0.0003	-0.2547	4,960.98	5	A0A7L8AB65,A0A7L8Z0C9,A0A7D7LL25,A0A7L9W8W9_NDM,A0A7L9F8J2	435,797	3_PNGase-F_Treated_Purified_Virions_2-19651-28167_28167	238	246
TRUE	0.1688	(R)FQTLLALHR(S)	96%	2.2677	0.6768		366.8856	1097.6348	3	0.0002	0.1620	4,994.33	5	A0A7L8AB65,A0A7L8Z0C9,A0A7D7LL25,A0A7L9W8W9_NDM,A0A7L9F8J2	423,990	3_PNGase-F_Treated_Purified_Virions_1-18576-27682_27682	238	246
TRUE	0.1580	(K)GIYQTSNFR(V)	97%	1.8943	0.7794		543.2726	1084.5307	2	0.0006	0.5209	3,814.02	4	A0A7L8Z0C9,A0A7D7LL25,A0A7L9W8W9_NDM,A0A7L9F8J2	626,867	3_PNGase-F_Treated_Purified_Virions_2-13337-20648_20648	311	319
TRUE	0.1580	(K)GIYQTSNFR(V)	96%	2.0406	0.8229		543.2713	1084.5280	2	-0.0021	-1.9530	3,844.11	4	A0A7L8Z0C9,A0A7D7LL25,A0A7L9W8W9_NDM,A0A7L9F8J2	657,407	3_PNGase-F_Treated_Purified_Virions_1-12603-20324_20324	311	319
TRUE	0.1585	(R)VQPTESIVR(F)	97%	2.1545	0.5921		514.7906	1027.5667	2	0.0005	0.4630	3,410.59	4	A0A7L8Z0C9,A0A7D7LL25,A0A7L9W8W9_NDM,A0A7L9F8J2	661,845	3_PNGase-F_Treated_Purified_Virions_2-11276-18092_18092	320	328
TRUE	0.1585	(R)VQPTESIVR(F)	96%	1.9877	0.5545		514.7907	1027.5669	2	0.0006	0.5816	3,440.76	4	A0A7L8Z0C9,A0A7D7LL25,A0A7L9W8W9_NDM,A0A7L9F8J2	780,276	3_PNGase-F_Treated_Purified_Virions_1-10733-17882_17882	320	328
TRUE	0.1585	(R)VQPTESIVR(F)	95%	1.7273	0.5011		514.7907	1027.5669	2	0.0006	0.5816	3,389.70	4	A0A7L8Z0C9,A0A7D7LL25,A0A7L9W8W9_NDM,A0A7L9F8J2	365,253	3_PNGase-F_Treated_Purified_Virions_2-11167-17958_17958	320	328
TRUE	0.1585	(R)VQPTESIVR(F)	94%	1.5213	0.4637		514.7908	1027.5670	2	0.0007	0.7003	3,419.76	4	A0A7L8Z0C9,A0A7D7LL25,A0A7L9W8W9_NDM,A0A7L9F8J2	161,472	3_PNGase-F_Treated_Purified_Virions_1-10633-17751_17751	320	328
TRUE	0.2136	(R)FASVYAWNR(K)	97%	2.3151	0.7888		557.2776	1112.5406	2	0.0005	0.4069	4,822.20	2	A0A7L8Z0C9,A0A7L9W8W9_NDM	1,047,410	3_PNGase-F_Treated_Purified_Virions_2-18859-27242_27242	347	355
TRUE	0.2136	(R)FASVYAWNR(K)	96%	2.1253	0.7141		557.2780	1112.5415	2	0.0013	1.1740	4,851.89	2	A0A7L8Z0C9,A0A7L9W8W9_NDM	716,410	3_PNGase-F_Treated_Purified_Virions_1-17828-26767_26767	347	355
TRUE	0.2124	(K)cYGVSPTK(L)	96%	2.1334	0.7404	Carbamidomethyl (+57)	456.2185	910.4225	2	0.0006	0.6417	2,759.24	2	A0A7L8Z0C9,A0A7L9W8W9_NDM	257,199	3_PNGase-F_Treated_Purified_Virions_1-7740-13842_13842	379	386
TRUE	0.2124	(K)cYGVSPTK(L)	96%	1.6549	0.6837	Carbamidomethyl (+57)	456.2182	910.4218	2	-0.0001	-0.1619	2,716.87	2	A0A7L8Z0C9,A0A7L9W8W9_NDM	131,418	3_PNGase-F_Treated_Purified_Virions_2-7972-13846_13846	379	386
TRUE	0.1624	(K)IADYNYK(L)	90%	2.0170	0.7673		443.7220	885.4294	2	0.0061	6.8660	3,088.53	4	A0A7L8AB65,A0A7L8Z0C9,A0A7D7LL25,A0A7L9W8W9_NDM	1,292,480	3_PNGase-F_Treated_Purified_Virions_1-9243-15825_15825	418	424
TRUE	0.1624	(K)IADYNYK(L)	90%	1.7636	0.7470		443.7206	885.4267	2	0.0034	3.8360	3,062.68	4	A0A7L8AB65,A0A7L8Z0C9,A0A7D7LL25,A0A7L9W8W9_NDM	4,307,390	3_PNGase-F_Treated_Purified_Virions_2-9631-15966_15966	418	424
TRUE	0.1846	(K)FLPFQQFGR(D)	97%	1.7527	0.8042		570.3038	1138.5930	2	0.0005	0.4575	5,858.06	3	A0A7L8AB65,A0A7L8Z0C9,A0A7L9W8W9_NDM	1,321,460	3_PNGase-F_Treated_Purified_Virions_2-24826-34144_34144	559	567
TRUE	0.1846	(K)FLPFQQFGR(D)	96%	1.7690	0.8141		570.3038	1138.5930	2	0.0005	0.4575	5,881.52	3	A0A7L8AB65,A0A7L8Z0C9,A0A7L9W8W9_NDM	1,018,840	3_PNGase-F_Treated_Purified_Virions_1-23437-33461_33461	559	567
TRUE	0.1846	(K)FLPFQQFGR(D)	96%	1.7673	0.7981		570.3040	1138.5934	2	0.0009	0.7788	5,861.09	3	A0A7L8AB65,A0A7L8Z0C9,A0A7L9W8W9_NDM	823,990	3_PNGase-F_Treated_Purified_Virions_1-23326-33328_33328	559	567
TRUE	0.1782	(R)DIADTTDAVR(D)	97%	1.3291	0.7683		538.7649	1075.5152	2	0.0005	0.4296	3,347.32	3	A0A7L8AB65,A0A7L8Z0C9,A0A7L9W8W9_NDM	873,387	3_PNGase-F_Treated_Purified_Virions_2-10953-17690_17690	568	577
TRUE	0.1678	(R)VYSTGSNVFQTR(A)	97%	3.3441	0.8418		679.8397	1357.6649	2	0.0023	1.7080	4,117.61	4	A0A7L8AB65,A0A7L8Z0C9,A0A7L9W8W9_NDM,A0A7L9F8J2	848,752	3_PNGase-F_Treated_Purified_Virions_2-14966-22618_22618	635	646
TRUE	0.1769	(R)SFIEDLLFNK(V)	97%	2.8636	0.7353		613.3271	1224.6396	2	0.0005	0.3689	6,581.63	4	A0A7L8AB65,A0A7L8Z0C9,A0A7L9W8W9_NDM,A0A7L9F8J2	1,798,220	3_PNGase-F_Treated_Purified_Virions_2-29084-38976_38976	816	825
TRUE	0.1769	(R)SFIEDLLFNK(V)	96%	2.7287	0.6910		613.3268	1224.6391	2	0.0000	-0.0295	6,581.03	4	A0A7L8AB65,A0A7L8Z0C9,A0A7L9W8W9_NDM,A0A7L9F8J2	1,370,570	3_PNGase-F_Treated_Purified_Virions_1-27530-38119_38119	816	825
TRUE	0.1624	(K)QYGDcLGDIAAR(D)	97%	2.9417	0.8188	Carbamidomethyl (+57)	669.8084	1337.6023	2	-0.0014	-1.0210	4,436.26	5	A0A7L8AB65,A0A7L8Z0C9,A0A7D7LL25,A0A7L9W8W9_NDM,A0A7L9F8J2	1,260,660	3_PNGase-F_Treated_Purified_Virions_2-16634-24662_24662	836	847
TRUE	0.1624	(K)QYGDcLGDIAAR(D)	96%	3.1760	0.8291	Carbamidomethyl (+57)	669.8084	1337.6023	2	-0.0014	-1.0210	4,467.28	5	A0A7L8AB65,A0A7L8Z0C9,A0A7D7LL25,A0A7L9W8W9_NDM,A0A7L9F8J2	1,224,550	3_PNGase-F_Treated_Purified_Virions_1-15683-24220_24220	836	847
TRUE	0.1800	(K)LIANQFNSAIGK(I)	96%	2.6862	0.7259		638.3564	1274.6983	2	0.0000	-0.0251	4,629.21	4	A0A7L8AB65,A0A7L8Z0C9,A0A7L9W8W9_NDM,A0A7L9F8J2	301,302	3_PNGase-F_Treated_Purified_Virions_1-16591-25293_25293	922	933
TRUE	0.1800	(K)LIANQFNSAIGK(I)	95%	1.8770	0.5454		638.3569	1274.6992	2	0.0008	0.6447	4,590.89	4	A0A7L8AB65,A0A7L8Z0C9,A0A7L9W8W9_NDM,A0A7L9F8J2	520,886	3_PNGase-F_Treated_Purified_Virions_2-17529-25700_25700	922	933
TRUE	0.1531	(R)LDKVEAEVQIDR(L)	100%	2.6277	0.5532		472.2569	1413.7488	3	0.0022	1.5550	4,037.08	4	A0A7L8AB65,A0A7L8Z0C9,A0A7D7LL25,A0A7L9W8W9_NDM	355,296	3_PNGase-F_Treated_Purified_Virions_2-14497-22074_22074	984	995
TRUE	0.1780	(R)LQSLQTYVTQQLIR(A)	97%	3.5916	0.8139		845.9792	1689.9439	2	0.0024	1.4040	6,236.56	3	A0A7L8AB65,A0A7L8Z0C9,A0A7L9W8W9_NDM	2,320,250	3_PNGase-F_Treated_Purified_Virions_2-27054-36672_36672	1001	1014
TRUE	0.1780	(R)LQSLQTYVTQQLIR(A)	96%	3.7699	0.7898		845.9786	1689.9427	2	0.0012	0.6817	6,235.21	3	A0A7L8AB65,A0A7L8Z0C9,A0A7L9W8W9_NDM	1,881,990	3_PNGase-F_Treated_Purified_Virions_1-25481-35803_35803	1001	1014
TRUE	0.1876	(R)ASANLAATK(M)	96%	2.7680	0.6600		423.7376	845.4607	2	0.0000	-0.0034	2,267.86	3	A0A7L8AB65,A0A7L8Z0C9,A0A7L9W8W9_NDM	598,948	3_PNGase-F_Treated_Purified_Virions_1-5767-11060_11060	1020	1028
TRUE	0.1876	(R)ASANLAATK(M)	96%	2.8312	0.6200		423.7377	845.4609	2	0.0002	0.2129	2,270.84	3	A0A7L8AB65,A0A7L8Z0C9,A0A7L9W8W9_NDM	606,657	3_PNGase-F_Treated_Purified_Virions_1-5779-11077_11077	1020	1028
TRUE	0.1924	(K)RVDFcGK(G)	90%	2.3953	0.6864	Carbamidomethyl (+57)	441.2178	880.4211	2	-0.0016	-1.7930	2,632.00	3	A0A7L8AB65,A0A7L8Z0C9,A0A7L9W8W9_NDM	718,518	3_PNGase-F_Treated_Purified_Virions_1-7233-13127_13127	1039	1045
TRUE	0.1924	(K)RVDFcGK(G)	90%	2.2308	0.6454	Carbamidomethyl (+57)	441.2180	880.4215	2	-0.0011	-1.2390	2,577.62	3	A0A7L8AB65,A0A7L8Z0C9,A0A7L9W8W9_NDM	319,475	3_PNGase-F_Treated_Purified_Virions_2-7380-13042_13042	1039	1045
**Surface glycoprotein OS=Severe acute respiratory syndrome coronavirus 2 OX=2697049 GN=S PE=4 SV=1 A0A7L9F8J2_NDM [c]**																		
TRUE	0.0919	(K)NIDGYFK(I)	90%	1.6610	0.6689		428.7141	855.4136	2	0.0008	0.9796	4,236.03	3	A0A7L8AB65,A0A7L8Z0C9,A0A7L9W8W9_NDM	1,065,270	3_PNGase-F_Treated_Purified_Virions_2-15582-23376_23376	196	202
TRUE	0.0919	(K)NIDGYFK(I)	90%	1.5320	0.6494		428.7140	855.4134	2	0.0007	0.7658	4,265.37	3	A0A7L8AB65,A0A7L8Z0C9,A0A7L9W8W9_NDM	801,284	3_PNGase-F_Treated_Purified_Virions_1-14714-22977_22977	196	202
TRUE	0.1067	(K)HTPINLVR(D)	96%	1.5640	0.7021		475.2830	948.5514	2	0.0009	0.9019	3,563.24	3	A0A7L8AB65,A0A7L8Z0C9,A0A7L9W8W9_NDM	833,341	3_PNGase-F_Treated_Purified_Virions_2-12093-19080_19080	207	214
TRUE	0.1067	(K)HTPINLVR(D)	96%	1.8884	0.7449		475.2831	948.5516	2	0.0010	1.0950	3,594.57	3	A0A7L8AB65,A0A7L8Z0C9,A0A7L9W8W9_NDM	869,729	3_PNGase-F_Treated_Purified_Virions_1-11510-18855_18855	207	214
TRUE	0.0719	(R)FQTLLALHR(S)	100%	2.5350	0.7561		366.8854	1097.6344	3	-0.0003	-0.2547	4,960.98	5	A0A7L8AB65,A0A7L8Z0C9,A0A7D7LL25,A0A7G3UUL1,A0A7L9W8W9_NDM	435,797	3_PNGase-F_Treated_Purified_Virions_2-19651-28167_28167	238	246
TRUE	0.0719	(R)FQTLLALHR(S)	96%	2.2677	0.6768		366.8856	1097.6348	3	0.0002	0.1620	4,994.33	5	A0A7L8AB65,A0A7L8Z0C9,A0A7D7LL25,A0A7G3UUL1,A0A7L9W8W9_NDM	423,990	3_PNGase-F_Treated_Purified_Virions_1-18576-27682_27682	238	246
TRUE	0.0673	(K)GIYQTSNFR(V)	97%	1.8943	0.7794		543.2726	1084.5307	2	0.0006	0.5209	3,814.02	4	A0A7L8Z0C9,A0A7D7LL25,A0A7G3UUL1,A0A7L9W8W9_NDM	626,867	3_PNGase-F_Treated_Purified_Virions_2-13337-20648_20648	311	319
TRUE	0.0673	(K)GIYQTSNFR(V)	96%	2.0406	0.8229		543.2713	1084.5280	2	-0.0021	-1.9530	3,844.11	4	A0A7L8Z0C9,A0A7D7LL25,A0A7G3UUL1,A0A7L9W8W9_NDM	657,407	3_PNGase-F_Treated_Purified_Virions_1-12603-20324_20324	311	319
TRUE	0.0675	(R)VQPTESIVR(F)	97%	2.1545	0.5921		514.7906	1027.5667	2	0.0005	0.4630	3,410.59	4	A0A7L8Z0C9,A0A7D7LL25,A0A7G3UUL1,A0A7L9W8W9_NDM	661,845	3_PNGase-F_Treated_Purified_Virions_2-11276-18092_18092	320	328
TRUE	0.0675	(R)VQPTESIVR(F)	96%	1.9877	0.5545		514.7907	1027.5669	2	0.0006	0.5816	3,440.76	4	A0A7L8Z0C9,A0A7D7LL25,A0A7G3UUL1,A0A7L9W8W9_NDM	780,276	3_PNGase-F_Treated_Purified_Virions_1-10733-17882_17882	320	328
TRUE	0.0675	(R)VQPTESIVR(F)	95%	1.7273	0.5011		514.7907	1027.5669	2	0.0006	0.5816	3,389.70	4	A0A7L8Z0C9,A0A7D7LL25,A0A7G3UUL1,A0A7L9W8W9_NDM	365,253	3_PNGase-F_Treated_Purified_Virions_2-11167-17958_17958	320	328
TRUE	0.0675	(R)VQPTESIVR(F)	94%	1.5213	0.4637		514.7908	1027.5670	2	0.0007	0.7003	3,419.76	4	A0A7L8Z0C9,A0A7D7LL25,A0A7G3UUL1,A0A7L9W8W9_NDM	161,472	3_PNGase-F_Treated_Purified_Virions_1-10633-17751_17751	320	328
TRUE	0.0000	(R)FPDITNLcPFGEVFDATR(F)	97%	5.2669	0.8652	Carbamidomethyl (+57)	1049.9979	2097.9813	2	-0.0019	-0.9235	6,632.94	1	A0A7L9W8W9_NDM	1,795,360	3_PNGase-F_Treated_Purified_Virions_2-29400-39326_39326	329	346
TRUE	0.0000	(R)FPDITNLcPFGEVFDATR(F)	96%	5.1096	0.8664	Carbamidomethyl (+57)	1049.9973	2097.9801	2	-0.0032	-1.5050	6,631.25	1	A0A7L9W8W9_NDM	1,339,390	3_PNGase-F_Treated_Purified_Virions_1-27840-38462_38462	329	346
TRUE	0.0920	(K)LNDLcFTNVYADSFVIR(G)	97%	3.7152	0.7610	Carbamidomethyl (+57)	1024.0061	2045.9977	2	0.0095	4.6500	6,488.96	3	A0A7L8Z0C9,A0A7D7LL25,A0A7L9W8W9_NDM	908,975	3_PNGase-F_Treated_Purified_Virions_2-28556-38361_38361	387	403
TRUE	0.0920	(K)LNDLcFTNVYADSFVIR(G)	96%	4.0813	0.8234	Carbamidomethyl (+57)	1024.0062	2045.9979	2	0.0098	4.7700	6,487.75	3	A0A7L8Z0C9,A0A7D7LL25,A0A7L9W8W9_NDM	886,567	3_PNGase-F_Treated_Purified_Virions_1-26981-37492_37492	387	403
TRUE	0.0712	(K)VGGNYNYLYR(L)	97%	3.4868	0.7657		609.7985	1217.5825	2	-0.0004	-0.3092	4,337.90	4	A0A7L8AB65,A0A7L8Z0C9,A0A7D7LL25,A0A7L9W8W9_NDM	1,383,380	3_PNGase-F_Treated_Purified_Virions_2-16111-24024_24024	445	454
TRUE	0.0712	(K)VGGNYNYLYR(L)	96%	3.3406	0.7891		609.7991	1217.5837	2	0.0008	0.6926	4,369.26	4	A0A7L8AB65,A0A7L8Z0C9,A0A7D7LL25,A0A7L9W8W9_NDM	1,363,820	3_PNGase-F_Treated_Purified_Virions_1-15222-23620_23620	445	454
TRUE	0.0786	(R)KSNLKPFER(D)	100%	2.1141	0.6261		373.5487	1117.6244	3	-0.0001	-0.0801	2,884.70	4	A0A7L8AB65,A0A7L8Z0C9,A0A7D7LL25,A0A7L9W8W9_NDM	1,289,000	3_PNGase-F_Treated_Purified_Virions_2-8780-14875_14875	458	466
TRUE	0.0786	(R)KSNLKPFER(D)	96%	1.6289	0.5833		373.5487	1117.6243	3	-0.0002	-0.1619	2,916.20	4	A0A7L8AB65,A0A7L8Z0C9,A0A7D7LL25,A0A7L9W8W9_NDM	739,797	3_PNGase-F_Treated_Purified_Virions_1-8449-14777_14777	458	466
TRUE	0.0786	(R)KSNLKPFER(D)	93%	1.5707	0.4993		373.5486	1117.6240	3	-0.0005	-0.4074	2,905.27	4	A0A7L8AB65,A0A7L8Z0C9,A0A7D7LL25,A0A7L9W8W9_NDM	1,013,180	3_PNGase-F_Treated_Purified_Virions_2-8883-15004_15004	458	466
TRUE	0.0601	(R)VVVLSFELLHAPATVcGPK(K)	100%	4.9151	0.7457	Carbamidomethyl (+57)	679.7120	2036.1141	3	0.0011	0.5207	6,370.21	3	A0A7L8Z0C9,A0A7D7LL25,A0A7L9W8W9_NDM	1,900,120	3_PNGase-F_Treated_Purified_Virions_2-27840-37566_37566	510	528
TRUE	0.0601	(R)VVVLSFELLHAPATVcGPK(K)	96%	3.4543	0.7949	Carbamidomethyl (+57)	679.7123	2036.1152	3	0.0022	1.0600	6,368.36	3	A0A7L8Z0C9,A0A7D7LL25,A0A7L9W8W9_NDM	649,757	3_PNGase-F_Treated_Purified_Virions_1-26257-36689_36689	510	528
TRUE	0.0715	(R)VYSTGSNVFQTR(A)	97%	3.3441	0.8418		679.8397	1357.6649	2	0.0023	1.7080	4,117.61	4	A0A7L8AB65,A0A7L8Z0C9,A0A7G3UUL1,A0A7L9W8W9_NDM	848,752	3_PNGase-F_Treated_Purified_Virions_2-14966-22618_22618	635	646
TRUE	0.0754	(R)SFIEDLLFNK(V)	97%	2.8636	0.7353		613.3271	1224.6396	2	0.0005	0.3689	6,581.63	4	A0A7L8AB65,A0A7L8Z0C9,A0A7G3UUL1,A0A7L9W8W9_NDM	1,798,220	3_PNGase-F_Treated_Purified_Virions_2-29084-38976_38976	816	825
TRUE	0.0754	(R)SFIEDLLFNK(V)	96%	2.7287	0.6910		613.3268	1224.6391	2	0.0000	-0.0295	6,581.03	4	A0A7L8AB65,A0A7L8Z0C9,A0A7G3UUL1,A0A7L9W8W9_NDM	1,370,570	3_PNGase-F_Treated_Purified_Virions_1-27530-38119_38119	816	825
TRUE	0.0692	(K)QYGDcLGDIAAR(D)	97%	2.9417	0.8188	Carbamidomethyl (+57)	669.8084	1337.6023	2	-0.0014	-1.0210	4,436.26	5	A0A7L8AB65,A0A7L8Z0C9,A0A7D7LL25,A0A7G3UUL1,A0A7L9W8W9_NDM	1,260,660	3_PNGase-F_Treated_Purified_Virions_2-16634-24662_24662	836	847
TRUE	0.0692	(K)QYGDcLGDIAAR(D)	96%	3.1760	0.8291	Carbamidomethyl (+57)	669.8084	1337.6023	2	-0.0014	-1.0210	4,467.28	5	A0A7L8AB65,A0A7L8Z0C9,A0A7D7LL25,A0A7G3UUL1,A0A7L9W8W9_NDM	1,224,550	3_PNGase-F_Treated_Purified_Virions_1-15683-24220_24220	836	847
TRUE	0.0767	(K)LIANQFNSAIGK(I)	96%	2.6862	0.7259		638.3564	1274.6983	2	0.0000	-0.0251	4,629.21	4	A0A7L8AB65,A0A7L8Z0C9,A0A7G3UUL1,A0A7L9W8W9_NDM	301,302	3_PNGase-F_Treated_Purified_Virions_1-16591-25293_25293	922	933
TRUE	0.0767	(K)LIANQFNSAIGK(I)	95%	1.8770	0.5454		638.3569	1274.6992	2	0.0008	0.6447	4,590.89	4	A0A7L8AB65,A0A7L8Z0C9,A0A7G3UUL1,A0A7L9W8W9_NDM	520,886	3_PNGase-F_Treated_Purified_Virions_2-17529-25700_25700	922	933
TRUE	0.0960	(K)IQDSLSSTASALGK(L)	96%	3.8149	0.8918		689.3645	1376.7145	2	-0.0004	-0.3039	4,433.10	3	A0A7L8AB65,A0A7L8Z0C9,A0A7L9W8W9_NDM	357,144	3_PNGase-F_Treated_Purified_Virions_1-15491-23994_23994	934	947
TRUE	0.0780	(K)LQDVVNQNAQALNTLVK(Q)	97%	2.7123	0.8093		934.5232	1867.0318	2	0.0154	8.2250	5,457.69	3	A0A7L8Z0C9,A0A7D7LL25,A0A7L9W8W9_NDM	1,055,200	3_PNGase-F_Treated_Purified_Virions_2-22494-31465_31465	948	964
TRUE	0.0780	(K)LQDVVNQNAQALNTLVK(Q)	96%	3.7087	0.8221		934.5233	1867.0320	2	0.0155	8.2900	5,483.31	3	A0A7L8Z0C9,A0A7D7LL25,A0A7L9W8W9_NDM	775,126	3_PNGase-F_Treated_Purified_Virions_1-21180-30835_30835	948	964
TRUE	0.0817	(R)EGVFVSNGTHWFVTQR(N)	96%	3.2124	0.6778		621.9753	1862.9040	3	-0.0024	-1.2650	5,825.46	2	A0A7L8Z0C9,A0A7D7LL25	331,928	3_PNGase-F_Treated_Purified_Virions_1-23141-33100_33100	1092	1107
**Spike glycoprotein OS=Severe acute respiratory syndrome coronavirus 2 OX=2697049 GN=S PE=3 SV=1 Accession A0A7D7LL25**																		
TRUE	0.1580	(R)GWIFGTTLDSK(T)	97%	2.5328	0.8053		612.8166	1223.6186	2	-0.0002	-0.1992	6,011.15	4	A0A7L8AB65,A0A7L8Z0C9,A0A7G3UUL1,A0A7L9W8W9_NDM	346,633	3_PNGase-F_Treated_Purified_Virions_2-25715-35163_35163	103	113
TRUE	0.1580	(R)GWIFGTTLDSK(T)	97%	2.4684	0.8142		612.8170	1223.6194	2	0.0005	0.3989	5,989.61	4	A0A7L8AB65,A0A7L8Z0C9,A0A7G3UUL1,A0A7L9W8W9_NDM	358,879	3_PNGase-F_Treated_Purified_Virions_2-25582-35016_35016	103	113
TRUE	0.1580	(R)GWIFGTTLDSK(T)	96%	2.7065	0.8265		612.8171	1223.6196	2	0.0007	0.5983	6,006.27	4	A0A7L8AB65,A0A7L8Z0C9,A0A7G3UUL1,A0A7L9W8W9_NDM	523,138	3_PNGase-F_Treated_Purified_Virions_1-24137-34277_34277	103	113
TRUE	0.1537	(K)SWMESEFR(V)	97%	1.6456	0.9016		536.2317	1070.4488	2	-0.0002	-0.2275	4,952.82	4	A0A7L8AB65,A0A7L8Z0C9,A0A7G3UUL1,A0A7L9W8W9_NDM	375,500	3_PNGase-F_Treated_Purified_Virions_2-19601-28112_28112	151	158
TRUE	0.1537	(K)SWMESEFR(V)	96%	1.8237	0.8855		536.2322	1070.4498	2	0.0007	0.6840	4,984.73	4	A0A7L8AB65,A0A7L8Z0C9,A0A7G3UUL1,A0A7L9W8W9_NDM	511,569	3_PNGase-F_Treated_Purified_Virions_1-18520-27618_27618	151	158
TRUE	0.1539	(R)FQTLLALHR(S)	100%	2.5350	0.7561		366.8854	1097.6344	3	-0.0003	-0.2547	4,960.98	5	A0A7L8AB65,A0A7L8Z0C9,A0A7G3UUL1,A0A7L9W8W9_NDM,A0A7L9F8J2	435,797	3_PNGase-F_Treated_Purified_Virions_2-19651-28167_28167	238	246
TRUE	0.1539	(R)FQTLLALHR(S)	96%	2.2677	0.6768		366.8856	1097.6348	3	0.0002	0.1620	4,994.33	5	A0A7L8AB65,A0A7L8Z0C9,A0A7G3UUL1,A0A7L9W8W9_NDM,A0A7L9F8J2	423,990	3_PNGase-F_Treated_Purified_Virions_1-18576-27682_27682	238	246
TRUE	0.1441	(K)GIYQTSNFR(V)	97%	1.8943	0.7794		543.2726	1084.5307	2	0.0006	0.5209	3,814.02	4	A0A7L8Z0C9,A0A7G3UUL1,A0A7L9W8W9_NDM,A0A7L9F8J2	626,867	3_PNGase-F_Treated_Purified_Virions_2-13337-20648_20648	311	319
TRUE	0.1441	(K)GIYQTSNFR(V)	96%	2.0406	0.8229		543.2713	1084.5280	2	-0.0021	-1.9530	3,844.11	4	A0A7L8Z0C9,A0A7G3UUL1,A0A7L9W8W9_NDM,A0A7L9F8J2	657,407	3_PNGase-F_Treated_Purified_Virions_1-12603-20324_20324	311	319
TRUE	0.1446	(R)VQPTESIVR(F)	97%	2.1545	0.5921		514.7906	1027.5667	2	0.0005	0.4630	3,410.59	4	A0A7L8Z0C9,A0A7G3UUL1,A0A7L9W8W9_NDM,A0A7L9F8J2	661,845	3_PNGase-F_Treated_Purified_Virions_2-11276-18092_18092	320	328
TRUE	0.1446	(R)VQPTESIVR(F)	96%	1.9877	0.5545		514.7907	1027.5669	2	0.0006	0.5816	3,440.76	4	A0A7L8Z0C9,A0A7G3UUL1,A0A7L9W8W9_NDM,A0A7L9F8J2	780,276	3_PNGase-F_Treated_Purified_Virions_1-10733-17882_17882	320	328
TRUE	0.1446	(R)VQPTESIVR(F)	95%	1.7273	0.5011		514.7907	1027.5669	2	0.0006	0.5816	3,389.70	4	A0A7L8Z0C9,A0A7G3UUL1,A0A7L9W8W9_NDM,A0A7L9F8J2	365,253	3_PNGase-F_Treated_Purified_Virions_2-11167-17958_17958	320	328
TRUE	0.1446	(R)VQPTESIVR(F)	94%	1.5213	0.4637		514.7908	1027.5670	2	0.0007	0.7003	3,419.76	4	A0A7L8Z0C9,A0A7G3UUL1,A0A7L9W8W9_NDM,A0A7L9F8J2	161,472	3_PNGase-F_Treated_Purified_Virions_1-10633-17751_17751	320	328
TRUE	0.1968	(K)LNDLcFTNVYADSFVIR(G)	97%	3.7152	0.7610	Carbamidomethyl (+57)	1024.0061	2045.9977	2	0.0095	4.6500	6,488.96	3	A0A7L8Z0C9,A0A7L9W8W9_NDM,A0A7L9F8J2	908,975	3_PNGase-F_Treated_Purified_Virions_2-28556-38361_38361	387	403
TRUE	0.1968	(K)LNDLcFTNVYADSFVIR(G)	96%	4.0813	0.8234	Carbamidomethyl (+57)	1024.0062	2045.9979	2	0.0098	4.7700	6,487.75	3	A0A7L8Z0C9,A0A7L9W8W9_NDM,A0A7L9F8J2	886,567	3_PNGase-F_Treated_Purified_Virions_1-26981-37492_37492	387	403
TRUE	0.1481	(K)IADYNYK(L)	90%	2.0170	0.7673		443.7220	885.4294	2	0.0061	6.8660	3,088.53	4	A0A7L8AB65,A0A7L8Z0C9,A0A7G3UUL1,A0A7L9W8W9_NDM	1,292,480	3_PNGase-F_Treated_Purified_Virions_1-9243-15825_15825	418	424
TRUE	0.1481	(K)IADYNYK(L)	90%	1.7636	0.7470		443.7206	885.4267	2	0.0034	3.8360	3,062.68	4	A0A7L8AB65,A0A7L8Z0C9,A0A7G3UUL1,A0A7L9W8W9_NDM	4,307,390	3_PNGase-F_Treated_Purified_Virions_2-9631-15966_15966	418	424
TRUE	0.1525	(K)VGGNYNYLYR(L)	97%	3.4868	0.7657		609.7985	1217.5825	2	-0.0004	-0.3092	4,337.90	4	A0A7L8AB65,A0A7L8Z0C9,A0A7L9W8W9_NDM,A0A7L9F8J2	1,383,380	3_PNGase-F_Treated_Purified_Virions_2-16111-24024_24024	445	454
TRUE	0.1525	(K)VGGNYNYLYR(L)	96%	3.3406	0.7891		609.7991	1217.5837	2	0.0008	0.6926	4,369.26	4	A0A7L8AB65,A0A7L8Z0C9,A0A7L9W8W9_NDM,A0A7L9F8J2	1,363,820	3_PNGase-F_Treated_Purified_Virions_1-15222-23620_23620	445	454
TRUE	0.1683	(R)KSNLKPFER(D)	100%	2.1141	0.6261		373.5487	1117.6244	3	-0.0001	-0.0801	2,884.70	4	A0A7L8AB65,A0A7L8Z0C9,A0A7L9W8W9_NDM,A0A7L9F8J2	1,289,000	3_PNGase-F_Treated_Purified_Virions_2-8780-14875_14875	458	466
TRUE	0.1683	(R)KSNLKPFER(D)	96%	1.6289	0.5833		373.5487	1117.6243	3	-0.0002	-0.1619	2,916.20	4	A0A7L8AB65,A0A7L8Z0C9,A0A7L9W8W9_NDM,A0A7L9F8J2	739,797	3_PNGase-F_Treated_Purified_Virions_1-8449-14777_14777	458	466
TRUE	0.1683	(R)KSNLKPFER(D)	93%	1.5707	0.4993		373.5486	1117.6240	3	-0.0005	-0.4074	2,905.27	4	A0A7L8AB65,A0A7L8Z0C9,A0A7L9W8W9_NDM,A0A7L9F8J2	1,013,180	3_PNGase-F_Treated_Purified_Virions_2-8883-15004_15004	458	466
TRUE	0.1287	(R)VVVLSFELLHAPATVcGPK(K)	100%	4.9151	0.7457	Carbamidomethyl (+57)	679.7120	2036.1141	3	0.0011	0.5207	6,370.21	3	A0A7L8Z0C9,A0A7L9W8W9_NDM,A0A7L9F8J2	1,900,120	3_PNGase-F_Treated_Purified_Virions_2-27840-37566_37566	510	528
TRUE	0.1287	(R)VVVLSFELLHAPATVcGPK(K)	96%	3.4543	0.7949	Carbamidomethyl (+57)	679.7123	2036.1152	3	0.0022	1.0600	6,368.36	3	A0A7L8Z0C9,A0A7L9W8W9_NDM,A0A7L9F8J2	649,757	3_PNGase-F_Treated_Purified_Virions_1-26257-36689_36689	510	528
TRUE	0.1481	(K)QYGDcLGDIAAR(D)	97%	2.9417	0.8188	Carbamidomethyl (+57)	669.8084	1337.6023	2	-0.0014	-1.0210	4,436.26	5	A0A7L8AB65,A0A7L8Z0C9,A0A7G3UUL1,A0A7L9W8W9_NDM,A0A7L9F8J2	1,260,660	3_PNGase-F_Treated_Purified_Virions_2-16634-24662_24662	836	847
TRUE	0.1481	(K)QYGDcLGDIAAR(D)	96%	3.1760	0.8291	Carbamidomethyl (+57)	669.8084	1337.6023	2	-0.0014	-1.0210	4,467.28	5	A0A7L8AB65,A0A7L8Z0C9,A0A7G3UUL1,A0A7L9W8W9_NDM,A0A7L9F8J2	1,224,550	3_PNGase-F_Treated_Purified_Virions_1-15683-24220_24220	836	847
TRUE	0.1669	(K)LQDVVNQNAQALNTLVK(Q)	97%	2.7123	0.8093		934.5232	1867.0318	2	0.0154	8.2250	5,457.69	3	A0A7L8Z0C9,A0A7L9W8W9_NDM,A0A7L9F8J2	1,055,200	3_PNGase-F_Treated_Purified_Virions_2-22494-31465_31465	948	964
TRUE	0.1669	(K)LQDVVNQNAQALNTLVK(Q)	96%	3.7087	0.8221		934.5233	1867.0320	2	0.0155	8.2900	5,483.31	3	A0A7L8Z0C9,A0A7L9W8W9_NDM,A0A7L9F8J2	775,126	3_PNGase-F_Treated_Purified_Virions_1-21180-30835_30835	948	964
TRUE	0.1396	(R)LDKVEAEVQIDR(L)	100%	2.6277	0.5532		472.2569	1413.7488	3	0.0022	1.5550	4,037.08	4	A0A7L8AB65,A0A7L8Z0C9,A0A7G3UUL1,A0A7L9W8W9_NDM	355,296	3_PNGase-F_Treated_Purified_Virions_2-14497-22074_22074	984	995
TRUE	0.1749	(R)EGVFVSNGTHWFVTQR(N)	96%	3.2124	0.6778		621.9753	1862.9040	3	-0.0024	-1.2650	5,825.46	2	A0A7L8Z0C9,A0A7L9F8J2	331,928	3_PNGase-F_Treated_Purified_Virions_1-23141-33100_33100	1092	1107
**Spike glycoprotein OS=Severe acute respiratory syndrome coronavirus 2 OX=2697049 GN=S PE=3 SV=1 Accession A0A7D5XMN0_NDM [c]**																		
TRUE	0.2745	(K)RFDNPVLPFNDGVYFASTEK(S)	100%	2.6473	0.6536		772.7159	2315.1258	3	0.0034	1.4870	6,263.08	2	A0A7L8Z0C9,A0A7L9W8W9_NDM	8,030,750	3_PNGase-F_Treated_Purified_Virions_2-27217-36852_36852	78	97
TRUE	0.3702	(K)VcEFQFcNDPFLGVYYHK(N)	100%	4.6695	0.8798	Carbamidomethyl (+57), Carbamidomethyl (+57)	775.0156	2322.0250	3	0.0011	0.4929	6,351.23	2	A0A7L8Z0C9,A0A7L9W8W9_NDM	1,460,760	3_PNGase-F_Treated_Purified_Virions_2-27724-37438_37438	130	147
TRUE	0.3702	(K)VcEFQFcNDPFLGVYYHK(N)	96%	4.9430	0.8719	Carbamidomethyl (+57), Carbamidomethyl (+57)	775.0164	2322.0272	3	0.0033	1.4390	6,351.11	2	A0A7L8Z0C9,A0A7L9W8W9_NDM	1,963,620	3_PNGase-F_Treated_Purified_Virions_1-26152-36573_36573	130	147
TRUE	0.0000	(R)DLPQGFSALEPLVDLPIGIDITR(F)	94%	5.2058	0.3290		1240.1747	2478.3348	2	-0.0028	-1.1120	6,749.62	1	A0A7L9W8W9_NDM	1,444,260	3_PNGase-F_Treated_Purified_Virions_2-30114-40118_40118	215	237
TRUE	0.0000	(R)DLPQGFSALEPLVDLPIGIDITR(F)	91%	3.4776	0.2746		827.1200	2478.3382	3	0.0006	0.2364	6,750.22	1	A0A7L9W8W9_NDM	488,681	3_PNGase-F_Treated_Purified_Virions_1-28568-39269_39269	215	237
TRUE	0.0000	(K)YNEDGTITDAVDcALDPLSETK(C)	97%	4.2618	0.8775	Carbamidomethyl (+57)	1214.0477	2426.0809	2	0.0010	0.4001	6,378.96	1	A0A7L9W8W9_NDM	741,945	3_PNGase-F_Treated_Purified_Virions_2-27894-37625_37625	279	300
TRUE	0.0000	(K)YNEDGTITDAVDcALDPLSETK(C)	96%	4.8463	0.8894	Carbamidomethyl (+57)	1214.0502	2426.0858	2	0.0059	2.4120	6,378.45	1	A0A7L9W8W9_NDM	1,101,920	3_PNGase-F_Treated_Purified_Virions_1-26319-36757_36757	279	300
TRUE	0.2745	(K)LPDDFTGcVIAWNSNNLDSK(V)	97%	5.2705	0.9424	Carbamidomethyl (+57)	1133.5253	2265.0360	2	-0.0015	-0.6402	6,188.35	2	A0A7L8Z0C9,A0A7L9W8W9_NDM	579,815	3_PNGase-F_Treated_Purified_Virions_2-26759-36346_36346	425	444
TRUE	0.3602	(R)ALTGIAVEQDKNTQEVFAQVK(Q)	96%	4.1841	0.7620		763.7409	2288.2009	3	-0.0006	-0.2542	5,456.09	2	A0A7L8Z0C9,A0A7L9W8W9_NDM	266,173	3_PNGase-F_Treated_Purified_Virions_1-21022-30653_30653	766	786
TRUE	0.5098	(K)TPPIKDFGGFDFSQILPDPSKPSK(R)	95%	2.8270	0.8015		655.3449	2617.3505	4	0.0072	2.7360	6,440.38	1	A0A7L9W8W9_NDM	585,524	3_PNGase-F_Treated_Purified_Virions_2-28259-38034_38034	791	814
TRUE	0.2867	(K)DHTSPDVDLGDISGIDASVVNIQK(E)	96%	5.0499	0.8717		832.4140	2494.2202	3	0.0010	0.4028	6,116.36	1	A0A7L9W8W9_NDM	343,736	3_PNGase-F_Treated_Purified_Virions_1-24759-35000_35000	1158	1181

4_Inactivated PNGase-F_Treated_Purified_Virions

Valid	Weight	Sequence a,b	Protein identification probability (Prob)	SEQUEST XCorr	SEQUEST deltaCn	Modifications	Observed Mass (Da)	Actual Mass (Da)	Charge	Delta Da	Delta PPM	Retention Time	# Other Proteins	Other Proteins	Total Ion Chromatogram (TIC)	Spectrum ID	Peptide Start Index	Peptide Stop Index
**Surface glycoprotein OS=Severe acute respiratory syndrome coronavirus 2 OX=2697049 GN=S PE=4 SV=1 A0A7L9W8W9_NDM [c]**																		
TRUE	0.0063	(R)TQLPPAYTNSFTR(G)	93%	2.1875	0.7608		748.3802	1494.7459	2	-0.0008	-0.5513	4,627.22	5	A0A7L8AB65,A0A7L8Z0C9,A0A7D7LL25,A0A7G3UUL1,A0A7L9F8J2	267,798	4_Inactivated_PNGase-F_Treated_Purified Virions_1-16963-25132_25132	9	21
TRUE	0.0071	(R)GWIFGTTLDSK(T)	93%	2.5846	0.8111		612.8164	1223.6183	2	-0.0006	-0.4982	6,025.31	4	A0A7L8AB65,A0A7L8Z0C9,A0A7D7LL25,A0A7G3UUL1	262,603	4_Inactivated_PNGase-F_Treated_Purified Virions_1-24989-34390_34390	90	100
TRUE	0.0071	(R)GWIFGTTLDSK(T)	91%	1.8062	0.7146		612.8172	1223.6198	2	0.0010	0.7976	6,017.86	4	A0A7L8AB65,A0A7L8Z0C9,A0A7D7LL25,A0A7G3UUL1	157,260	4_Inactivated_PNGase-F_Treated_Purified Virions_2-21609-32468_32468	90	100
TRUE	0.0192	(K)VcEFQFcNDPFLGVYYHK(N)	98%	1.8994	0.6971	Carbamidomethyl (+57), Carbamidomethyl (+57)	775.0149	2322.0228	3	-0.0011	-0.4529	6,349.75	2	A0A7L8Z0C9,A0A7D5XMN0	250,005	4_Inactivated_PNGase-F_Treated_Purified Virions_2-23511-34649_34649	117	134
TRUE	0.0069	(K)SWMESEFR(V)	93%	1.6519	0.9152		536.2332	1070.4519	2	0.0028	2.6210	4,979.05	4	A0A7L8AB65,A0A7L8Z0C9,A0A7D7LL25,A0A7G3UUL1	305,930	4_Inactivated_PNGase-F_Treated_Purified Virions_1-18929-27453_27453	138	145
TRUE	0.0089	(K)NIDGYFK(I)	93%	1.4635	0.6497		428.7134	855.4123	2	-0.0005	-0.5882	4,276.05	3	A0A7L8AB65,A0A7L8Z0C9,A0A7L9F8J2	1,060,970	4_Inactivated_PNGase-F_Treated_Purified Virions_1-15057-22852_22852	183	189
TRUE	0.0089	(K)NIDGYFK(I)	90%	1.4136	0.6404		428.7139	855.4132	2	0.0005	0.5520	4,271.92	3	A0A7L8AB65,A0A7L8Z0C9,A0A7L9F8J2	752,161	4_Inactivated_PNGase-F_Treated_Purified Virions_2-12019-21119_21119	183	189
TRUE	0.0103	(K)HTPINLVR(D)	93%	1.9083	0.7661		475.2825	948.5504	2	-0.0002	-0.1909	3,588.61	3	A0A7L8AB65,A0A7L8Z0C9,A0A7L9F8J2	696,137	4_Inactivated_PNGase-F_Treated_Purified Virions_1-11636-18571_18571	194	201
TRUE	0.0103	(K)HTPINLVR(D)	91%	1.8660	0.7101		475.2829	948.5512	2	0.0006	0.6448	3,565.99	3	A0A7L8AB65,A0A7L8Z0C9,A0A7L9F8J2	447,323	4_Inactivated_PNGase-F_Treated_Purified Virions_2-8665-16834_16834	194	201
TRUE	0.0070	(R)FQTLLALHR(S)	98%	1.7411	0.5229		366.8858	1097.6355	3	0.0008	0.7453	4,992.36	5	A0A7L8AB65,A0A7L8Z0C9,A0A7D7LL25,A0A7G3UUL1,A0A7L9F8J2	149,623	4_Inactivated_PNGase-F_Treated_Purified Virions_2-15930-25794_25794	225	233
TRUE	0.0070	(R)FQTLLALHR(S)	97%	1.5288	0.6154		366.8853	1097.6340	3	-0.0006	-0.5880	4,994.13	5	A0A7L8AB65,A0A7L8Z0C9,A0A7D7LL25,A0A7G3UUL1,A0A7L9F8J2	139,042	4_Inactivated_PNGase-F_Treated_Purified Virions_1-19022-27555_27555	225	233
TRUE	0.0065	(K)GIYQTSNFR(V)	93%	2.0250	0.7860		543.2731	1084.5317	2	0.0015	1.4210	3,841.61	4	A0A7L8Z0C9,A0A7D7LL25,A0A7G3UUL1,A0A7L9F8J2	925,100	4_Inactivated_PNGase-F_Treated_Purified Virions_1-12760-20062_20062	298	306
TRUE	0.0065	(R)VQPTESIVR(F)	92%	1.4220	0.5163		514.7902	1027.5659	2	-0.0004	-0.3678	3,419.23	4	A0A7L8Z0C9,A0A7D7LL25,A0A7G3UUL1,A0A7L9F8J2	168,668	4_Inactivated_PNGase-F_Treated_Purified Virions_1-10778-17506_17506	307	315
TRUE	0.0065	(R)VQPTESIVR(F)	91%	1.5907	0.5241		514.7902	1027.5658	2	-0.0005	-0.4865	3,413.18	4	A0A7L8Z0C9,A0A7D7LL25,A0A7G3UUL1,A0A7L9F8J2	203,935	4_Inactivated_PNGase-F_Treated_Purified Virions_2-7953-15912_15912	307	315
TRUE	0.0065	(R)VQPTESIVR(F)	91%	1.3860	0.5171		514.7902	1027.5659	2	-0.0004	-0.3678	3,392.50	4	A0A7L8Z0C9,A0A7D7LL25,A0A7G3UUL1,A0A7L9F8J2	123,342	4_Inactivated_PNGase-F_Treated_Purified Virions_2-7852-15781_15781	307	315
TRUE	0.0088	(R)FASVYAWNR(K)	93%	2.7088	0.8889		557.2787	1112.5428	2	0.0027	2.3800	4,874.98	2	A0A7L8Z0C9,A0A7G3UUL1	317,612	4_Inactivated_PNGase-F_Treated_Purified Virions_1-18333-26758_26758	334	342
TRUE	0.0088	(R)FASVYAWNR(K)	91%	2.3036	0.8589		557.2786	1112.5426	2	0.0024	2.1610	4,876.47	2	A0A7L8Z0C9,A0A7G3UUL1	303,701	4_Inactivated_PNGase-F_Treated_Purified Virions_2-15278-25026_25026	334	342
TRUE	0.0087	(K)cYGVSPTK(L)	93%	1.5966	0.8148	Carbamidomethyl (+57)	456.2192	910.4238	2	0.0019	2.0480	2,779.32	2	A0A7L8Z0C9,A0A7G3UUL1	149,312	4_Inactivated_PNGase-F_Treated_Purified Virions_1-7612-13531_13531	366	373
TRUE	0.0087	(K)cYGVSPTK(L)	91%	1.7819	0.7251	Carbamidomethyl (+57)	456.2192	910.4238	2	0.0019	2.0480	2,729.68	2	A0A7L8Z0C9,A0A7G3UUL1	148,769	4_Inactivated_PNGase-F_Treated_Purified Virions_2-5200-12067_12067	366	373
TRUE	0.0067	(K)IADYNYK(L)	90%	1.8556	0.8586		443.7212	885.4278	2	0.0045	5.0760	3,103.10	4	A0A7L8AB65,A0A7L8Z0C9,A0A7D7LL25,A0A7G3UUL1	1,115,660	4_Inactivated_PNGase-F_Treated_Purified Virions_1-9272-15572_15572	405	411
TRUE	0.0067	(K)IADYNYK(L)	90%	1.6961	0.7985		443.7201	885.4256	2	0.0024	2.6660	3,075.25	4	A0A7L8AB65,A0A7L8Z0C9,A0A7D7LL25,A0A7G3UUL1	823,219	4_Inactivated_PNGase-F_Treated_Purified Virions_2-6530-13962_13962	405	411
TRUE	0.0116	(K)IADYNYKLPDDFTGcVIAWNSNNLDSK(V)	98%	1.8445	0.8513	Carbamidomethyl (+57)	1045.1608	3132.4605	3	0.0103	3.2980	6,351.82	2	A0A7D7XU89,A0A7L8Z0C9	53,111	4_Inactivated_PNGase-F_Treated_Purified Virions_2-23524-34663_34663	405	431
TRUE	0.0116	(K)IADYNYKLPDDFTGcVIAWNSNNLDSK(V)	97%	2.1637	0.8766	Carbamidomethyl (+57)	1045.1584	3132.4535	3	0.0034	1.0780	6,356.32	2	A0A7D7XU89,A0A7L8Z0C9	56,920	4_Inactivated_PNGase-F_Treated_Purified Virions_1-26917-36583_36583	405	431
TRUE	0.0143	(K)LPDDFTGcVIAWNSNNLDSK(V)	93%	4.3011	0.9432	Carbamidomethyl (+57)	1133.5265	2265.0384	2	0.0010	0.4371	6,181.58	2	A0A7L8Z0C9,A0A7D5XMN0	261,016	4_Inactivated_PNGase-F_Treated_Purified Virions_1-25887-35420_35420	412	431
TRUE	0.0143	(K)LPDDFTGcVIAWNSNNLDSK(V)	91%	2.4746	0.9400	Carbamidomethyl (+57)	1133.5221	2265.0296	2	-0.0078	-3.4410	6,174.66	2	A0A7L8Z0C9,A0A7D5XMN0	97,202	4_Inactivated_PNGase-F_Treated_Purified Virions_2-22491-33490_33490	412	431
TRUE	0.0069	(K)VGGNYNYLYR(L)	93%	3.5527	0.8049		609.7979	1217.5812	2	-0.0017	-1.4110	4,354.07	4	A0A7L8AB65,A0A7L8Z0C9,A0A7D7LL25,A0A7L9F8J2	622,786	4_Inactivated_PNGase-F_Treated_Purified Virions_1-15451-23342_23342	432	441
TRUE	0.0069	(K)VGGNYNYLYR(L)	91%	2.2351	0.6696		609.7984	1217.5822	2	-0.0006	-0.5095	4,346.59	4	A0A7L8AB65,A0A7L8Z0C9,A0A7D7LL25,A0A7L9F8J2	250,654	4_Inactivated_PNGase-F_Treated_Purified Virions_2-12388-21577_21577	432	441
TRUE	0.0058	(R)VVVLSFELLHAPATVcGPK(K)	98%	4.5895	0.8170	Carbamidomethyl (+57)	679.7122	2036.1146	3	0.0016	0.7904	6,368.86	3	A0A7L8Z0C9,A0A7D7LL25,A0A7L9F8J2	556,905	4_Inactivated_PNGase-F_Treated_Purified Virions_2-23627-34777_34777	497	515
TRUE	0.0058	(R)VVVLSFELLHAPATVcGPK(K)	97%	4.3779	0.8383	Carbamidomethyl (+57)	679.7113	2036.1121	3	-0.0010	-0.4680	6,370.91	3	A0A7L8Z0C9,A0A7D7LL25,A0A7L9F8J2	651,551	4_Inactivated_PNGase-F_Treated_Purified Virions_1-27005-36681_36681	497	515
TRUE	0.0076	(K)FLPFQQFGR(D)	93%	1.6718	0.8265		570.3042	1138.5938	2	0.0014	1.2070	5,889.86	3	A0A7L8AB65,A0A7L8Z0C9,A0A7G3UUL1	453,594	4_Inactivated_PNGase-F_Treated_Purified Virions_1-24179-33483_33483	546	554
TRUE	0.0076	(K)FLPFQQFGR(D)	93%	1.6218	0.7426		570.2994	1138.5842	2	-0.0083	-7.2550	5,869.08	3	A0A7L8AB65,A0A7L8Z0C9,A0A7G3UUL1	476,881	4_Inactivated_PNGase-F_Treated_Purified Virions_1-24057-33344_33344	546	554
TRUE	0.0076	(K)FLPFQQFGR(D)	91%	1.2733	0.8785		570.3035	1138.5925	2	0.0000	0.0290	5,885.88	3	A0A7L8AB65,A0A7L8Z0C9,A0A7G3UUL1	386,237	4_Inactivated_PNGase-F_Treated_Purified Virions_2-20850-31595_31595	546	554
TRUE	0.0069	(R)VYSTGSNVFQTR(A)	93%	3.2350	0.8099		679.8387	1357.6629	2	0.0004	0.2706	4,129.45	4	A0A7L8AB65,A0A7L8Z0C9,A0A7G3UUL1,A0A7L9F8J2	732,431	4_Inactivated_PNGase-F_Treated_Purified Virions_1-14306-21925_21925	622	633
TRUE	0.0187	(R)ALTGIAVEQDKNTQEVFAQVK(Q)	97%	4.1310	0.8809		763.7421	2288.2046	3	0.0031	1.3460	5,448.28	2	A0A7L8Z0C9,A0A7D5XMN0	189,807	4_Inactivated_PNGase-F_Treated_Purified Virions_1-21581-30536_30536	753	773
TRUE	0.0073	(R)SFIEDLLFNK(V)	91%	2.9527	0.7587		613.3266	1224.6386	2	-0.0005	-0.4278	6,581.94	4	A0A7L8AB65,A0A7L8Z0C9,A0A7G3UUL1,A0A7L9F8J2	662,570	4_Inactivated_PNGase-F_Treated_Purified Virions_2-24891-36198_36198	803	812
TRUE	0.0185	(R)SFIEDLLFNKVTLADAGFIK(Q)	91%	0.8539	0.5289		747.7430	2240.2071	3	-0.0025	-1.0960	6,726.47	2	A0A7L8Z0C9,A0A7D5XMN0	41,962	4_Inactivated_PNGase-F_Treated_Purified Virions_2-25754-37166_37166	803	822
TRUE	0.0067	(K)QYGDcLGDIAAR(D)	93%	2.7680	0.8205	Carbamidomethyl (+57)	669.8095	1337.6045	2	0.0008	0.6205	4,439.36	5	A0A7L8AB65,A0A7L8Z0C9,A0A7D7LL25,A0A7G3UUL1,A0A7L9F8J2	679,192	4_Inactivated_PNGase-F_Treated_Purified Virions_1-15892-23883_23883	823	834
TRUE	0.0074	(K)LIANQFNSAIGK(I)	93%	1.6266	0.5534		638.3572	1274.6999	2	0.0016	1.2190	4,603.87	4	A0A7L8AB65,A0A7L8Z0C9,A0A7G3UUL1,A0A7L9F8J2	102,905	4_Inactivated_PNGase-F_Treated_Purified Virions_1-16826-24976_24976	909	920
TRUE	0.0074	(K)LIANQFNSAIGK(I)	91%	2.5254	0.7359		638.3560	1274.6974	2	-0.0010	-0.7906	4,598.59	4	A0A7L8AB65,A0A7L8Z0C9,A0A7G3UUL1,A0A7L9F8J2	109,265	4_Inactivated_PNGase-F_Treated_Purified Virions_2-13766-23217_23217	909	920
TRUE	0.0093	(K)IQDSLSSTASALGK(L)	93%	2.8668	0.8227		689.3619	1376.7093	2	-0.0055	-4.0250	4,411.14	3	A0A7L8AB65,A0A7L8Z0C9,A0A7L9F8J2	174,426	4_Inactivated_PNGase-F_Treated_Purified Virions_1-15737-23698_23698	921	934
TRUE	0.0075	(K)LQDVVNQNAQALNTLVK(Q)	93%	2.6329	0.8366		934.5165	1867.0185	2	0.0021	1.1020	5,475.00	3	A0A7L8Z0C9,A0A7D7LL25,A0A7L9F8J2	337,109	4_Inactivated_PNGase-F_Treated_Purified Virions_1-21736-30714_30714	935	951
TRUE	0.0075	(K)LQDVVNQNAQALNTLVK(Q)	91%	1.1296	0.6600		934.5166	1867.0187	2	0.0022	1.1670	5,471.10	3	A0A7L8Z0C9,A0A7D7LL25,A0A7L9F8J2	86,132	4_Inactivated_PNGase-F_Treated_Purified Virions_2-18512-28880_28880	935	951
TRUE	0.0073	(R)LQSLQTYVTQQLIR(A)	93%	3.5543	0.8021		845.9778	1689.9410	2	-0.0006	-0.3289	6,235.25	3	A0A7L8AB65,A0A7L8Z0C9,A0A7G3UUL1	761,881	4_Inactivated_PNGase-F_Treated_Purified Virions_1-26215-35782_35782	988	1001
TRUE	0.0077	(R)ASANLAATK(M)	93%	2.3295	0.5915		423.7382	845.4619	2	0.0012	1.4390	2,315.54	3	A0A7L8AB65,A0A7L8Z0C9,A0A7G3UUL1	269,357	4_Inactivated_PNGase-F_Treated_Purified Virions_1-5334-10662_10662	1007	1015
TRUE	0.0077	(R)ASANLAATK(M)	91%	1.8353	0.5742		423.7386	845.4626	2	0.0019	2.2320	2,195.42	3	A0A7L8AB65,A0A7L8Z0C9,A0A7G3UUL1	220,430	4_Inactivated_PNGase-F_Treated_Purified Virions_2-3405-9284_9284	1007	1015
TRUE	0.0079	(K)RVDFcGK(G)	90%	2.2057	0.6023	Carbamidomethyl (+57)	441.2182	880.4218	2	-0.0008	-0.9616	2,607.16	3	A0A7L8AB65,A0A7L8Z0C9,A0A7G3UUL1	437,414	4_Inactivated_PNGase-F_Treated_Purified Virions_2-4752-11405_11405	1026	1032
**Surface glycoprotein OS=Severe acute respiratory syndrome coronavirus 2 OX=2697049 GN=S PE=4 SV=1 Accession A0A7L8Z0C9**																		
TRUE	0.0630	(R)TQLPPAYTNSFTR(G)	93%	2.1875	0.7608		748.3802	1494.7459	2	-0.0008	-0.5513	4,627.22	5	A0A7L8AB65,A0A7D7LL25,A0A7G3UUL1,A0A7L9W8W9_NDM,A0A7L9F8J2	267,798	4_Inactivated_PNGase-F_Treated_Purified Virions_1-16963-25132_25132	22	34
TRUE	0.0708	(R)GWIFGTTLDSK(T)	93%	2.5846	0.8111		612.8164	1223.6183	2	-0.0006	-0.4982	6,025.31	4	A0A7L8AB65,A0A7D7LL25,A0A7G3UUL1,A0A7L9W8W9_NDM	262,603	4_Inactivated_PNGase-F_Treated_Purified Virions_1-24989-34390_34390	103	113
TRUE	0.0708	(R)GWIFGTTLDSK(T)	91%	1.8062	0.7146		612.8172	1223.6198	2	0.0010	0.7976	6,017.86	4	A0A7L8AB65,A0A7D7LL25,A0A7G3UUL1,A0A7L9W8W9_NDM	157,260	4_Inactivated_PNGase-F_Treated_Purified Virions_2-21609-32468_32468	103	113
TRUE	0.1908	(K)VcEFQFcNDPFLGVYYHK(N)	98%	1.8994	0.6971	Carbamidomethyl (+57), Carbamidomethyl (+57)	775.0149	2322.0228	3	-0.0011	-0.4529	6,349.75	2	A0A7D5XMN0,A0A7L9W8W9_NDM	250,005	4_Inactivated_PNGase-F_Treated_Purified Virions_2-23511-34649_34649	130	147
TRUE	0.0689	(K)SWMESEFR(V)	93%	1.6519	0.9152		536.2332	1070.4519	2	0.0028	2.6210	4,979.05	4	A0A7L8AB65,A0A7D7LL25,A0A7G3UUL1,A0A7L9W8W9_NDM	305,930	4_Inactivated_PNGase-F_Treated_Purified Virions_1-18929-27453_27453	151	158
TRUE	0.0882	(K)NIDGYFK(I)	90%	1.4635	0.6497		428.7134	855.4123	2	-0.0005	-0.5882	4,276.05	3	A0A7L8AB65,A0A7L9W8W9_NDM,A0A7L9F8J2	1,060,970	4_Inactivated_PNGase-F_Treated_Purified Virions_1-15057-22852_22852	196	202
TRUE	0.0882	(K)NIDGYFK(I)	90%	1.4136	0.6404		428.7139	855.4132	2	0.0005	0.5520	4,271.92	3	A0A7L8AB65,A0A7L9W8W9_NDM,A0A7L9F8J2	752,161	4_Inactivated_PNGase-F_Treated_Purified Virions_2-12019-21119_21119	196	202
TRUE	0.1024	(K)HTPINLVR(D)	93%	1.9083	0.7661		475.2825	948.5504	2	-0.0002	-0.1909	3,588.61	3	A0A7L8AB65,A0A7L9W8W9_NDM,A0A7L9F8J2	696,137	4_Inactivated_PNGase-F_Treated_Purified Virions_1-11636-18571_18571	207	214
TRUE	0.1024	(K)HTPINLVR(D)	91%	1.8660	0.7101		475.2829	948.5512	2	0.0006	0.6448	3,565.99	3	A0A7L8AB65,A0A7L9W8W9_NDM,A0A7L9F8J2	447,323	4_Inactivated_PNGase-F_Treated_Purified Virions_2-8665-16834_16834	207	214

Identified Spike Peptides

Valid	Weight	Sequence [a,b]	Protein identification probability (Prob)	SEQUEST XCorr	SEQUEST deltaCn	Modifications	Observed Mass (Da)	Actual Mass (Da)	Charge	Delta Da	Delta PPM	Retention Time	# Other Proteins	Other Proteins	Total Ion Chromatogram (TIC)	Spectrum ID	Peptide Start Index	Peptide Stop Index
TRUE	0.0690	(R)FQTLLALHR(S)	98%	1.7411	0.5229		366.8858	1097.6355	3	0.0008	0.7453	4,992.36	5	A0A7L8AB65,A0A7D7LL25,A0A7G3UUL1,A0A7L9W8W9_NDM,A0A7L9F8J2	149,623	4_Inactivated_PNGase-F_Treated_Purified Virions_2-15930-25794_25794	238	246
TRUE	0.0690	(R)FQTLLALHR(S)	97%	1.5288	0.6154		366.8853	1097.6340	3	-0.0006	-0.5880	4,994.13	5	A0A7L8AB65,A0A7D7LL25,A0A7G3UUL1,A0A7L9W8W9_NDM,A0A7L9F8J2	139,042	4_Inactivated_PNGase-F_Treated_Purified Virions_1-19022-27555_27555	238	246
TRUE	0.0646	(K)GIYQTSNFR(V)	93%	2.0250	0.7860		543.2731	1084.5317	2	0.0015	1.4210	3,841.61	4	A0A7D7LL25,A0A7G3UUL1,A0A7L9W8W9_NDM,A0A7L9F8J2	925,100	4_Inactivated_PNGase-F_Treated_Purified Virions_1-12760-20062_20062	311	319
TRUE	0.0648	(R)VQPTESIVR(F)	92%	1.4220	0.5163		514.7902	1027.5659	2	-0.0004	-0.3678	3,419.23	4	A0A7D7LL25,A0A7G3UUL1,A0A7L9W8W9_NDM,A0A7L9F8J2	168,668	4_Inactivated_PNGase-F_Treated_Purified Virions_1-10778-17506_17506	320	328
TRUE	0.0648	(R)VQPTESIVR(F)	91%	1.5907	0.5241		514.7902	1027.5658	2	-0.0005	-0.4865	3,413.18	4	A0A7D7LL25,A0A7G3UUL1,A0A7L9W8W9_NDM,A0A7L9F8J2	203,935	4_Inactivated_PNGase-F_Treated_Purified Virions_2-7953-15912_15912	320	328
TRUE	0.0648	(R)VQPTESIVR(F)	91%	1.3860	0.5171		514.7902	1027.5659	2	-0.0004	-0.3678	3,392.50	4	A0A7D7LL25,A0A7G3UUL1,A0A7L9W8W9_NDM,A0A7L9F8J2	123,342	4_Inactivated_PNGase-F_Treated_Purified Virions_2-7852-15781_15781	320	328
TRUE	0.0873	(R)FASVYAWNR(K)	93%	2.7088	0.8889		557.2787	1112.5428	2	0.0027	2.3800	4,874.98	2	A0A7G3UUL1,A0A7L9W8W9_NDM	317,612	4_Inactivated_PNGase-F_Treated_Purified Virions_1-18333-26758_26758	347	355
TRUE	0.0873	(R)FASVYAWNR(K)	91%	2.3036	0.8589		557.2786	1112.5426	2	0.0024	2.1610	4,876.47	2	A0A7G3UUL1,A0A7L9W8W9_NDM	303,701	4_Inactivated_PNGase-F_Treated_Purified Virions_2-15278-25026_25026	347	355
TRUE	0.0868	(K)cYGVSPTK(L)	93%	1.5966	0.8148	Carbamidomethyl (+57)	456.2192	910.4238	2	0.0019	2.0480	2,779.32	2	A0A7G3UUL1,A0A7L9W8W9_NDM	149,312	4_Inactivated_PNGase-F_Treated_Purified Virions_1-7612-13531_13531	379	386
TRUE	0.0868	(K)cYGVSPTK(L)	91%	1.7819	0.7251	Carbamidomethyl (+57)	456.2192	910.4238	2	0.0019	2.0480	2,729.68	2	A0A7G3UUL1,A0A7L9W8W9_NDM	148,769	4_Inactivated_PNGase-F_Treated_Purified Virions_2-5200-12067_12067	379	386
TRUE	0.0664	(K)IADYNYK(L)	90%	1.8556	0.8586		443.7212	885.4278	2	0.0045	5.0760	3,103.10	4	A0A7L8AB65,A0A7D7LL25,A0A7G3UUL1,A0A7L9W8W9_NDM	1,115,660	4_Inactivated_PNGase-F_Treated_Purified Virions_1-9272-15572_15572	418	424
TRUE	0.0664	(K)IADYNYK(L)	90%	1.6961	0.7985		443.7201	885.4256	2	0.0024	2.6660	3,075.25	4	A0A7L8AB65,A0A7D7LL25,A0A7G3UUL1,A0A7L9W8W9_NDM	823,219	4_Inactivated_PNGase-F_Treated_Purified Virions_2-6530-13962_13962	418	424
TRUE	0.1155	(K)IADYNYKLPDDFTGcVIAWNSNNLDSK(V)	98%	1.8445	0.8513	Carbamidomethyl (+57)	1045.1608	3132.4605	3	0.0103	3.2980	6,351.82	2	A0A7D7XU89,A0A7L9W8W9_NDM	53,111	4_Inactivated_PNGase-F_Treated_Purified Virions_2-23524-34663_34663	418	444
TRUE	0.1155	(K)IADYNYKLPDDFTGcVIAWNSNNLDSK(V)	97%	2.1637	0.8766	Carbamidomethyl (+57)	1045.1584	3132.4535	3	0.0034	1.0780	6,356.32	2	A0A7D7XU89,A0A7L9W8W9_NDM	56,920	4_Inactivated_PNGase-F_Treated_Purified Virions_1-26917-36583_36583	418	444
TRUE	0.1415	(K)LPDDFTGcVIAWNSNNLDSK(V)	93%	4.3011	0.9432	Carbamidomethyl (+57)	1133.5265	2265.0384	2	0.0010	0.4371	6,181.58	2	A0A7D5XMN0,A0A7L9W8W9_NDM	261,016	4_Inactivated_PNGase-F_Treated_Purified Virions_1-25887-35420_35420	425	444
TRUE	0.1415	(K)LPDDFTGcVIAWNSNNLDSK(V)	91%	2.4746	0.9400	Carbamidomethyl (+57)	1133.5221	2265.0296	2	-0.0078	-3.4410	6,174.66	2	A0A7D5XMN0,A0A7L9W8W9_NDM	97,202	4_Inactivated_PNGase-F_Treated_Purified Virions_2-22491-33490_33490	425	444
TRUE	0.0684	(K)VGGNYNYLYR(L)	93%	3.5527	0.8049		609.7979	1217.5812	2	-0.0017	-1.4110	4,354.07	4	A0A7L8AB65,A0A7D7LL25,A0A7L9W8W9_NDM,A0A7L9F8J2	622,786	4_Inactivated_PNGase-F_Treated_Purified Virions_1-15451-23342_23342	445	454
TRUE	0.0684	(K)VGGNYNYLYR(L)	91%	2.2351	0.6696		609.7984	1217.5822	2	-0.0006	-0.5095	4,346.59	4	A0A7L8AB65,A0A7D7LL25,A0A7L9W8W9_NDM,A0A7L9F8J2	250,654	4_Inactivated_PNGase-F_Treated_Purified Virions_2-12388-21577_21577	445	454
TRUE	0.0577	(R)VVVLSFELLHAPATVcGPK(K)	98%	4.5895	0.8170	Carbamidomethyl (+57)	679.7122	2036.1146	3	0.0016	0.7904	6,368.86	3	A0A7D7LL25,A0A7L9W8W9_NDM,A0A7L9F8J2	556,905	4_Inactivated_PNGase-F_Treated_Purified Virions_2-23627-34777_34777	510	528
TRUE	0.0577	(R)VVVLSFELLHAPATVcGPK(K)	97%	4.3779	0.8383	Carbamidomethyl (+57)	679.7113	2036.1121	3	-0.0010	-0.4680	6,370.91	3	A0A7D7LL25,A0A7L9W8W9_NDM,A0A7L9F8J2	651,551	4_Inactivated_PNGase-F_Treated_Purified Virions_1-27005-36681_36681	510	528
TRUE	0.0755	(K)FLPFQQFGR(D)	93%	1.6718	0.8265		570.3042	1138.5938	2	0.0014	1.2070	5,889.86	3	A0A7L8AB65,A0A7G3UUL1,A0A7L9W8W9_NDM	453,594	4_Inactivated_PNGase-F_Treated_Purified Virions_1-24179-33483_33483	559	567
TRUE	0.0755	(K)FLPFQQFGR(D)	93%	1.6218	0.7426		570.2994	1138.5842	2	-0.0083	-7.2550	5,869.08	3	A0A7L8AB65,A0A7G3UUL1,A0A7L9W8W9_NDM	476,881	4_Inactivated_PNGase-F_Treated_Purified Virions_1-24057-33344_33344	559	567
TRUE	0.0755	(K)FLPFQQFGR(D)	91%	1.2733	0.8785		570.3035	1138.5925	2	0.0000	0.0290	5,885.88	3	A0A7L8AB65,A0A7G3UUL1,A0A7L9W8W9_NDM	386,237	4_Inactivated_PNGase-F_Treated_Purified Virions_2-20850-31595_31595	559	567
TRUE	0.0686	(R)VYSTGSNVFQTR(A)	93%	3.2350	0.8099		679.8387	1357.6629	2	0.0004	0.2706	4,129.45	4	A0A7L8AB65,A0A7G3UUL1,A0A7L9W8W9_NDM,A0A7L9F8J2	732,431	4_Inactivated_PNGase-F_Treated_Purified Virions_1-14306-21925_21925	635	646
TRUE	0.1857	(R)ALTGIAVEQDKNTQEVFAQVK(Q)	97%	4.1310	0.8809		763.7421	2288.2046	3	0.0031	1.3460	5,448.28	2	A0A7D5XMN0,A0A7L9W8W9_NDM	189,807	4_Inactivated_PNGase-F_Treated_Purified Virions_1-21581-30536_30536	766	786
TRUE	0.0723	(R)SFIEDLLFNK(V)	91%	2.9527	0.7587		613.3266	1224.6386	2	-0.0005	-0.4278	6,581.94	4	A0A7L8AB65,A0A7G3UUL1,A0A7L9W8W9_NDM,A0A7L9F8J2	662,570	4_Inactivated_PNGase-F_Treated_Purified Virions_2-24891-36198_36198	816	825
TRUE	0.1841	(R)SFIEDLLFNKVTLADAGFIK(Q)	91%	0.8539	0.5289		747.7430	2240.2071	3	-0.0025	-1.0960	6,726.47	2	A0A7D5XMN0,A0A7L9W8W9_NDM	41,962	4_Inactivated_PNGase-F_Treated_Purified Virions_2-25754-37166_37166	816	835
TRUE	0.0664	(K)QYGDcLGDIAAR(D)	93%	2.7680	0.8205	Carbamidomethyl (+57)	669.8095	1337.6045	2	0.0008	0.6205	4,439.36	5	A0A7L8AB65,A0A7D7LL25,A0A7G3UUL1,A0A7L9W8W9_NDM,A0A7L9F8J2	679,192	4_Inactivated_PNGase-F_Treated_Purified Virions_1-15892-23883_23883	836	847
TRUE	0.0736	(K)LIANQFNSAIGK(I)	93%	1.6266	0.5534		638.3572	1274.6999	2	0.0016	1.2190	4,603.87	4	A0A7L8AB65,A0A7G3UUL1,A0A7L9W8W9_NDM,A0A7L9F8J2	102,905	4_Inactivated_PNGase-F_Treated_Purified Virions_1-16826-24976_24976	922	933
TRUE	0.0736	(K)LIANQFNSAIGK(I)	91%	2.5254	0.7359		638.3560	1274.6974	2	-0.0010	-0.7906	4,598.59	4	A0A7L8AB65,A0A7G3UUL1,A0A7L9W8W9_NDM,A0A7L9F8J2	109,265	4_Inactivated_PNGase-F_Treated_Purified Virions_2-13766-23217_23217	922	933
TRUE	0.0921	(K)IQDSLSSTASALGK(L)	93%	2.8668	0.8227		689.3619	1376.7093	2	-0.0055	-4.0250	4,411.14	3	A0A7L8AB65,A0A7L9W8W9_NDM,A0A7L9F8J2	174,426	4_Inactivated_PNGase-F_Treated_Purified Virions_1-15737-23698_23698	934	947
TRUE	0.0748	(K)LQDVVNQNAQALNTLVK(Q)	93%	2.6329	0.8366		934.5165	1867.0185	2	0.0021	1.1020	5,475.00	3	A0A7D7LL25,A0A7L9W8W9_NDM,A0A7L9F8J2	337,109	4_Inactivated_PNGase-F_Treated_Purified Virions_1-21736-30714_30714	948	964
TRUE	0.0748	(K)LQDVVNQNAQALNTLVK(Q)	91%	1.1296	0.6600		934.5166	1867.0187	2	0.0022	1.1670	5,471.10	3	A0A7D7LL25,A0A7L9W8W9_NDM,A0A7L9F8J2	86,132	4_Inactivated_PNGase-F_Treated_Purified Virions_2-18512-28880_28880	948	964
TRUE	0.0728	(R)LQSLQTYVTQQLIR(A)	93%	3.5543	0.8021		845.9778	1689.9410	2	-0.0006	-0.3289	6,235.25	3	A0A7L8AB65,A0A7G3UUL1,A0A7L9W8W9_NDM	761,881	4_Inactivated_PNGase-F_Treated_Purified Virions_1-26215-35782_35782	1001	1014
TRUE	0.0767	(R)ASANLAATK(M)	93%	2.3295	0.5915		423.7382	845.4619	2	0.0012	1.4390	2,315.54	3	A0A7L8AB65,A0A7G3UUL1,A0A7L9W8W9_NDM	269,357	4_Inactivated_PNGase-F_Treated_Purified Virions_1-5334-10662_10662	1020	1028
TRUE	0.0767	(R)ASANLAATK(M)	91%	1.8353	0.5742		423.7386	845.4626	2	0.0019	2.2320	2,195.42	3	A0A7L8AB65,A0A7G3UUL1,A0A7L9W8W9_NDM	220,430	4_Inactivated_PNGase-F_Treated_Purified Virions_2-3405-9284_9284	1020	1028
TRUE	0.0787	(K)RVDFcGK(G)	90%	2.2057	0.6023	Carbamidomethyl (+57)	441.2182	880.4218	2	-0.0008	-0.9616	2,607.16	3	A0A7L8AB65,A0A7G3UUL1,A0A7L9W8W9_NDM	437,414	4_Inactivated_PNGase-F_Treated_Purified Virions_2-4752-11405_11405	1039	1045
**Surface glycoprotein OS=Severe acute respiratory syndrome coronavirus 2 OX=2697049 GN=S PE=4 SV=1 A0A7L8AB65**																		
TRUE	0.1430	(R)TQLPPAYTNSFTR(G)	93%	2.1875	0.7608		748.3802	1494.7459	2	-0.0008	-0.5513	4,627.22	5	A0A7L8Z0C9,A0A7D7LL25,A0A7G3UUL1,A0A7L9W8W9_NDM,A0A7L9F8J2	267,798	4_Inactivated_PNGase-F_Treated_Purified Virions_1-16963-25132_25132	22	34
TRUE	0.1608	(R)GWIFGTTLDSK(T)	93%	2.5846	0.8111		612.8164	1223.6183	2	-0.0006	-0.4982	6,025.31	4	A0A7L8Z0C9,A0A7D7LL25,A0A7G3UUL1,A0A7L9W8W9_NDM	262,603	4_Inactivated_PNGase-F_Treated_Purified Virions_1-24989-34390_34390	103	113
TRUE	0.1608	(R)GWIFGTTLDSK(T)	91%	1.8062	0.7146		612.8172	1223.6198	2	0.0010	0.7976	6,017.86	4	A0A7L8Z0C9,A0A7D7LL25,A0A7G3UUL1,A0A7L9W8W9_NDM	157,260	4_Inactivated_PNGase-F_Treated_Purified Virions_2-21609-32468_32468	103	113
TRUE	0.1565	(K)SWMESEFR(V)	93%	1.6519	0.9152		536.2332	1070.4519	2	0.0028	2.6210	4,979.05	4	A0A7L8Z0C9,A0A7D7LL25,A0A7G3UUL1,A0A7L9W8W9_NDM	305,930	4_Inactivated_PNGase-F_Treated_Purified Virions_1-18929-27453_27453	151	158
TRUE	0.2003	(K)NIDGYFK(I)	90%	1.4635	0.6497		428.7134	855.4123	2	-0.0005	-0.5882	4,276.05	3	A0A7L8Z0C9,A0A7L9W8W9_NDM,A0A7L9F8J2	1,060,970	4_Inactivated_PNGase-F_Treated_Purified Virions_1-15057-22852_22852	196	202
TRUE	0.2003	(K)NIDGYFK(I)	90%	1.4136	0.6404		428.7139	855.4132	2	0.0005	0.5520	4,271.92	3	A0A7L8Z0C9,A0A7L9W8W9_NDM,A0A7L9F8J2	752,161	4_Inactivated_PNGase-F_Treated_Purified Virions_2-12019-21119_21119	196	202
TRUE	0.2325	(K)HTPINLVR(D)	93%	1.9083	0.7661		475.2825	948.5504	2	-0.0002	-0.1909	3,588.61	3	A0A7L8Z0C9,A0A7L9W8W9_NDM,A0A7L9F8J2	696,137	4_Inactivated_PNGase-F_Treated_Purified Virions_1-11636-18571_18571	207	214
TRUE	0.2325	(K)HTPINLVR(D)	91%	1.8660	0.7101		475.2829	948.5512	2	0.0006	0.6448	3,565.99	3	A0A7L8Z0C9,A0A7L9W8W9_NDM,A0A7L9F8J2	447,323	4_Inactivated_PNGase-F_Treated_Purified Virions_2-8665-16834_16834	207	214
TRUE	0.1567	(R)FQTLLALHR(S)	98%	1.7411	0.5229		366.8858	1097.6355	3	0.0008	0.7453	4,992.36	5	A0A7L8Z0C9,A0A7D7LL25,A0A7G3UUL1,A0A7L9W8W9_NDM,A0A7L9F8J2	149,623	4_Inactivated_PNGase-F_Treated_Purified Virions_2-15930-25794_25794	238	246
TRUE	0.1567	(R)FQTLLALHR(S)	97%	1.5288	0.6154		366.8853	1097.6340	3	-0.0006	-0.5880	4,994.13	5	A0A7L8Z0C9,A0A7D7LL25,A0A7G3UUL1,A0A7L9W8W9_NDM,A0A7L9F8J2	139,042	4_Inactivated_PNGase-F_Treated_Purified Virions_1-19022-27555_27555	238	246
TRUE	0.1507	(K)IADYNYK(L)	90%	1.8556	0.8586		443.7212	885.4278	2	0.0045	5.0760	3,103.10	4	A0A7L8Z0C9,A0A7D7LL25,A0A7G3UUL1,A0A7L9W8W9_NDM	1,115,660	4_Inactivated_PNGase-F_Treated_Purified Virions_1-9272-15572_15572	418	424
TRUE	0.1507	(K)IADYNYK(L)	90%	1.6961	0.7985		443.7201	885.4256	2	0.0024	2.6660	3,075.25	4	A0A7L8Z0C9,A0A7D7LL25,A0A7G3UUL1,A0A7L9W8W9_NDM	823,219	4_Inactivated_PNGase-F_Treated_Purified Virions_2-6530-13962_13962	418	424
TRUE	0.1552	(K)VGGNYNYLYR(L)	93%	3.5527	0.8049		609.7979	1217.5812	2	-0.0017	-1.4110	4,354.07	4	A0A7L8Z0C9,A0A7D7LL25,A0A7L9W8W9_NDM,A0A7L9F8J2	622,786	4_Inactivated_PNGase-F_Treated_Purified Virions_1-15451-23342_23342	445	454
TRUE	0.1552	(K)VGGNYNYLYR(L)	91%	2.2351	0.6696		609.7984	1217.5822	2	-0.0006	-0.5095	4,346.59	4	A0A7L8Z0C9,A0A7D7LL25,A0A7L9W8W9_NDM,A0A7L9F8J2	250,654	4_Inactivated_PNGase-F_Treated_Purified Virions_2-12388-21577_21577	445	454
TRUE	0.1713	(K)FLPFQQFGR(D)	93%	1.6718	0.8265		570.3042	1138.5938	2	0.0014	1.2070	5,889.86	3	A0A7L8Z0C9,A0A7G3UUL1,A0A7L9W8W9_NDM	453,594	4_Inactivated_PNGase-F_Treated_Purified Virions_1-24179-33483_33483	559	567
TRUE	0.1713	(K)FLPFQQFGR(D)	93%	1.6218	0.7426		570.2994	1138.5842	2	-0.0083	-7.2550	5,869.08	3	A0A7L8Z0C9,A0A7G3UUL1,A0A7L9W8W9_NDM	476,881	4_Inactivated_PNGase-F_Treated_Purified Virions_1-24057-33344_33344	559	567
TRUE	0.1713	(K)FLPFQQFGR(D)	91%	1.2733	0.8785		570.3035	1138.5925	2	0.0000	0.0290	5,885.88	3	A0A7L8Z0C9,A0A7G3UUL1,A0A7L9W8W9_NDM	386,237	4_Inactivated_PNGase-F_Treated_Purified Virions_2-20850-31595_31595	559	567
TRUE	0.1557	(R)VYSTGSNVFQTR(A)	93%	3.2350	0.8099		679.8387	1357.6629	2	0.0004	0.2706	4,129.45	4	A0A7L8Z0C9,A0A7G3UUL1,A0A7L9W8W9_NDM,A0A7L9F8J2	732,431	4_Inactivated_PNGase-F_Treated_Purified Virions_1-14306-21925_21925	635	646
TRUE	0.1642	(R)SFIEDLLFNK(V)	91%	2.9527	0.7587		613.3266	1224.6386	2	-0.0005	-0.4278	6,581.94	4	A0A7L8Z0C9,A0A7G3UUL1,A0A7L9W8W9_NDM,A0A7L9F8J2	662,570	4_Inactivated_PNGase-F_Treated_Purified Virions_2-24891-36198_36198	816	825
TRUE	0.1507	(K)QYGDcLGDIAAR(D)	93%	2.7680	0.8205	Carbamidomethyl (+57)	669.8095	1337.6045	2	0.0008	0.6205	4,439.36	5	A0A7L8Z0C9,A0A7D7LL25,A0A7G3UUL1,A0A7L9W8W9_NDM,A0A7L9F8J2	679,192	4_Inactivated_PNGase-F_Treated_Purified Virions_1-15892-23883_23883	836	847
TRUE	0.1670	(K)LIANQFNSAIGK(I)	93%	1.6266	0.5534		638.3572	1274.6999	2	0.0016	1.2190	4,603.87	4	A0A7L8Z0C9,A0A7G3UUL1,A0A7L9W8W9_NDM,A0A7L9F8J2	102,905	4_Inactivated_PNGase-F_Treated_Purified Virions_1-16826-24976_24976	922	933
TRUE	0.1670	(K)LIANQFNSAIGK(I)	91%	2.5254	0.7359		638.3560	1274.6974	2	-0.0010	-0.7906	4,598.59	4	A0A7L8Z0C9,A0A7G3UUL1,A0A7L9W8W9_NDM,A0A7L9F8J2	109,265	4_Inactivated_PNGase-F_Treated_Purified Virions_2-13766-23217_23217	922	933
TRUE	0.2091	(K)IQDSLSSTASALGK(L)	93%	2.8668	0.8227		689.3619	1376.7093	2	-0.0055	-4.0250	4,411.14	3	A0A7L8Z0C9,A0A7L9W8W9_NDM,A0A7L9F8J2	174,426	4_Inactivated_PNGase-F_Treated_Purified Virions_1-15737-23698_23698	934	947
TRUE	0.1652	(R)LQSLQTYVTQQLIR(A)	93%	3.5543	0.8021		845.9778	1689.9410	2	-0.0006	-0.3289	6,235.25	3	A0A7L8Z0C9,A0A7G3UUL1,A0A7L9W8W9_NDM	761,881	4_Inactivated_PNGase-F_Treated_Purified Virions_1-26215-35782_35782	1001	1014
TRUE	0.1741	(R)ASANLAATK(M)	93%	2.3295	0.5915		423.7382	845.4619	2	0.0012	1.4390	2,315.54	3	A0A7L8Z0C9,A0A7G3UUL1,A0A7L9W8W9_NDM	269,357	4_Inactivated_PNGase-F_Treated_Purified Virions_1-5334-10662_10662	1020	1028
TRUE	0.1741	(R)ASANLAATK(M)	91%	1.8353	0.5742		423.7386	845.4626	2	0.0019	2.2320	2,195.42	3	A0A7L8Z0C9,A0A7G3UUL1,A0A7L9W8W9_NDM	220,430	4_Inactivated_PNGase-F_Treated_Purified Virions_2-3405-9284_9284	1020	1028
TRUE	0.1786	(K)RVDFcGK(G)	90%	2.2057	0.6023	Carbamidomethyl (+57)	441.2182	880.4218	2	-0.0008	-0.9616	2,607.16	3	A0A7L8Z0C9,A0A7G3UUL1,A0A7L9W8W9_NDM	437,414	4_Inactivated_PNGase-F_Treated_Purified Virions_2-4752-11405_11405	1039	1045
**Spike glycoprotein OS=Severe acute respiratory syndrome coronavirus 2 OX=2697049 GN=S PE=3 SV=1 A0A7G3UUL1**																		
TRUE	0.1541	(R)TQLPPAYTNSFTR(G)	93%	2.1875	0.7608		748.3802	1494.7459	2	-0.0008	-0.5513	4,627.22	5	A0A7L8AB65,A0A7L8Z0C9,A0A7D7LL25,A0A7L9W8W9_NDM,A0A7L9F8J2	267,798	4_Inactivated_PNGase-F_Treated_Purified Virions_1-16963-25132_25132	22	34
TRUE	0.1733	(R)GWIFGTTLDSK(T)	93%	2.5846	0.8111		612.8164	1223.6183	2	-0.0006	-0.4982	6,025.31	4	A0A7L8AB65,A0A7L8Z0C9,A0A7D7LL25,A0A7L9W8W9_NDM	262,603	4_Inactivated_PNGase-F_Treated_Purified Virions_1-24989-34390_34390	103	113
TRUE	0.1733	(R)GWIFGTTLDSK(T)	91%	1.8062	0.7146		612.8172	1223.6198	2	0.0010	0.7976	6,017.86	4	A0A7L8AB65,A0A7L8Z0C9,A0A7D7LL25,A0A7L9W8W9_NDM	157,260	4_Inactivated_PNGase-F_Treated_Purified Virions_2-21609-32468_32468	103	113
TRUE	0.1686	(K)SWMESEFR(V)	93%	1.6519	0.9152		536.2332	1070.4519	2	0.0028	2.6210	4,979.05	4	A0A7L8AB65,A0A7L8Z0C9,A0A7D7LL25,A0A7L9W8W9_NDM	305,930	4_Inactivated_PNGase-F_Treated_Purified Virions_1-18929-27453_27453	151	158
TRUE	0.1688	(R)FQTLLALHR(S)	98%	1.7411	0.5229		366.8858	1097.6355	3	0.0008	0.7453	4,992.36	5	A0A7L8AB65,A0A7L8Z0C9,A0A7D7LL25,A0A7L9W8W9_NDM,A0A7L9F8J2	149,623	4_Inactivated_PNGase-F_Treated_Purified Virions_2-15930-25794_25794	238	246
TRUE	0.1688	(R)FQTLLALHR(S)	97%	1.5288	0.6154		366.8853	1097.6340	3	-0.0006	-0.5880	4,994.13	5	A0A7L8AB65,A0A7L8Z0C9,A0A7D7LL25,A0A7L9W8W9_NDM,A0A7L9F8J2	139,042	4_Inactivated_PNGase-F_Treated_Purified Virions_1-19022-27555_27555	238	246
TRUE	0.1580	(K)GIYQTSNFR(V)	93%	2.0250	0.7860		543.2731	1084.5317	2	0.0015	1.4210	3,841.61	4	A0A7L8Z0C9,A0A7D7LL25,A0A7L9W8W9_NDM,A0A7L9F8J2	925,100	4_Inactivated_PNGase-F_Treated_Purified Virions_1-12760-20062_20062	311	319
TRUE	0.1585	(R)VQPTESIVR(F)	92%	1.4220	0.5163		514.7902	1027.5659	2	-0.0004	-0.3678	3,419.23	4	A0A7L8Z0C9,A0A7D7LL25,A0A7L9W8W9_NDM,A0A7L9F8J2	168,668	4_Inactivated_PNGase-F_Treated_Purified Virions_1-10778-17506_17506	320	328
TRUE	0.1585	(R)VQPTESIVR(F)	91%	1.5907	0.5241		514.7902	1027.5658	2	-0.0005	-0.4865	3,413.18	4	A0A7L8Z0C9,A0A7D7LL25,A0A7L9W8W9_NDM,A0A7L9F8J2	203,935	4_Inactivated_PNGase-F_Treated_Purified Virions_2-7953-15912_15912	320	328
TRUE	0.1585	(R)VQPTESIVR(F)	91%	1.3860	0.5171		514.7902	1027.5659	2	-0.0004	-0.3678	3,392.50	4	A0A7L8Z0C9,A0A7D7LL25,A0A7L9W8W9_NDM,A0A7L9F8J2	123,342	4_Inactivated_PNGase-F_Treated_Purified Virions_2-7852-15781_15781	320	328
TRUE	0.2136	(R)FASVYAWNR(K)	93%	2.7088	0.8889		557.2787	1112.5428	2	0.0027	2.3800	4,874.98	2	A0A7L8Z0C9,A0A7L9W8W9_NDM	317,612	4_Inactivated_PNGase-F_Treated_Purified Virions_1-18333-26758_26758	347	355
TRUE	0.2136	(R)FASVYAWNR(K)	91%	2.3036	0.8589		557.2786	1112.5426	2	0.0024	2.1610	4,876.47	2	A0A7L8Z0C9,A0A7L9W8W9_NDM	303,701	4_Inactivated_PNGase-F_Treated_Purified Virions_2-15278-25026_25026	347	355
TRUE	0.2124	(K)cYGVSPTK(L)	93%	1.5966	0.8148	Carbamidomethyl (+57)	456.2192	910.4238	2	0.0019	2.0480	2,779.32	2	A0A7L8Z0C9,A0A7L9W8W9_NDM	149,312	4_Inactivated_PNGase-F_Treated_Purified Virions_1-7612-13531_13531	379	386
TRUE	0.2124	(K)cYGVSPTK(L)	91%	1.7819	0.7251	Carbamidomethyl (+57)	456.2192	910.4238	2	0.0019	2.0480	2,729.68	2	A0A7L8Z0C9,A0A7L9W8W9_NDM	148,769	4_Inactivated_PNGase-F_Treated_Purified Virions_2-5200-12067_12067	379	386
TRUE	0.1624	(K)IADYNYK(L)	90%	1.8556	0.8586		443.7212	885.4278	2	0.0045	5.0760	3,103.10	4	A0A7L8AB65,A0A7L8Z0C9,A0A7D7LL25,A0A7L9W8W9_NDM	1,115,660	4_Inactivated_PNGase-F_Treated_Purified Virions_1-9272-15572_15572	418	424
TRUE	0.1624	(K)IADYNYK(L)	90%	1.6961	0.7985		443.7201	885.4256	2	0.0024	2.6660	3,075.25	4	A0A7L8AB65,A0A7L8Z0C9,A0A7D7LL25,A0A7L9W8W9_NDM	823,219	4_Inactivated_PNGase-F_Treated_Purified Virions_2-6530-13962_13962	418	424
TRUE	0.1846	(K)FLPFQQFGR(D)	93%	1.6718	0.8265		570.3042	1138.5938	2	0.0014	1.2070	5,889.86	3	A0A7L8AB65,A0A7L8Z0C9,A0A7L9W8W9_NDM	453,594	4_Inactivated_PNGase-F_Treated_Purified Virions_1-24179-33483_33483	559	567
TRUE	0.1846	(K)FLPFQQFGR(D)	93%	1.6218	0.7426		570.2994	1138.5842	2	-0.0083	-7.2550	5,869.08	3	A0A7L8AB65,A0A7L8Z0C9,A0A7L9W8W9_NDM	476,881	4_Inactivated_PNGase-F_Treated_Purified Virions_1-24057-33344_33344	559	567
TRUE	0.1846	(K)FLPFQQFGR(D)	91%	1.2733	0.8785		570.3035	1138.5925	2	0.0000	0.0290	5,885.88	3	A0A7L8AB65,A0A7L8Z0C9,A0A7L9W8W9_NDM	386,237	4_Inactivated_PNGase-F_Treated_Purified Virions_2-20850-31595_31595	559	567
TRUE	0.1678	(R)VYSTGSNVFQTR(A)	93%	3.2350	0.8099		679.8387	1357.6629	2	0.0004	0.2706	4,129.45	4	A0A7L8AB65,A0A7L8Z0C9,A0A7L9W8W9_NDM,A0A7L9F8J2	732,431	4_Inactivated_PNGase-F_Treated_Purified Virions_1-14306-21925_21925	635	646
TRUE	0.1769	(R)SFIEDLLFNK(V)	91%	2.9527	0.7587		613.3266	1224.6386	2	-0.0005	-0.4278	6,581.94	4	A0A7L8AB65,A0A7L8Z0C9,A0A7L9W8W9_NDM,A0A7L9F8J2	662,570	4_Inactivated_PNGase-F_Treated_Purified Virions_2-24891-36198_36198	816	825
TRUE	0.1624	(K)QYGDcLGDIAAR(D)	93%	2.7680	0.8205	Carbamidomethyl (+57)	669.8095	1337.6045	2	0.0008	0.6205	4,439.36	5	A0A7L8AB65,A0A7L8Z0C9,A0A7D7LL25,A0A7L9W8W9_NDM,A0A7L9F8J2	679,192	4_Inactivated_PNGase-F_Treated_Purified Virions_1-15892-23883_23883	836	847
TRUE	0.1800	(K)LIANQFNSAIGK(I)	93%	1.6266	0.5534		638.3572	1274.6999	2	0.0016	1.2190	4,603.87	4	A0A7L8AB65,A0A7L8Z0C9,A0A7L9W8W9_NDM,A0A7L9F8J2	102,905	4_Inactivated_PNGase-F_Treated_Purified Virions_1-16826-24976_24976	922	933
TRUE	0.1800	(K)LIANQFNSAIGK(I)	91%	2.5254	0.7359		638.3560	1274.6974	2	-0.0010	-0.7906	4,598.59	4	A0A7L8AB65,A0A7L8Z0C9,A0A7L9W8W9_NDM,A0A7L9F8J2	109,265	4_Inactivated_PNGase-F_Treated_Purified Virions_2-13766-23217_23217	922	933
TRUE	0.1780	(R)LQSLQTYVTQQLIR(A)	93%	3.5543	0.8021		845.9778	1689.9410	2	-0.0006	-0.3289	6,235.25	3	A0A7L8AB65,A0A7L8Z0C9,A0A7L9W8W9_NDM	761,881	4_Inactivated_PNGase-F_Treated_Purified Virions_1-26215-35782_35782	1001	1014
TRUE	0.1876	(R)ASANLAATK(M)	93%	2.3295	0.5915		423.7382	845.4619	2	0.0012	1.4390	2,315.54	3	A0A7L8AB65,A0A7L8Z0C9,A0A7L9W8W9_NDM	269,357	4_Inactivated_PNGase-F_Treated_Purified Virions_1-5334-10662_10662	1020	1028
TRUE	0.1876	(R)ASANLAATK(M)	91%	1.8353	0.5742		423.7386	845.4626	2	0.0019	2.2320	2,195.42	3	A0A7L8AB65,A0A7L8Z0C9,A0A7L9W8W9_NDM	220,430	4_Inactivated_PNGase-F_Treated_Purified Virions_2-3405-9284_9284	1020	1028
TRUE	0.1924	(K)RVDFcGK(G)	90%	2.2057	0.6023	Carbamidomethyl (+57)	441.2182	880.4218	2	-0.0008	-0.9616	2,607.16	3	A0A7L8AB65,A0A7L8Z0C9,A0A7L9W8W9_NDM	437,414	4_Inactivated_PNGase-F_Treated_Purified Virions_2-4752-11405_11405	1039	1045
**Surface glycoprotein OS=Severe acute respiratory syndrome coronavirus 2 OX=2697049 GN=S PE=4 SV=1 A0A7L9F8J2**																		
TRUE	0.0657	(R)TQLPPAYTNSFTR(G)	93%	2.1875	0.7608		748.3802	1494.7459	2	-0.0008	-0.5513	4,627.22	5	A0A7L8AB65,A0A7L8Z0C9,A0A7D7LL25,A0A7G3UUL1,A0A7L9W8W9_NDM	267,798	4_Inactivated_PNGase-F_Treated_Purified Virions_1-16963-25132_25132	22	34
TRUE	0.0919	(K)NIDGYFK(I)	90%	1.4635	0.6497		428.7134	855.4123	2	-0.0005	-0.5882	4,276.05	3	A0A7L8AB65,A0A7L8Z0C9,A0A7L9W8W9_NDM	1,060,970	4_Inactivated_PNGase-F_Treated_Purified Virions_1-15057-22852_22852	196	202
TRUE	0.0919	(K)NIDGYFK(I)	90%	1.4136	0.6404		428.7139	855.4132	2	0.0005	0.5520	4,271.92	3	A0A7L8AB65,A0A7L8Z0C9,A0A7L9W8W9_NDM	752,161	4_Inactivated_PNGase-F_Treated_Purified Virions_2-12019-21119_21119	196	202
TRUE	0.1067	(K)HTPINLVR(D)	93%	1.9083	0.7661		475.2825	948.5504	2	-0.0002	-0.1909	3,588.61	3	A0A7L8AB65,A0A7L8Z0C9,A0A7L9W8W9_NDM	696,137	4_Inactivated_PNGase-F_Treated_Purified Virions_1-11636-18571_18571	207	214
TRUE	0.1067	(K)HTPINLVR(D)	91%	1.8660	0.7101		475.2829	948.5512	2	0.0006	0.6448	3,565.99	3	A0A7L8AB65,A0A7L8Z0C9,A0A7L9W8W9_NDM	447,323	4_Inactivated_PNGase-F_Treated_Purified Virions_2-8665-16834_16834	207	214
TRUE	0.0719	(R)FQTLLALHR(S)	98%	1.7411	0.5229		366.8858	1097.6355	3	0.0008	0.7453	4,992.36	5	A0A7L8AB65,A0A7L8Z0C9,A0A7D7LL25,A0A7G3UUL1,A0A7L9W8W9_NDM	149,623	4_Inactivated_PNGase-F_Treated_Purified Virions_2-15930-25794_25794	238	246
TRUE	0.0719	(R)FQTLLALHR(S)	97%	1.5288	0.6154		366.8853	1097.6340	3	-0.0006	-0.5880	4,994.13	5	A0A7L8AB65,A0A7L8Z0C9,A0A7D7LL25,A0A7G3UUL1,A0A7L9W8W9_NDM	139,042	4_Inactivated_PNGase-F_Treated_Purified Virions_1-19022-27555_27555	238	246
TRUE	0.0673	(K)GIYQTSNFR(V)	93%	2.0250	0.7860		543.2731	1084.5317	2	0.0015	1.4210	3,841.61	4	A0A7L8Z0C9,A0A7D7LL25,A0A7G3UUL1,A0A7L9W8W9_NDM	925,100	4_Inactivated_PNGase-F_Treated_Purified Virions_1-12760-20062_20062	311	319
TRUE	0.0675	(R)VQPTESIVR(F)	92%	1.4220	0.5163		514.7902	1027.5659	2	-0.0004	-0.3678	3,419.23	4	A0A7L8Z0C9,A0A7D7LL25,A0A7G3UUL1,A0A7L9W8W9_NDM	168,668	4_Inactivated_PNGase-F_Treated_Purified Virions_1-10778-17506_17506	320	328
TRUE	0.0675	(R)VQPTESIVR(F)	91%	1.5907	0.5241		514.7902	1027.5658	2	-0.0005	-0.4865	3,413.18	4	A0A7L8Z0C9,A0A7D7LL25,A0A7G3UUL1,A0A7L9W8W9_NDM	203,935	4_Inactivated_PNGase-F_Treated_Purified Virions_2-7953-15912_15912	320	328
TRUE	0.0675	(R)VQPTESIVR(F)	91%	1.3860	0.5171		514.7902	1027.5659	2	-0.0004	-0.3678	3,392.50	4	A0A7L8Z0C9,A0A7D7LL25,A0A7G3UUL1,A0A7L9W8W9_NDM	123,342	4_Inactivated_PNGase-F_Treated_Purified Virions_2-7852-15781_15781	320	328
TRUE	0.0712	(K)VGGNYNYLYR(L)	93%	3.5527	0.8049		609.7979	1217.5812	2	-0.0017	-1.4110	4,354.07	4	A0A7L8AB65,A0A7L8Z0C9,A0A7D7LL25,A0A7L9W8W9_NDM	622,786	4_Inactivated_PNGase-F_Treated_Purified Virions_1-15451-23342_23342	445	454
TRUE	0.0712	(K)VGGNYNYLYR(L)	91%	2.2351	0.6696		609.7984	1217.5822	2	-0.0006	-0.5095	4,346.59	4	A0A7L8AB65,A0A7L8Z0C9,A0A7D7LL25,A0A7L9W8W9_NDM	250,654	4_Inactivated_PNGase-F_Treated_Purified Virions_2-12388-21577_21577	445	454
TRUE	0.0601	(R)VVVLSFELLHAPATVcGPK(K)	98%	4.5895	0.8170	Carbamidomethyl (+57)	679.7122	2036.1146	3	0.0016	0.7904	6,368.86	3	A0A7L8Z0C9,A0A7D7LL25,A0A7L9W8W9_NDM	556,905	4_Inactivated_PNGase-F_Treated_Purified Virions_2-23627-34777_34777	510	528
TRUE	0.0601	(R)VVVLSFELLHAPATVcGPK(K)	97%	4.3779	0.8383	Carbamidomethyl (+57)	679.7113	2036.1121	3	-0.0010	-0.4680	6,370.91	3	A0A7L8Z0C9,A0A7D7LL25,A0A7L9W8W9_NDM	651,551	4_Inactivated_PNGase-F_Treated_Purified Virions_1-27005-36681_36681	510	528
TRUE	0.0715	(R)VYSTGSNVFQTR(A)	93%	3.2350	0.8099		679.8387	1357.6629	2	0.0004	0.2706	4,129.45	4	A0A7L8AB65,A0A7L8Z0C9,A0A7G3UUL1,A0A7L9W8W9_NDM	732,431	4_Inactivated_PNGase-F_Treated_Purified Virions_1-14306-21925_21925	635	646
TRUE	0.0754	(R)SFIEDLLFNK(V)	91%	2.9527	0.7587		613.3266	1224.6386	2	-0.0005	-0.4278	6,581.94	4	A0A7L8AB65,A0A7L8Z0C9,A0A7G3UUL1,A0A7L9W8W9_NDM	662,570	4_Inactivated_PNGase-F_Treated_Purified Virions_2-24891-36198_36198	816	825
TRUE	0.0692	(K)QYGDcLGDIAAR(D)	93%	2.7680	0.8205	Carbamidomethyl (+57)	669.8095	1337.6045	2	0.0008	0.6205	4,439.36	5	A0A7L8AB65,A0A7L8Z0C9,A0A7D7LL25,A0A7G3UUL1,A0A7L9W8W9_NDM	679,192	4_Inactivated_PNGase-F_Treated_Purified Virions_1-15892-23883_23883	836	847
TRUE	0.0767	(K)LIANQFNSAIGK(I)	93%	1.6266	0.5534		638.3572	1274.6999	2	0.0016	1.2190	4,603.87	4	A0A7L8AB65,A0A7L8Z0C9,A0A7G3UUL1,A0A7L9W8W9_NDM	102,905	4_Inactivated_PNGase-F_Treated_Purified Virions_1-16826-24976_24976	922	933
TRUE	0.0767	(K)LIANQFNSAIGK(I)	91%	2.5254	0.7359		638.3560	1274.6974	2	-0.0010	-0.7906	4,598.59	4	A0A7L8AB65,A0A7L8Z0C9,A0A7G3UUL1,A0A7L9W8W9_NDM	109,265	4_Inactivated_PNGase-F_Treated_Purified Virions_2-13766-23217_23217	922	933
TRUE	0.0960	(K)IQDSLSSTASALGK(L)	93%	2.8668	0.8227		689.3619	1376.7093	2	-0.0055	-4.0250	4,411.14	3	A0A7L8AB65,A0A7L8Z0C9,A0A7L9W8W9_NDM	174,426	4_Inactivated_PNGase-F_Treated_Purified Virions_1-15737-23698_23698	934	947
TRUE	0.0780	(K)LQDVVNQNAQALNTLVK(Q)	93%	2.6329	0.8366		934.5165	1867.0185	2	0.0021	1.1020	5,475.00	3	A0A7L8Z0C9,A0A7D7LL25,A0A7L9W8W9_NDM	337,109	4_Inactivated_PNGase-F_Treated_Purified Virions_1-21736-30714_30714	948	964
TRUE	0.0780	(K)LQDVVNQNAQALNTLVK(Q)	91%	1.1296	0.6600		934.5166	1867.0187	2	0.0022	1.1670	5,471.10	3	A0A7L8Z0C9,A0A7D7LL25,A0A7L9W8W9_NDM	86,132	4_Inactivated_PNGase-F_Treated_Purified Virions_2-18512-28880_28880	948	964
**Spike glycoprotein OS=Severe acute respiratory syndrome coronavirus 2 OX=2697049 GN=S PE=3 SV=1 A0A7D7LL25**																		
TRUE	0.1405	(R)TQLPPAYTNSFTR(G)	93%	2.1875	0.7608		748.3802	1494.7459	2	-0.0008	-0.5513	4,627.22	5	A0A7L8AB65,A0A7L8Z0C9,A0A7G3UUL1,A0A7L9W8W9_NDM,A0A7L9F8J2	267,798	4_Inactivated_PNGase-F_Treated_Purified Virions_1-16963-25132_25132	22	34
TRUE	0.1580	(R)GWIFGTTLDSK(T)	93%	2.5846	0.8111		612.8164	1223.6183	2	-0.0006	-0.4982	6,025.31	4	A0A7L8AB65,A0A7L8Z0C9,A0A7G3UUL1,A0A7L9W8W9_NDM	262,603	4_Inactivated_PNGase-F_Treated_Purified Virions_1-24989-34390_34390	103	113
TRUE	0.1580	(R)GWIFGTTLDSK(T)	91%	1.8062	0.7146		612.8172	1223.6198	2	0.0010	0.7976	6,017.86	4	A0A7L8AB65,A0A7L8Z0C9,A0A7G3UUL1,A0A7L9W8W9_NDM	157,260	4_Inactivated_PNGase-F_Treated_Purified Virions_2-21609-32468_32468	103	113
TRUE	0.1537	(K)SWMESEFR(V)	93%	1.6519	0.9152		536.2332	1070.4519	2	0.0028	2.6210	4,979.05	4	A0A7L8AB65,A0A7L8Z0C9,A0A7G3UUL1,A0A7L9W8W9_NDM	305,930	4_Inactivated_PNGase-F_Treated_Purified Virions_1-18929-27453_27453	151	158
TRUE	0.1539	(R)FQTLLALHR(S)	98%	1.7411	0.5229		366.8858	1097.6355	3	0.0008	0.7453	4,992.36	5	A0A7L8AB65,A0A7L8Z0C9,A0A7G3UUL1,A0A7L9W8W9_NDM,A0A7L9F8J2	149,623	4_Inactivated_PNGase-F_Treated_Purified Virions_2-15930-25794_25794	238	246
TRUE	0.1539	(R)FQTLLALHR(S)	97%	1.5288	0.6154		366.8853	1097.6340	3	-0.0006	-0.5880	4,994.13	5	A0A7L8AB65,A0A7L8Z0C9,A0A7G3UUL1,A0A7L9W8W9_NDM,A0A7L9F8J2	139,042	4_Inactivated_PNGase-F_Treated_Purified Virions_1-19022-27555_27555	238	246
TRUE	0.1441	(K)GIYQTSNFR(V)	93%	2.0250	0.7860		543.2731	1084.5317	2	0.0015	1.4210	3,841.61	4	A0A7L8Z0C9,A0A7G3UUL1,A0A7L9W8W9_NDM,A0A7L9F8J2	925,100	4_Inactivated_PNGase-F_Treated_Purified Virions_1-12760-20062_20062	311	319
TRUE	0.1446	(R)VQPTESIVR(F)	92%	1.4220	0.5163		514.7902	1027.5659	2	-0.0004	-0.3678	3,419.23	4	A0A7L8Z0C9,A0A7G3UUL1,A0A7L9W8W9_NDM,A0A7L9F8J2	168,668	4_Inactivated_PNGase-F_Treated_Purified Virions_1-10778-17506_17506	320	328
TRUE	0.1446	(R)VQPTESIVR(F)	91%	1.5907	0.5241		514.7902	1027.5658	2	-0.0005	-0.4865	3,413.18	4	A0A7L8Z0C9,A0A7G3UUL1,A0A7L9W8W9_NDM,A0A7L9F8J2	203,935	4_Inactivated_PNGase-F_Treated_Purified Virions_2-7953-15912_15912	320	328
TRUE	0.1446	(R)VQPTESIVR(F)	91%	1.3860	0.5171		514.7902	1027.5659	2	-0.0004	-0.3678	3,392.50	4	A0A7L8Z0C9,A0A7G3UUL1,A0A7L9W8W9_NDM,A0A7L9F8J2	123,342	4_Inactivated_PNGase-F_Treated_Purified Virions_2-7852-15781_15781	320	328
TRUE	0.1481	(K)IADYNYK(L)	90%	1.8556	0.8586		443.7212	885.4278	2	0.0045	5.0760	3,103.10	4	A0A7L8AB65,A0A7L8Z0C9,A0A7G3UUL1,A0A7L9W8W9_NDM	1,115,660	4_Inactivated_PNGase-F_Treated_Purified Virions_1-9272-15572_15572	418	424
TRUE	0.1481	(K)IADYNYK(L)	90%	1.6961	0.7985		443.7201	885.4256	2	0.0024	2.6660	3,075.25	4	A0A7L8AB65,A0A7L8Z0C9,A0A7G3UUL1,A0A7L9W8W9_NDM	823,219	4_Inactivated_PNGase-F_Treated_Purified Virions_2-6530-13962_13962	418	424
TRUE	0.1525	(K)VGGNYNYLYR(L)	93%	3.5527	0.8049		609.7979	1217.5812	2	-0.0017	-1.4110	4,354.07	4	A0A7L8AB65,A0A7L8Z0C9,A0A7L9W8W9_NDM,A0A7L9F8J2	622,786	4_Inactivated_PNGase-F_Treated_Purified Virions_1-15451-23342_23342	445	454
TRUE	0.1525	(K)VGGNYNYLYR(L)	91%	2.2351	0.6696		609.7984	1217.5822	2	-0.0006	-0.5095	4,346.59	4	A0A7L8AB65,A0A7L8Z0C9,A0A7L9W8W9_NDM,A0A7L9F8J2	250,654	4_Inactivated_PNGase-F_Treated_Purified Virions_2-12388-21577_21577	445	454
TRUE	0.1287	(R)VVVLSFELLHAPATVcGPK(K)	98%	4.5895	0.8170	Carbamidomethyl (+57)	679.7122	2036.1146	3	0.0016	0.7904	6,368.86	3	A0A7L8Z0C9,A0A7L9W8W9_NDM,A0A7L9F8J2	556,905	4_Inactivated_PNGase-F_Treated_Purified Virions_2-23627-34777_34777	510	528
TRUE	0.1287	(R)VVVLSFELLHAPATVcGPK(K)	97%	4.3779	0.8383	Carbamidomethyl (+57)	679.7113	2036.1121	3	-0.0010	-0.4680	6,370.91	3	A0A7L8Z0C9,A0A7L9W8W9_NDM,A0A7L9F8J2	651,551	4_Inactivated_PNGase-F_Treated_Purified Virions_1-27005-36681_36681	510	528
TRUE	0.1481	(K)QYGDcLGDIAAR(D)	93%	2.7680	0.8205	Carbamidomethyl (+57)	669.8095	1337.6045	2	0.0008	0.6205	4,439.36	5	A0A7L8AB65,A0A7L8Z0C9,A0A7G3UUL1,A0A7L9W8W9_NDM,A0A7L9F8J2	679,192	4_Inactivated_PNGase-F_Treated_Purified Virions_1-15892-23883_23883	836	847
TRUE	0.1669	(K)LQDVVNQNAQALNTLVK(Q)	93%	2.6329	0.8366		934.5165	1867.0185	2	0.0021	1.1020	5,475.00	3	A0A7L8Z0C9,A0A7L9W8W9_NDM,A0A7L9F8J2	337,109	4_Inactivated_PNGase-F_Treated_Purified Virions_1-21736-30714_30714	948	964
TRUE	0.1669	(K)LQDVVNQNAQALNTLVK(Q)	91%	1.1296	0.6600		934.5166	1867.0187	2	0.0022	1.1670	5,471.10	3	A0A7L8Z0C9,A0A7L9W8W9_NDM,A0A7L9F8J2	86,132	4_Inactivated_PNGase-F_Treated_Purified Virions_2-18512-28880_28880	948	964
**Spike glycoprotein OS=Severe acute respiratory syndrome coronavirus 2 OX=2697049 GN=S PE=3 SV=1 A0A7D5XMN0**																		
TRUE	0.3702	(K)VcEFQFcNDPFLGVYYHK(N)	98%	1.8994	0.6971	Carbamidomethyl (+57), Carbamidomethyl (+57)	775.0149	2322.0228	3	-0.0011	-0.4529	6,349.75	2	A0A7L8Z0C9,A0A7L9W8W9_NDM	250,005	4_Inactivated_PNGase-F_Treated_Purified Virions_2-23511-34649_34649	130	147
TRUE	0.2745	(K)LPDDFTGcVIAWNSNNLDSK(V)	93%	4.3011	0.9432	Carbamidomethyl (+57)	1133.5265	2265.0384	2	0.0010	0.4371	6,181.58	2	A0A7L8Z0C9,A0A7L9W8W9_NDM	261,016	4_Inactivated_PNGase-F_Treated_Purified Virions_1-25887-35420_35420	425	444
TRUE	0.2745	(K)LPDDFTGcVIAWNSNNLDSK(V)	91%	2.4746	0.9400	Carbamidomethyl (+57)	1133.5221	2265.0296	2	-0.0078	-3.4410	6,174.66	2	A0A7L8Z0C9,A0A7L9W8W9_NDM	97,202	4_Inactivated_PNGase-F_Treated_Purified Virions_2-22491-33490_33490	425	444
TRUE	0.3602	(R)ALTGIAVEQDKNTQEVFAQVK(Q)	97%	4.1310	0.8809		763.7421	2288.2046	3	0.0031	1.3460	5,448.28	2	A0A7L8Z0C9,A0A7L9W8W9_NDM	189,807	4_Inactivated_PNGase-F_Treated_Purified Virions_1-21581-30536_30536	766	786
TRUE	0.3571	(R)SFIEDLLFNKVTLADAGFIK(Q)	91%	0.8539	0.5289		747.7430	2240.2071	3	-0.0025	-1.0960	6,726.47	2	A0A7L8Z0C9,A0A7L9W8W9_NDM	41,962	4_Inactivated_PNGase-F_Treated_Purified Virions_2-25754-37166_37166	816	835

[a] NDM, Asn to Asp modification. The NDM annotation was manually added following the accession number of the original Spike sequence to indicate that the asparagine (N) residues were in-silico-modified to aspartic acid (D) by an in-house script, to account for an experimental *N*-deglycosylation by PNGase-F treatment.

[b] c, Modified Cys residue, as indicated in the Modification column.

[c] The resulting identified *N*-deglycosylated site (D, bold blue, underlined) and the two additional amino acids (X-T/S) (bold blue) of the conserved *N*-glycosylation sequon are indicated.

Identified M proteins

Bio Sample Category / Sequence Coverage [a]	Protein	Accession	Category/Bio Sample	Protein identification probability (Prob)	Exclusive unique peptide count (#Pep)	Exclusive unique spectrum count (#Unique)	Total spectrum count (#Spec)	Percentage of total spectra (%Spec)	Percentage of amino acids identified (%Cov)	Protein molecular mass (kDa)
**1_Control_Purified_Virions**										
A0A7M1HLZ4 (64%), 25,215.5 Da<br>Membrane glycoprotein OS=Severe acute respiratory syndrome coronavirus 2 OX=2697049 GN=M PE=4 SV=1<br>0 exclusive unique peptides, 0 exclusive unique spectra, 1 total spectra, 22/222 amino acids (10% coverage)<br>MSDSNGTITV EELKKLLEQW NLVIGFLFLT WICLLQFAYA NRNRFLYIIK LIFLWLLWPV TLACFVLAAV YRINWITGGI<br>AIAMACLVGL MWLSYFIASF RLFARTRSMW SFNPETNILL NVPLHGTIXX XXXXXXXXXX XXXXXXXXLR IAGHHLGRCD<br>IKDLPKEITV ATSRTLSYYK LGASQRVAGD SGFAAYSRYR IGNYKLNTDH SSSSDNIALL VQ	Membrane glycoprotein OS=Severe acute respiratory syndrome coronavirus 2 OX=2697049 GN=M PE=4 SV=1	A0A7M1HLZ4	1_Control_Purified_virons_1	64%	0	0	1	0.0021%	9.9%	25 kDa
A0A6M4NE17 (40%), 25,149.2 Da<br>Membrane protein OS=Severe acute respiratory syndrome coronavirus 2 OX=2697049 GN=M PE=3 SV=1<br>0 exclusive unique peptides, 0 exclusive unique spectra, 1 total spectra, 22/222 amino acids (10% coverage)<br>MADSNGTITV EELKKLLEQW NLVIGFLFLT WICLLQFAYA NRNRFLYIIK LIFLWLLWPV TLACFVLAAV YRINWITGGI<br>AIAMACLVGL MWLSYFIASF RLFARTRSMW SFDPETNILL NVPLHGTILT RPLLESELVI GAVILRGHLR IAGHHLGRCD<br>IKDLPKEITV ATSRTLSYYK LGASQRVAGD SGFAAYSRYR IGNYKLNTDH SSSSDNIALL VQ	Membrane protein OS=Severe acute respiratory syndrome coronavirus 2 OX=2697049 GN=M PE=3 SV=1	A0A6M4NE17	1_Control_Purified_virons_1	40%	0	0	1	0.0021%	9.9%	25 kDa
**2_Mock_Treated_Purified_Virions**										
A0A7M1HLZ4 (71%), 25,215.5 Da<br>Membrane glycoprotein OS=Severe acute respiratory syndrome coronavirus 2 OX=2697049 GN=M PE=4 SV=1<br>0 exclusive unique peptides, 0 exclusive unique spectra, 3 total spectra, 38/222 amino acids (17% coverage)<br>MSDSNGTITV EELKKLLEQW NLVIGFLFLT WICLLQFAYA NRNRFLYIIK LIFLWLLWPV TLACFVLAAV YRINWITGGI<br>AIAMACLVGL MWLSYFIASF RLFARTRSMW SFNPETNILL NVPLHGTIXX XXXXXXXXXX XXXXXXXXLR IAGHHLGRCD<br>IKDLPKEITV ATSRTLSYYK LGASQRVAGD SGFAAYSRYR IGNYKLNTDH SSSSDNIALL VQ	Membrane glycoprotein OS=Severe acute respiratory syndrome coronavirus 2 OX=2697049 GN=M PE=4 SV=1	A0A7M1HLZ4	2_Mock_Purified_Virons_mock_treated_1	71%	0	0	3	0.0063%	17.1%	25 kDa
A0A6M4NE17 (46%), 25,149.2 Da<br>Membrane protein OS=Severe acute respiratory syndrome coronavirus 2 OX=2697049 GN=M PE=3 SV=1<br>0 exclusive unique peptides, 0 exclusive unique spectra, 3 total spectra, 38/222 amino acids (17% coverage)<br>MADSNGTITV EELKKLLEQW NLVIGFLFLT WICLLQFAYA NRNRFLYIIK LIFLWLLWPV TLACFVLAAV YRINWITGGI<br>AIAMACLVGL MWLSYFIASF RLFARTRSMW SFDPETNILL NVPLHGTILT RPLLESELVI GAVILRGHLR IAGHHLGRCD<br>IKDLPKEITV ATSRTLSYYK LGASQRVAGD SGFAAYSRYR IGNYKLNTDH SSSSDNIALL VQ	Membrane protein OS=Severe acute respiratory syndrome coronavirus 2 OX=2697049 GN=M PE=3 SV=1	A0A6M4NE17	2_Mock_Purified_Virons_mock_treated_1	46%	0	0	3	0.0063%	17.1%	25 kDa
**3_PNGase-F_Treated_Purified_Virions**										
A0A7M1HLZ4 (96%), 25,215.5 Da<br>Membrane glycoprotein OS=Severe acute respiratory syndrome coronavirus 2 OX=2697049 GN=M PE=4 SV=1<br>0 exclusive unique peptides, 0 exclusive unique spectra, 15 total spectra, 53/222 amino acids (24% coverage)<br>MSDSNGTITV EELKKLLEQW NLVIGFLFLT WICLLQFAYA NRNRFLYIIK LIFLWLLWPV TLACFVLAAV YRINWITGGI<br>AIAMACLVGL MWLSYFIASF RLFARTRSMW SFNPETNILL NVPLHGTIXX XXXXXXXXXX XXXXXXXXLR IAGHHLGRCD<br>IKDLPKEITV ATSRTLSYYK LGASQRVAGD SGFAAYSRYR IGNYKLNTDH SSSSDNIALL VQ	Membrane glycoprotein OS=Severe acute respiratory syndrome coronavirus 2 OX=2697049 GN=M PE=4 SV=1	A0A7M1HLZ4	3_PNG+_purified_virons_PNGase-F_treated_1	96%	0	0	15	0.0314%	23.9%	25 kDa
A0A6M4NE17 (97%), 25,149.2 Da<br>Membrane protein OS=Severe acute respiratory syndrome coronavirus 2 OX=2697049 GN=M PE=3 SV=1<br>0 exclusive unique peptides, 0 exclusive unique spectra, 15 total spectra, 53/222 amino acids (24% coverage)<br>MADSNGTITV EELKKLLEQW NLVIGFLFLT WICLLQFAYA NRNRFLYIIK LIFLWLLWPV TLACFVLAAV YRINWITGGI<br>AIAMACLVGL MWLSYFIASF RLFARTRSMW SFDPETNILL NVPLHGTILT RPLLESELVI GAVILRGHLR IAGHHLGRCD<br>IKDLPKEITV ATSRTLSYYK LGASQRVAGD SGFAAYSRYR IGNYKLNTDH SSSSDNIALL VQ	Membrane protein OS=Severe acute respiratory syndrome coronavirus 2 OX=2697049 GN=M PE=3 SV=1	A0A6M4NE17	3_PNG+_purified_virons_PNGase-F_treated_1	97%	0	0	15	0.0314%	23.9%	25 kDa
**4_Inactivated PNGase-F_Treated_Purified_Virions**										
A0A7M1HLZ4 (92%), 25,215.5 Da<br>Membrane glycoprotein OS=Severe acute respiratory syndrome coronavirus 2 OX=2697049 GN=M PE=4 SV=1<br>0 exclusive unique peptides, 0 exclusive unique spectra, 13 total spectra, 45/222 amino acids (20% coverage)<br>MSDSNGTITV EELKKLLEQW NLVIGFLFLT WICLLQFAYA NRNRFLYIIK LIFLWLLWPV TLACFVLAAV YRINWITGGI<br>AIAMACLVGL MWLSYFIASF RLFARTRSMW SFNPETNILL NVPLHGTIXX XXXXXXXXXX XXXXXXXXLR IAGHHLGRCD<br>IKDLPKEITV ATSRTLSYYK LGASQRVAGD SGFAAYSRYR IGNYKLNTDH SSSSDNIALL VQ	Membrane glycoprotein OS=Severe acute respiratory syndrome coronavirus 2 OX=2697049 GN=M PE=4 SV=1	A0A7M1HLZ4	4_Inactiv_purified_virons_PNGase-F_inactivated_1	92%	0	0	13	0.0303%	20.3%	25 kDa
A0A6M4NE17 (37%), 25,149.2 Da<br>Membrane protein OS=Severe acute respiratory syndrome coronavirus 2 OX=2697049 GN=M PE=3 SV=1<br>0 exclusive unique peptides, 0 exclusive unique spectra, 13 total spectra, 45/222 amino acids (20% coverage)<br>MADSNGTITV EELKKLLEQW NLVIGFLFLT WICLLQFAYA NRNRFLYIIK LIFLWLLWPV TLACFVLAAV YRINWITGGI<br>AIAMACLVGL MWLSYFIASF RLFARTRSMW SFDPETNILL NVPLHGTILT RPLLESELVI GAVILRGHLR IAGHHLGRCD<br>IKDLPKEITV ATSRTLSYYK LGASQRVAGD SGFAAYSRYR IGNYKLNTDH SSSSDNIALL VQ	Membrane protein OS=Severe acute respiratory syndrome coronavirus 2 OX=2697049 GN=M PE=3 SV=1	A0A6M4NE17	4_Inactiv_purified_virons_PNGase-F_inactivated_1	37%	0	0	13	0.0303%	20.3%	25 kDa

[a] The identified peptides are highlighted in orange; modified Cys residues are highlighted in green.

[b] NDM, Asn to Asp modification. The NDM annotation was manually added following the accession number of the original Spike sequence to indicate that the asparagine (N) residues were in-silico-modified to aspartic acid (D) by an in-house script, to account for an experimental *N*-deglycosylation by PNGase-F treatment.

Identified M Peptides

Valid	Weight	Sequence [a]	Protein identification probability (Prob)	SEQUEST XCorr	SEQUEST deltaCn	Modifications	Observed Mass (Da)	Actual Mass (Da)	Charge	Delta Da	Delta PPM	Retention Time	# Other Proteins	Other Proteins	Total Ion Chromatogram (TIC)	Spectrum ID	Peptide Start Index	Peptide Stop Index
**1_Control_Purified_Virions**																		
**Membrane glycoprotein OS=Severe acute respiratory syndrome coronavirus 2 OX=2697049 GN=M PE=4 SV=1 Accession A0A7M1HLZ4**																		
TRUE	0.2233	(R)IGNYKLNTDHSSSSDNIALLVQ(-)	99%	3.4264	0.7533		797.0711	2388.1915	3	-0.0008	-0.3271	5,502.49	1	A0A6M4NE17	5,133,970	1_Control_Purified_Virions_1-21185-30704_30704	201	222
**Membrane protein OS=Severe acute respiratory syndrome coronavirus 2 OX=2697049 GN=M PE=3 SV=1 Accession A0A6M4NE17**																		
TRUE	0.1169	(R)IGNYKLNTDHSSSSDNIALLVQ(-)	99%	3.4264	0.7533		797.0711	2388.1915	3	-0.0008	-0.3271	5,502.49	1	A0A7M1HLZ4	5,133,970	1_Control_Purified_Virions_1-21185-30704_30704	201	222
**2_Mock_Treated_Purified_Virions**																		
**Membrane glycoprotein OS=Severe acute respiratory syndrome coronavirus 2 OX=2697049 GN=M PE=4 SV=1 Accession A0A7M1HLZ4**																		
TRUE	0.1850	(R)cDIKDLPKEITVATSR(T)	96%	4.3560	0.8228	Carbamidomethyl (+57)	615.9951	1844.9635	3	-0.0034	-1.8490	5,203.70	1	A0A6M4NE17	1,115,740	2_Mock_Treated_Purified_Virions_2-19647-29048_29048	159	174
TRUE	0.1850	(R)cDIKDLPKEITVATSR(T)	96%	1.6986	0.5611	Carbamidomethyl (+57)	462.2492	1844.9677	4	0.0008	0.4252	5,201.45	1	A0A6M4NE17	260,680	2_Mock_Treated_Purified_Virions_1-19476-28683_28683	159	174
TRUE	0.2233	(R)IGNYKLNTDHSSSSDNIALLVQ(-)	96%	4.2156	0.7798		797.0696	2388.1869	3	-0.0054	-2.2430	5,538.09	1	A0A6M4NE17	3,857,450	2_Mock_Treated_Purified_Virions_2-21573-31267_31267	201	222
**Membrane protein OS=Severe acute respiratory syndrome coronavirus 2 OX=2697049 GN=M PE=3 SV=1 Accession A0A6M4NE17**																		
TRUE	0.0969	(R)cDIKDLPKEITVATSR(T)	96%	4.3560	0.8228	Carbamidomethyl (+57)	615.9951	1844.9635	3	-0.0034	-1.8490	5,203.70	1	A0A7M1HLZ4	1,115,740	2_Mock_Treated_Purified_Virions_2-19647-29048_29048	159	174
TRUE	0.0969	(R)cDIKDLPKEITVATSR(T)	96%	1.6986	0.5611	Carbamidomethyl (+57)	462.2492	1844.9677	4	0.0008	0.4252	5,201.45	1	A0A7M1HLZ4	260,680	2_Mock_Treated_Purified_Virions_1-19476-28683_28683	159	174
TRUE	0.1169	(R)IGNYKLNTDHSSSSDNIALLVQ(-)	96%	4.2156	0.7798		797.0696	2388.1869	3	-0.0054	-2.2430	5,538.09	1	A0A7M1HLZ4	3,857,450	2_Mock_Treated_Purified_Virions_2-21573-31267_31267	201	222
**3_PNGase-F_Treated_Purified_Virions**																		
**Membrane glycoprotein OS=Severe acute respiratory syndrome coronavirus 2 OX=2697049 GN=M PE=4 SV=1 Accession A0A7M1HLZ4**																		
TRUE	0.1935	(R)IAGHHLGR(C)	97%	1.8688	0.7610		430.7462	859.4779	2	0.0002	0.1976	2,003.10	1	A0A6M4NE17	1,016,910	3_PNGase-F_Treated_Purified_Virions_2-5037-9778_9778	151	158
TRUE	0.1935	(R)IAGHHLGR(C)	96%	1.5032	0.8443		430.7464	859.4782	2	0.0004	0.4813	2,154.20	1	A0A6M4NE17	511,700	3_PNGase-F_Treated_Purified_Virions_1-5322-10432_10432	151	158
TRUE	0.2037	(R)cDIKDLPK(E)	97%	2.9740	0.7020	Carbamidomethyl (+57)	494.7603	987.5060	2	-0.0002	-0.2216	3,324.86	1	A0A6M4NE17	621,187	3_PNGase-F_Treated_Purified_Virions_2-10827-17540_17540	159	166
TRUE	0.2037	(R)cDIKDLPK(E)	96%	3.1616	0.6943	Carbamidomethyl (+57)	494.7609	987.5073	2	0.0011	1.0750	3,351.54	1	A0A6M4NE17	977,911	3_PNGase-F_Treated_Purified_Virions_1-10298-17321_17321	159	166
TRUE	0.1750	(K)EITVATSR(T)	97%	1.9847	0.6836		438.7430	875.4714	2	0.0001	0.1589	2,811.65	1	A0A6M4NE17	816,236	3_PNGase-F_Treated_Purified_Virions_2-8433-14430_14430	167	174
TRUE	0.1750	(K)EITVATSR(T)	95%	1.8295	0.6244		438.7429	875.4713	2	0.0000	0.0196	2,854.28	1	A0A6M4NE17	646,781	3_PNGase-F_Treated_Purified_Virions_1-8173-14412_14412	167	174
TRUE	0.1750	(K)EITVATSR(T)	94%	1.6332	0.5684		438.7427	875.4709	2	-0.0004	-0.4678	2,832.71	1	A0A6M4NE17	204,129	3_PNGase-F_Treated_Purified_Virions_2-8537-14561_14561	167	174
TRUE	0.1750	(K)EITVATSR(T)	93%	1.5956	0.5395		438.7433	875.4721	2	0.0009	0.9946	2,875.58	1	A0A6M4NE17	239,368	3_PNGase-F_Treated_Purified_Virions_1-8276-14543_14543	167	174
TRUE	0.2266	(R)VAGDSGFAAYSR(Y)	97%	3.7174	0.8590		600.7866	1199.5587	2	0.0016	1.3490	3,797.55	1	A0A6M4NE17	960,832	3_PNGase-F_Treated_Purified_Virions_2-13265-20551_20551	187	198
TRUE	0.2266	(R)VAGDSGFAAYSR(Y)	97%	3.2595	0.8700		600.7857	1199.5569	2	-0.0002	-0.1762	3,817.71	1	A0A6M4NE17	713,943	3_PNGase-F_Treated_Purified_Virions_2-13353-20670_20670	187	198
TRUE	0.2266	(R)VAGDSGFAAYSR(Y)	96%	3.7937	0.8743		600.7866	1199.5587	2	0.0016	1.3490	3,826.81	1	A0A6M4NE17	1,061,920	3_PNGase-F_Treated_Purified_Virions_1-12537-20227_20227	187	198
TRUE	0.2266	(R)VAGDSGFAAYSR(Y)	96%	3.5872	0.8744		600.7866	1199.5586	2	0.0015	1.2470	3,847.44	1	A0A6M4NE17	315,625	3_PNGase-F_Treated_Purified_Virions_1-12622-20347_20347	187	198
TRUE	0.2105	(K)LNTDHSSSSDNIALLVQ(-)	97%	3.2589	0.8944		907.4501	1812.8856	2	0.0001	0.0683	5,253.76	1	A0A6M4NE17	1,944,790	3_PNGase-F_Treated_Purified_Virions_2-21341-30121_30121	206	222
TRUE	0.2105	(K)LNTDHSSSSDNIALLVQ(-)	96%	3.7648	0.8943		907.4504	1812.8862	2	0.0007	0.4048	5,280.12	1	A0A6M4NE17	2,064,470	3_PNGase-F_Treated_Purified_Virions_1-20099-29532_29532	206	222
TRUE	0.2105	(K)LNTDHSSSSDNIALLVQ(-)	95%	1.3971	0.8266		907.4496	1812.8847	2	-0.0007	-0.4028	5,233.12	1	A0A6M4NE17	190,195	3_PNGase-F_Treated_Purified_Virions_2-21217-29982_29982	206	222
**Membrane protein OS=Severe acute respiratory syndrome coronavirus 2 OX=2697049 GN=M PE=3 SV=1 A0A6M4NE17**																		
TRUE	0.1013	(R)IAGHHLGR(C)	97%	1.8688	0.7610		430.7462	859.4779	2	0.0002	0.1976	2,003.10	1	A0A7M1HLZ4	1,016,910	3_PNGase-F_Treated_Purified_Virions_2-5037-9778_9778	151	158
TRUE	0.1013	(R)IAGHHLGR(C)	96%	1.5032	0.8443		430.7464	859.4782	2	0.0004	0.4813	2,154.20	1	A0A7M1HLZ4	511,700	3_PNGase-F_Treated_Purified_Virions_1-5322-10432_10432	151	158
TRUE	0.1067	(R)cDIKDLPK(E)	97%	2.9740	0.7020	Carbamidomethyl (+57)	494.7603	987.5060	2	-0.0002	-0.2216	3,324.86	1	A0A7M1HLZ4	621,187	3_PNGase-F_Treated_Purified_Virions_2-10827-17540_17540	159	166
TRUE	0.1067	(R)cDIKDLPK(E)	96%	3.1616	0.6943	Carbamidomethyl (+57)	494.7609	987.5073	2	0.0011	1.0750	3,351.54	1	A0A7M1HLZ4	977,911	3_PNGase-F_Treated_Purified_Virions_1-10298-17321_17321	159	166
TRUE	0.0917	(K)EITVATSR(T)	97%	1.9847	0.6836		438.7430	875.4714	2	0.0001	0.1589	2,811.65	1	A0A7M1HLZ4	816,236	3_PNGase-F_Treated_Purified_Virions_2-8433-14430_14430	167	174
TRUE	0.0917	(K)EITVATSR(T)	95%	1.8295	0.6244		438.7429	875.4713	2	0.0000	0.0196	2,854.28	1	A0A7M1HLZ4	646,781	3_PNGase-F_Treated_Purified_Virions_1-8173-14412_14412	167	174
TRUE	0.0917	(K)EITVATSR(T)	94%	1.6332	0.5684		438.7427	875.4709	2	-0.0004	-0.4678	2,832.71	1	A0A7M1HLZ4	204,129	3_PNGase-F_Treated_Purified_Virions_2-8537-14561_14561	167	174
TRUE	0.0917	(K)EITVATSR(T)	93%	1.5956	0.5395		438.7433	875.4721	2	0.0009	0.9946	2,875.58	1	A0A7M1HLZ4	239,368	3_PNGase-F_Treated_Purified_Virions_1-8276-14543_14543	167	174
TRUE	0.1187	(R)VAGDSGFAAYSR(Y)	97%	3.7174	0.8590		600.7866	1199.5587	2	0.0016	1.3490	3,797.55	1	A0A7M1HLZ4	960,832	3_PNGase-F_Treated_Purified_Virions_2-13265-20551_20551	187	198
TRUE	0.1187	(R)VAGDSGFAAYSR(Y)	97%	3.2595	0.8700		600.7857	1199.5569	2	-0.0002	-0.1762	3,817.71	1	A0A7M1HLZ4	713,943	3_PNGase-F_Treated_Purified_Virions_2-13353-20670_20670	187	198
TRUE	0.1187	(R)VAGDSGFAAYSR(Y)	96%	3.7937	0.8743		600.7866	1199.5587	2	0.0016	1.3490	3,826.81	1	A0A7M1HLZ4	1,061,920	3_PNGase-F_Treated_Purified_Virions_1-12537-20227_20227	187	198
TRUE	0.1187	(R)VAGDSGFAAYSR(Y)	96%	3.5872	0.8744		600.7866	1199.5586	2	0.0015	1.2470	3,847.44	1	A0A7M1HLZ4	315,625	3_PNGase-F_Treated_Purified_Virions_1-12622-20347_20347	187	198
TRUE	0.1102	(K)LNTDHSSSSDNIALLVQ(-)	97%	3.2589	0.8944		907.4501	1812.8856	2	0.0001	0.0683	5,253.76	1	A0A7M1HLZ4	1,944,790	3_PNGase-F_Treated_Purified_Virions_2-21341-30121_30121	206	222
TRUE	0.1102	(K)LNTDHSSSSDNIALLVQ(-)	96%	3.7648	0.8943		907.4504	1812.8862	2	0.0007	0.4048	5,280.12	1	A0A7M1HLZ4	2,064,470	3_PNGase-F_Treated_Purified_Virions_1-20099-29532_29532	206	222
TRUE	0.1102	(K)LNTDHSSSSDNIALLVQ(-)	95%	1.3971	0.8266		907.4496	1812.8847	2	-0.0007	-0.4028	5,233.12	1	A0A7M1HLZ4	190,195	3_PNGase-F_Treated_Purified_Virions_2-21217-29982_29982	206	222
**4_Inactivated PNGase-F_Treated_Purified_Virions**																		
**4_Inactiv_purified_virons_PNGase-F_inactivated_1 A0A7M1HLZ4**																		
TRUE	0.2037	(R)cDIKDLPK(E)	93%	3.3037	0.7293	Carbamidomethyl (+57)	494.7609	987.5072	2	0.0010	1.0130	3,349.45	1	A0A6M4NE17	560,915	4_Inactivated_PNGase-F_Purified_Virions_1-10431-17072_17072	159	166
TRUE	0.2037	(R)cDIKDLPK(E)	91%	2.8560	0.6945	Carbamidomethyl (+57)	494.7613	987.5080	2	0.0017	1.7540	3,319.89	1	A0A6M4NE17	455,696	4_Inactivated_PNGase-F_Purified_Virions_2-7538-15355_15355	159	166
TRUE	0.1750	(K)EITVATSR(T)	93%	1.9068	0.6144		438.7431	875.4717	2	0.0004	0.5071	2,857.04	1	A0A6M4NE17	538,508	4_Inactivated_PNGase-F_Purified_Virions_1-8037-14035_14035	167	174
TRUE	0.1750	(K)EITVATSR(T)	93%	1.7285	0.6008		438.7429	875.4712	2	-0.0001	-0.1196	2,877.49	1	A0A6M4NE17	303,154	4_Inactivated_PNGase-F_Purified_Virions_1-8122-14152_14152	167	174
TRUE	0.1750	(K)EITVATSR(T)	91%	2.1375	0.7169		438.7428	875.4711	2	-0.0002	-0.1893	2,812.82	1	A0A6M4NE17	2,849,730	4_Inactivated_PNGase-F_Purified_Virions_2-5559-12546_12546	167	174
TRUE	0.1750	(K)EITVATSR(T)	91%	1.3316	0.5497		438.7429	875.4712	2	0.0000	-0.0500	2,833.16	1	A0A6M4NE17	185,931	4_Inactivated_PNGase-F_Purified_Virions_2-5621-12647_12647	167	174
TRUE	0.2266	(R)VAGDSGFAAYSR(Y)	93%	3.6994	0.8761		600.7858	1199.5570	2	-0.0001	-0.0745	3,813.86	1	A0A6M4NE17	794,522	4_Inactivated_PNGase-F_Purified_Virions_1-12627-19892_19892	187	198
TRUE	0.2266	(R)VAGDSGFAAYSR(Y)	93%	3.2152	0.9065		600.7849	1199.5553	2	-0.0018	-1.4980	3,834.60	1	A0A6M4NE17	210,293	4_Inactivated_PNGase-F_Purified_Virions_1-12726-20019_20019	187	198
TRUE	0.2266	(R)VAGDSGFAAYSR(Y)	91%	3.1606	0.8915		600.7842	1199.5538	2	-0.0033	-2.7180	3,815.42	1	A0A6M4NE17	266,334	4_Inactivated_PNGase-F_Purified_Virions_2-9684-18250_18250	187	198
TRUE	0.2266	(R)VAGDSGFAAYSR(Y)	91%	3.3918	0.8489		600.7862	1199.5578	2	0.0008	0.6373	3,794.65	1	A0A6M4NE17	516,065	4_Inactivated_PNGase-F_Purified_Virions_2-9612-18138_18138	187	198
TRUE	0.2105	(K)LNTDHSSSSDNIALLVQ(-)	93%	2.9937	0.9105		907.4500	1812.8855	2	0.0000	0.0010	5,269.31	1	A0A6M4NE17	1,298,710	4_Inactivated_PNGase-F_Purified_Virions_1-20554-29354_29354	206	222
TRUE	0.2105	(K)LNTDHSSSSDNIALLVQ(-)	91%	2.4156	0.9355		907.4504	1812.8862	2	0.0007	0.4048	5,269.52	1	A0A6M4NE17	504,310	4_Inactivated_PNGase-F_Purified_Virions_2-17435-27593_27593	206	222
TRUE	0.2105	(K)LNTDHSSSSDNIALLVQ(-)	91%	1.2895	0.6943		907.4526	1812.8907	2	0.0053	2.8950	5,248.03	1	A0A6M4NE17	82,198	4_Inactivated_PNGase-F_Purified_Virions_2-17310-27450_27450	206	222
**Membrane protein OS=Severe acute respiratory syndrome coronavirus 2 OX=2697049 GN=M PE=3 SV=1 A0A6M4NE17**																		
TRUE	0.1067	(R)cDIKDLPK(E)	93%	3.3037	0.7293	Carbamidomethyl (+57)	494.7609	987.5072	2	0.0010	1.0130	3,349.45	1	A0A7M1HLZ4	560,915	4_Inactivated_PNGase-F_Purified_Virions_1-10431-17072_17072	159	166
TRUE	0.1067	(R)cDIKDLPK(E)	91%	2.8560	0.6945	Carbamidomethyl (+57)	494.7613	987.5080	2	0.0017	1.7540	3,319.89	1	A0A7M1HLZ4	455,696	4_Inactivated_PNGase-F_Purified_Virions_2-7538-15355_15355	159	166
TRUE	0.0917	(K)EITVATSR(T)	93%	1.9068	0.6144		438.7431	875.4717	2	0.0004	0.5071	2,857.04	1	A0A7M1HLZ4	538,508	4_Inactivated_PNGase-F_Purified_Virions_1-8037-14035_14035	167	174
TRUE	0.0917	(K)EITVATSR(T)	93%	1.7285	0.6008		438.7429	875.4712	2	-0.0001	-0.1196	2,877.49	1	A0A7M1HLZ4	303,154	4_Inactivated_PNGase-F_Purified_Virions_1-8122-14152_14152	167	174
TRUE	0.0917	(K)EITVATSR(T)	91%	2.1375	0.7169		438.7428	875.4711	2	-0.0002	-0.1893	2,812.82	1	A0A7M1HLZ4	2,849,730	4_Inactivated_PNGase-F_Purified_Virions_2-5559-12546_12546	167	174
TRUE	0.0917	(K)EITVATSR(T)	91%	1.3316	0.5497		438.7429	875.4712	2	0.0000	-0.0500	2,833.16	1	A0A7M1HLZ4	185,931	4_Inactivated_PNGase-F_Purified_Virions_2-5621-12647_12647	167	174
TRUE	0.1187	(R)VAGDSGFAAYSR(Y)	93%	3.6994	0.8761		600.7858	1199.5570	2	-0.0001	-0.0745	3,813.86	1	A0A7M1HLZ4	794,522	4_Inactivated_PNGase-F_Purified_Virions_1-12627-19892_19892	187	198
TRUE	0.1187	(R)VAGDSGFAAYSR(Y)	93%	3.2152	0.9065		600.7849	1199.5553	2	-0.0018	-1.4980	3,834.60	1	A0A7M1HLZ4	210,293	4_Inactivated_PNGase-F_Purified_Virions_1-12726-20019_20019	187	198
TRUE	0.1187	(R)VAGDSGFAAYSR(Y)	91%	3.1606	0.8915		600.7842	1199.5538	2	-0.0033	-2.7180	3,815.42	1	A0A7M1HLZ4	266,334	4_Inactivated_PNGase-F_Purified_Virions_2-9684-18250_18250	187	198
TRUE	0.1187	(R)VAGDSGFAAYSR(Y)	91%	3.3918	0.8489		600.7862	1199.5578	2	0.0008	0.6373	3,794.65	1	A0A7M1HLZ4	516,065	4_Inactivated_PNGase-F_Purified_Virions_2-9612-18138_18138	187	198
TRUE	0.1102	(K)LNTDHSSSSDNIALLVQ(-)	93%	2.9937	0.9105		907.4500	1812.8855	2	0.0000	0.0010	5,269.31	1	A0A7M1HLZ4	1,298,710	4_Inactivated_PNGase-F_Purified_Virions_1-20554-29354_29354	206	222
TRUE	0.1102	(K)LNTDHSSSSDNIALLVQ(-)	91%	2.4156	0.9355		907.4504	1812.8862	2	0.0007	0.4048	5,269.52	1	A0A7M1HLZ4	504,310	4_Inactivated_PNGase-F_Purified_Virions_2-17435-27593_27593	206	222
TRUE	0.1102	(K)LNTDHSSSSDNIALLVQ(-)	91%	1.2895	0.6943		907.4526	1812.8907	2	0.0053	2.8950	5,248.03	1	A0A7M1HLZ4	82,198	4_Inactivated_PNGase-F_Purified_Virions_2-17310-27450_27450	206	222

[a] c, Modified Cys residue, as indicated in the Modifications column.
