# Supplementary material for: Inhibition of Protein N-Glycosylation Blocks SARS-CoV-2 Infection
Source: mBio. 2022 Feb 15;13(1):e03718-21. doi: 10.1128/mbio.03718-21 (PMC8844921; doi:10.1128/mbio.03718-21)
Supplement: DATA SET S5 [file mbio.03718-21-sd005.pdf]

A

1

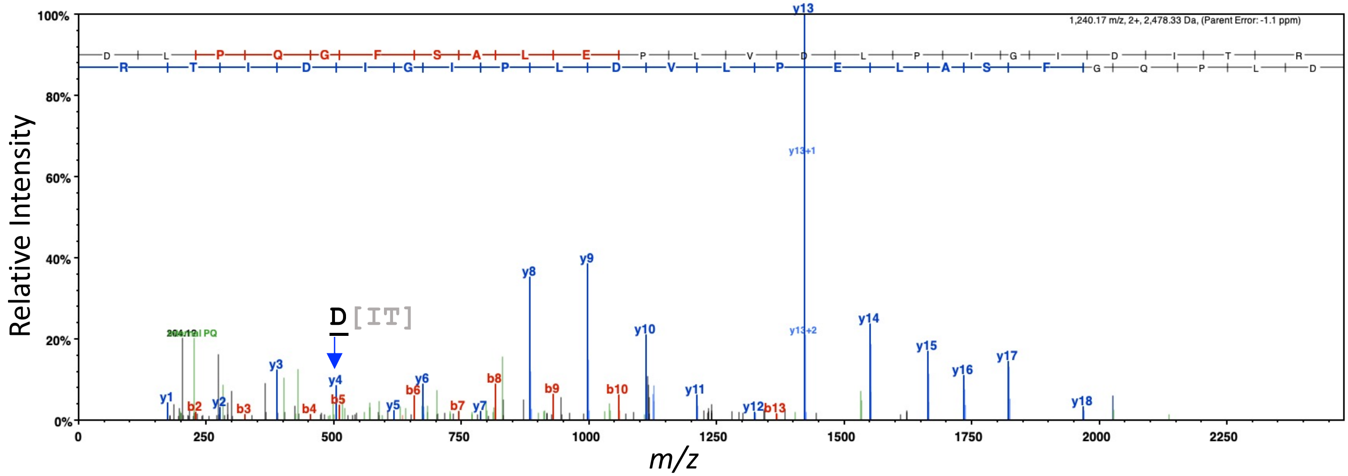

2

B	B Ions	B+2H	B-NH3	B-H2O	AA	Y Ions	Y+2H	Y-NH3	Y-H2O	Y
1	116.0			98.0	D	2,479.3	1,240.2	2,462.3	2,461.3	23
2	229.1			211.1	L	2,364.3	1,182.7	2,347.3	2,346.3	22
3	326.2			308.2	P	2,251.2	1,126.1	2,234.2	2,233.2	21
4	454.2		437.2	436.2	Q	2,154.2	1,077.6	2,137.2	2,136.2	20
5	511.3		494.2	493.2	G	2,026.1	1,013.6	2,009.1	2,008.1	19
6	658.3	329.7	641.3	640.3	F	1,969.1	985.1	1,952.1	1,951.1	18
7	745.4	373.2	728.3	727.3	S	1,822.0	911.5	1,805.0	1,804.0	17
8	816.4	408.7	799.4	798.4	A	1,735.0	868.0	1,718.0	1,717.0	16
9	929.5	465.2	912.4	911.5	L	1,664.0	832.5	1,646.9	1,646.0	15
10	1,058.5	529.8	1,041.5	1,040.5	E	1,550.9	775.9	1,533.9	1,532.9	14
11	1,155.6	578.3	1,138.5	1,137.6	P	1,421.8	711.4	1,404.8	1,403.8	13
12	1,268.7	634.8	1,251.6	1,250.6	L	1,324.8	662.9	1,307.8	1,306.8	12
13	1,367.7	684.4	1,350.7	1,349.7	V	1,211.7	606.4	1,194.7	1,193.7	11
14	1,482.7	741.9	1,465.7	1,464.7	D	1,112.6	556.8	1,095.6	1,094.6	10
15	1,595.8	798.4	1,578.8	1,577.8	L	997.6	499.3	980.6	979.6	9
16	1,692.9	846.9	1,675.9	1,674.9	P	884.5	442.8	867.5	866.5	8
17	1,806.0	903.5	1,788.9	1,788.0	I	787.5	394.2	770.4	769.5	7
18	1,863.0	932.0	1,846.0	1,845.0	G	674.4	337.7	657.4	656.4	6
19	1,976.1	988.5	1,959.0	1,958.1	I	617.4		600.3	599.4	5
20	2,091.1	1,046.1	2,074.1	2,073.1	D	504.3		487.3	486.3	4
21	2,204.2	1,102.6	2,187.2	2,186.2	I	389.3		372.2	371.2	3
22	2,305.2	1,153.1	2,288.2	2,287.2	T	276.2		259.1	258.2	2
23	2,479.3	1,240.2	2,462.3	2,461.3	R	175.1		158.1		1

3

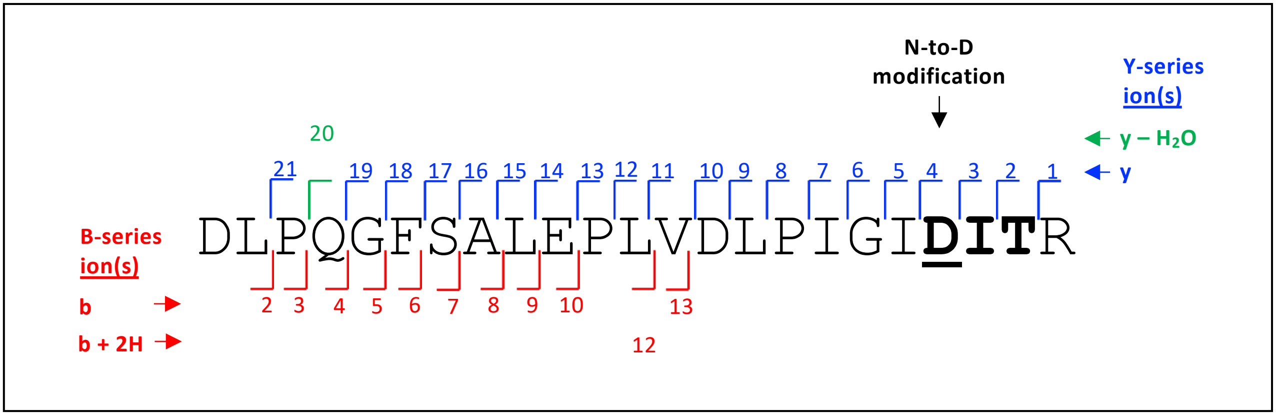

B

1

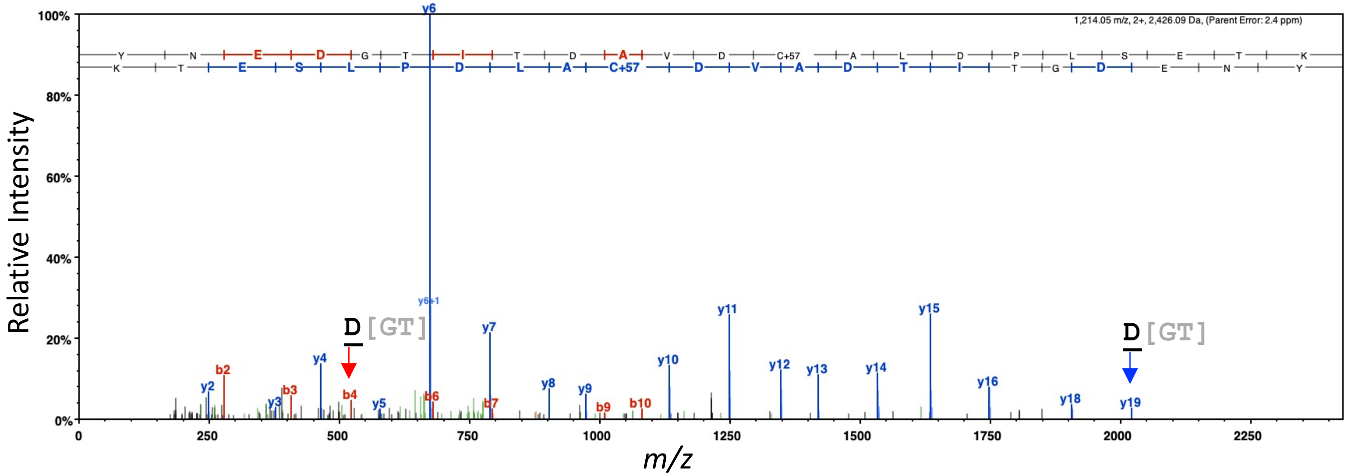

2

B	B Ions	B+2H	B-NH3	B-H2O	AA	Y Ions	Y+2H	Y-NH3	Y-H2O	Y
1	164.1			146.1	Y	2,427.1	1,214.0	2,410.1	2,409.1	22
2	278.1		261.1	260.1	N	2,264.0	1,132.5	2,247.0	2,246.0	21
3	407.2		390.1	389.1	E	2,150.0	1,075.5	2,133.0	2,132.0	20
4	522.2		505.2	504.2	D	2,020.9	1,011.0	2,003.9	2,002.9	19
5	579.2		562.2	561.2	G	1,905.9	953.5	1,888.9	1,887.9	18
6	680.3	340.6	663.2	662.2	T	1,848.9	924.9	1,831.9	1,830.9	17
7	793.3	397.2	776.3	775.3	I	1,747.8	874.4	1,730.8	1,729.8	16
8	894.4	447.7	877.4	876.4	T	1,634.8	817.9	1,617.7	1,616.7	15
9	1,009.4	505.2	992.4	991.4	D	1,533.7	767.4	1,516.7	1,515.7	14
10	1,080.4	540.7	1,063.4	1,062.4	A	1,418.7	709.8	1,401.7	1,400.7	13
11	1,179.5	590.3	1,162.5	1,161.5	V	1,347.6	674.3	1,330.6	1,329.6	12
12	1,294.5	647.8	1,277.5	1,276.5	D	1,248.6	624.8	1,231.6	1,230.6	11
13	1,454.6	727.8	1,437.5	1,436.6	C+57	1,133.6	567.3	1,116.5	1,115.5	10
14	1,525.6	763.3	1,508.6	1,507.6	A	973.5	487.3	956.5	955.5	9
15	1,638.7	819.9	1,621.7	1,620.7	L	902.5	451.7	885.5	884.5	8
16	1,753.7	877.4	1,736.7	1,735.7	D	789.4	395.2	772.4	771.4	7
17	1,850.8	925.9	1,833.7	1,832.8	P	674.4	337.7	657.3	656.4	6
18	1,963.9	982.4	1,946.8	1,945.8	L	577.3		560.3	559.3	5
19	2,050.9	1,025.9	2,033.9	2,032.9	S	464.2		447.2	446.2	4
20	2,179.9	1,090.5	2,162.9	2,161.9	E	377.2		360.2	359.2	3
21	2,281.0	1,141.0	2,264.0	2,263.0	T	248.2		231.1	230.1	2
22	2,427.1	1,214.0	2,410.1	2,409.1	K	147.1		130.1	129.1	1

3

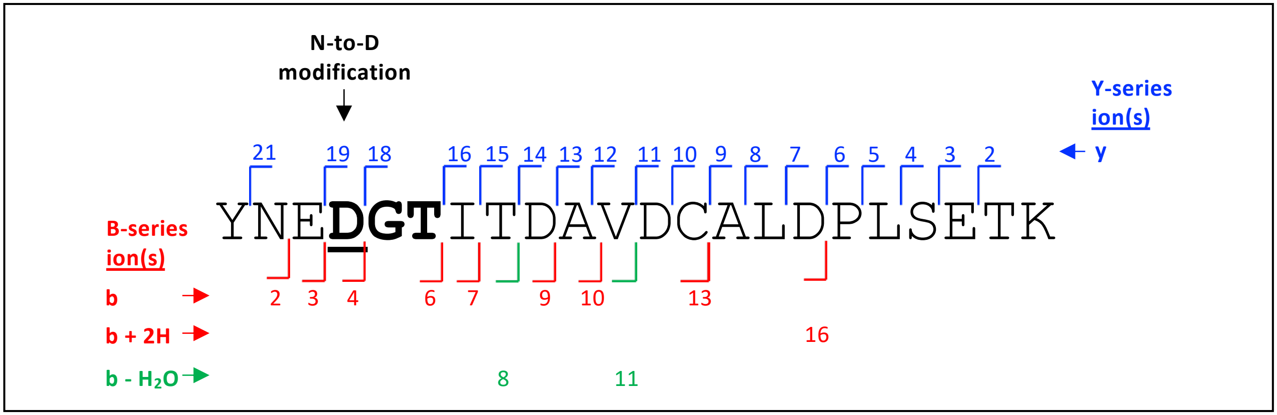

C

1

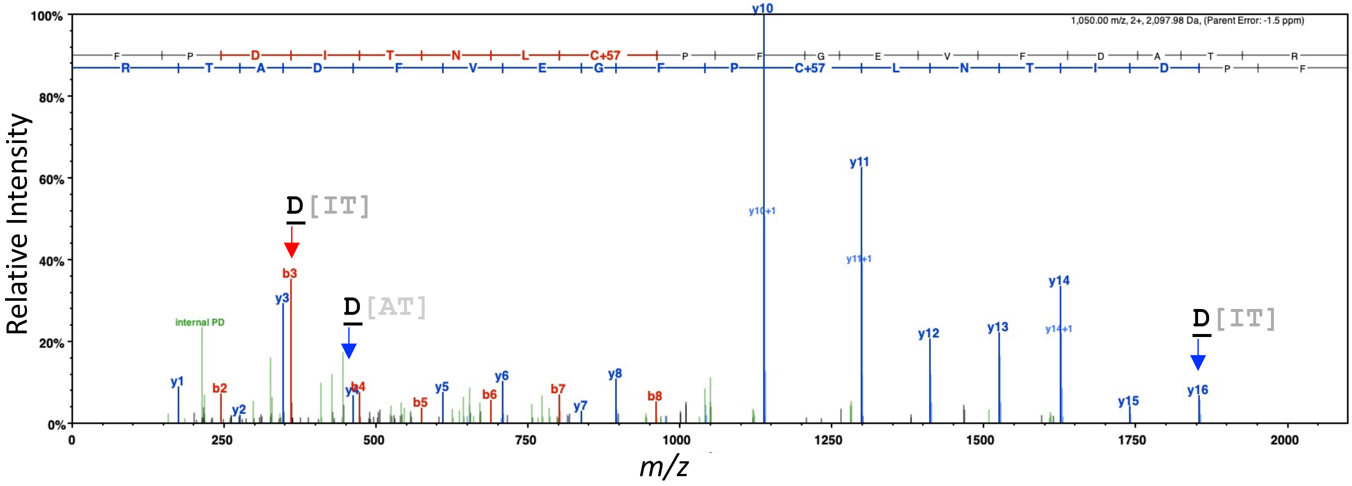

2

B	B Ions	B+2H	B-NH3	B-H2O	AA	Y Ions	Y+2H	Y-NH3	Y-H2O	Y
1	148.1				F	2,099.0	1,050.0	2,082.0	2,081.0	18
2	245.1				P	1,951.9	976.5	1,934.9	1,933.9	17
3	360.2			342.1	D	1,854.9	927.9	1,837.8	1,836.9	16
4	473.2			455.2	I	1,739.8	870.4	1,722.8	1,721.8	15
5	574.3			556.3	T	1,626.8	813.9	1,609.7	1,608.7	14
6	688.3	344.7	671.3	670.3	N	1,525.7	763.4	1,508.7	1,507.7	13
7	801.4	401.2	784.4	783.4	L	1,411.7	706.3	1,394.6	1,393.7	12
8	961.4	481.2	944.4	943.4	C+57	1,298.6	649.8	1,281.6	1,280.6	11
9	1,058.5	529.8	1,041.5	1,040.5	P	1,138.6	569.8	1,121.5	1,120.5	10
10	1,205.6	603.3	1,188.5	1,187.6	F	1,041.5	521.3	1,024.5	1,023.5	9
11	1,262.6	631.8	1,245.6	1,244.6	G	894.4	447.7	877.4	876.4	8
12	1,391.6	696.3	1,374.6	1,373.6	E	837.4	419.2	820.4	819.4	7
13	1,490.7	745.9	1,473.7	1,472.7	V	708.4	354.7	691.3	690.4	6
14	1,637.8	819.4	1,620.7	1,619.8	F	609.3		592.3	591.3	5
15	1,752.8	876.9	1,735.8	1,734.8	D	462.2		445.2	444.2	4
16	1,823.8	912.4	1,806.8	1,805.8	A	347.2		330.2	329.2	3
17	1,924.9	962.9	1,907.9	1,906.9	T	276.2		259.1	258.2	2
18	2,099.0	1,050.0	2,082.0	2,081.0	R	175.1		158.1		1

3

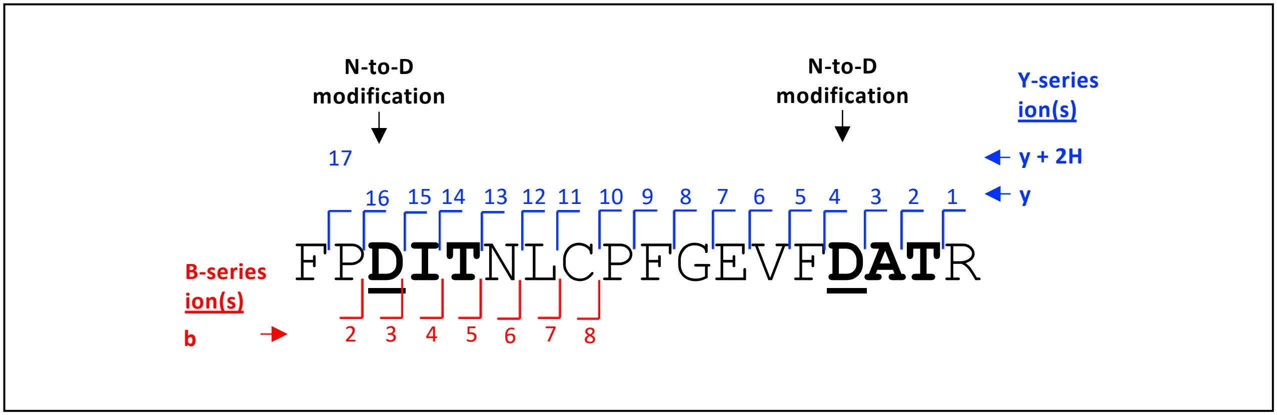

D

1

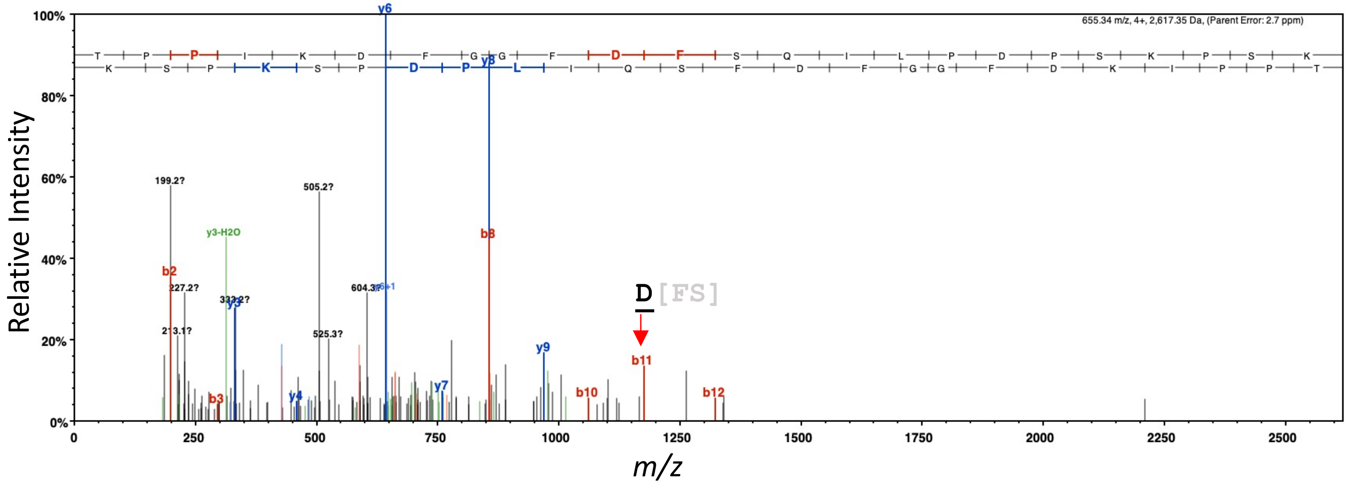

2

B	B Ions	B+2H	B-NH3	B-H2O	AA	Y Ions	Y+2H	Y-NH3	Y-H2O	Y
1	102.1	51.5		84.0	T	2,618.4	1,309.7	2,601.3	2,600.3	24
2	199.1	100.1		181.1	P	2,517.3	1,259.2	2,500.3	2,499.3	23
3	296.2	148.6		278.2	P	2,420.2	1,210.6	2,403.2	2,402.2	22
4	409.2	205.1		391.2	I	2,323.2	1,162.1	2,306.2	2,305.2	21
5	537.3	269.2	520.3	519.3	K	2,210.1	1,105.6	2,193.1	2,192.1	20
6	652.4	326.7	635.3	634.4	D	2,082.0	1,041.5	2,065.0	2,064.0	19
7	799.4	400.2	782.4	781.4	F	1,967.0	984.0	1,950.0	1,949.0	18
8	856.5	428.7	839.4	838.4	G	1,819.9	910.5	1,802.9	1,801.9	17
9	913.5	457.2	896.5	895.5	G	1,762.9	882.0	1,745.9	1,744.9	16
10	1,060.5	530.8	1,043.5	1,042.5	F	1,705.9	853.4	1,688.9	1,687.9	15
11	1,175.6	588.3	1,158.5	1,157.6	D	1,558.8	779.9	1,541.8	1,540.8	14
12	1,322.6	661.8	1,305.6	1,304.6	F	1,443.8	722.4	1,426.8	1,425.8	13
13	1,409.7	705.3	1,392.6	1,391.7	S	1,296.7	648.9	1,279.7	1,278.7	12
14	1,537.7	769.4	1,520.7	1,519.7	Q	1,209.7	605.3	1,192.7	1,191.7	11
15	1,650.8	825.9	1,633.8	1,632.8	I	1,081.6	541.3	1,064.6	1,063.6	10
16	1,763.9	882.5	1,746.9	1,745.9	L	968.5	484.8	951.5	950.5	9
17	1,861.0	931.0	1,843.9	1,842.9	P	855.5	428.2	838.4	837.4	8
18	1,976.0	988.5	1,959.0	1,958.0	D	758.4	379.7	741.4	740.4	7
19	2,073.0	1,037.0	2,056.0	2,055.0	P	643.4	322.2	626.4	625.4	6
20	2,160.1	1,080.5	2,143.0	2,142.1	S	546.3	273.7	529.3	528.3	5
21	2,288.2	1,144.6	2,271.1	2,270.1	K	459.3	230.1	442.3	441.3	4
22	2,385.2	1,193.1	2,368.2	2,367.2	P	331.2	166.1	314.2	313.2	3
23	2,472.2	1,236.6	2,455.2	2,454.2	S	234.1	117.6	217.1	216.1	2
24	2,618.4	1,309.7	2,601.3	2,600.3	K	147.1	74.1	130.1		1

3

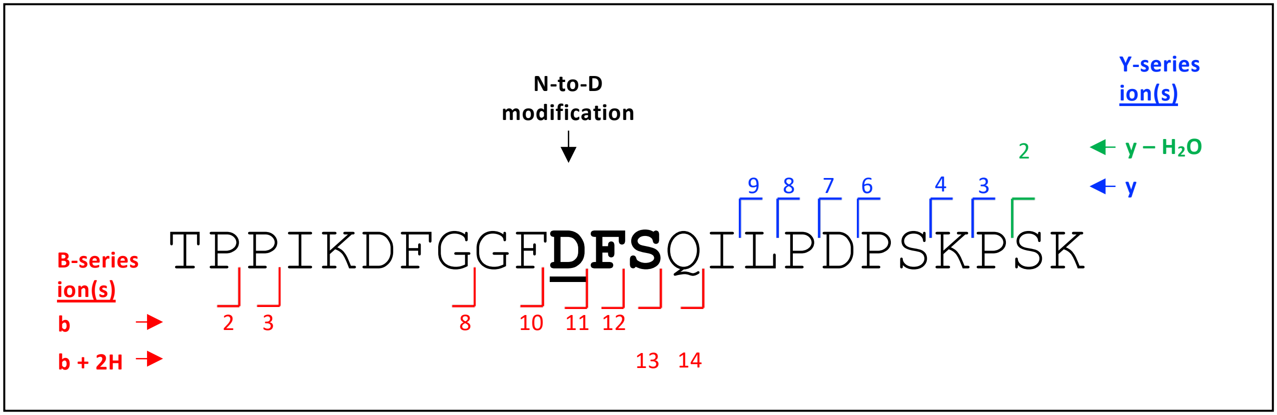

E

1

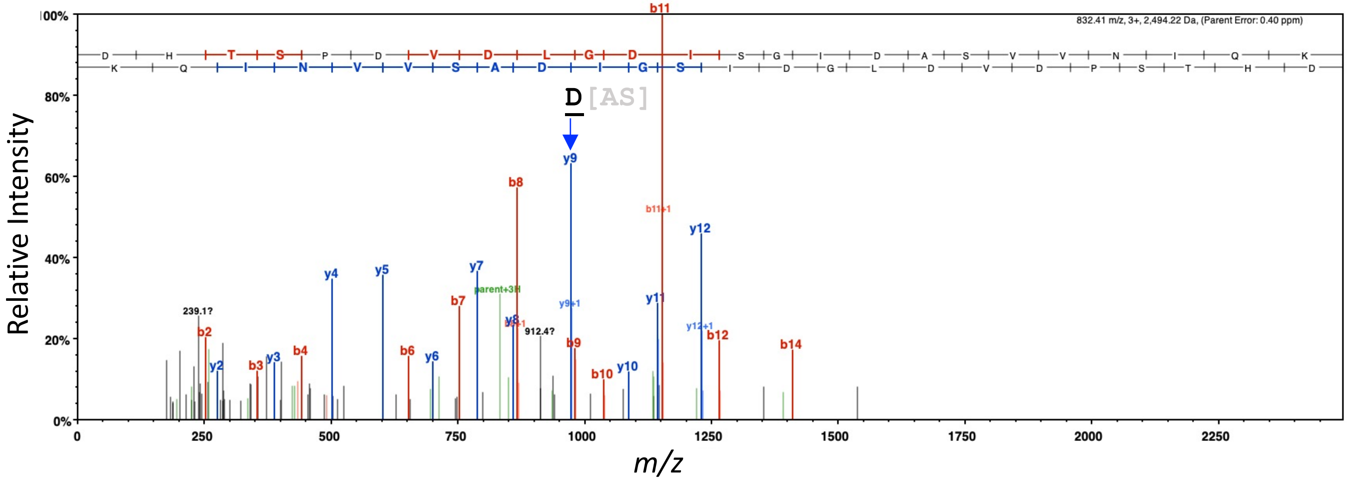

2

B	B Ions	B+2H	B-NH3	B-H2O	AA	Y Ions	Y+2H	Y-NH3	Y-H2O	Y
1	116.0	58.5		98.0	D	2,495.2	1,248.1	2,478.2	2,477.2	24
2	253.1	127.1		235.1	H	2,380.2	1,190.6	2,363.2	2,362.2	23
3	354.1	177.6		336.1	T	2,243.1	1,122.1	2,226.1	2,225.1	22
4	441.2	221.1		423.2	S	2,142.1	1,071.6	2,125.1	2,124.1	21
5	538.2	269.6		520.2	P	2,055.1	1,028.0	2,038.0	2,037.1	20
6	653.3	327.1		635.2	D	1,958.0	979.5	1,941.0	1,940.0	19
7	752.3	376.7		734.3	V	1,843.0	922.0	1,826.0	1,825.0	18
8	867.3	434.2		849.3	D	1,743.9	872.5	1,726.9	1,725.9	17
9	980.4	490.7		962.4	L	1,628.9	814.9	1,611.9	1,610.9	16
10	1,037.5	519.2		1,019.4	G	1,515.8	758.4	1,498.8	1,497.8	15
11	1,152.5	576.7		1,134.5	D	1,458.8	729.9	1,441.8	1,440.8	14
12	1,265.6	633.3		1,247.6	I	1,343.8	672.4	1,326.7	1,325.7	13
13	1,352.6	676.8		1,334.6	S	1,230.7	615.8	1,213.6	1,212.7	12
14	1,409.6	705.3		1,391.6	G	1,143.6	572.3	1,126.6	1,125.6	11
15	1,522.7	761.9		1,504.7	I	1,086.6	543.8	1,069.6	1,068.6	10
16	1,637.7	819.4		1,619.7	D	973.5	487.3	956.5	955.5	9
17	1,708.8	854.9		1,690.8	A	858.5	429.8	841.5	840.5	8
18	1,795.8	898.4		1,777.8	S	787.5	394.2	770.4	769.5	7
19	1,894.9	947.9		1,876.9	V	700.4	350.7	683.4		6
20	1,993.9	997.5		1,975.9	V	601.4	301.2	584.3		5
21	2,108.0	1,054.5	2,091.0	2,090.0	N	502.3	251.7	485.3		4
22	2,221.1	1,111.0	2,204.0	2,203.1	I	388.3	194.6	371.2		3
23	2,349.1	1,175.1	2,332.1	2,331.1	Q	275.2	138.1	258.1		2
24	2,495.2	1,248.1	2,478.2	2,477.2	K	147.1	74.1	130.1		1

3

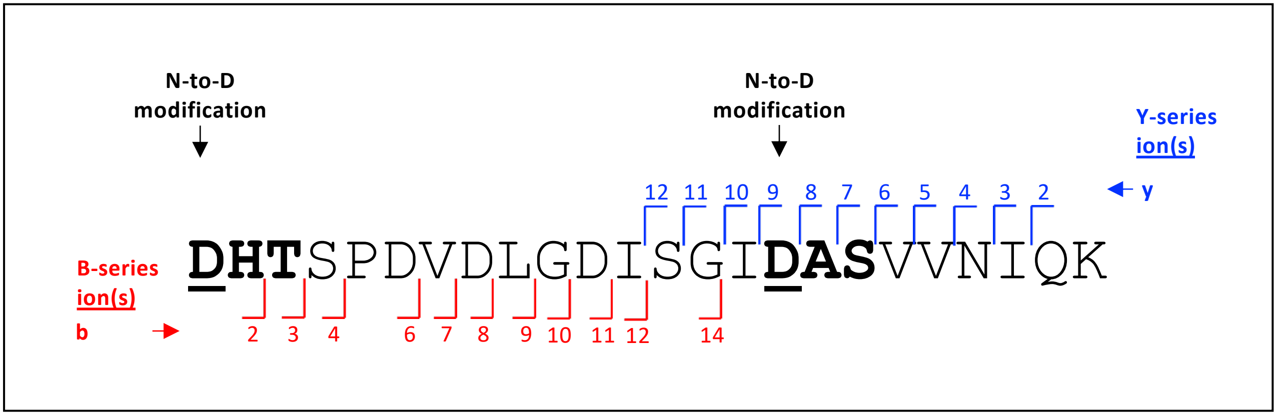

F

>tr|A0A7L9W8W9|A0A7L9W8W9_SARS2 Surface glycoprotein OS=Severe acute respiratory syndrome coronavirus 2 OX=2697049 GN=S PE=4 SV=1

```
QCV[1]NLTTRTQ   LPPAYTNSFT   RGVYYPDKVF   RSSVLHSTQD   LFLPFFN[2]NVT   WFHAIHVSGT   [3]NGTKRFDNPV   LPFNDGVYFA   STEKSNIIRG   90
WIFGTTLDSK   TQSLLIVN[4]NA   TNVVIKVCEF   QFCNDPFLGV   YYHKN[5]NKSWM   ESEFRVYSSA   N[6]NCTFEYVSQ   PFLMDLEGKQ   GNFKNLREFV   180
FKNIDGYFKI   YSKHTPINLV   RDLPQGFSAL   EPLVDLPIGI   [7]NITRFQTLLA   LHRSYLTPGD   SSSGWTAGAA   AYYVGYLQPR   TFLLKYNE[8]NG   270
TITDAVDCAL   DPLSETKCTL   KSFTVEKGIY   QTSNFRVQPT   ESIVRFP[9]NIT   NLCPFGEVF[10]N   ATRFASVYAW   NRKRISNCVA   DYSILYNSAS   360
FSTFKCYGVS   PTKLNDLCFT   NVYADSFVIR   GDEVRQIAPG   QTGKIADYNY   KLPDDFTGCV   IAWNSNNLDS   KVGGNYNYLY   RLFRKSNLKP   450
FERDISTEIY   QAGSTPCNGV   EGFNCYFPLQ   SYGFQPTNGV   GYQPYRVVVL   SFELLHAPAT   VCGPKKSTNL   VKNKCVNFNF   NGLTGTGVLT   540
ESNKKFLPFQ   QFGRDIADTT   DAVRDPQTLE   ILDITPCSFG   GVSVITPGT[11]N   TSNQVAVLYQ   GV[12]NCTEVPVA   IHADQLTPTW   RVYSTGSNVF   630
QTRAGCLIGA   EHV[13]NNSYECD   IPIGAGICAS   YQTQTNSPRR   ARSVASQSII   AYTMSLGAEN   SVAYS[14]NNSIA   IPT[15]NFTISVT   TEILPVSMTK   720
TSVDCTMYIC   GDSTECSNLL   LQYGSFCTQL   NRALTGIAVE   QDKNTQEVFA   QVKQIYKTPP   IKDFGGF[16]NFS   QILPDPSKPS   KRSFIEDLLF   810
NKVTLADAGF   IKQYGDCLGD   IAARDLICAQ   KFNGLTVLPP   LLTDEMIAQY   TSALLAGTIT   SGWTFGAGAA   LQIPFAMQMA   YRFNGIGVTQ   900
NVLYENQKLI   ANQFNSAIGK   IQDSLSSTAS   ALGKLQDVVN   QNAQALNTLV   KQLSSNFGAI   SSVLNDILSR   LDKVEAEVQI   DRLITGRLQS   990
LQTYVTQQLI   RAAEIRASAN   LAATKMSECV   LGQSKRVDFC   GKGYHLMSFP   QSAPHGVVFL   HVTYVPAQEK   [17]NFTTAPAICH   DGKAHFPREG   1080
VFVS[18]NGTHWF   VTQRNFYEPQ   IITTDNTFVS   GNCDVVIGIV   [19]NNTVYDPLQP   ELDSFKEELD   KYFK[20]NHTSPD   VDLGDISGI[21]N   ASVVNIQKEI   1170
DRLNEVAKNL   [22]NESLIDLQEL   GKYEQYIKWP   WYIWLGFIAG   LIAIVMVTIM   LCCMTSCCSC   LKGCCSCGSC   CKFDEDDSEP   VLKGVKLHYT   1260
```

>tr|A0A7L9W8W9|A0A7L9W8W9_NDM_SARS2 Surface glycoprotein OS=Severe acute respiratory syndrome coronavirus 2 OX=2697049 GN=S PE=4 SV=1_Mature protein with N-to-D modification in potential *N*-glycosylation sites (22)

Confirmed de-*N*-glycosylated peptides

```
QCV[1]DLTTRTQ   LPPAYTNSFT   RGVYYPDKVF   RSSVLHSTQD   LFLPFFD[2]DVT   WFHAIHVSGT   [3]DGTKRFDNPV   LPFNDGVYFA   STEKSNIIRG   90
WIFGTTLDSK   TQSLLIVN[4]DA   TNVVIKVCEF   QFCNDPFLGV   YYHKN[5]DKSWM   ESEFRVYSSA   N[6]DCTFEYVSQ   PFLMDLEGKQ   GNFKNLREFV   180
FKNIDGYFKI   YSKHTPINLV   RDLPQGFSAL   EPLVDLPIGI   [7]DITRFQTLLA   LHRSYLTPGD   SSSGWTAGAA   AYYVGYLQPR   TFLLKYNE[8]DG   270
TITDAVDCAL   DPLSETKCTL   KSFTVEKGIY   QTSNFRVQPT   ESIVRFP[9]DIT   NLCPFGEVF[10]D   ATRFASVYAW   NRKRISNCVA   DYSILYNSAS   360
FSTFKCYGVS   PTKLNDLCFT   NVYADSFVIR   GDEVRQIAPG   QTGKIADYNY   KLPDDFTGCV   IAWNSNNLDS   KVGGNYNYLY   RLFRKSNLKP   450
FERDISTEIY   QAGSTPCNGV   EGFNCYFPLQ   SYGFQPTNGV   GYQPYRVVVL   SFELLHAPAT   VCGPKKSTNL   VKNKCVNFNF   NGLTGTGVLT   540
ESNKKFLPFQ   QFGRDIADTT   DAVRDPQTLE   ILDITPCSFG   GVSVITPGT[11]D   TSNQVAVLYQ   GV[12]DCTEVPVA   IHADQLTPTW   RVYSTGSNVF   630
QTRAGCLIGA   EHV[13]DNSYECD   IPIGAGICAS   YQTQTNSPRR   ARSVASQSII   AYTMSLGAEN   SVAYS[14]DNSIA   IPT[15]DFTISVT   TEILPVSMTK   720
TSVDCTMYIC   GDSTECSNLL   LQYGSFCTQL   NRALTGIAVE   QDKNTQEVFA   QVKQIYKTPP   IKDFGGF[16]DFS   QILPDPSKPS   KRSFIEDLLF   810
NKVTLADAGF   IKQYGDCLGD   IAARDLICAQ   KFNGLTVLPP   LLTDEMIAQY   TSALLAGTIT   SGWTFGAGAA   LQIPFAMQMA   YRFNGIGVTQ   900
NVLYENQKLI   ANQFNSAIGK   IQDSLSSTAS   ALGKLQDVVN   QNAQALNTLV   KQLSSNFGAI   SSVLNDILSR   LDKVEAEVQI   DRLITGRLQS   990
LQTYVTQQLI   RAAEIRASAN   LAATKMSECV   LGQSKRVDFC   GKGYHLMSFP   QSAPHGVVFL   HVTYVPAQEK   [17]DFTTAPAICH   DGKAHFPREG   1080
VFVS[18]DGTHWF   VTQRNFYEPQ   IITTDNTFVS   GNCDVVIGIV   [19]DNTVYDPLQP   ELDSFKEELD   KYFK[20]DHTSPD   VDLGDISGI[21]D   ASVVNIQKEI   1170
DRLNEVAKNL   [22]DESLIDLQEL   GKYEQYIKWP   WYIWLGFIAG   LIAIVMVTIM   LCCMTSCCSC   LKGCCSCGSC   CKFDEDDSEP   VLKGVKLHYT   1260
```
