# Supplementary material for: Inhibition of Protein N-Glycosylation Blocks SARS-CoV-2 Infection
Source: mBio. 2022 Feb 15;13(1):e03718-21. doi: 10.1128/mbio.03718-21 (PMC8844921; doi:10.1128/mbio.03718-21)
Supplement: TEXT S1 [file mbio.03718-21-s0001.docx]

**Inhibition of protein *N-*glycosylation blocks SARS-CoV-2 infection**

Aitor Casas-Sanchez^1,2^*, Alessandra Romero-Ramirez^1,2^, Eleanor Hargreaves^3^, Cameron C. Ellis^4^, Brian I. Grajeda^4^, Igor Estevao^4^, Edward I. Patterson^1,2,5^, Grant L. Hughes^1,2^, Igor C. Almeida^4^, Tobias Zech^3^, Álvaro Acosta-Serrano^1,2^

^1^Department of Vector Biology and ^2^Department of Tropical Disease Biology, Liverpool School of Tropical Medicine, UK

^3^Department of Molecular and Cellular Physiology, University of Liverpool, UK

^4^Department of Biological Sciences, Border Biomedical Research Center, University of Texas at El Paso, TX, USA

^5^Department of Biological Sciences, Brock University, Canada

*Corresponding author: aitor.casas-sanchez@lstmed.ac.uk

SUPPLEMENTAL RESULTS

**Proteomics to identify deglycosylated spike sites.** Using high-resolution mass spectrometry, we identified the *N*-glycosylation sites that were susceptible to PNGase F treatment on the spike protein. To that end, purified virions produced by Vero E6 cells were subjected to treatment with PNGase F alone, which after cleavage converts the Asn (N) residues into Asp (D) (1). As negative controls for the N-to-D conversion, we used purified virions treated with heat-inactivated PNGase F, as well as purified virions alone or subjected to PNGase F mock treatment. Following treatment, samples were subjected to trypsin digestion and the resulting peptides were analyzed by LC-HR-MS/MS using a high-resolution 50-cm µPAC capLC C18 column directly coupled to the QE Plus MS. The identified proteins and peptides from SARS-CoV-2 (samples #1-4) following PD and Scaffold Q+ analyses are shown in **Supplemental Files S2-S5**. Clusters of spike (S), membrane (M), and nucleocapsid (N) proteins were identified (**Supplemental File S2**). Based on the normalized total spectra of each sample, N proteins were the major proteins found in all virion-containing samples, followed by spike and M proteins were of low abundance (**Supplemental File S2).** To locate the D residues resulting from PNGase F removal of *N*-glycans from asparagine residues, we used an in-house script that converted the N-to-D in all SARS CoV-2 proteins deposited in the UniProtKB database, including the 22 potential *N*-glycosylation sites in the spike protein. **Supplemental Files S3-S4** show all the identified spike proteins in the four samples. We included the complete sequence of the spike proteins highlighting the peptides identified by LC-HR-MS/MS to facilitate the visualization of the location of these peptides in each protein sequence and the protein sequence coverage. While no deglycosylated peptides were detected in positive control samples (purified virions alone and mock-treated purified virions) (**Supplemental File S3**), in purified virions treated with PNGase F we detected identified five peptides with N-to-D conversion (DLPQGFSALEPLVDLPIGI**DIT**R, YNE**DGT**ITDAVDcALDPLSETK, FP**DIT**NLcPFGEVF**DAT**R, TPPIKDFGGF**DFS**QILPDPSKPSK, and **DHT**SPDVDLGDISGI**DAS**VVNIQK) (**Supplemental File S4**). The MS/MS fragmentation and fragment-ion assignments of these peptides are shown in **Supplemental File S5**. Of particular interest, peptide FP**DIT**NLcPFGEVF**DAT**R (residues 329-346) is located within the spike RBD, which is essential for the virus binding to the ACE-2 human receptor. A BLASTp analysis of this peptide sequence showed that in all 5,000 spike protein sequences analyzed (maximum number of sequences allowed by BLASTp search), the sequence is fully conserved, strongly indicating that these two *N*-glycans could be essential for interaction with ACE2. Moreover, we found six peptide sequences in the purified virion sample treated with PNGase F (sample # 3) in which the N residue had not been substituted by D, thus indicating some S protein *N*-glycosylation sites remain unglycosylated (**Supplemental File S3**). It is important to underscore that PNGase F does not cleave complex *N*-glycans with a fucose residue α1-3-linked to the reducing GlcNAc linked to the N residue (1,2). This indicates that most likely the two cleaved complex-type *N*-glycans on N331 and N343 are modified with an α1-6-linked fucose residue. Finally, since part of the M protein appears to be *N*-glycosylated (**Figure 4D**) we also searched for any potential N-to-D conversion after PNGase F treatment. Intriguingly, we found that all tryptic peptide sequences identified in M were located near the C-terminus of the protein, but we failed to find peptide sequences indicative of an N-to-D conversion (**Supplemental File S4**). Of note, the only potential *N*-glycosylation sequon found in 763 M protein sequences analyzed through BLASTp and multiple sequence alignment was located at the N-terminus within an apparent ER signal peptide (SP) (e.g., MSDS**NGT**ITV..., MADS**NGT**ITV…), which could not be confirmed by SignalP 4.1 analysis (http://www.cbs.dtu.dk/services/SignalP-4.1/). Therefore, the shift in migration of M following *N*-glycan removal by PNGase F (**Figure 4D**) can only be explained if the non-canonical *N*-terminus SP of M proteins is not cleaved after protein synthesis in the ER and the *N*-glycan is eventually attached to it. This suggests that the N-terminus tryptic peptide containing the N-glycan is either further processed (e.g. cleaved or modified with another post-translational modification) or was somewhat altered during sample preparation.

**REFERENCES**

1. T. H. Plummer, J. H. Elder, S. Alexander, A. W. Phelan, A. L. Tarentino, Demonstration of peptide:N-glycosidase F activity in endo-beta-N-acetylglucosaminidase F preparations. *J. Biol. Chem.* **259**, 10700–10704 (1984).

2. F. Maley, R. B. Trimble, A. L. Tarentino, T. H. Plummer, Characterization of glycoproteins and their associated oligosaccharides through the use of endoglycosidases. *Anal Biochem*. **180**, 195–204 (1989).
